# Author Correction: A nanoengineered topical transmucosal cisplatin delivery system induces anti-tumor response in animal models and patients with oral cancer

**DOI:** 10.1038/s41467-022-35449-1

**Published:** 2022-12-21

**Authors:** Manijeh Goldberg, Aaron Manzi, Amritpreet Birdi, Brandon Laporte, Peter Conway, Stefanie Cantin, Vasudha Mishra, Alka Singh, Alexander T. Pearson, Eric R. Goldberg, Sam Goldberger, Benjamin Flaum, Rifat Hasina, Nyall R. London, Gary L. Gallia, Chetan Bettegowda, Simon Young, Vlad Sandulache, James Melville, Jonathan Shun, Sonya E. O’Neill, Erkin Aydin, Alex Zhavoronkov, Anxo Vidal, Atenea Soto, Maria Jose Alonso, Ari J. Rosenberg, Mark W. Lingen, Anil D’Cruz, Nishant Agrawal, Evgeny Izumchenko

**Affiliations:** 1 David H. Koch Institute for Integrative Cancer Research, Massachusetts Institute of Technology, Cambridge, MA USA; 2 Harvard-MIT Division of Health Sciences and Technology, Massachusetts Institute of Technology, Cambridge, MA USA; 3 Department of Biomedical Engineering, University of Massachusetts Lowell, Lowell, MA USA; 4 Privo Technologies, Peabody, MA USA; 5 Department of Medicine, Section of Hematology and Oncology, University of Chicago, Chicago, IL USA; 6 Department of Surgery, Section of Otolaryngology-Head and Neck Surgery, University of Chicago, Chicago, IL USA; 7 Department of Otolaryngology-Head and Neck Surgery, Johns Hopkins University School of Medicine, Baltimore, MD USA; 8 Department of Neurosurgery, Johns Hopkins University School of Medicine, Baltimore, MD USA; 9 Department of Neurosurgery and Oncology, Johns Hopkins University School of Medicine, Baltimore, MD USA; 10 Department of Oral Maxillofacial Surgery, The University of Texas Health Science Center at Houston, Houston, TX USA; 11 Department of Otolaryngology-Head & Neck Surgery, Baylor College of Medicine, Houston, TX USA; 12 Massachusetts College of Pharmacy and Health Sciences, Boston, MA USA; 13 Insilico Medicine, Pak Shek Kok, Hong Kong; 14 Department of Pharmacy and Pharmaceutical Technology, University of Santiago de Compostela, Galicia, Spain; 15 Department of Pathology, University of Chicago, Chicago, IL USA; 16 Department of Oncology, Apollo Hospital, Mumbai, India

**Keywords:** Chemotherapy, Oral cancer

Correction to: *Nature Communications* 10.1038/s41467-022-31859-3, published online 17 August 2022

In the original version of this Article, the following authors (Amritpreet Birdi, Brandon Laporte, Simon Young, Vlad Sandulache, James Melville, Jonathan Shun) were omitted from the author list.

Author list, affiliations, author contributions and competing interests sections have now been updated in both the PDF and HTML versions of the Article.

