# Supplementary material for: A nanoengineered topical transmucosal cisplatin delivery system induces anti-tumor response in animal models and patients with oral cancer
Source: Nat Commun. 2022 Aug 17;13:4829. doi: 10.1038/s41467-022-31859-3 (PMC9385702; doi:10.1038/s41467-022-31859-3)
Supplement: Supplementary file 3 — Reporting Summary [file 41467_2022_31859_MOESM3_ESM.pdf]

nature portfolio

Corresponding author(s): Evgeny Izumchenko
Manijeh Goldberg
Evgeny Izumchenko

Last updated by author(s): Jun 23, 2022

Reporting Summary

Nature Portfolio wishes to improve the reproducibility of the work that we publish. This form provides structure for consistency and transparency in reporting. For further information on Nature Portfolio policies, see our Editorial Policies and the Editorial Policy Checklist.

Statistics

For all statistical analyses, confirm that the following items are present in the figure legend, table legend, main text, or Methods section.

n/a	Confirmed	
☐	☒	The exact sample size (*n*) for each experimental group/condition, given as a discrete number and unit of measurement
☐	☒	A statement on whether measurements were taken from distinct samples or whether the same sample was measured repeatedly
☐	☒	The statistical test(s) used AND whether they are one- or two-sided<br>*Only common tests should be described solely by name; describe more complex techniques in the Methods section.*
☐	☒	A description of all covariates tested
☐	☒	A description of any assumptions or corrections, such as tests of normality and adjustment for multiple comparisons
☐	☒	A full description of the statistical parameters including central tendency (e.g. means) or other basic estimates (e.g. regression coefficient) AND variation (e.g. standard deviation) or associated estimates of uncertainty (e.g. confidence intervals)
☐	☒	For null hypothesis testing, the test statistic (e.g. *F*, *t*, *r*) with confidence intervals, effect sizes, degrees of freedom and *P* value noted<br>*Give P values as exact values whenever suitable.*
☒	☐	For Bayesian analysis, information on the choice of priors and Markov chain Monte Carlo settings
☒	☐	For hierarchical and complex designs, identification of the appropriate level for tests and full reporting of outcomes
☒	☐	Estimates of effect sizes (e.g. Cohen's *d*, Pearson's *r*), indicating how they were calculated

Our web collection on statistics for biologists contains articles on many of the points above.

Software and code

Policy information about availability of computer code

Data collection	Platinum quantification was performed using Perkin Elmer Nexion 350X inductively coupled plasma mass spectrometry (ICP-MS) instrument. Immunohistochemical staining was performed using automated Ventana Benchmark station and slides were digitalized using Aperio (Leica Biosystems) scanner. Fluorescent images were taken using EVOS FLoid imaging systems (ThermoFisher).
Data analysis	Statistical analyses were conducted using GraphPad Software (version is 9.4.0). ImageJ software (ersion 1.53r) was used for immunohistochemical analysis.

For manuscripts utilizing custom algorithms or software that are central to the research but not yet described in published literature, software must be made available to editors and reviewers. We strongly encourage code deposition in a community repository (e.g. GitHub). See the Nature Portfolio guidelines for submitting code & software for further information.

Data

Policy information about availability of data

All manuscripts must include a data availability statement. This statement should provide the following information, where applicable:

- Accession codes, unique identifiers, or web links for publicly available datasets
- A description of any restrictions on data availability
- For clinical datasets or third party data, please ensure that the statement adheres to our policy

The authors declare that the data supporting the findings of this study are available within the article and its supplementary information files.

Human research participants

Policy information about studies involving human research participants and Sex and Gender in Research.

Reporting on sex and gender	Both men and women over age of 18 were included, and there was no preferential selection requirement by gender. All participants regardless of race or ethnicity were eligible to participate in this study.
Population characteristics	Five male and five female patients with OCSCC (average age: 64.3) were recruited to the study. Detailed clinicopathological and demographic patients' characteristics are summarized in Tables 1 and 2 of the manuscript.
Recruitment	Key eligibility criteria included pathologically and clinically confirmed T1 (≤2 cm) or T2 (≥2 cm but ≤4 cm) SCC of the mucosal lip or oral cavity amenable to surgical resection. Previous radiation for head and neck cancer was excluded. Additional criteria included ECOG performance status of ≤2, adequate renal function, and absence of serious underlying medical conditions which could impair the ability of the subject to participate in the study. Patients were enrolled between August 30th 2018 and May 6th 2020. Detailed description of the inclusion/exclusion criteria is provided in the clinical trial protocol submitted as supplementary material and at: https://clinicaltrials.gov/ct2/show/NCT03502148. There were no self-selection bias or any other biases during the recruitment that are likely to impact results.
Ethics oversight	This study was approved by the IRB representing the participating institution and registered on clinicaltrials.gov (NCT03502148; https://clinicaltrials.gov/ct2/show/NCT03502148). Written informed consent was obtained from each subject prior to performing any study-related procedures. The trial was designed and monitored in accordance with the Good Clinical Practice (GCP) principles and the Declaration of Helsinki.

Note that full information on the approval of the study protocol must also be provided in the manuscript.

Field-specific reporting

Please select the one below that is the best fit for your research. If you are not sure, read the appropriate sections before making your selection.

☒ Life sciences ☐ Behavioural & social sciences ☐ Ecological, evolutionary & environmental sciences

For a reference copy of the document with all sections, see nature.com/documents/nr-reporting-summary-flat.pdf

Life sciences study design

All studies must disclose on these points even when the disclosure is negative.

Sample size	Sample size determination for animal experiments: The sample size of 6 animals per treatment group was considered adequate as it provides over 80% power to detect halving tumor growth when comparing two groups, assuming a 2-tailed, 2-sample t test with alpha of 0.05.<br><br>Sample size pre-determination rationale for human trial: The planned enrollment for the final dose is intended to obtain N=11 evaluable subjects. Based on Simon's 2-stage procedure, the null hypothesis that the true response rate is 0.30 will be tested against a 1-sided alternative. In the first stage, 5 evaluable subjects will be accrued. If there are 2 or fewer responses in these 5 subjects, the dose will be escalated. Otherwise, the dose will remain the same or de-escalated based on safety data. Additional subjects will be accrued for a total of up to 11 evaluable subjects at the final dose. The null hypothesis will be rejected if 7 or more responses are observed at the final dose. This design yields a type I error rate of 0.018 and power of 96.44% when the true response rate is 0.85.
Data exclusions	No data were excluded from the analyses
Replication	For all experiments using tissue samples derived from tumor bearing animals (mice and hamsters) all procedures and measurements (detection of platinum in blood and tissue samples, IHC staining, and permeation analysis) were replicated in at least 3 animals per group. This study does not include experiments other than those involving animals and human subjects.
Randomization	Mice and hamsters were randomized into treatment groups using blinded block randomization. This study does not include experiments other than those involving animals and human subjects.
Blinding	For animal experiments: The researcher performing tumor measurements and histological quantifications was blinded to group allocation.<br>For human trial: No blinding procedures were used because this was an open-label, single arm study.<br>This study does not include experiments other than those involving animals and human subjects.

Reporting for specific materials, systems and methods

We require information from authors about some types of materials, experimental systems and methods used in many studies. Here, indicate whether each material, system or method listed is relevant to your study. If you are not sure if a list item applies to your research, read the appropriate section before selecting a response.

Materials & experimental systems

n/a	Involved in the study
☐	☒ Antibodies
☐	☒ Eukaryotic cell lines
☒	☐ Palaeontology and archaeology
☐	☒ Animals and other organisms
☐	☒ Clinical data
☒	☐ Dual use research of concern

Methods

n/a	Involved in the study
☒	☐ ChIP-seq
☒	☐ Flow cytometry
☒	☐ MRI-based neuroimaging

Antibodies

Antibodies used	anti-CD3 (1:100, Ventana, 790-4341), anti-CD8 (1:100, Ventana, 790–4460) and anti-CD4 (1:100, Ventana, 790-4423).
Validation	All antibodies have been comprehensively validated by the manufacturer and in the literature for use in human immunohistochemistry. Detailed validation (such as analytical performance in multiple human tissues and across various pathological conditions, including head and neck cancer) for each antibody used in this study is available on the manufacturer's web-page.

Eukaryotic cell lines

Policy information about cell lines and Sex and Gender in Research

Cell line source(s)	FaDu cell line was obtained from ATCC. HCPC-1 cell line was kindly provided by the founder, Dr. Shklar (Harvard School of Dental Medicine, Boston, MA, USA).
Authentication	Sort Tandem Repeat (STR) fingerprinting was performed to ensure authenticity.
Mycoplasma contamination	Cell cultures were routinely tested for mycoplasma contamination and were negative before usage.
Commonly misidentified lines (See ICLAC register)	No commonly misidentified lines were used

Animals and other research organisms

Policy information about studies involving animals; ARRIVE guidelines recommended for reporting animal research, and Sex and Gender in Research

Laboratory animals	Athymic nude mice (6–8 weeks old) and golden Syrian hamster (8 weeks old) were purchased from Charles River Laboratories (Wilmington, MA, USA) . Animals were kept in a pathogen-free facility, with 12 light/12 dark cycle, 23°C, and 40-50% humidity. Males and females were used in this study.
Wild animals	No wild animals were used.
Reporting on sex	Animals were randomized at a 1:1 male/female ratio to account for any biological variables between the sexes that may impact treatment outcome.
Field-collected samples	No field-collected animals were used.
Ethics oversight	All animal procedures were performed in accordance with National Institutes of Health Animal Care guidelines and Massachusetts Institute of Technology, Division of Comparative Medicine requirements, under a protocol 1112-115-15 (2014) approved by the Institutional Animal Care and Usage Committee.

Note that full information on the approval of the study protocol must also be provided in the manuscript.

Clinical data

Policy information about clinical studies

All manuscripts should comply with the ICMJEguidelines for publication of clinical research and a completedCONSORT checklist must be included with all submissions.

Clinical trial registration	NCT03502148
Study protocol	A redacted version of the protocol was sent to Nature Communications
Data collection	Data collection sites:<br>* Advanced ENT and Allergy, Louisville, Kentucky,<br>* University of Cincinnati Cancer Institute, Cincinnati, Ohio<br>* Ben Taub Hospital, Houston, Texas<br>* Memorial Hermann Hospital, Houston, Texas

	* The University of Texas Health Science Center School of Dentistry, Houston, Texas<br>Actual start date: June 19, 2018<br>Actual completion date: May 6, 2020
Outcomes	Primary Outcome Measures: Determination of a safe and efficacious dose of PRV111 based on the incidence of adverse events and tumor response. Secondary Outcome Measures: (i) Evaluate tumor volume shrinkage assessed at baseline and at the time of surgical excision, (ii) Assess long-term safety - determine safety during post surgery follow-up visits by review of laboratory values and adverse events graded by CTCAE. (iii) Evaluate systemic, tumor and lymph node (if available) platinum levels following PRV111 administration assessed using bioanalytical methods. (iv) Evaluate device technical success including the residual cisplatin level in the patch after application - evaluate patch adhesion during application and residual cisplatin level post administration by bioanalytical methods.
