# Supplementary material for: A nanoengineered topical transmucosal cisplatin delivery system induces anti-tumor response in animal models and patients with oral cancer
Source: Nat Commun. 2022 Aug 17;13:4829. doi: 10.1038/s41467-022-31859-3 (PMC9385702; doi:10.1038/s41467-022-31859-3)
Supplement: Supplementary file 1 — Supplementary Information [file 41467_2022_31859_MOESM1_ESM.pdf]

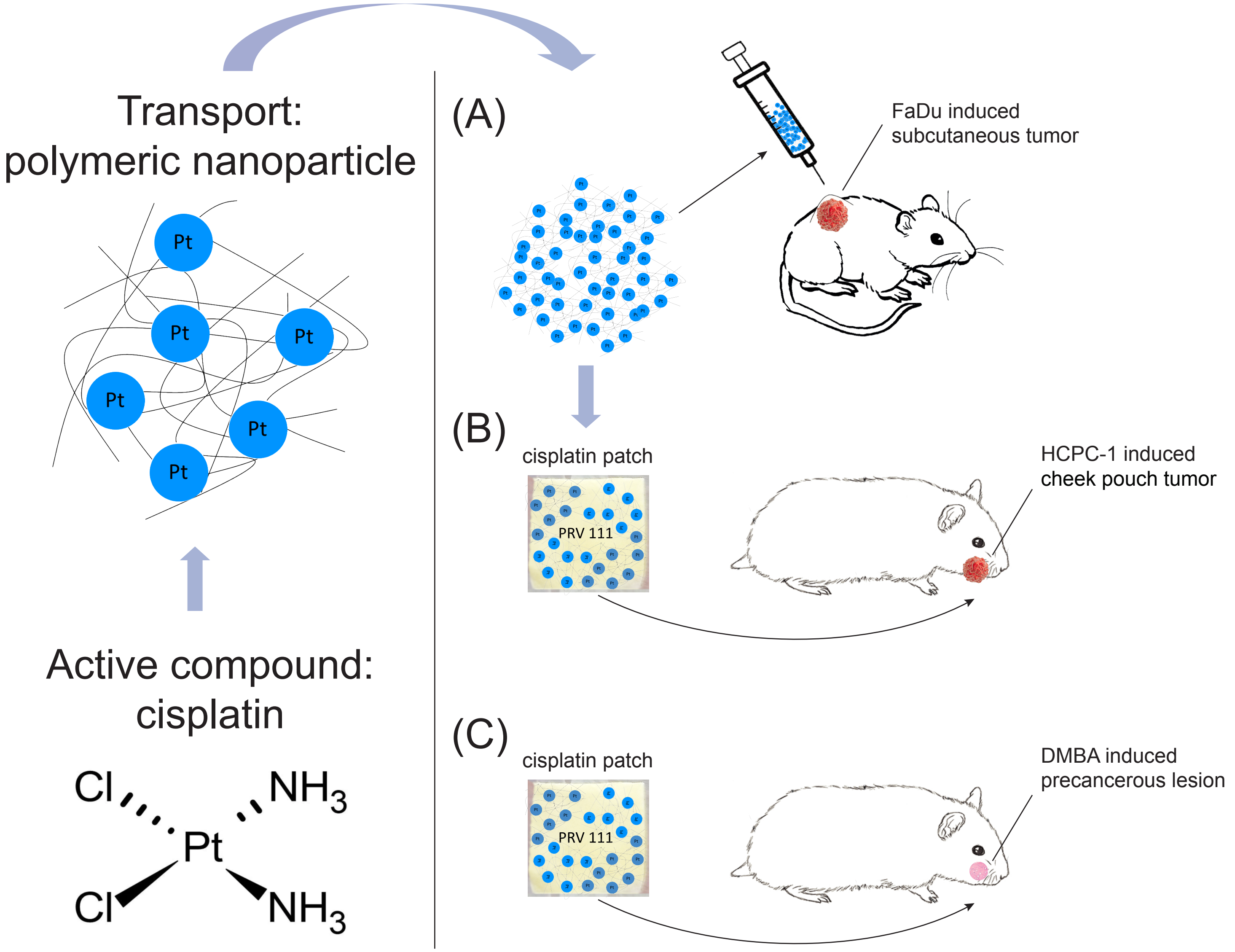

Supplementary Figure 1. Schematic representation of the animal models used in this study. **A.** Immunodeficient nude mice bearing subcutaneous human HNSCC FaDu cell line induced tumors treated with IT administration of CDDP-NPs. **B.** Golden Syrian hamsters bearing orthotopic cheek pouch tumors induced by HCPC-1 cell line, treated with PRV111. **C.** Golden Syrian hamsters bearing carcinogen induced premalignant lesions, treated with PRV111.

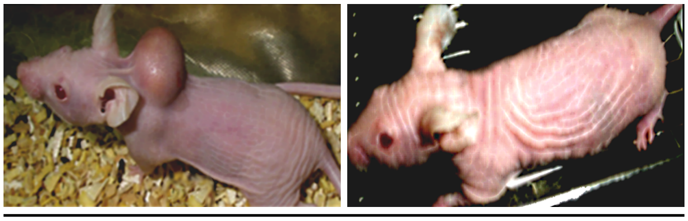

Supplementary Figure 2. Representative images of mice treated with either BLK-NP (n=4) or CDDP-NP (n=5) taken after 5 treatment rounds (at day 18).

A.

Hamsters under the treatment with PRV111

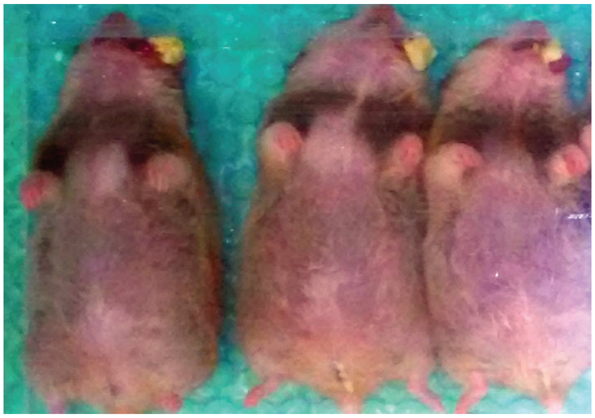

B.

Day 0 BLK-patch PRV111

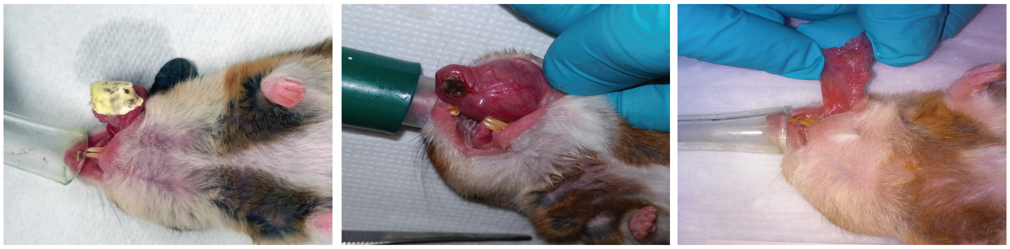

After 4 treatments (day 15)

Supplementary Figure 3. Images of hamsters treated with PRV111 of BLK patch. **A.** Image of hamsters under anesthesia with PRV111 patch applied topically on the lesion surface (3 of 6 animals shows). Moist gauze was placed on the exposed backing side of the patch to keep it hydrated during the treatment period to allow proper drug release. **B.** Representative images of tumor bearing animals (6 per group): left - baseline cheek pouch tumor (note a mucoadhesive PRV111 patch embracing the tumor), middle - cheek pouch tumor treated with BLK-patch for 15 days, right - cheek pouch tumor treated with PRV111 for 15 days.

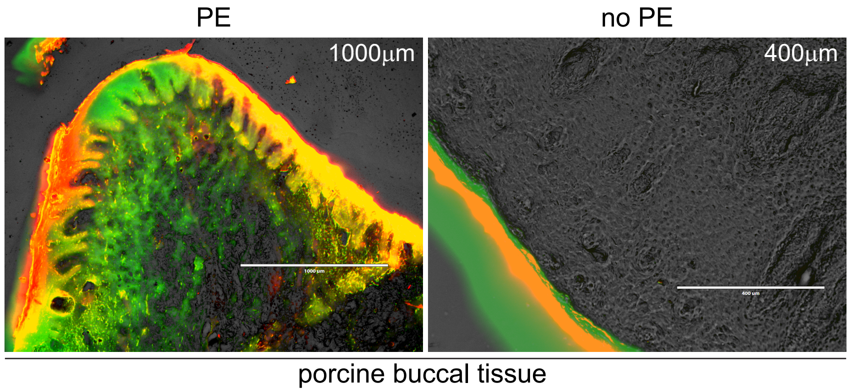

Supplementary Figure 4. Representative fluorescence images (n=5) of the freshly harvested normal porcine buccal tissue sections taken after treatment of the specimen with PRV111 patch containing chitosan particles labeled with Cy5 (red) and encapsulating FITC (green). *Right* – PE was applied prior to PRV111; *left* – PE was not applied.

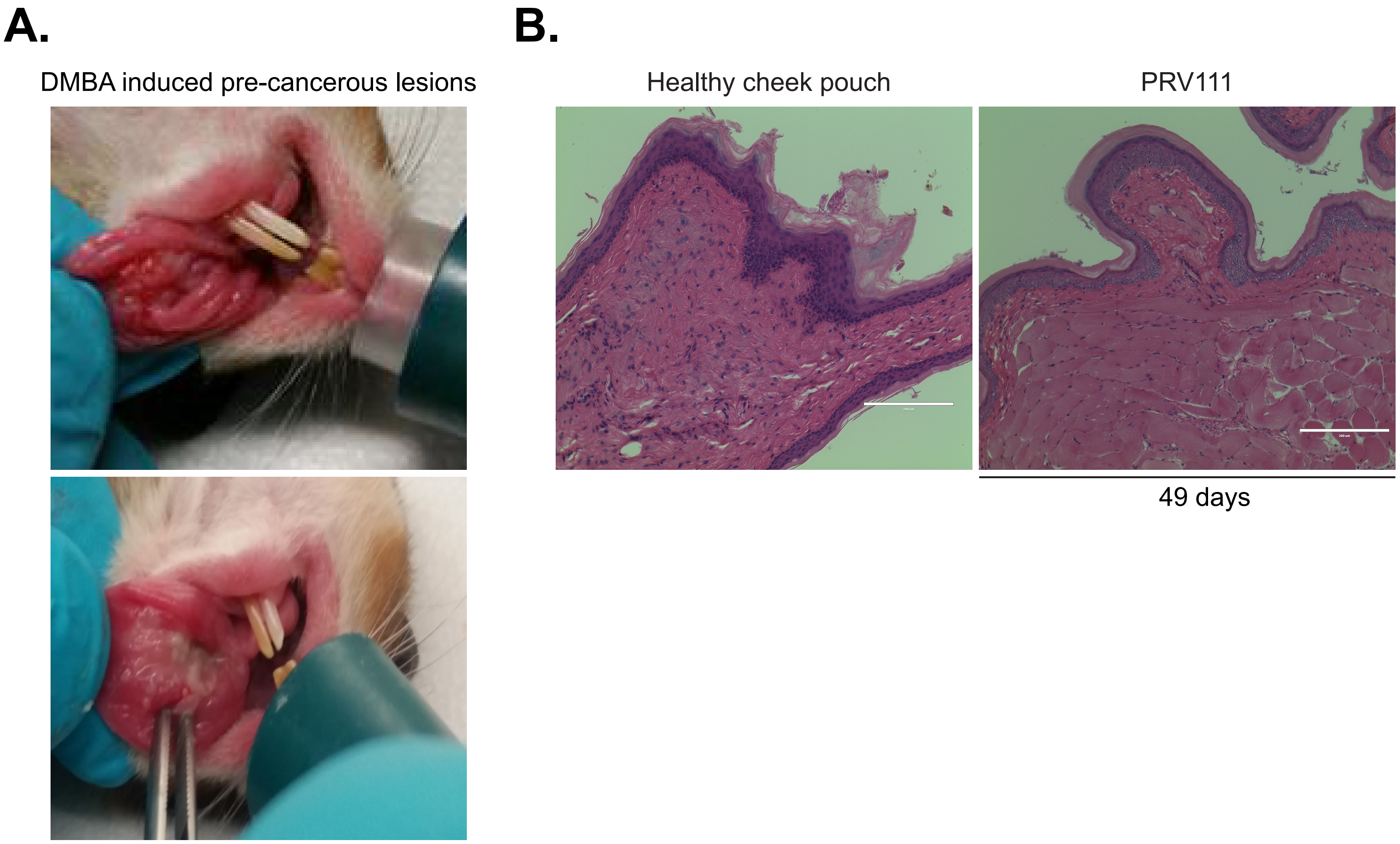

Supplementary Figure 5. A. Representative images of DMBA induced pre-cancerous lesion (n=9). **B.** Representative H&E stained histopathological images (n=3 per group) of a healthy cheek pouch (left) and cheek pouch harvested from hamster treated with PRV111 (right). Scale bar – 200um.

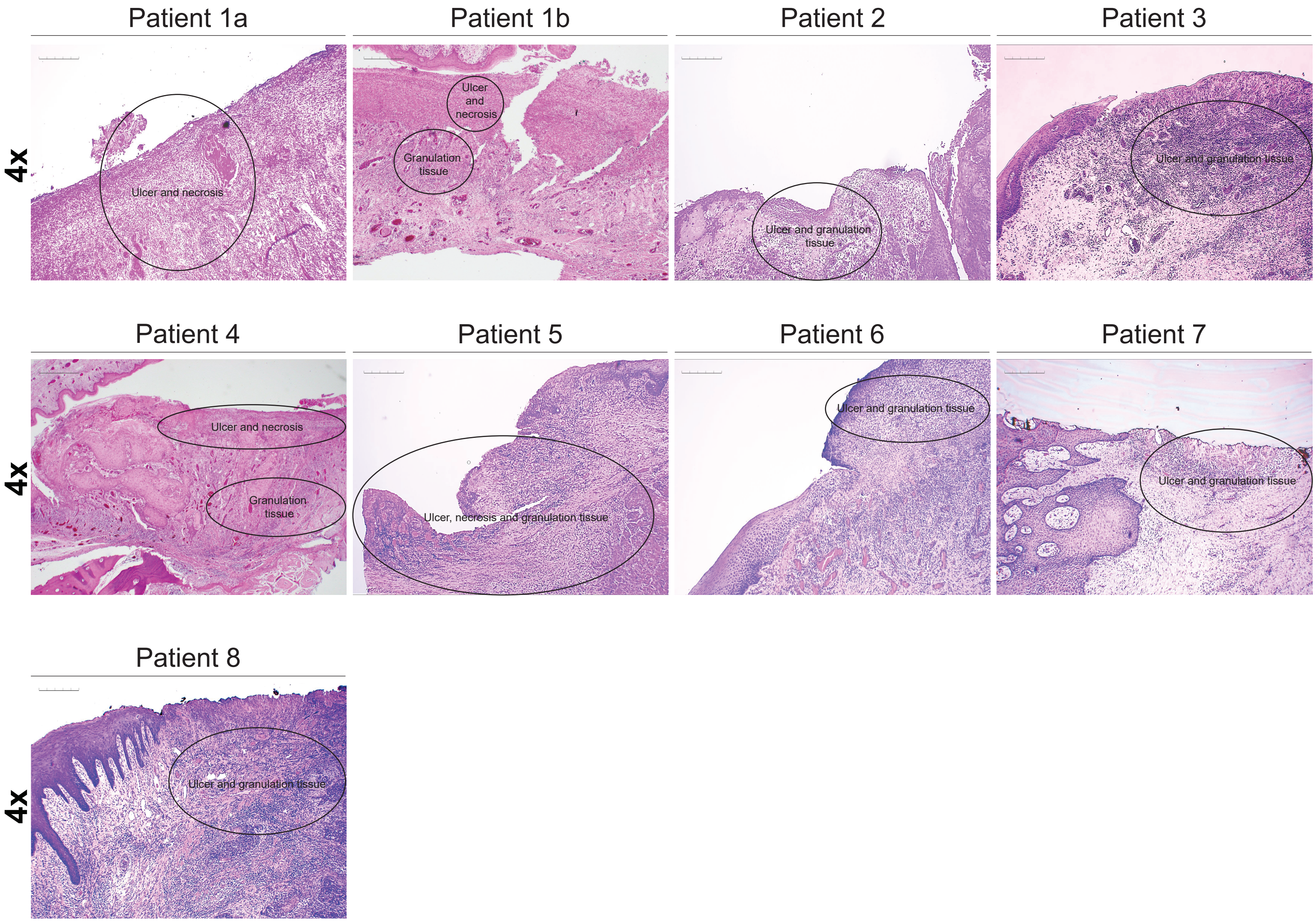

Supplementary Figure 6. Representative H&E stained histologic images (4×; scale bar – 1000um) of tumor bed areas post-PRV111 treatment demonstrate marked ulceration, necrosis and granulation tissue formation at the site of patch placement.

Supplementary Table 1: Power and sample size

Null response rate: P_0	Alt. response rate: P_1	Alpha one-tailed	Power	n1	r1	N	R
0.30	0.85	0.018	96.44%	5	2	11	6

N - total number of evaluable subjects at the final dose.
n1 - number of evaluable subjects accrued during Stage 1.
If r1 or fewer responses were observed during Stage 1, escalate the dose if safety requirements were met.
If R or fewer responses are observed by the end of Stage 2 in N subjects, then an effective dose was not found.

Supplementary Table 2: Device performance – peel and adhesion summary

**Subject ID**<br>**Sex**	**Total Number of Patches Applied**	**Number of Patches with Peel Issues**	**Number of Patches with Adhesion Issues**
**1**<br>F	24	0	0
**2**<br>F	24	0	0
**3**<br>M	12	0	1
**4**<br>M	24	0	2
**5**<br>F	12	0	2
**6**<br>M	12	0	0
**7**<br>F	24	0	0
**8**<br>F	12	0	0
**9**<br>M	24	0	0
**10**<br>M	14	0	0
**Total patches used**	**182**	**0**	**5**
Percentage of patches with peel issues	**0%**		
Percentage of patches with adhesion issues	**2.75%**		

F=female; ID=identification; M=male;

Supplementary Table 3: Residual cisplatin platinum (Pt) in the PRV111 patch

.

Subject	% Residual Patch Pt [μg/g]
1	6.9%
2	5.4%
3	9.4%
4	7.9%
5	9.7%
6	7.3%
7	5.4%
8	9.1%
9	13.4%
10	8.8%
**Average**	**8.3%**

Pt=platinum

Supplementary Table 4: Percent mean residual drug in PRV111 patches by treatment visit

**Patch Applied**	**% Mean Remaining**	**% Standard Deviation**
Visit 1, Application 1	8.71	4.17
Visit 1, Application 2	9.58	2.89
Visit 1, Application 3	9.09	4.43
Visit 2, Application 1	7.15	3.65
Visit 2, Application 2	8.62	2.84
Visit 2, Application 3	8.14	2.96
Visit 3, Application 1	7.72	2.74
Visit 3, Application 2	8.52	3.72
Visit 3, Application 3	7.45	3.87
Visit 4, Application 1	8.26	2.74
Visit 4, Application 2	7.79	1.97
Visit 4, Application 3	7.99	2.14

182 patches applied in total.

Supplementary Table 5: Tumor volume percent change from baseline by subject

Subject Number<br>Sex	Tumor<br>Volume based on first measurement<br>(mm^3)	Tumor Volume<br>Based on last measurement (V5)<br>(mm^3)	%Change from Baseline
**1**<br>F	(Connected tumor) 292	22	-93%
	(Connected tumor) 21	0	-100%
**2**<br>F	837	419	-50%
**3**<br>M	18	0	-100%
**4**<br>M	1231	14	-99%
**5**<br>F	691	65	-91%
**6**<br>M	1978	1282	-35%
**7**<br>F	432	4	-99%
**8**<br>F	8	12	+50%
**Average**	**612**	**202**	**69**

M=Male, F=Female.

Supplementary Table 6: Clinical response by total tumor volume change by visit

Visit Number<br>Category [a]	(N=8)<br>n (%)	p-value [b]
**Visit 2**		
Response	2 (25.0)	0.4482
Complete	0	
Strong	1	
Partial	1	
Non-Response	6 (75.0)	
Stable Disease	6	
Progressive Disease	0	
**Visit 3**		
Response	6 (75.0)	0.0113
Complete	0	
Strong	4	
Partial	2	
Non-Response	2 (25.0)	
Stable Disease	2	
Progressive Disease	0	
**Visit 4**		
Response	7 (87.5)	0.0013
Complete	1	
Strong	4	
Partial	2	
Non-Response	1 (12.5)	
Stable Disease	0	
Progressive Disease	1	
**Visit 5**		
Response	7 (87.5)	0.0013
Complete	1	
Strong	4	
Partial	2	
Non-Response	1 (12.5)	
Stable Disease	0	
Progressive Disease	1	

[a] Response category based on total tumor volume percent change from baseline, appearance of new lesion(s) was categorized as progressive disease regardless of the total tumor volume change.
[b] p-value based on one-sided test of response rate less than 30%.

Supp. Table 7: Treatment-related treatment-emergent adverse events during the patch treatment period by system organ class and preferred term

**MedDRA System Organ Class**<br>**Preferred Term**	**Safety Population**<br>**(N=10)**<br>**n (%)**
Subjects with any TEAE	8 (80.0)
Gastrointestinal disorders	8 (80.0)
Dry mouth	1 (10.0)
Gastrooesophageal reflux disease	1 (10.0)
Glossodynia	3 (30.0)
Lip blister	2 (20.0)
Nausea	1 (10.0)
Oral mucosal blistering	2 (20.0)
Oral pain	4 (40.0)
Oropharyngeal pain	1 (10.0)
Salivary duct inflammation	1 (10.0)
Stomatitis	2 (20.0)
Swollen tongue	1 (10.0)
Tongue blistering	2 (20.0)
Tongue discomfort	2 (20.0)
Tongue ulceration	1 (10.0)
Toothache	1 (10.0)
Nervous system disorders	1 (10.0)
Headache	1 (10.0)

MedDRA=Medical Dictionary for Regulatory Activities; TEAE=treatment-emergent adverse event

Supplementary Table 8. Results of clinical safety assessments for cisplatin-related toxicity

**Presence of known cisplatin toxicities (when administered systemically) (Yes/No)**			
Subject	Ototoxicity	Renal Dysfunction	Neuropathy
1	No	No	No
2	No	No	No
3	No	No	No
4	No	No	No
5	No	No	No
6	No	No	No
7	No	No	No
8	No	No	No
9	No	No	No
10	No	No	No

Supplementary Table 9. Results of complete blood count (CBC) clinical safety labs.

Clinically significant change in circulating immune cells/white blood cells (Yes/No)						
Subject	Basophils Eosinophils	Erythrocytes Monocytes	Leukocytes Neutrophils	Lymphocytes	Platelets	Reticulocytes
1	No	No	No	No	No	No
2	No	No	No	No	No	No
3	No	No	No	No	No	No
4	No	No	No	No	No	No
5	No	No	No	No	No	No
6	No	No	No	No	No	No
7	No	No	No	No	No	No
8	No	No	No	No	No	No
9	No	No	No	No	No	No
10	No	No	No	No	No	No

Supplementary Table 10. Summary of lymph nodes in which cisplatin platinum was measured.

Patient	Baseline CT Swollen/Suspicious nodes	Node Location	Pt in Lymph [ug/g]	Cancer in nodes where Pt was detected	Cancer in other lymph nodes
1	Suspicious lymph nodes in the right neck. Several asymmetrically prominent right submandibular lymph nodes are noted	Not provided	54	No	No
2	None noted	Not provided	40	No	No
3	None noted	I1	304	No	No
4	None noted	C2	80	No	No
5	None noted	I3	245	No	No
6	Several <1cm nodes noted bilaterally	D2, D12	D2 - 98<br>D12 - 61	No	No
7	None noted	N/A	N/A	N/A	N/A
8	Tumor on RT side, Suspicious nodes (At least 3); Right L2; Left L2/L3	G1, I2	Right G1 – 183<br>Left I2 - 106	No	Yes – Right L2 (1/6)<br>Yes – Left L3 (6/16)<br>Yes – Left L4 (1/1)
9	N/A	Not provided	11	N/A	Yes
10	Suspicious lymph nodes noted	Not provided	29	N/A	Yes

Pt=platinum

Supplementary Table 11. Distribution of cisplatin platinum in tumor tissue and serum following PRV111 patch administration.

Subject	Concentration in Tumor [μg/g]	Concentration in Lymph Nodes [μg/g]	C_{max} Pt in Serum [μM]
1	261	54	0.27
2	134	40	0.62
3	1395	305	0.16
4	201	80	0.06
5	425	246	0.27
6	167	80	0.08
7	66	--	0.21
8	471	145	0.26
9	215	11	0.09
10	33	29	0.35
**Average**	**336.8**	**110**	**0.24**

C_{max}=maximum concentration; Pt=platinum

Supplementary Note 1

Privo Technologies
Tougher on Cancer, Easier on the Patient

CLINICAL STUDY PROTOCOL

**Title:**	Phase 1/2, Open-Label, Single-Arm Safety, Tolerability, Anti-Tumor Effects, Systemic Exposure, and Device Technical Effects of PRV111 (Cisplatin Transmucosal System) in Subjects with Oral Squamous Cell Carcinoma
**Test Product:**	PRV111 (Cisplatin Transmucosal System) Drug Product
**Protocol Identification:**	CLN-001
**Sponsor Name, Address, and Telephone Number:**	Privo Technologies<br>200 Corporate Place, Suite 6B, Peabody, MA 01960<br>Phone : 978-587-2322<br>Fax : 978-278-5114
**Compliance Statement:**	The trial will be conducted in accordance with standards of Good Clinical Practice, as defined by the International Council for Harmonization and all applicable national and local regulations.
**Date of Original Protocol:**	3 August 2017
**Date of Amendment 1:**<br>**Date of Amendment 2:**<br>**Date of Amendment 3:**<br>**Date of Amendment 4:**<br>**Date of Amendment 5:**	17 October 2017<br>11 May 2018<br>26 March 2019<br>24 May 2019<br>23 March 2020
**President and CEO:**	Manijeh Goldberg, PhD, MBA<br>Signature Date: March 23, 2020
**Medical Monitor:**	[redacted]<br>[redacted]<br>Signature Date : March 23, 2020

Confidential Information

The information contained herein is confidential and may not be used, divulged, published, or otherwise disclosed without the prior written consent of Privo Technologies, Inc.

PROTOCOL SIGNATURE PAGE

Protocol : CLN-001

Title: Phase 1/2, Open-Label, Single-Arm Safety and Efficacy Dose-Finding, Systemic Exposure, and Device Technical Effects of PRV111 (Cisplatin Transmucosal System) in Subjects with Oral Squamous Cell Carcinoma

Amendment 5: 23 May 2020

Investigator Statement: I have received and completely reviewed the above-named protocol, including all appendices and amendments. As Investigator, I agree to conduct the trial in accordance with all stipulations of the protocol and in accordance with 21 Code of Federal Regulations (CFR) Part 50, International Council for Harmonization (ICH) Guidelines for Good Clinical Practices (GCP), and the Declaration of Helsinki.

INVESTIGATOR SIGNATURE AND CONTACT INFORMATION	
**Investigator (signature)**	
**Investigator (please print)**	
**Date of Signature**	
**Investigator's Address**	
**City, State, Country, Zip Code**	
**Telephone No.**	
**Fax No.**	
**Email**	

Signature on this page assures Privo Technologies that, to the best of the Investigator's knowledge, the affiliated Institutional Review Board operates in accordance with applicable local and national regulations, and that the Investigator understands and agrees to abide by all regulatory obligations and ICH GCP Guidelines while conducting this clinical trial.

Once signed, the original of this form should be detached from the protocol and returned to the Sponsor.
(Please retain a copy for your files)

STUDY CONTACT INFORMATION

Unless otherwise instructed, Serious Adverse Event and Serious and Unexpected Suspected Adverse Reaction reports are to be submitted to:

[redacted]

Who will forward a copy to:

Medical Monitor:
[redacted]

1 SYNOPSIS

Name of Sponsor/Company: Privo Technologies

Name of Finished Product: PRV111 (Cisplatin Transmucosal System)

Name of Active Ingredient: Cisplatin

Title of Study: Phase 1/2, Open-Label, Single-Arm, Safety and Efficacy Dose-Finding, Systemic Exposure, and Device Technical Effects of PRV111 (Cisplatin Transmucosal System) in Subjects with Oral Squamous Cell Carcinoma

Phase of Development: Phase 1/2

Objectives:
Primary Objectives

- Determine a safe and efficacious dose of PRV111 (Cisplatin Transmucosal System) (cisplatin drug-loaded patch + permeation enhancer [PE]) under the conditions of the trial.

Secondary Objectives

- Evaluate tumor response
- Assess the safety and tolerability of the treatment
- Evaluate systemic, tumor and lymph node (if available) platinum levels following administration
- Evaluate device technical success including the residual cisplatin level in the patch after the application period

Methodology: This is a Phase 1/2, open-label, prospective, single-arm study of the safety and efficacy dose-finding, systemic exposure, and device technical success of PRV111 (Cisplatin Transmucosal System) (cisplatin drug-loaded patch+PE) in subjects with oral squamous cell carcinoma (SCC) amenable to surgery.

Study Design: This is a 2-stage adaptive study. For Stage 1, up to 10 subjects will be enrolled for the purpose of 5 evaluable subjects for the 1st stage of the study to determine a safe and efficacious dose. For Stage 2, the target goal is 11 evaluable subjects for the efficacy analysis of the final dose. If the dose remains the same as Stage 1, Stage 2 will target 6 additional evaluable subjects but up to 21 subjects will be recruited if the dose is changed between Stage 1 and 2.

For the purpose of dose selection, tolerable dose is defined as a dose where fewer than 33% of subjects being evaluated within the safety population present with dose-limiting toxicities (DLTs). Effective dose is determined based on the evaluable population as described in the Statistical Methods section.

The 2 stages of the study are described below:

Stage 1: Up to 10 subjects will be enrolled for the purpose of 5 evaluable subjects and undergo 3 treatment applications at each of the 4 planned treatment visits. Each treatment visit will include 1 ml of the PE which will be applied pre and post patch application (see Pharmacy Manual for details). Starting approximately 5 minutes after application of the PE, 1 or 2 patches (depending on tumor size) will be applied. A total of 3 treatment applications of patches will occur. These successive applications will span approximately 35 minutes. The 3 applications provide a total of 6 mg of cisplatin per visit (12 mg if 2 patches are applied at the same time). The 4 treatment visits will be separated by 2 to 6 days (at least 48 hours apart).

The following decisions will be made based on safety and efficacy of the dose:

Dosing Rules for Stage 1:

1) If fewer than 2 subjects experience DLTs, then the following scheme will be considered for stage 2 dosing:
   a. If fewer than or equal to 2 of the evaluable subjects have a response (Tumor volume change from baseline (CFB) expressed as percent change from baseline (PCFB) complete response [CR], strong response [SR] or partial response [PR]) the dose will be escalated from 3 to 5 treatment applications per treatment visit for Stage 2.

**Name of Sponsor/Company:** Privo Technologies
**Name of Finished Product:** PRV111 (Cisplatin Transmucosal System)
**Name of Active Ingredient:** Cisplatin

b. If 3 or more evaluable subjects have a response (as tumor volume PCFB complete response [CR], strong response [SR] or partial response [PR]), the dose will remain the same for Stage 2 (3 applications per treatment visit).

2) If 2 or more subjects experience DLTs at any time during Stage 1, the following scheme will be considered for dose de-escalation in Stage 2:
   a. If there are 5 evaluable subjects in Stage 1:
      - If out of 5 evaluable subjects, 2 or more subjects have a response (as tumor volume PCFB complete response [CR], strong response [SR] or partial response [PR]), the dose will be de-escalated to 2 applications per treatment visit.
      - If out of 5 evaluable subjects, fewer than 2 have a response (as tumor volume PCFB complete response [CR], strong response [SR] or partial response [PR]), the dose will be considered ineffective and the study will be stopped.
   b. If Stage 1 dosing was stopped prior to having 5 evaluable subjects, the dose will be de-escalated from 3 applications to 2 applications per treatment visit.

Stage 1 Dose Determination Scheme:

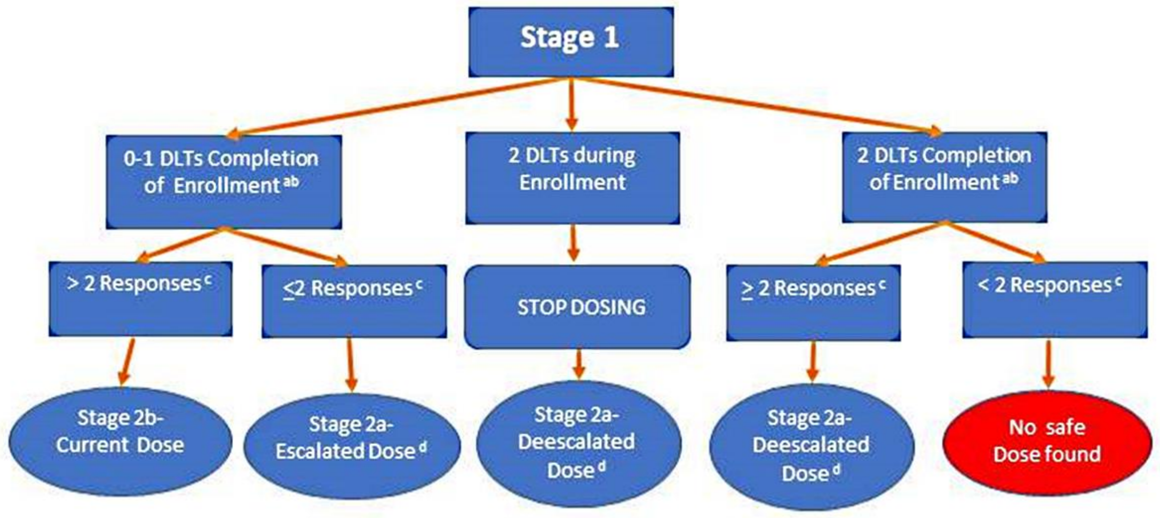

a) Stage 1 Enrollment Goal: 5 Evaluable Subjects. See section "Evaluable Subject"
b) 2 subjects with DLTs, evaluated based on Safety Subjects
c) Response based on evaluable subjects
d) Target Dose for Stage 2a

Stage 2: In case of dose escalation or de-escalation, Stage 2 will be conducted in 2 parts: Stage 2a and Stage 2b.

Subjects will receive 2, 3, or 5 treatment applications at each of the 4 planned treatment visits, depending on the results of Stage 1. Up to 21 additional subjects will be enrolled in Stage 2 if necessary, for the purpose of 11 total evaluable subjects at the final dose for the efficacy analysis.

Stage 2a: This stage will include 5 subjects (Stage 2a flowcharts). In this stage, only safety outcomes will be considered for determining the dosing regimen for the set of subjects to be enrolled in Stage 2b. All subjects enrolled in this stage will be monitored for possible DLTs.

Name of Sponsor/Company: Privo Technologies

Name of Finished Product: PRV111 (Cisplatin Transmucosal System)

Name of Active Ingredient: Cisplatin

1) If fewer than 2 subjects with DLTs in Stage 2a, enrollment will continue for the remaining number of subjects required in Stage 2b at the same dose.
2) If 2 subjects experience DLTs in Stage 2a, dosing will be re-considered. It may be stopped or, when possible, decreased for the remaining subjects enrolled in Stage 2b:
   a. If "Stage 2a - Escalated Dose", the original dose will be used for up to 10 additional evaluable subjects enrolled for Stage 2b.
   b. If "Stage 2a - De-escalated Dose", the study will stop, as no safe dose could be determined.

Stage 2a Safety Scheme in Case of Escalated Dose Decision:

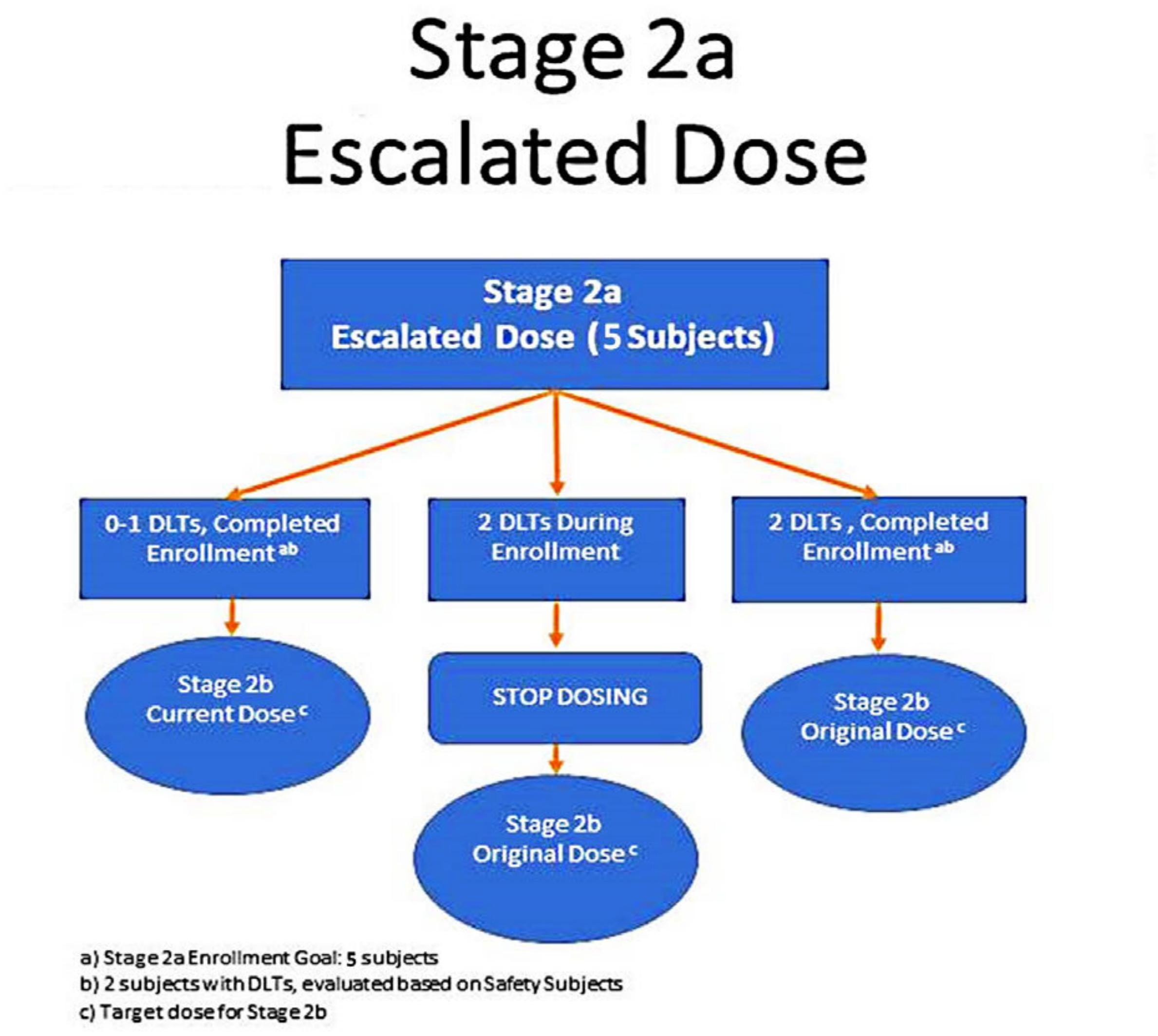

**Name of Sponsor/Company:** Privo Technologies
**Name of Finished Product:** PRV111 (Cisplatin Transmucosal System)
**Name of Active Ingredient:** Cisplatin

Stage 2a Safety Scheme in Case of De-escalated Dose Decision:

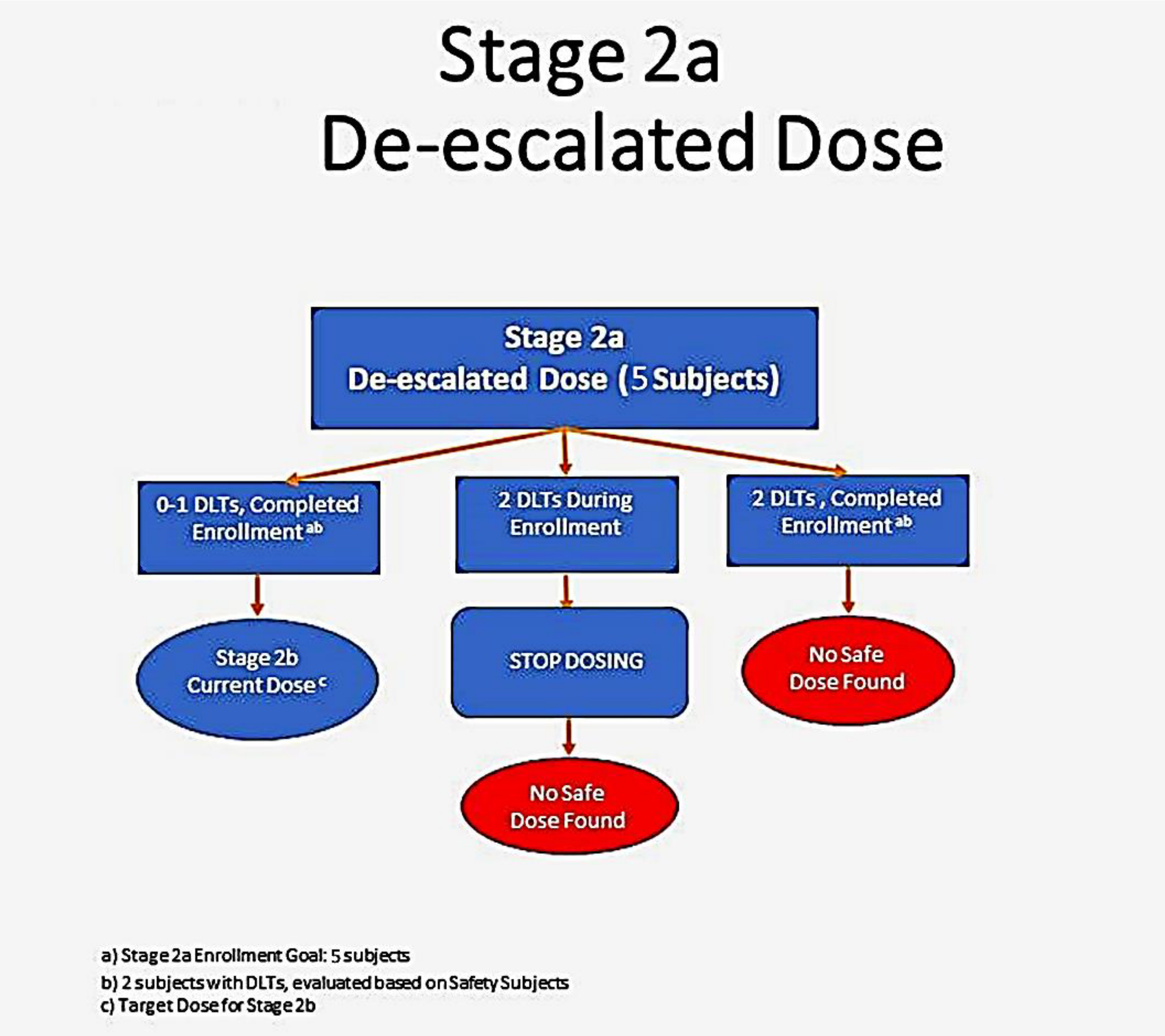

Stage 2b: In this stage both safety and efficacy will be evaluated. All subjects will be monitored for possible DLTs (Stage 2b – All Doses flowchart).

Stopping Rules at Stage 2b:

If 3 subjects with DLTs, no additional subjects will be dosed.

Efficacy Rules at Stage 2b:

Efficacy rules are described in the statistical plans for this stage since it is end of the study.

**Name of Sponsor/Company:** Privo Technologies	
**Name of Finished Product:** PRV111 (Cisplatin Transmucosal System)	
**Name of Active Ingredient:** Cisplatin	

Stage 2b Safety and Efficacy Scheme:

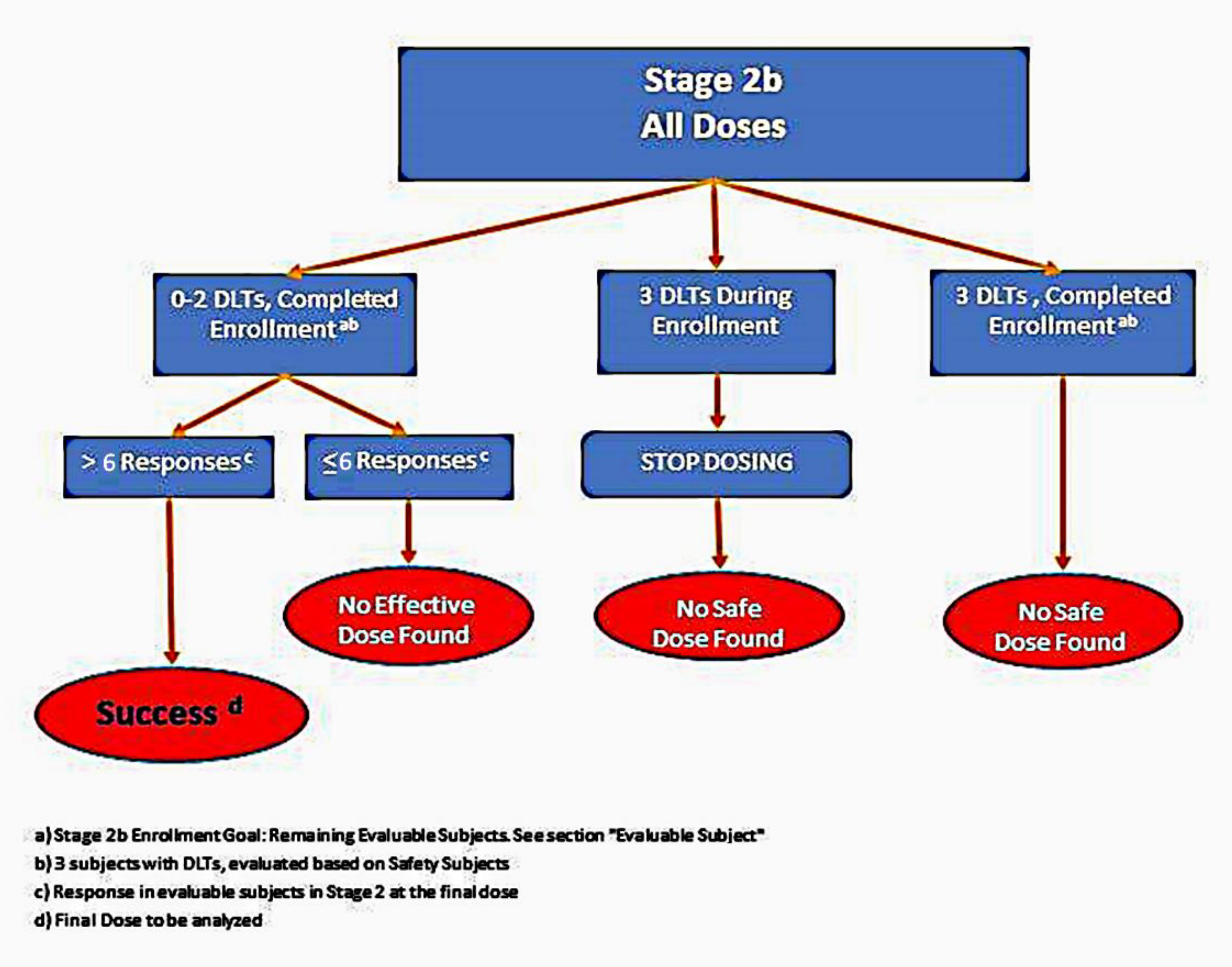

Note: The PRV111 treatment should not cause a delay to the scheduled surgery. Visit 1 can be performed within 42 days prior to the scheduled surgery.
All subjects will subsequently undergo standard of care, surgical ablation of the tumor and satellite nodes, and possibly additional radiotherapy and chemotherapy. All subjects will be followed for up to 6 months post-surgery at 3 visits: 1 month, 3 months, and 6 months or standard of care (discretion of the investigator) post-surgery for loco-regional response and safety.

Number of Subjects: Up to 31 subjects will be enrolled in the United States for the purpose of 11 evaluable subjects at the final dose for the efficacy analysis.

Diagnosis and Main Criteria for Inclusion:

In order to be eligible to participate in the study, an individual must meet all of the following criteria:

1. Adult subjects, men and women, defined by age ≥18 years at the time of screening.

**Name of Sponsor/Company:** Privo Technologies
**Name of Finished Product:** PRV111 (Cisplatin Transmucosal System)
**Name of Active Ingredient:** Cisplatin

2. Pathologically proven and clinically confirmed T1 (<2 cm) or T2 (>2 cm but ≤4 cm) SCC of the lip or oral cavity (anterior 2/3 of the tongue, floor of mouth, lower and upper gingiva, salivary gland, hard palate, and buccal mucosa).
3. Tumor must be easily accessible, with no evidence of infection or active bleeding, encroaching major vessels or clinical evidence of neural invasion. Tumors should not have been previously irradiated.
4. Tumor must be amenable to surgical resection no later than 42 days post Visit 1.
5. Clinically and/or radiologically measurable tumor.
6. Eastern Collaborative Oncology Group Performance Status of ≤2.
7. Adequate renal function as demonstrated by renal creatinine clearance ≥50 mL/min (calculated using the Cockcroft-Gault formula).
8. Adequate organ function defined as: ANC ≥1000/µL, platelet ≥100,000/µL; hemoglobin ≥9.0 g/dL; AST and ALT ≤2.5 times the upper limit of normal (ULN), serum bilirubin ≤1.5 times the ULN or direct bilirubin ≤ULN for subjects with total bilirubin >1.5 times the ULN.
9. Candidates for standard of care treatment consisting of surgery.
10. Male and female subjects of childbearing potential must agree to use 2 methods of effective contraception from screening and for at least 30 days after the final dose of investigational product. Appropriate birth control is defined as barrier methods with spermicides, oral or parenteral contraceptives and/or intrauterine devices, or naturally or surgically sterile (with documentation in the subject's medical records). Postmenopausal women are defined as presenting at least 12 months' natural spontaneous amenorrhea, or at least 6 weeks following surgical menopause (bilateral oophorectomy). Females of childbearing potential must be non-lactating and have a negative serum hCG within 14 days of treatment initiation.
11. Absence of any serious underlying medical conditions which could impair the ability of the subject to participate in the study.
12. Have a life expectancy of ≥3 months.
13. Willing and able to provide written informed consent.
14. Able to return to study site for treatment and follow-up visits as defined in the Protocol.

Criteria for Exclusion:

An individual who meets any of the following criteria will be excluded from participation in the study:

1. Known distant metastasis of the SCC of the oral cavity
2. Systemic chemotherapy for the treatment of SCC of the head and neck less than 2 years prior to Screening
3. Concurrent documented malignancy, with the exception of localized SCCs of the skin
4. Exposure to any investigational agent within 3 months prior to Screening
5. Known allergy or hypersensitivity to platinum-containing agents, or known intolerance to a prior platinum-containing agent, or to any of the excipients, which, in the judgement of the physician will preclude re-exposure to platinum-containing agent
6. Active, uncontrolled infection requiring systemic therapy, such as but not limited to HIV, Hepatitis B or C
7. Uncontrolled intercurrent illness that would risk subject safety, interfere with the objectives of the Protocol, or limit subject compliance with study requirements, as determined by the Investigator
8. Known or suspected pregnancy, planned pregnancy, or lactation
9. Any medical or psychiatric condition that may compromise the ability to give written informed consent

Test Product, Dose and Mode of Administration: PRV111 (Cisplatin Transmucosal System) consists of:

- PE [redacted] to be applied superficially to the malignant tumor to improve the drug permeation into the tumor (1 mL at each treatment visit) (Refer to the Pharmacy Manual).

**Name of Sponsor/Company:** Privo Technologies
**Name of Finished Product:** PRV111 (Cisplatin Transmucosal System)
**Name of Active Ingredient:** Cisplatin
• Patch with mucoadhesive polymeric matrix, embedded cisplatin-loaded chitosan particles (CLPs), and a non-permeable backing. Each 2 cm x 2 cm PRV111 topical patch covers a tumor region of 4 cm^2 and contains 0.5 mg/cm^2 (total 2 mg) of cisplatin.<br>patch(es). The number of patches used for each subject should remain consistent throughout treatment. Refer to the Pharmacy Manual for detailed instructions for use.
**Duration of Treatment:** Up to 6 weeks of treatment prior to the scheduled resection of the tumor and 6 months post-surgical follow-up. ***Note:*** The PRV111 treatment should not cause a delay to the scheduled surgery. Visit 1 can be performed within 42 days prior to the scheduled surgery.
**Criteria for Evaluation:**<br>***Safety/Tolerability evaluation based on:***<br>• Adverse events<br>• Physical examination, vital signs, and weight<br>• Blood and urinary tests: complete blood count, chemistry panel, and urinalysis<br>• Electrocardiogram<br>• Oral examination: including observation for mucositis and other signs of local reaction to the test article<br>• Neck lymph nodes examination<br><br>***Efficacy, based on:***<br>• Tumor volume change from baseline clinical assessment (CR, PR, SR, SD, or PD). Response defined as CR, SR, or PR; non-response defined as SD or PD.<br>• Ruler or caliper measurements of tumor volume<br>• Computed tomography or magnetic resonance imaging of the head and neck<br>• Histology at the time of surgical excision<br>• Tumor photography and measurements<br>• Loco-regional control: subjects will be followed-up for disease recurrence and safety following surgery<br><br>***Pharmacokinetics:***<br>• Whole blood samples will be assessed for total platinum content<br><br>***Technical Success will be defined as:***<br>• Taste of the PE solution<br>• Taste of the PRV111 patch<br>• Adhesion, including the time of adhesion of PRV111 patch<br>• PRV111 patch peel-off<br>• Residual cisplatin level in the patch after the application period. All PRV111 patches will be assessed for the amount of residual cisplatin in each patch after each application period. If the product detaches prematurely, the residual cisplatin will be assessed and the time of detachment will be noted.
**Statistical Methods:**<br>Safety outcomes resulting in a DLT will be used during the conduct of this study to select doses being tested according to pre-defined thresholds of toxicity.<br>• Local and systemic safety and tolerability will be summarized using descriptive statistics.<br>The number of subjects with DLTs will be capped to be fewer than 2 subjects with DLTs out of 5 new subjects enrolled.<br>The treatment regimen will be deemed tolerable if fewer than a third of the subjects exposed to it present DLTs.

**Name of Sponsor/Company:** Privo Technologies
**Name of Finished Product:** PRV111 (Cisplatin Transmucosal System)
**Name of Active Ingredient:** Cisplatin

- Efficacy based on tumor volume CFB criteria will be summarized, and the null hypothesis that the probability of response is at most 30%, where a response is the occurrence of CR, SR or PR will be tested using a 1-sided test exact binomial test at level 0.018.
- Other efficacy assessments such as change in tumor volume or tumor histology will be summarized using descriptive statistics.
- Systemic and tumor and lymph nodes (if available) platinum levels will be summarized using descriptive statistics.
- Variables measuring technical success (including residual cisplatin level in the patch) will be summarized using descriptive statistics.

All summaries will be presented by dose, as appropriate. Incidence of pre-surgery AEs will be summarized using number and percent for each System Organ Class and Preferred Term (MedDRA), including Investigator assessment of relatedness and severity grade.

All assessments will be listed and summarized using mean, standard deviation, median, minimum, and maximum for continuous variables and using number and percent for categorical assessments.

Data from this study will be summarized at 2 timepoints:

- Final report at End of Stage 2: after all subjects complete PRV111treatment, efficacy, pharmacokinetics, and technical data will be analyzed, including all available safety data from the long-term follow-up.
- Long term safety report: after all subjects complete all observations.

Power and Sample Size

The planned enrollment for the final dose is intended to obtain N=11 evaluable subjects.

The planned enrollment for the final dose is intended to recruit N=11 evaluable subjects.

In the first stage, 5 evaluable subjects will be accrued. If there are 2 or fewer responses in these 5 subjects, the dose will be escalated. Otherwise, the dose will remain the same or de-escalated based on safety data. Additional subjects will be accrued for a total of up to 11 evaluable subjects at the final dose.

If the dose is not increased after Stage 1, the test will be performed using all evaluable subjects from Stage 1 and 2. If the dose is adjusted after Stage 1, the test will be performed using all evaluable subjects from Stage 2.

Based on Simon's 2-stage procedure, the null hypothesis that the true response rate is 0.30 will be tested against a one-sided alternative. The null hypothesis will be rejected if 7 or more responses are observed at the final dose. This design yields a type I error rate of 0.018 and power of 96.44% when the true response rate is 0.85.

Null response rate: P_0	Alt. response rate: P_1	Alpha one-tailed	Power	n1	r1	N	R
0.30	0.85	0.018	96.44%	5	2	11	6

N *is the total number of evaluable subjects at the final dose*
n*1 is the number of evaluable subjects accrued during Stage 1*
r*1, if* r1 *or fewer responses are observed during stage 1, escalate the dose if safety requirements are met*
R, *if* R *or fewer responses are observed by the end of Stage 2 in N subjects, then an effective dose is not found*

Pharmacokinetics

Pharmacokinetics parameters will include area under the curve, maximum concentration, minimum concentration, time to maximum concentration, and half-life

Table 1. Schedule of Events

	Screening Within 28 Days Prior to Visit 1	Days					Surgery	Months Post Surgery
		Treatment Visits				Post Treatment Pre-Op		Standard of Care (SOC) Follow-up
		Visit 1	Visit 2	Visit 3	Visit 4	Visit 5	Visit 6	Visits 7, 8, 9
	0	1	≥48 and ≤ 144 hours, post previous visit			≥72 hours post last treatment	≤42 days post Visit 1	1, 3, & 6 months (all can be +/-3 weeks) SOC visits
Confirmed SCC	X							
Informed consent	X							
Inclusion/exclusion criteria	X	X[a]						
Schedule study visits	X	X	X	X	X	X		
Demographic, medical/history	X							
Physical examination,[b,s] VS[c]	X	X[a]	X[a]	X[a]	X[a]	X		X
Anti-emetics		X[a]	X[a]	X[a]	X[a]			
CT chest [s]	X							
Oral cavity examination[d]	X	X[a]	X[a]	X[a]	X[a]	X		X
Neck lymph nodes exam	X	X[a]	X[a]	X[a]	X[a]	X		X
ECG [s]	X					X		
CT or MRI head and neck[e, s]	X					X		
Clinical labs[f]	X	X[a]		X[a]		X	X[o]	
HPV [s]	X[g]							
Pregnancy test	X[h]	X[a]			X			
PK sampling		X[i]	X[j]	X[j]	X[i]			
Tumor resection							X[k]	
PRV111 patch treatment		X[p]	X[p]	X[p]	X[p]			
Tumor measurements[q]		X[a]	X[a]	X[a]	X[a]	X		

	Screening Within 28 Days Prior to Visit 1	Days					Surgery	Months Post Surgery
		Treatment Visits				Post Treatment Pre-Op		Standard of Care (SOC) Follow-up
		Visit 1	Visit 2	Visit 3	Visit 4	Visit 5	Visit 6	Visits 7, 8, 9
	0	1	≥48 and ≤ 144 hours, post previous visit			≥72 hours post last treatment	≤42 days post Visit 1	1, 3, & 6 months (all can be +/-3 weeks) SOC visits
Tumor Volume change from baseline (CFB) criteria			X[a]	X[a]	X[a]	X		
Tumor photographs		X[l]	X[l]	X[l]	X[l]	X		
Loco-regional recurrence								X
Histology[s]	X[m]						X[m]	
Technical success[n]		X	X	X	X			
Concomitant therapy	X	X	X	X	X	X		X
AE assessment	X	X	X	X	X	X[r]		

Abbreviations: AE=adverse event; CT=computed tomography; ECG=electrocardiogram; HPV=human papilloma virus; MRI=magnetic resonance imaging; PE=permeation enhancer; PK=pharmacokinetics; SCC=squamous cell carcinoma; VS=vital signs.

a Performed prior to dosing.
b If a physical exam was performed within 14 days prior to the screening visit, it does not need to be repeated at the screening.
c VS: seated blood pressure and pulse will be measured after the subject has been sitting for at least 3 minutes, respiration, temperature, height and weight will be recorded at every visit. VS will be obtained pre-dose at all visits and subject will also be monitored at 30+5 minutes, 60+5 minutes and 120+5 minutes following application of the last patch applied at Visits 1 and 4 and at 30+5 minutes post last patch application at Visits 2 and 3.
d Take photos of the tumor region for further assessment of tumor shrinkage.
e Every attempt should be made to use the same test for each subject for both timepoints.
f CBC and chemistry panel (creatinine to determine the creatinine clearance, BUN, LFT, electrolytes (potassium, magnesium, calcium, and sodium), urinalysis.
g Tissue will be collected at Screening but the HPV status will be ascertained at any time during the study using immunohistochemistry for p16 according to institutional clinical protocols.
h Serum pregnancy test to establish eligibility for female subjects of childbearing potential. A negative urine pregnancy test must be documented within 7 days prior to the first treatment visit, and at Visit 5.
i Visit 1 & 4: PK blood samples for total platinum collected pre- treatment (time -15 to 0 minutes), and 30±5 minutes, 60±5 minutes and 120±5 minutes post removal of last patch.
j Visit 2 & 3: PK blood samples for total platinum collected pre- treatment (time -15 to 0 minutes), and 30±5 minutes, post removal of last patch and PE application.
k Sampling for tumor and lymph nodes (if available) platinum levels.
l Photos need to be taken prior to patch application.
m Confirmation of tumor histology by a clinical pathologist is required prior to enrollment. A final pathology report, issued by a clinical pathologist is required following surgical resection of the tumor specimen.
n Taste of the PE solution and patch, adhesion, peel-off, residual cisplatin level in the patch.
o Performed after surgery if possible

p PE should be applied to the tumor area prior to first patch and allow to stay for 5 minutes. Then applied again to the tumor area for 1 minute. Note that the mouth should be kept open for the saliva not wash off the PE.

q Use “0.05” cm for the “Height” of a flat tumor. If a concave tumor, the “Height” can be shown as a negative number. If a non-measurable dent, use “-0.05” cm.

r Only SAEs within 30 days post visit 5 need to be recorded.

s. May use historical SOC information prior to consent if collected within 28 days prior to first treatment.

2 TABLE OF CONTENTS

TITLE PAGE 1
PROTOCOL SIGNATURE PAGE 2
STUDY CONTACT INFORMATION 3
1 SYNOPSIS 4
2 TABLE OF CONTENTS 15
2.1 List of Tables 18
2.2 List of Figures 19
3 LIST OF ABBREVIATIONS AND TERMS 20
4 INTRODUCTION 22
4.1 Background of Disease 22
4.2 Privo's PRV111 Patch 23
4.3 Rationale for Use of the Product 23
4.4 Rationale for Dose Selection 24
5 TRIAL OBJECTIVES 24
5.1 Primary Objective 24
5.2 Secondary Objectives 24
6 INVESTIGATIONAL PLAN 25
6.1 Overall Trial Design and Plan: Description 25
6.2 Discussion of Trial Design 29
7 SELECTION OF SUBJECTS 30
7.1 Inclusion Criteria 30
7.2 Exclusion Criteria 30
7.3 Criteria for Individual Subject Discontinuation of Protocol Therapy 31
7.4 Criteria for Individual Subject Discontinuation from the Trial 31
7.5 Handling of Subject Withdrawals or Subject Discontinuation of Study Intervention . 31
7.6 Premature Termination or Suspension of Study 32
7.7 Replacement of Subjects 32
8 TREATMENTS 32
8.1 Treatments Administered 32
8.1.1 Description of Test Product 32
8.1.2 Packaging 33
8.1.3 Labeling 33

8.1.4 Preparation ... 33
8.1.5 Dosing and Administration ... 33
8.2 Dose-limiting Toxicity (Stopping Rules for an Individual Subject) ... 33
8.3 Dose Modification ... 34
8.4 Dose Interruption ... 34
8.5 Study Drug Handling and Accountability ... 34
8.6 Study Drug Disposal ... 35
8.7 Concomitant Medications and Therapy ... 35
8.8 Drugs Contraindicated with PRV111 ... 36
9 VARIABLES ASSESSED ... 36
9.1 Eligibility Only Assessments ... 36
9.1.1 Confirmation of Squamous Cell Carcinoma ... 36
9.1.2 Tumor Staging ... 36
9.1.3 Demographics, Medical and Medication History ... 36
9.1.4 Pregnancy Test ... 37
9.1.5 Eastern Collaborative Oncology Group ... 37
9.2 Efficacy Assessments ... 37
9.2.1 Tumor Photography and Measurements ... 37
9.2.2 Response and Non-Response (Tumor Volume Change from Baseline Criteria) ... 38
9.2.3 Computed Tomography or Magnetic Resonance Imaging of the Head and Neck ... 38
9.2.4 Histopathology Response to Treatment ... 38
9.2.5 Loco-regional Control ... 39
9.3 Safety Assessments ... 39
9.3.1 Physical Examination, Vital Signs, Weight ... 39
9.3.2 Oral Cavity Examination ... 39
9.3.3 Neck Lymph Nodes Examination ... 39
9.3.4 Safety Laboratory Assessments ... 39
9.3.5 Electrocardiograms ... 40
9.3.6 Adverse Event Definitions/Reporting Requirements ... 40
9.3.6.1 Expected Events ... 40
9.3.6.2 Suspected Adverse Reaction ... 40
9.3.6.3 Adverse Reaction ... 41
9.3.6.4 Adverse Device Effects ... 41
9.3.6.5 Serious Adverse Event Definitions / Reporting Requirements ... 41

9.3.6.5.1 Serious Adverse Event or Serious Suspected Adverse Reaction 41
9.3.6.5.2 Pregnancy 42
9.3.6.5.3 Serious Unexpected and Suspected Adverse Reaction 42
9.3.6.6 Recording Adverse Events 42
9.3.6.7 Determining Relationship of Adverse Events to Study Drug 43
9.3.6.8 Reporting Deaths and Other Serious Adverse Events 43
9.3.6.8.1 Events to Be Reported and Timeframe 43
9.3.6.8.2 Governing Regulatory Requirements 44
9.3.6.9 Initial Information Provided by the Investigator 44
9.3.6.10 Follow-up Information 45
9.3.6.11 Serious Adverse Event that are Unexpected and related Serious Adverse Reaction Review that have a Potential Impact on Trial Conduct 45
9.4 Pharmacokinetics Assessment 46
9.5 Technical Success Assessments 46
9.6 Timing of Assessments 46
9.6.1 Screening (Visit 0) 46
9.6.2 Baseline, Treatment (Visit 1) 47
9.6.3 Treatment (Visit 2) 48
9.6.4 Treatment (Visit 3) 48
9.6.5 Treatment (Visit 4) 49
9.6.6 Post-Treatment/Pre-Op Visit (Visit 5) 50
9.6.7 Surgery Day (Visit 6) 50
9.6.8 Post-Treatment Follow-Up (Visits 7, 8, & 9) 50
10 STATISTICAL METHODS AND PLANNED ANALYSES 51
10.1 General Considerations 51
10.2 Sample Size Determination 51
10.3 Pharmacokinetics 52
10.4 Study Populations 52
10.4.1 Safety Population 52
10.4.2 Evaluable Population 52
10.5 Statistical Analyses 52
10.5.1 Analysis Timepoints 52
10.5.2 Subject Accountability, Demographics, and Baseline Characteristics 52
10.5.3 Primary Endpoints 52

10.5.4 Secondary Endpoints 52
10.5.5 Efficacy Data 53
10.5.6 Safety Data 53
10.5.7 Pharmacokinetics Data 54
10.5.8 Technical Data 54
11 QUALITY CONTROL AND QUALITY ASSURANCE 54
12 DOCUMENTATION AND INSPECTIONS 54
12.1 Study Monitoring 54
12.2 Audits and Inspections 55
13 ETHICAL AND LEGAL ISSUES 55
13.1 Ethical Conduct/Good Clinical Practice 55
13.2 Institutional Review Board 55
13.3 Written Informed Consent 56
13.3.1 Update of Informed Consent 56
13.4 Records and Case Report Forms 56
13.5 Protocol Deviations 57
13.6 Retention of Records 57
14 REFERENCES 57
15 APPENDIX 1: AMENDMENT 1 59
16 APPENDIX 2: AMENDMENT 2 79
17 APPENDIX 3: AMENDMENT 3 110
18 APPENDIX 4: AMENDMENT 4 123
19 APPENDIX 5: AMENDMENT 5 130

2.1 List of Tables

Table 1. Schedule of Events 12
Table 2. Dosing Terminology 33
Table 3. Eastern Collaborative Oncology Group Performance Status 37
Table 4. Clinical Response to Treatment Based on Tumor Volume Change* 38
Table 5. Pathological Response to Treatment 38
Table 6. Clinical Laboratories 39
Table 7. Clinical Response to Treatment 113

Table 8. Clinical Response to Treatment Based on Tumor Volume Change* 114

2.2 List of Figures

Figure 1. PRV111 (Cisplatin Transmucosal System) 23
Figure 2. Stage 1 dose determination scheme 26
Figure 3. Stage 2a safety scheme in case of escalated dose decision 27
Figure 4. Stage 2a safety scheme in case of de-escalated dose decision 28
Figure 5. Stage 2b safety and efficacy scheme 29

3 LIST OF ABBREVIATIONS AND TERMS

**Abbreviation**	**Definition**
AE	Adverse Event/Adverse Experience
AUC	Area under the curve
BUN	Blood urea nitrogen
CBC	Complete Blood Count
CFB	Change from Baseline
CFR	Code of Federal Regulations
CLP	Cisplatin-Loaded Chitosan Particles
C_{max}	Maximum concentration
C_{min}	Minimum concentration
CR	Complete response
CRF	Case Report Form
CT	Computed tomography
CTCAE	Common Terminology Criteria for Adverse Events
DLT	Dose-limiting toxicity
ECG	Electrocardiogram
ECOG PS	Eastern Collaborative Oncology Group Performance Status
FDA	Food and Drug Administration
GCP	Good Clinical Practice
HPV	Human papillomavirus
IB	Investigator’s Brochure
ICF	Informed Consent Form
ICH	International Council on Harmonisation
IRB	Institutional Review Board
IV	Intravenous
LFT	Liver function test
MRI	Magnetic resonance imaging
NCI	National Cancer Institute
PCFB	Percent change from baseline
PD	Progressive disease
PE	Permeation enhancer
PI	Principal Investigator

**Abbreviation**	**Definition**
PK	Pharmacokinetics
PR	Partial response
PRMC	Protocol Review and Monitoring Committee
PRV111	Cisplatin Transmucosal System
QA	Quality Assurance
RECIST	Response Evaluation Criteria in Solid Tumors
SAE	Serious Adverse Event
SCC	Squamous cell carcinoma
SD	Stable disease
SUSAR	Serious and Unexpected Suspected Adverse Reaction
$t_{1/2}$	Half life
TEAE	Treatment emergent adverse event
T_{max}	Time to maximum concentration
ULN	Upper limit of normal
US	United States
VS	Vital signs

4 INTRODUCTION

4.1 Background of Disease

Squamous cell carcinomas (SCC) of the head and neck include tumors that occur in the oral cavity (lips, buccal mucosa, anterior tongue, floor of the mouth, hard palate, upper gingiva, lower gingiva, buccal mucosa and retromolar trigone) (Brockstein, 2016). Most lip and oral cavity cancers originate in squamous cells, the thin flat cells that line the lips and oral cavity. Risk factors for oral cancer include chronic heavy use of alcohol and tobacco, long periods of exposure to natural or artificial sunlight, and exposure to human papillomavirus (HPV). Statistics also show that males and those of low socioeconomic background are at a higher risk of developing oral cancer than other demographics (Brockstein, 2016; da Silva, 2012).

In 2017, an estimated 50,000 new cases of oral and pharynx cancer will be reported in the United States (US); approximately 11.1 adults per 100,000 will develop oral and pharynx cancer per year (National Cancer Institute, 2016). The median age of diagnosis is 62 years. The incidence of oral cancer increases with age (National Cancer Institute, 2016; National Institute of Dental and Craniofacial Research, 2016).

The 5-year survival rate for oral cancer is approximately 60%; diagnosis at an early stage significantly increases the 5-year survival rate (83% for Stage I versus 10%–22% for Stage IV) (da Silva, 2012; National Cancer Institute, 2016; National Institute of Dental and Craniofacial Research, 2014). Approximately 30% of subjects have early (Stage I or II) localized disease, with most subjects diagnosed with advanced disease (Brockstein, 2016; da Silva, 2012; National Cancer Institute, 2016).

Oral cancer has a high rate of recurrence (18%–76% for subjects who undergo standard treatment), and is considered the major cause of poor survival rates. Recurrence can vary by tumor location, with a median time to recurrence of 9.5 months (range 2–79 months) for tongue, 8 months (range 2–120 months) for buccal tumor, and 5–8 months for lip tumors (da Silva, 2012; Gurudutt, 2011; Kelner, 2014). The median time to recurrence across all oral cancer sites is 7.5 months, with most recurrences (86%) occurring within 24 months (da Silva, 2012).

Approximately 1/3 of subjects treated with surgery and adjuvant therapy will experience local or regional recurrence and/or distant metastasis. Local and regional recurrences account for up to 90% of treatment failures post-surgery and radiotherapy (da Silva, 2012). An estimated 9,700 deaths will occur due to oral and pharynx cancer in the US in 2017 (National Cancer Institute, 2016).

The treatment and management of oral cancer is dependent upon the stage of disease. Standard treatment for localized early disease is surgery with or without radiation (Brockstein, 2016). For subjects with pathologically loco-regionally advanced disease (Stage III/IV), or for those treated with surgery for early stage disease identified to have close or positive margins, or positive lymph nodes, adjuvant postoperative radiotherapy with or without chemotherapy is recommended (Brockstein, 2016). Typical treatment approaches for loco-regionally advanced disease will include neoadjuvant chemotherapy or radiotherapy, primary surgery followed by

adjuvant radiotherapy or chemoradiotherapy, or chemotherapy or chemoradiotherapy without surgery (Brockstein, 2016; da Silva, 2012; Gregoire, 2010; Marta, 2015; Seiwert, 2007). Salvage surgery, adjuvant radiation, re-irradiation, and/or palliative chemotherapy is recommended for subjects with locally recurrent disease (Milano, 2015). For subjects with locally recurrent metastatic disease, palliative chemotherapy, immunotherapy, and/or supportive care are recommended (Brockstein, 2016). The most common chemotherapy drugs for advanced oral cancer includes cisplatin, methotrexate, carboplatin, 5-fluorouracil, paclitaxel, and docetaxel, which when administered alone or in combination have been shown to provide clinical benefit with demonstrated improvement in overall survival (da Silva, 2012; Gregoire, 2010; Vermorken, 2010).

4.2 Privo's PRV111 Patch

PRV111 (Cisplatin Transmucosal System) is a thin, 2-layer, matrix-type, non-sterile transmucosal patch consisting of a chitosan matrix layer embedded with cisplatin loaded chitosan particles (CLPs) and a non-woven fabric adhesive unidirectional backing, which is applied to the matrix layer during manufacturing (Figure 1). The patch is self-adhesive.

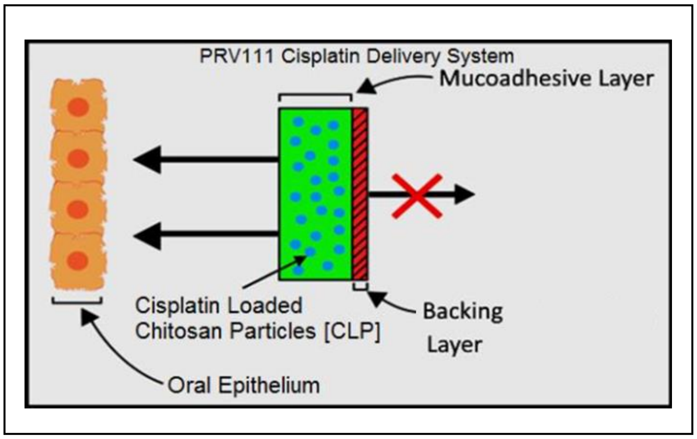

Figure 1. PRV111 (Cisplatin Transmucosal System)

In addition to the PRV111 drug product, a separately packaged Permeation Enhancer (PE) [redacted] is used in conjunction with the PRV111 drug product. The reconstituted PE Solution is intended to improve the absorption of the cisplatin active ingredient and will be applied pre and post patch application (see Pharmacy Manual for details).

4.3 Rationale for Use of the Product

Privo's PRV111 is intended as a local induction chemotherapy, to improve the local control of the disease prior to surgery by shrinking the tumor size. Local cisplatin delivery may result in larger and more effective doses of cisplatin delivered to the tumor while significantly reducing or eliminating the systemic toxicities and side effects traditionally associated with intravenous (IV)

cisplatin chemotherapy. If proven safe and effective for the treatment of SCC of the oral cavity, PRV111, in association with the standard care, may decrease the risk of local tumor recurrence.

Cisplatin (cis-diamminedichloroplatinum) has been approved in the US for 38 years as an antineoplastic agent and is commonly administered systemically IV as a standard of care chemotherapy for the treatment of oral cancer, alone and in combination with other chemotherapies (da Silva, 2012; Vermorken, 2007; Vermorken, 2010). However, systemic treatment with cisplatin can lead to dose-limiting toxicities (DLTs) and significant side effects, including renal toxicity, ototoxicity, and myelosuppression, which can limit the extent and duration of treatment (WG Critical Care, 2015). These side effects of IV cisplatin administration can be dose-limiting, reducing the dosage and length of cisplatin treatment for subjects. Treatment with cisplatin which is targeted to the tumor site and not administered systemically may reduce the dose-limiting systemic toxicities that are observed with IV cisplatin administration.

The antineoplastic activity of cisplatin can be primarily attributed to its ability to bind and modify nuclear and mitochondrial DNA, forming multiple intrastrand and interstrand DNA adducts, which leads to the activation of the p53 apoptotic pathway and subsequently cell death (Galluzzi, 2014; Johnstone, 2015). Cisplatin has also been linked to inducing chemokine and T-cell infiltration of tumor sites, thereby impeding tumor growth (Hong, 2011).

4.4 Rationale for Dose Selection

The maximum total planned PRV111 cisplatin dose if dose escalation proceeds as planned in Stage 2 (10 mg per treatment visit for 5 single patch applications, and 20 mg per treatment visit for 5 double patch applications) is approximately 9 to 18 times lower than the recommended dose of IV cisplatin (180 mg for an 80 kg person) (WG Critical Care, 2015).

Version 10 of the drug product will be used for this clinical trial since it has shown good safety and efficacy in 4 nonclinical studies. This version has the optimal dose per patch as 2 mg (0.5 mg/cm^2) that balances the quality, drug release and uniformity of the patch.

5 TRIAL OBJECTIVES

5.1 Primary Objective

- Determine a safe and efficacious dose of PRV111 (Cisplatin Transmucosal System) (cisplatin drug-loaded patch + PE) under the conditions of the trial.

5.2 Secondary Objectives

- Evaluate tumor response
- Assess the safety and tolerability of the treatment
- Evaluate systemic, tumor , and lymph nodes (if available) platinum levels following administration
- Evaluate device technical success including the residual cisplatin level in the patch after the application period

6 INVESTIGATIONAL PLAN

6.1 Overall Trial Design and Plan: Description

Study Design: This is a 2-stage adaptive study. For Stage 1, up to 10 subjects will be enrolled for the purpose of 5 evaluable subjects for the 1st stage of the study to determine a safe and efficacious dose. For Stage 2, the target goal is 11 evaluable subjects for the efficacy analysis of the final dose. If the dose remains the same as Stage 1, Stage 2 will target 6 additional evaluable subjects but up to 21 subjects will be recruited if the dose is changed between Stage 1 and 2.

For the purpose of dose selection, tolerable dose is defined as a dose where fewer than 33% of subjects being evaluated within the safety population present with DLTs. Effective dose is determined based on the evaluable population as described in the Section 10.2.

The 2 stages of the study are described below:

Stage 1: Up to 10 subjects will be enrolled for the purpose of 5 evaluable subjects and undergo 3 treatment applications at each of the 4 planned treatment visits. Each treatment visit will involve application of 1 ml of the permeation enhancer and will be applied pre and post patch application (see Pharmacy Manual for details) (One ml vial of PE per visit, see Pharmacy Manual). Starting approximately 5 minutes after application of the PE, 1 or 2 patches (depending on tumor size) will be applied. A total of 3 treatment applications of patches will occur. These successive applications will span approximately 35 minutes. The 3 applications provide a total of 6 mg of cisplatin per visit (12 mg if 2 patches are applied at the same time). The 4 treatment visits will be separated by 2 to 6 days (at least 48 hours apart).

The following decisions will be made based on safety and efficacy of the dose:

Dosing Rules for Stage 1:

1) If fewer than 2 subjects experience DLTs, then the following scheme will be considered for Stage 2 dosing:
   a. If fewer than or equal to 2 of the evaluable subjects have a response (as tumor volume PCFB complete response [CR], strong response [SR] or partial response [PR])) the dose will be escalated from 3 to 5 treatment applications per treatment visit for Stage 2.
   b. If 3 or more evaluable subjects have a response (as tumor volume PCFB complete response [CR], strong response [SR] or partial response [PR])), the dose will remain the same for Stage 2 (3 applications per treatment visit).
2) If 2 or more subjects experience DLTs at any time during Stage 1, the following scheme will be considered for dose de-escalation in Stage 2:
   a. If there are 5 evaluable subjects in Stage 1:
      - If out of 5 evaluable subjects, 2 or more subjects have a response (as tumor volume PCFB complete response [CR], strong response [SR] or partial response [PR])), the dose will be de-escalated to 2 applications per treatment visit.

- If out of 5 evaluable subjects, fewer than 2 have a response (as tumor volume PCFB complete response [CR], strong response [SR] or partial response [PR])), the dose will be considered intolerable and the study will be stopped.

b. If Stage 1 dosing was stopped prior to having 5 evaluable subjects, the dose will be de-escalated from 3 applications to 2 applications per treatment visit.

c.

Figure 2. Stage 1 dose determination scheme

Stage 2: In case of dose escalation or de-escalation, Stage 2 will be conducted in 2 parts: Stage 2a and Stage 2b.

Subjects will receive 2, 3, or 5 treatment applications at each of the 4 planned treatment visits, depending on the results of Stage 1. Up to 21 additional subjects will be enrolled in Stage 2 if necessary, for the purpose of 11 total evaluable subjects at the final dose for the efficacy analysis.

Stage 2a:

This stage will include 5 subjects (Stage 2a flowcharts). In this stage, only safety outcomes will be considered for determining the dosing regimen for the set of subjects to be enrolled in Stage 2b. All subjects enrolled in this stage will be monitored for possible DLTs.

1) If fewer than 2 subjects with DLTs in Stage 2a, enrollment will continue for the remaining number of subjects required in Stage 2b at the same dose.

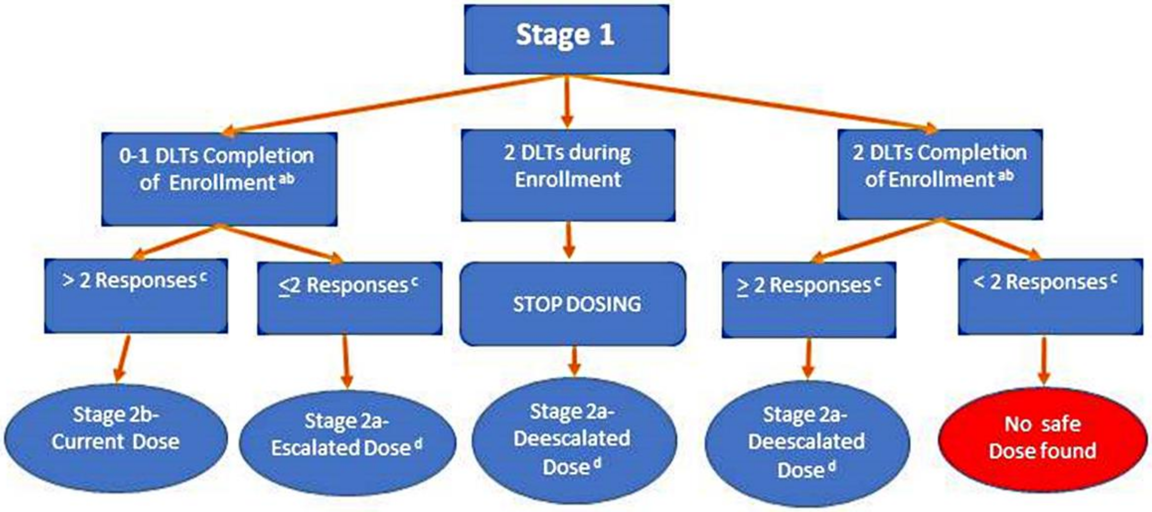

a) Stage 1 Enrollment Goal: 5 Evaluable Subjects. See section "Evaluable Subject"
b) 2 subjects with DLTs, evaluated based on Safety Subjects
c) Response based on evaluable subjects
d) Target Dose for Stage 2a

2) If 2 subjects experience DLTs in Stage 2a, dosing will be re-considered. It may be stopped or, when possible, decreased for the remaining subjects enrolled in Stage 2b:
   a. If “Stage 2a - Escalated Dose”, the original dose will be used on the remaining patients.
   b. If “Stage 2a - De-escalated Dose”, the study will stop, as no safe dose could be determined.

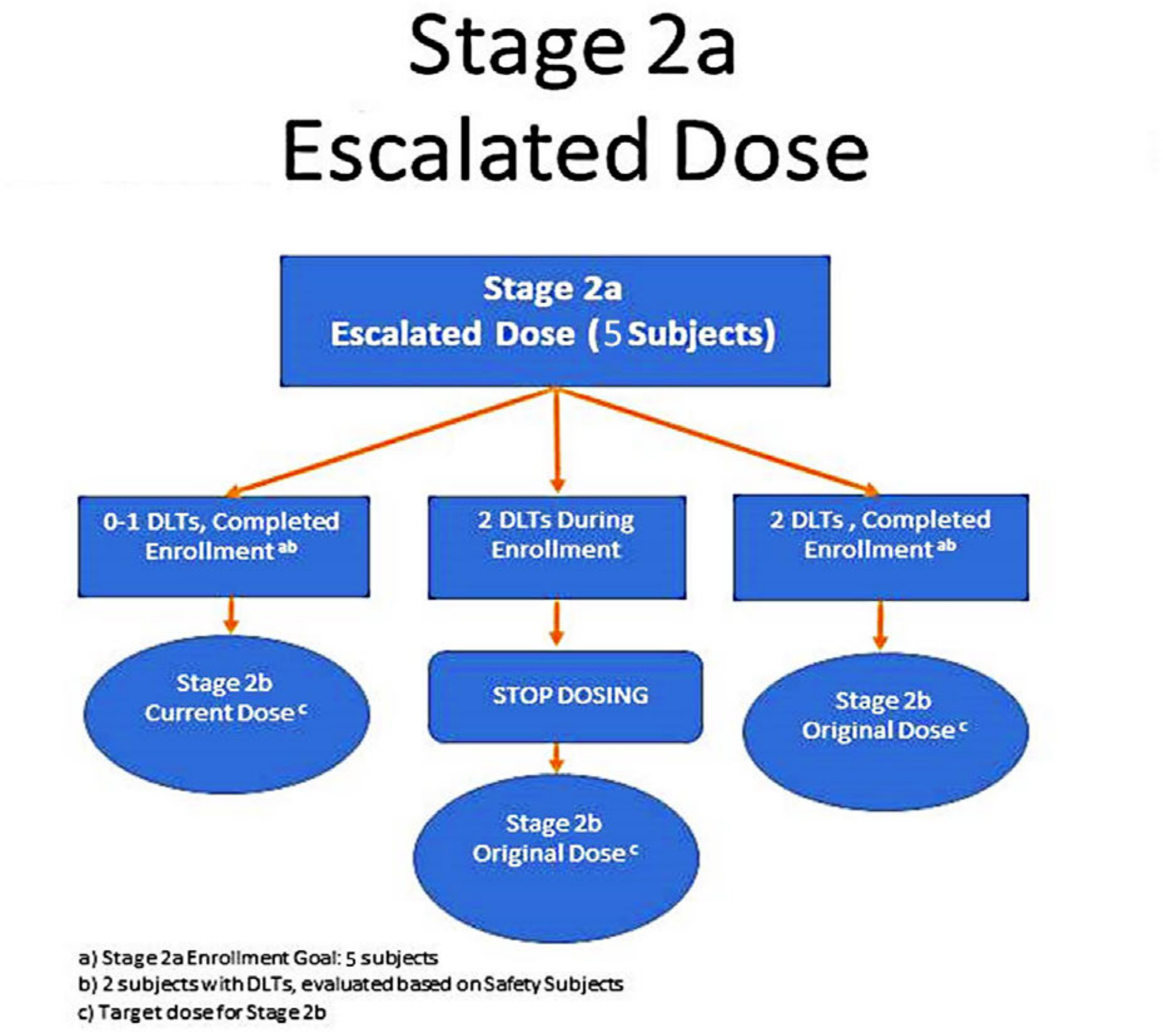

Figure 3. Stage 2a safety scheme in case of escalated dose decision

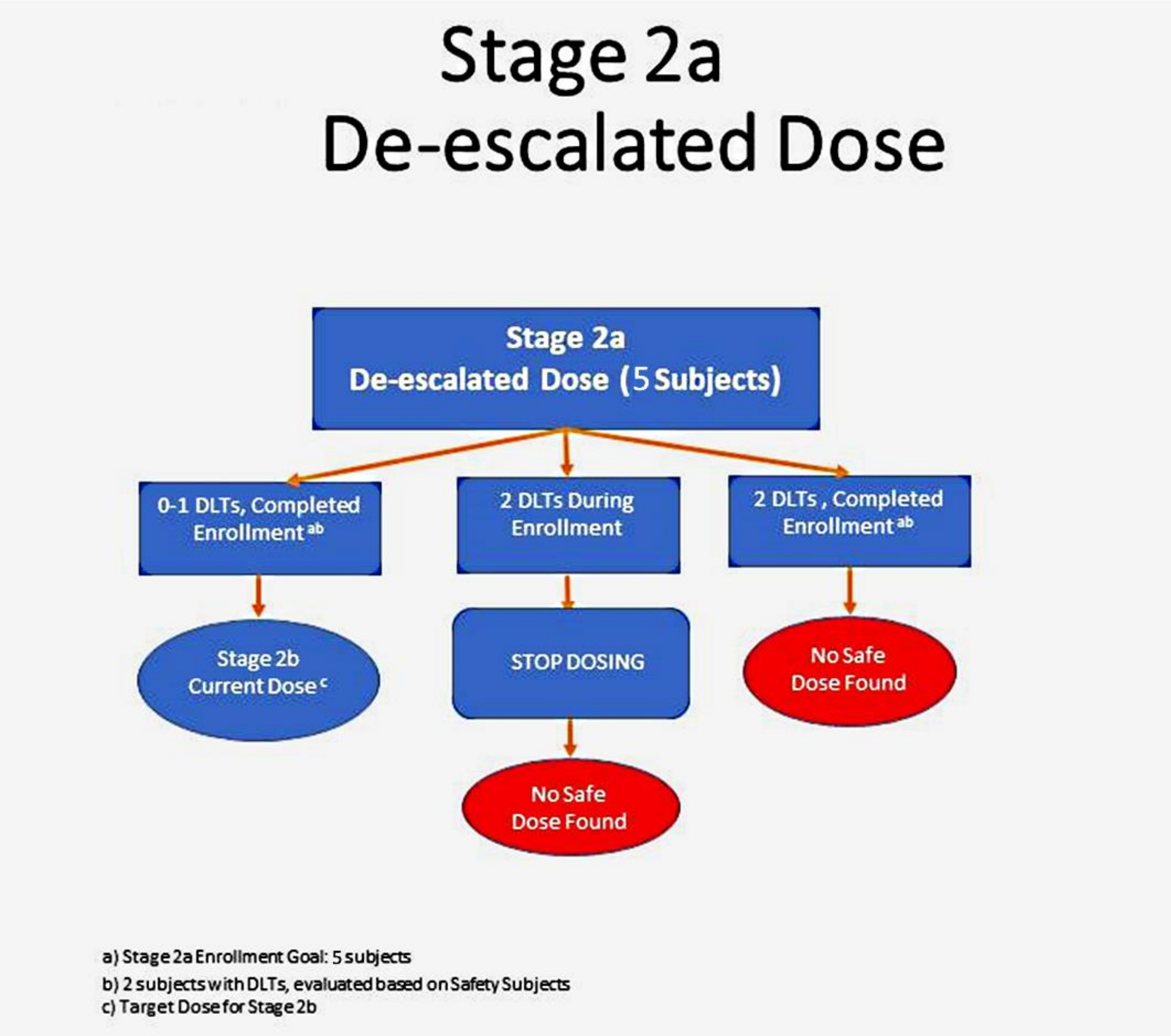

Figure 4. Stage 2a safety scheme in case of de-escalated dose decision

Stage 2b: In this stage both safety and efficacy will be evaluated. All subjects will be monitored for possible DLTs (Stage 2b – All Doses flowchart).

Stopping Rules at Stage 2b:
If 3 subjects with DLTs, no additional subjects will be dosed.

Efficacy Rules at Stage 2b:
Efficacy rules are described in the statistical plans for this stage since it is end of the study.

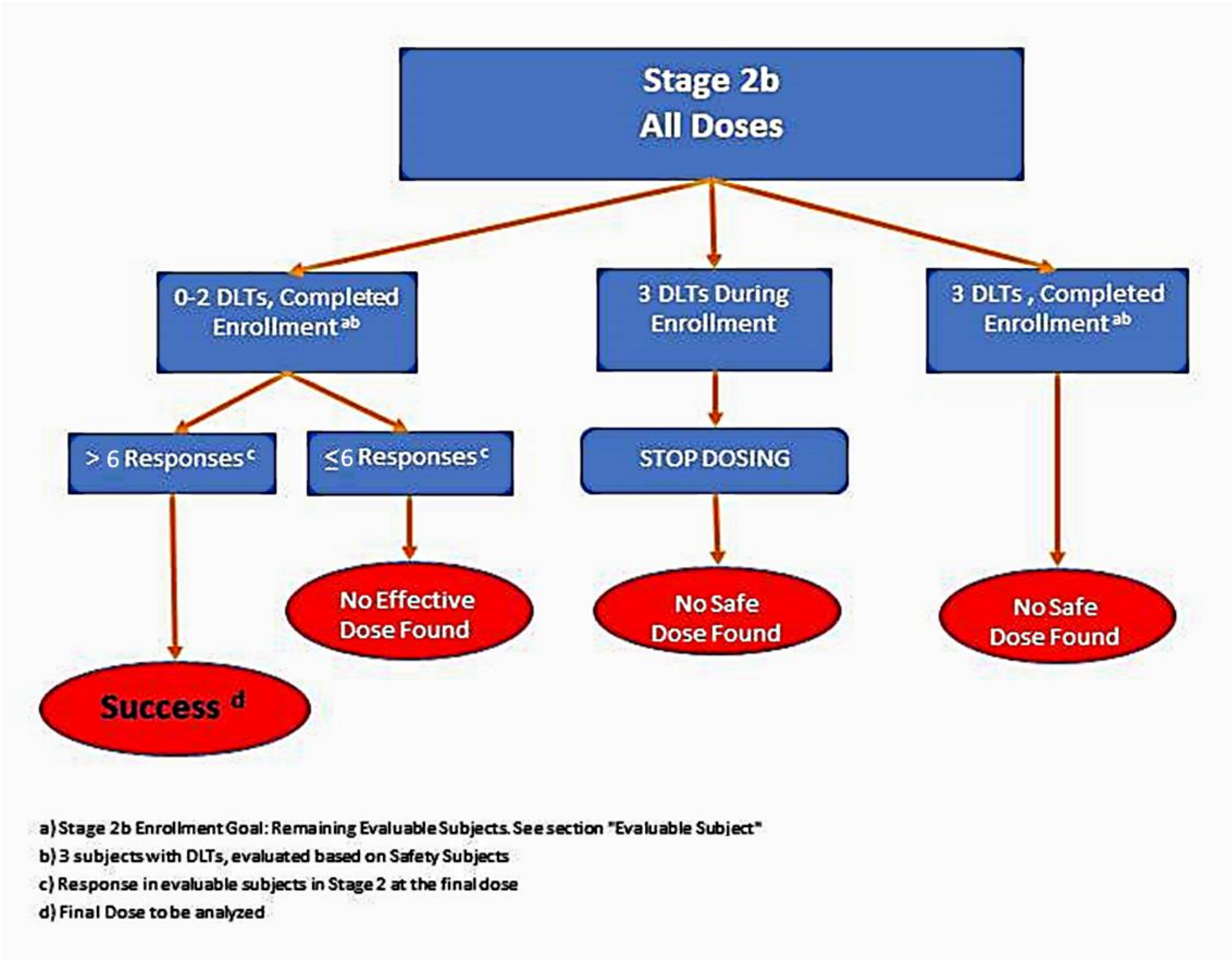

Figure 5. Stage 2b safety and efficacy scheme

Note: The surgery is to be performed no later than 42 days post Visit 1.

All subjects will subsequently undergo standard of care, surgical ablation of the tumor and satellite nodes, and possibly additional radiotherapy and chemotherapy. All subjects will be followed for up to 6 months post-surgery at 3 visits: 1 month, 3 months, and 6 months post-surgery for loco-regional response and safety. Follow-up visits may coincide with standard of care follow-up visits, if deemed appropriate by the Principal Investigator.

6.2 Discussion of Trial Design

This study is intended to establish the first-in-human safety and efficacy observations and the optimal dose of the PRV111 (Cisplatin Transmucosal System) prior to the surgical removal of SCC tumors of the oral cavity.

Based on discussions with Food and Drug Administration (FDA), only subjects with T1 or T2 tumors are planned in this study. FDA's concerns are based on safety in a first-in-human study.

7 SELECTION OF SUBJECTS

7.1 Inclusion Criteria

Subjects must meet all of the following inclusion criteria to be eligible for the trial:

1. Adult subjects, men and women, defined by age ≥18 years at the time of screening
2. Pathologically proven and clinically confirmed T1 (≤2 cm) or T2 (≥2 cm but ≤4 cm) SCC of the lip or oral cavity (anterior 2/3 of the tongue, floor of mouth, lower and upper gingiva, salivary gland, hard palate, and buccal mucosa)
3. Tumor must be easily accessible, with no evidence of infection or active bleeding, encroaching major vessels or clinical evidence of neural invasion. Tumor should not have been previously irradiated
4. Tumor must be amenable to surgical resection no later than 42 days post Visit 1
5. Clinically and/or radiologically measurable tumor
6. Eastern Collaborative Oncology Group Performance Status (ECOG PS) of ≤2
7. Adequate renal function as demonstrated by renal creatinine clearance >50 mL/min (calculated using the Cockcroft-Gault formula)
8. Adequate organ function defined as: ANC ≥1000/µL, platelet ≥100,000/µL; Hb ≥9.0 g/dL; AST and ALT ≤2.5 times the upper limit of normal (ULN), serum bilirubin ≤1.5 times the ULN or direct bilirubin ≤ULN for subjects with total bilirubin >1.5 times the ULN
9. Candidates for standard of care treatment consisting of surgery
10. Male and female subjects of childbearing potential must agree to use 2 methods of effective contraception from Screening during the entire duration of the study and for at least 30 days after the final dose of investigational product. An appropriate birth control is defined as barrier methods with spermicides, oral or parenteral contraceptives and/or intrauterine devices, or naturally or surgically sterile (with documentation in the subject's medical records). Postmenopausal women are defined as presenting at least 12 months' natural spontaneous amenorrhea, or at least 6 weeks following surgical menopause (bilateral oophorectomy). Females of childbearing potential must be non-lactating and have a negative serum hCG within 14 days of treatment initiation
11. Absence of any serious underlying medical conditions which could impair the ability of the subject to participate in the study
12. Have a life expectancy of >3 months
13. Willing and able to provide written informed consent
14. Able to return to study site for treatment and follow-up visits as defined in the Protocol

7.2 Exclusion Criteria

Subjects meeting any of the following criteria are ineligible for the trial:

1. Known distant metastasis of the SCC of the oral cavity

2. Systemic chemotherapy for the treatment of SCC of the head and neck less than 2 years prior to Screening
3. Concurrent documented malignancy, with the exception of localized SCC of the skin
4. Exposure to any investigational agent within 3 months prior to Screening
5. Known allergy or hypersensitivity to platinum-containing agents, or known intolerance to a prior platinum-containing agent, or to any of the excipients, which, in the judgement of the physician will preclude re-exposure to platinum-containing agent
6. Active, uncontrolled infection requiring systemic therapy, such as but not limited to HIV, Hepatitis B or C
7. Uncontrolled intercurrent illness that would risk subject safety, interfere with the objectives of the Protocol, or limit subject compliance with study requirements, as determined by the Investigator
8. Known or suspected pregnancy, planned pregnancy, or lactation
9. Any medical or psychiatric condition that may compromise the ability to give written informed consent

7.3 Criteria for Individual Subject Discontinuation of Protocol Therapy

Subjects should be discontinued from protocol therapy, while follow-up as per protocol, for the following reasons:

- Completion of the scheduled PRV111 therapy
- Meets 1 of the stopping rules for an individual subject (Section 8.2)
- Pregnancy
- Noncompliance with trial or follow-up procedures
- Withdrawal at the Investigator’s request for any reason that the Investigator believes continuation of study drug therapy would not be in the subject’s best interest
- Subjects may be withdrawn at any time for drug intolerability, disease progression, availability of other therapeutic options, or subject judgment

7.4 Criteria for Individual Subject Discontinuation from the Trial

Subjects may withdraw from further participation in the study at any time and for any reason without prejudice. The degree to which a subject withdraws can vary and efforts will be made to collect important safety data if feasible and the subject agrees. A subject will be considered to be withdrawn from the trial for the following reasons:

- Withdrawn consent from all treatment and follow-up
- Death
- Lost to follow-up
- Termination of the trial by the Sponsor

7.5 Handling of Subject Withdrawals or Subject Discontinuation of Study Intervention

If a subject withdraws consent for the treatment, the Investigator will make every effort to complete all final evaluations and procedures required by the Protocol. The subject will be asked to return to the clinic for a follow-up visit to assess overall safety prior to study completion. In the event a subject withdraws for safety reasons (adverse event/serious adverse event [AE/SAE], National Cancer Institute's (NCI) Common Terminology Criteria for Adverse Events [CTCAE] toxicity related events, etc.), every effort will be made to continue to follow-up with the subject until the documented condition resolves to protocol-established grades.

The reasons for discontinuation of dosing must be clearly documented on the case report form (CRF).

7.6 Premature Termination or Suspension of Study

The study may be suspended or prematurely terminated if there is sufficient reasonable cause. Written notification, documenting the reason for study suspension or termination, will be provided by the suspending or terminating party to Privo, Institutional Review Board (IRB), US FDA, and appropriate committees. If the study is prematurely terminated or suspended, the Principle Investigator (PI) will be required to promptly inform the IRB and provide the reason(s) for the termination or suspension.

Circumstances that may warrant termination include, but are not limited to:

- Determination of unexpected, significant, or unacceptable risk to subjects
- Determination of futility
- Excessive study treatment toxicity leading to delays in the scheduled surgery

7.7 Replacement of Subjects

Subjects will not be replaced.

8 TREATMENTS

Study treatment is to be administered only to subjects who have provided informed consent and have met the criteria outlined in Sections 7.1 and 7.2.

8.1 Treatments Administered

8.1.1 Description of Test Product

PRV111 (Cisplatin Transmucosal System) (referred to as PRV111) consists of:

- PRV111 4 cm^2 patch containing 2 mg of cisplatin (0.5 mg/cm^2) backed with a pressure sensitive acrylic adhesive non-woven fabric backing.
- PE Powder for Reconstitution (4 mL borosilicate glass vial containing 50 mg (on anhydrous basis) each of sodium cholate hydrate, sodium taurodeoxycholate hydrate, sodium deoxycholate and sodium glycodeoxycholate) (intended for reconstitution in 1 mL US Pharmacopeia Water for Injection, 50 mg/mL).

8.1.2 *Packaging*

The PRV111 4 cm^2 patch and PE Powder for Reconstitution is packaged in a self-sealing pouch, 2.25” x 4.0” (constructed of medical grade paper). The primary pouch is enclosed in a secondary heat sealed triple laminated Mylar foil pouch (to protect the patch from light and moisture).

All components are packaged in a quaternary cardboard carton which is labeled appropriately.

8.1.3 *Labeling*

The proposed labeling of the PRV111 patch (Cisplatin Transmucosal System) study drug is described in the Pharmacy Manual.

8.1.4 *Preparation*

Refer to the Pharmacy Manual for a complete description of preparation. Each investigational site, and all site personnel handing the PRV111 patch, will be trained on application and handling.

8.1.5 *Dosing and Administration*

Each treatment visit will include 1 mL application of the PE. Starting 5 minutes after application of the PE, 1 or 2 patches (depending on tumor size) will be applied. For Stage 1, a total of 3 treatment applications of patches will occur. These successive applications will span approximately 35 minutes. The 3 applications provide a total of 6 mg of cisplatin per visit (12 mg if 2 patches are applied at the same time).

In Stage 2 (Stage 2a and Stage 2b), subjects will receive 2, 3, or 5 treatment applications at each of the 4 planned treatment visits, depending on the results of Stage 1.

Refer to the Pharmacy Manual for detailed instructions on applying the PRV111 patches.

Table 2 presents definition used for dosing and treatment in this study. Treatment is expected to be approximately 35 minutes for each treatment visit.

Table 2. Dosing Terminology

Term	Definition
Treatment visit	Two to 6 days apart, during which PRV111 patches will be administered.
Treatment application	Three patch applications will occur during each treatment visit in Stage 1.<br>Two, 3, or 5 patch applications will occur during each treatment visit in Stage 2.

8.2 Dose-limiting Toxicity (Stopping Rules for an Individual Subject)

Toxicity will be graded and recorded according to the NCI CTCAE version 4.03 (US Department of Health and Human Services, 2010), which are considered at least possibly related to PRV111 Patch. A DLT is defined as a clinically significant Treatment-Emergent AE

(TEAE) or laboratory abnormality unrelated to disease progression, concurrent illness, or concomitant therapy and that meets any of the following criteria:

- Grade 3 or 4 non-hematologic toxicity excluding alopecia
- Grade 3 or 4 nausea, vomiting, or diarrhea, lasting >48 hours, despite standard prophylaxis and/or treatment (refer to Section 8.7 for a list of suggested anti-emetic medications)
- Grade 4 neutropenia lasting >5 days.
- Febrile neutropenia, defined as absolute neutrophil count <1000/mm^3 with a single temperature of >38.3°C (101°F) or a sustained temperature of ≥38°C (100.4°F) for more than 1 hour.
- Grade 4 thrombocytopenia or thrombocytopenia with clinically significant bleeding (ie, bleeding that requires blood or platelet transfusion or other medical intervention, or that may cause disability or death, such as cerebral hemorrhage).
- Any toxicity that precludes administration of at least 3 of the 4 planned applications.
- Grade 4 local toxicity- oral mucositis according to World Health Organization scale:
  - Grade 0 - No oral mucositis
  - Grade 1 - Erythema and soreness
  - Grade 2 - Ulcer, able to eat solids
  - Grade 3 - Ulcers, requires liquid diet (due to mucositis)
  - Grade 4 - Ulcers, alimentation not possible (due to mucositis)
- Any related AE leading to surgery delay for more than 96 hours is a DLT

8.3 Dose Modification

If in the clinical judgment of the Medical Monitor and the PIs, clinically relevant safety issues are identified, the dose regimen (number of patches/visit and/or number of visits) may be reduced at any time during the conduct of the study.

8.4 Dose Interruption

At the Investigator's discretion, study drug may be interrupted, for reasons not related to toxicity (eg, intercurrent illness, subject scheduling conflict). Such interruption may not last more than 144 hours and may not occur more than 1 time during the subject's treatment schedule.

Subjects who experience an AE(s) requiring interruption of study drug for ≥144 hours will be removed from treatment.

Subjects who experience a DLT will be terminated from dosing, but will remain in the study for the purpose of data collection. The DLT period will be defined as the start of treatment to 14 days after the last treatment application or the time of surgery, whichever is first.

8.5 Study Drug Handling and Accountability

The Investigator (or designee, ie, study pharmacist) at the site will be responsible for handling all study drug supplies and maintaining required documentation. The Investigator must maintain complete and current inventory and dispensing records.

The Investigator (or designee) will:

- Ensure study drug supplies (PRV111 study kits) are stored between 2º and 8º C (in an appropriately controlled limited-access room in a secure location.
- Dispense the appropriate number of study patches.
- Record pertinent information regarding the number of study kits used (eg, subject identification code, date of use, number of patches used, etc.) on the Drug Dispensing Log, or other appropriate inventory form. This inventory will be maintained throughout the trial and will be periodically reviewed by a Sponsor representative.
- All used and unused patches during a treatment visit must be sent to the pharmacokinetics (PK) lab. They should be separated into properly labeled sealing plastic bags. The used patches will be processed by the PK lab to assess the residual levels of cisplatin of each application. Preparation for shipping and shipping logistics will be found in the Laboratory Manual.

8.6 Study Drug Disposal

Periodically throughout and at the conclusion of the trial, an inventory of unused study drug kits will be conducted by a Sponsor representative. All unused study drug materials during a treatment visits must be sent to the PK lab in labelled, sealing plastic bags and they will be disposed in an appropriate manner according to the laboratory policy.

At the treatment completion, any remaining unopened and unused study drug in the kit will be sent back to the Sponsor. Preparation for shipping and shipping logistics will be found in the Pharmacy Manual. At the termination of the trial, a final drug accountability review and reconciliation must be completed and any discrepancies must be investigated and their resolution documented.

8.7 Concomitant Medications and Therapy

Concomitant medications and therapies include other prescription medications, over-the-counter drugs, or dietary supplements that a study participant takes in addition to the investigational product. Based on FDA’s recommendation, anti-emetic prophylaxis will be mandatory.

Symptomatic anti-emetics will be used during the study.

All subjects will receive emesis prophylaxis, administered approximately 30 minutes before PRV111 treatment. The drug used may be either ondansetron or promethazine, the dose to be determined at the discretion of the PI. After treatment, subjects may self-administer orally or via feeding tube approximately every 12 hours for 24 to 48 hours for persistent nausea. Alternatively, subjects may receive promethazine 12.5 mg orally approximately 60 minutes before administration of the PRV111 treatment. After treatment, subjects may self-administer promethazine 12.5 mg approximately every 4 to 6 hours for 24 to 48 hours for persistent nausea.

Subjects with diarrhea will receive appropriate interventions/medications at the discretion of the treating physician consistent with current clinical practice.

Based upon the Investigator's judgment, the subject might need topical anesthetic (ie, lidocaine, Magic Mouthwash) to ameliorate discomfort or prevent accidental touching and removal of the patch.

8.8 Drugs Contraindicated with PRV111

Refer to the package insert for Platinol-AQ Injection for a list of drugs that interact with cisplatin.

Plasma levels of anticonvulsant agents may become subtherapeutic during cisplatin therapy.

9 VARIABLES ASSESSED

Refer to Table 1for scheduling of study assessments.

9.1 Eligibility Only Assessments

Refer to Table 1for for timing of assessments.

9.1.1 Confirmation of Squamous Cell Carcinoma

Subjects eligible for enrollment must have histological confirmation of SCC.

HPV, the possible etiology of SCC, will be ascertained using immunohistochemistry for p16 overexpression per institutional standards. Results are not an eligibility requirement, but should be available at some point during the subject's participation in the study.

9.1.2 Tumor Staging

The size or direct extent of the primary tumor intended for surgical resection will be assessed for eligibility (National Cancer Institute, 2015):

- Tx: tumour cannot be assessed
- Tis: carcinoma in situ
- T0: no evidence of tumour
- T1, T2, T3, T4: size and/or extension of the primary tumour

9.1.3 Demographics, Medical and Medication History

Demographic information, including age, sex, race, history of tobacco and alcohol use, and medical history will be obtained by interviewing the subject.

Baseline characteristics consist of a complete medical history and detailed disease history. Medical history includes prior and ongoing medical diagnoses and conditions, surgical procedures not related to the primary diagnosis, and medications taken within the previous

28 days. Disease history is a history of the primary diagnosis and should include the date of initial oral SCC diagnosis and staging; for recurrent cases- date of most recent relapse and/or re-staging; histologic diagnosis; prior treatments (eg, chemotherapy, radiotherapy); and any current signs and symptoms related to the primary diagnosis.

9.1.4 Pregnancy Test

A serum pregnancy test to establish eligibility for female subjects of childbearing potential will be performed. In addition, a negative urine pregnancy test must be documented within 7 days prior to the first treatment visit, and at Visit 5.

9.1.5 Eastern Collaborative Oncology Group

Performance status will be assessed using ECOG PS criteria (Table 3).

Table 3. Eastern Collaborative Oncology Group Performance Status

Grade	ECOG PS
0	Fully active, able to carry on all pre-disease performance without restriction
1	Restricted in physically strenuous activity but ambulatory and able to carry out work of a light or sedentary nature, eg, light house work, office work
2	Ambulatory and capable of all self-care but unable to carry out any work activities; up and about more than 50% of waking hours
3	Capable of only limited self-care; confined to bed or chair more than 50% of waking hours
4	Completely disabled; cannot carry on any self-care; totally confined to bed or chair
5	Dead

Abbreviations: ECOG PS=Eastern Collaborative Oncology Group Performance Status.

9.2 Efficacy Assessments

Refer to Table 1for timing of assessments.

9.2.1 Tumor Photography and Measurements

The primary lesion will be photographed before PRV111 treatment. This will be done to allow for a clinical assessment of response to treatment. For details refer to Study Manual for instructions.

The tumor will be measured in cm to 1 digit to the right of the decimal point using a disposable plastic ruler or caliper. Three dimensions must be recorded (Length x Width x Height).

Note: If tumor or cancerous lesion is flat, a small number (0.05 cm) will be recorded for the Height. If the tumor is a concave, a negative Height is recorded. If the height is an unmeasurable dent, it can be recorded as (-0.05 cm).

The following formula will be used to calculate tumor volume (cm^3)

$$L \times W \times H \times \pi/6$$

9.2.2 *Response and Non-Response (Tumor Volume Change from Baseline Criteria)*

The terms "response" and "non-response" will be defined according to the tumor volume change from baseline (CFB) criteria:

PCFB = % tumor volume change from the baseline

Response will be defined as CR, PR or SR. Non-Response will be defined as SD or PD (Table 4).

Table 4. Clinical Response to Treatment Based on Tumor Volume Change*

Complete Response (CR)	Disappearance of primary lesion.
Partial Response (PR)	PCFB: ≥30% to 69% decrease in the total tumor volume shrinkage (as determined by clinical measurement, taking as reference the baseline volume.
Strong Response (SR)	PCFB: ≥70% to 99% decrease in the total tumor volume shrinkage (as determined by clinical measurement, taking as reference the baseline volume.
Progressive Disease (PD)	PCFB: At least a 20% increase tumor volume as determined by clinical measurement taking as reference the baseline volume (Note: the appearance of one or more new lesions is also considered progression).
Stable Disease (SD)	Neither sufficient shrinkage to qualify for PR nor sufficient increase to qualify for PD, taking as reference the volume determined by clinical measurement at baseline

* Necrosis/inflammation will not be included towards clinical tumor measurements.

9.2.3 *Computed Tomography or Magnetic Resonance Imaging of the Head and Neck*

A computed tomography scan (CT) or magnetic resonance imaging (MRI) of the head and neck will be performed. For each subject, the same imaging modality should be used at Screening and Visit 5 for consistency.

9.2.4 *Histopathology Response to Treatment*

Histological samples obtained (Laboratory Manual) at Baseline and from the surgical tumor excision will be compared. A pathologist will assess tumor margins and regression defined as: reduction of tumor density, necrosis, fibrosis, inflammatory infiltrates, histocyte accumulation, and giant cell reactions.

Histological response will be assessed by the examination of at least 2 slices/cm of the resected tumor specimen. The pathological response will be graded according to Table 5.

Table 5. Pathological Response to Treatment

Grade 1	Extensive residual cancer with no evident tumor regression (poor or no response, more than 90% of viable tumor).
Grade 2	Residual cancer with evident tumor regression, but still more than half of tumor left (PR, approximately 51%-89% of viable tumor).

Grade 3	Great response, with less than half tumor left, but more than single cells or rare small groups of cancer cells (PR, approximately 11%-50% of viable tumor).
Grade 4	Single cells or rare small groups of cancer cells (near CR, ≤10% of viable tumor).
Grade 5	No viable cancer cells (CR).

Abbreviations: CR=complete response; PR=partial response.

9.2.5 Loco-regional Control

Subjects will be followed for up to 6 months, and the incidence of local and regional (nodes) tumor recurrence will be recorded.

9.3 Safety Assessments

Refer to Table 1for timing of assessments.

9.3.1 Physical Examination, Vital Signs, Weight

A complete physical examination will be performed by the Investigator or Sub-investigator. The physical examination will include a review of all body systems (head, eyes, nose and throat, cardiovascular, respiratory, gastrointestinal, musculoskeletal, neurological, lymph nodes, dermatological, extremities/joints), and weight.

Seated blood pressure (mmHg), pulse (beats per minute) and temperature (degrees Celsius) will be measured after the subject has been sitting for at least 3 minutes.

Neuropathy and ototoxicity will be assessed via clinical examination since cisplatin can potentially cause neurotoxicity and ototoxicity. Questions will be asked to document symptoms such as tinnitus, pain in the extremities, or loss of sensation in the eCRFs.

9.3.2 Oral Cavity Examination

A systematic and thorough visual and manual oral examination will be performed. This will include a visual examination of all the soft tissues of the mouth, including manual extension of the tongue to examine its base, a bi-manual palpation of the floor of the mouth, and a digital examination of the borders of the tongue.

Special attention must be given to the application site and the tumor, with documentation of any local adverse reactions, including documentation with photographs if warranted.

9.3.3 Neck Lymph Nodes Examination

A cervical and local lymph nodes examination will be performed.

9.3.4 Safety Laboratory Assessments

Clinical laboratories to be assessed will be as in Table 6.

Table 6. Clinical Laboratories

<table>
<tr><th>COMPLETE BLOOD COUNT</th><th colspan="2">CHEMISTRY PANEL</th></tr>
<tr><td>• hematocrit
• hemoglobin
• platelet count
• red blood cell count
• white blood cell count and differential (absolute and percentage); neutrophils, lymphocytes, monocytes, eosinophils, basophils, and reticulocyte count</td><td rowspan="3">• alanine aminotransferase
• albumin
• alkaline phosphatase
• aspartate aminotransferase
• bilirubin (direct and total)
• blood urea nitrogen
• calcium
• CO2</td><td rowspan="3">• chloride
• creatinine[a]
• gamma glutamyl transferase
• glucose
• magnesium
• potassium
• sodium
• total protein</td></tr>
<tr><th>URINALYSIS</th></tr>
<tr><td>• urinalysis dipstick with reflex microscopic examination for abnormalities</td></tr>
</table>

[a] Creatinine Clearance will be used to assess renal function using Cockcroft-Gault Equation

9.3.5 Electrocardiograms

Electrocardiograms (ECGs) will be evaluated for clinically relevant changes from Baseline.

9.3.6 Adverse Event Definitions/Reporting Requirements

An AE (also known as an "adverse experience") is defined as any untoward medical occurrence associated with the use of a drug in humans, whether or not considered drug related (US Code of Federal Regulations [CFR] 312.32).

An AE can therefore be any unfavorable and unintended sign (including an abnormal laboratory finding), symptom, or disease temporally associated with the use of the drug, without any judgment about causality.

All clinical AEs noted during the study will be reported on the appropriate AE page of the CRF.

AEs that are serious, unexpected, and at least possibly related to study participation will be reported to the IRB and to the US FDA within the timelines described below. All other AEs, such as those that are expected, or definitely not related to the study participation, are to be reported per the IRB's policy and to FDA as part of regular data submission at the time of annual Investigational New Drug report.

9.3.6.1 Expected Events

Expected events are those that have been previously identified as resulting from administration of the study drug. An AE is considered unexpected, for expedited reporting purposes only, when either the type of event or the severity of the event is not listed in the current Investigator's Brochure (IB).

9.3.6.2 Suspected Adverse Reaction

A suspected adverse reaction is a subset of all AEs for which there is a reasonable possibility (ie, evidence to suggest a causal relationship between the drug and the AE) that the drug caused the

event. Suspected adverse reaction implies a lesser degree of certainty about causality than adverse reaction.

9.3.6.3 Adverse Reaction

An adverse reaction is a subset of all suspected adverse reactions, and is defined as any AE caused by a drug.

9.3.6.4 Adverse Device Effects

Adverse device effects are those AEs thought to be related to the use of an investigational medical device. This definition includes AEs resulting from insufficient or inadequate instructions for use, deployment, implantation, installation, or operation, or any malfunction of the investigational medical device. This definition also includes any event resulting from use error or from intentional misuse of the investigational medical device.

An Unanticipated Adverse Device Effect is an adverse device effect, which by its nature, incidence, severity or outcome has not been identified in available information on the device.

Additional device issues, malfunctions or misuse will be documented in the electronic CRF.

9.3.6.5 Serious Adverse Event Definitions / Reporting Requirements

9.3.6.5.1 Serious Adverse Event or Serious Suspected Adverse Reaction

An AE or suspected adverse reaction is considered serious if, in the view of either the Investigator or Sponsor, it results in any of the following outcomes:

- Death. This includes any death that occurs while the subject is on study and/or within 30 days after the last dose of study drug. An autopsy will be requested, and if performed, results will be submitted to the Sponsor.
- A life-threatening AE. An AE or suspected adverse reaction is considered life-threatening if, in the view of either the Investigator or Sponsor, its occurrence places the subject at immediate risk of death. It does not include a reaction that had it occurred in a more severe form, might have caused death.
- Inpatient hospitalization or prolongation of existing hospitalization. In the absence of an AE, hospitalization or prolongation of hospitalization should not be reported as a serious AE (SAE) in the following situations:
    - Hospitalization or prolongation of hospitalization is needed for a procedure required by the Protocol.
    - Hospitalization or prolongation of hospitalization is part of routine procedure followed by study center.
    - Hospitalization for survey visits or annual physicals.
    - For a hospitalization planned (and documented) before the start of the study for a pre-existing condition which has not worsened.

- A persistent or significant incapacity or substantial disruption of the ability to conduct normal life functions.
- A congenital anomaly/birth defect.

Important medical events that may not result in death, be life-threatening, or require hospitalization may be considered serious when, based upon appropriate medical judgment, they may jeopardize the subject and may require medical or surgical intervention to prevent one of the outcomes listed in this definition. Examples include allergic bronchospasm requiring intensive treatment in an emergency room or at home, blood dyscrasias or convulsions that do not result in inpatient hospitalization, or the development of drug dependency or drug abuse.

9.3.6.5.2 Pregnancy

For safety reporting purposes, any pregnancy occurring during the study will be considered an "important medical event" (Section 9.3.6.5.1). If a study subject becomes pregnant while participating in the study, the study drug will be discontinued immediately and the pregnancy will be reported to the Sponsor. The Investigator will then report follow-up information to the Sponsor regarding the course of the pregnancy, including perinatal and neonatal outcome, regardless of the fact that the subject has discontinued the study. Once the newborn is determined to be healthy, as defined by and agreed upon by the Sponsor and Investigator, additional follow-up will no longer be required.

9.3.6.5.3 Serious Unexpected and Suspected Adverse Reaction

An unexpected AE (or serious and unexpected suspected adverse reaction [SUSAR]) is any AE or suspected adverse reaction that is not listed in the IB or is not listed at the specificity or severity that has been observed, if an IB is not required/available, is not consistent with the risk information described in the general investigational plan. This also refers to AEs or suspected adverse reactions mentioned in the IB as occurring with a class of drugs or as anticipated from the pharmacological properties of the drug but are not specifically mentioned as occurring with the study drug. A SUSAR requires mandatory expedited reporting to the applicable regulatory authority(ies) (Section 9.3.6.8). The Sponsor will forward the formal notification describing the SUSAR to Investigators. Each Investigator must then notify his/her IRB of the SUSAR, in accordance with the IRB's policy.

The Sponsor should be notified within 24 hours of the Investigator becoming aware of a SUSAR and the event documented on the CRF.

9.3.6.6 Recording Adverse Events

All AEs (including SAEs) within the 5 pre-surgery visits are to be accurately recorded on the AE/SAE page(s) of the CRF. In addition, any SAEs within 30 days of visit 5 will need to be recorded. The Investigator will carefully evaluate each AE to determine:

- Duration (start and end dates)
- Severity (grade)

- Seriousness
- Relationship to study drug (Not Related, Possible, Definite)
- Action taken (none, study drug modification, medical intervention)
- Outcome (resolved without sequelae, resolved with sequelae, ongoing)

The Investigator will evaluate the recorded AEs and SAEs with regard to maximum intensity and relationship to study drug. Maximum intensity should be assigned using one of the severity grades as outlined in the NCI CTCAE v4.0. If the AE is not specifically listed in CTCAE v4.0, the following grades will be used:

- Grade 1: mild
- Grade 2: moderate
- Grade 3: severe
- Grade 4: life-threatening or disabling
- Grade 5: death

9.3.6.7 Determining Relationship of Adverse Events to Study Drug

The Investigator must attempt to determine if an AE or SAE is related to the use of the study drug. This relationship should be described as follows:

Possible The event follows a reasonable temporal sequence from administration of the study drug and the event follows a known response pattern to the study drug BUT the event could have been produced by an intercurrent medical condition which, based on the pathophysiology of the condition and the pharmacology of the study drug, would be unlikely to be related to the use of the study drug OR the event could be the effect of a concomitant therapy.

Definite The event follows a reasonable temporal sequence from administration of the study drug, the event follows a known response pattern to the study drug and based on the known pharmacology of the study drug, the event is clearly related to the effect of the study drug.

Not Related Based on the evidence available, causality is not possible.

9.3.6.8 Reporting Deaths and Other Serious Adverse Events

9.3.6.8.1 Events to Be Reported and Timeframe

Any death or other SAE experienced by the subject during treatment or within 30 days of receiving study drug, regardless of expectedness, relationship to study drug, or any death that occurs more than 30 days after receiving study drug and is believed to be study drug-related, must be promptly reported to the Sponsor (*within 24 hours (of a working day) of the Investigator becoming aware of the event*) by telephone or email transmission.

9.3.6.8.2 Governing Regulatory Requirements

Compliance with this request for prompt reporting is essential in that the Sponsor is responsible for informing the FDA and other regulatory authorities as well as all other participating Investigators of the event.

Under FDA ruling (US CFR, Title 21 CFR Part 312.32) and the International Council on Harmonisation (ICH) Guidelines for Clinical Safety Data Management Definitions and Standards for Expedited Reporting, the Sponsor is required to submit written documentation, in the form of an IND Safety Report, on the following:

- SUSARs
- Unexpected fatal or life-threatening suspected adverse reactions
- Findings from other studies that suggest a significant risk to humans exposed to drug
- Findings from animal or in vitro testing that suggest a significant risk to humans exposed to drug
- Increased rate of occurrence of serious suspected adverse reactions from what is reported in the IB or the Protocol

Written submission must be made by the Sponsor to the FDA/other regulatory authorities (and by the Investigator to the IRB) as soon as possible, and in no event later than **<u>15 calendar days</u>** after the Sponsor determines that the information qualifies for reporting. The Sponsor shall also inform all Investigators.

In addition, the Sponsor is further required to report, by either telephone or facsimile transmission or in writing to the FDA/other regulatory authorities the occurrence of any unexpected fatal or life-threatening event associated with the use of the drug (SUSARs) no later than **<u>7 calendar days</u>** after notification of the event, followed by a written report **<u>no later than 15 calendar days</u>** after the initial report receipt date. The Sponsor shall also inform all Investigators.

The Sponsor will provide expedited reports of the following SUSARs to the Investigators for reporting to their IRB:

- SUSARs that have arisen in the clinical trial that was assessed by the IRB.
- SUSARs that have arisen in other clinical trials of the same Sponsor and with the same medicinal product, and that could have consequences for the safety of the subjects involved in the clinical trial that was assessed by the IRB.

Investigators are required to promptly report to the IRB all unanticipated problems involving risk to human subjects, including AEs that should be considered unanticipated problems (21 CFR 312.53(c)(1)(vii), 312.66, and 21 CFR 56.108(b)(1)).

9.3.6.9 Initial Information Provided by the Investigator

The Investigator must transmit sufficient initial information to the Sponsor (or designee) should an SAE report meet the criteria for completion of an IND Safety Report. As much of the following information about the subject and the event will be requested:

- Subject identification code, gender, and age or date of birth
- Underlying diagnosis and extent of disease
- Lot number and expiration date of study drug (if available)
- Dose, route, frequency, and duration of study drug administered
- Date of last study drug administration
- Description of event, including date of onset and duration
- Date of death (if applicable)
- Intervention(s) required
- Concomitant therapy (including regimens and indications)
- Pertinent laboratory data/diagnostic study (including dates)
- Pertinent medical history
- Study drug status (dose interrupted, discontinued)
- Did event abate after interruption of study drug administration (if applicable)?
- Did event recur after study drug was reintroduced (if applicable)?
- Severity of the AE
- Relationship of the AE to study treatment
- Outcome of the AE

9.3.6.10 Follow-up Information

Follow-up data concerning the SAE (eg, diagnostic test reports, physician's summaries, etc.) must also be submitted to the Sponsor as they become available by email as soon as they become available, and until the event resolves or stabilizes.

9.3.6.11 Serious Adverse Event that are Unexpected and related Serious Adverse Reaction Review that have a Potential Impact on Trial Conduct

The Investigator will review each SAE report and evaluate further the relationship of the SAE to study drug and to the subject’s underlying disease.

Based on the Investigator’s and Sponsor’s assessment of causality of the AE and discussions with the Medical Monitor, a decision will be made by the Sponsor concerning the need for further action with respect to the future conduct of the study. The primary consideration governing further action is whether new findings affect the safety of other subjects participating in the clinical study. If the discovery of a new SAE related to study drug raises concern over the safety of its continued administration to subjects, the Sponsor will take immediate steps to notify FDA, other regulatory authorities, and all Investigators participating in clinical studies with the study drug.

Further action required may include any of the following:

- Alteration of the existing research program by modification of the Protocol
- Discontinuation or suspension of the study
- Alteration of the informed consent process by modification of the existing consent form and informing current study participants of new findings
- Modification of previously identified expected suspected adverse reaction lists to include SAEs newly identified as study drug-related

9.4 Pharmacokinetics Assessment

Whole blood samples will be assessed for PK parameters area under the curve (AUC), maximum concentration (C_{max}), minimum concentration (C_{min}), time to maximum concentration (T_{max}), and half-life ($t_{1/2}$), and total platinum content for all subjects (See Laboratory Manual for sample collection and shipping instructions). Tumor and lymph node cis platinum content may be provided as well.

9.5 Technical Success Assessments

Technical assessment data will include:

- Taste of the PE solution
- Taste of the PRV111 patch
- Adhesion, including the time of adhesion of PRV111 patch
- PRV111 patch peel-off
- Residual cisplatin level in the patch after the application period. All PRV111 patches will be assessed for the amount of residual cisplatin in each patch after each application period. If the product detaches prematurely, the residual cisplatin will be assessed and the time of detachment will be noted.

9.6 Timing of Assessments

Refer to Table 1for a schedule of events.

9.6.1 Screening (Visit 0)

- Visit and all historical exams/tests must occur within 28 days prior to initial treatment (Visit 1). Confirmed SCC
- Obtain and document consent from potential subject on Informed Consent Form (ICF)
- Review inclusion/exclusion criteria to determine eligibility
- Review demographics, medical and medications history to determine eligibility based on inclusion/exclusion criteria
- Obtain or perform a general physical examination (must be within 14 days of first dose) and check VS
- Obtain historical or perform CT of the chest (with or without contrast)
- Perform a systematic and thorough visual and manual examination of the oral cavity mouth and cervical and local lymph nodes examination
- Obtain historical or perform an ECG

- Obtain historical or perform CT or MRI head and neck. For each subject, the same imaging modality should be used at Screening and Visit 5 for consistency.
- Perform clinical labs: blood draw for complete blood count (CBC), chemistry panel (creatinine for determining creatinine clearance, blood urea nitrogen (BUN), liver function test (LFT), electrolytes [potassium, magnesium, calcium, and sodium], hCG [serum]) for females of childbearing potential and perform urinalysis.
- AE documentation begins after subject signs the ICF
- Schedule study visits
- Obtain historical or test tissue for HPV

9.6.2 Baseline, Treatment (Visit 1)

Occurs within 28 days following Screening visit (Visit 0).**Predose Assessments (Baseline):**

- Verify inclusion/exclusion criteria
- Perform and record result of physical examination and VS
- Perform and record visual oral cavity examination and neck lymph node examination
- Perform and document baseline clinical tumor measurement
- Collect clinical labs: blood draw for CBC, chemistry panel (creatinine for determining creatinine clearance, BUN, LFT, electrolytes [potassium, magnesium, calcium, and sodium], hCG [urine] for females of childbearing potential), and perform urinalysis.
- Administer oral anti-emetics (ondansetron or promethazine, the dose to be determined at the discretion of the PI) before the start of PRV111 treatment
- Photograph the tumor
- Draw blood for PK (-15 to 0 minutes predose)
- Administer PE (refer to Pharmacy Manual for administration instructions)
    - document time of application
    - ask for taste of PE solution
- Administer PRV111 patch(es) and document time of each application and removal. Ask for taste of patch.

Postdose Assessments:

- Draw blood for PK 30±5 minutes, 60±5 minutes and 120 ±5 minutes following application of the last patch at that treatment visit.
- VS shall be monitored at 30±5 minutes, 60±5 minutes and 120 ±5 minutes following application of the last patch applied.
- Collect all used patches (include the ones that detached prematurely), record the time of the detachment of each patch, store the patches in the plastic bags supplied by the Sponsor (refer to Laboratory Manual for instructions).
- Record AEs as reported by subject or observed by Investigator
- Review any concomitant therapy
- Schedule study visits

9.6.3 *Treatment (Visit 2)*

Occurs at least 48 hours, up to 144 hours, after previous visit.

Predose Assessments:

- Perform and document physical examination and VS
- Perform and document visual oral examination and neck lymph node examination
- Perform and document clinical tumor measurement and Tumor volume CFB criteria
- Administer oral anti-emetics (ondansetron or promethazine, the dose to be determined at the discretion of the PI) before the start of PRV111 treatment
- Photograph the tumor
- Draw blood for PK (-15 to 0 minutes predose)
- Administer PE (refer to Pharmacy Manual for administration instructions)
  - document time of application
- Administer PRV111 patch(es) and document time of each application and removal
  - Document adherence of each patch

Postdose Assessments:

- Draw blood for PK 30±5 minutes following application of the last patch applied at that treatment visit. VS shall be monitored at 30±5 minutes following application of the last patch at that treatment visit.
- Collect all used patches (include the ones that detached prematurely), record the time of the detachment of each patch, store the patches in the plastic bags supplied by the Sponsor (refer to Laboratory Manual for instructions)
- Record AEs as reported by subject or observed by Investigator
- Review any concomitant therapy
- Schedule study visits

9.6.4 *Treatment (Visit 3)*

Occurs at least 48 hours , up to 144 hours, after previous visit.

Predose Assessments:

- Perform and document physical examination and VS
- Perform and document visual oral examination and neck lymph node examination
- Perform and document clinical tumor measurement and Tumor volume change from baseline
- Collect clinical labs: blood draw for CBC, chemistry panel (creatinine for determining creatinine clearance, BUN, LFT, electrolytes [potassium, magnesium, calcium, and sodium], and perform urinalysis.
- Administer oral anti-emetics (ondansetron or promethazine, the dose to be determined at the discretion of the PI) before the start of PRV111 treatment
- Photograph the tumor

- Draw blood for PK (-15 to 0 minutes predose)
- Administer PE (refer to Pharmacy Manual for administration instructions)
    - Document time of application
- Administer PRV111 patch(es) and document time of each application and removal
    - Document adherence of each patch

Postdose Assessments:

- Draw blood for PK 30±5 minutes
- VS shall be monitored for 30±5 minutes following application of the last patch applied at that treatment visit
- Collect all used patches (include the ones that detached prematurely), record the time of the detachment of each patch, store the patches in the plastic bags supplied by the Sponsor (refer to Laboratory Manual for instructions).
- Record AEs as reported by subject or observed by Investigator
- Review any concomitant therapy
- Schedule study visits for subjects

9.6.5 Treatment (Visit 4)

Occurs at least 48 hours, up to 144 hours, after previous visit.

Predose Assessments:

- Perform and document physical examination and VS
- hCG [urine] for females of childbearing potential
- Perform and document clinical tumor measurement and tumor volume change from baseline criteria
- Perform and document visual oral examination and neck lymph node examination
- Administer oral anti-emetics (ondansetron or promethazine, the dose to be determined at the discretion of the PI) before the start of PRV111 treatment
- Photograph the tumor
- Draw blood for PK (-15 to 0 minutes predose)
- Administer PE (refer to Pharmacy Manual for administration instructions)
    - document time of application
- Administer PRV111 patch(es) and document time of each application and removal
    - Document adherence of each patch

Postdose Assessments:

- Draw blood for PK 30±5 minutes, 60±5 minutes and 120±5 minutes
- VS shall be monitored at 30±5 minutes, 60±5 minutes and 120±5 minutes following application of the last patch applied.

- Collect all used patches (include the ones that detached prematurely), record the time of the detachment of each patch, store the patches in the plastic bags supplied by the Sponsor (refer to Laboratory Manual for instructions).
- Record AEs as reported by subject or observed by Investigator
- Review any concomitant therapy
- Schedule study visits

9.6.6 *Post-Treatment/Pre-Op Visit (Visit 5)*

- Occurs a minimum of 72 hours after the last visit and prior to surgery. Perform and document physical examination and VS
- Perform and document visual oral examination and neck lymph node examination
- Perform CT or MRI of the head and neck. For each subject, the same imaging modality should be used at Screening and Visit 5 for consistency.
- Perform and document clinical tumor measurement and Tumor volume CFB criteria
- ECG
- Collect clinical labs: blood draw for CBC, chemistry panel (creatinine, BUN, LFT, electrolytes [potassium, magnesium, calcium, and sodium], and perform urinalysis
- Photograph the tumor
- Record AEs as reported by subject or observed by Investigator
- Review any concomitant therapy

9.6.7 *Surgery Day (Visit 6)*

- Tumor resection as per the standard of care
- Obtain tissue samples (Laboratory Manual):
  - Hematoxylin & Eosin staining for histology evaluation
  - Platinum level in the tumor and lymph nodes (if available)
- Post-Surgery, collect clinical labs. Draw blood for CBC, chemistry panel (creatinine, BUN, LFT, electrolytes [potassium, magnesium, calcium, and sodium], and perform urinalysis. Post-surgical labs may be used, if deemed appropriate by the Principal Investigator

9.6.8 *Post-Treatment Follow-Up (Visits 7, 8, & 9)*

All subjects will be followed for up to 6 months post-surgery at 3 visits: 1, 3, and 6 months post-surgery for loco-regional response and safety. Follow-up visits may coincide with standard of care follow-up visits, if deemed appropriate by the Principal Investigator.

- Perform and record results of physical examination and VS
- Perform visual oral examination and neck lymph node examination to assess for signs of loco-regional recurrence
- Review any concomitant therapy

10 STATISTICAL METHODS AND PLANNED ANALYSES

10.1 General Considerations

Clinical response to treatment will be evaluated according to Table 4.

10.2 Sample Size Determination

Safety outcomes resulting in DLT will be used during the conduct of this study to select doses being tested according to pre-defined thresholds of toxicity.

- Local and systemic safety and tolerability will be summarized using descriptive statistics.
- The number of subjects with DLTs will be capped to be fewer than 2 subjects with DLTs out of 5 new subjects enrolled.
- The treatment regimen will be deemed tolerable if fewer than a third of the subjects exposed to it present DLTs.
- Efficacy based on Tumor volume CFB criteria will be summarized, and the null hypothesis that the probability of response is at most 30%, where a response is the occurrence of CR or PR will be tested using a one-sided test exact binomial test at level 0.018.
- Other efficacy assessments such as change in tumor volume or tumor histology will be summarized using descriptive statistics.
- Variables measuring technical success (including residual cisplatin level in the patch) will be summarized using descriptive statistics.

At the end of Stage 2, the following is a statistical analysis of the sample size as it pertains to efficacy:

Power and Sample Size

The planned enrollment for the final dose is intended to obtain N=11 evaluable subjects.

Based on Simon's 2-stage procedure, the null hypothesis that the true response rate is 0.30 will be tested against a 1-sided alternative. In the first stage, 5 evaluable subjects will be accrued. If there are 2 or fewer responses in these 5 subjects, the dose will be escalated. Otherwise, the dose will remain the same or de-escalated based on safety data. Additional subjects will be accrued for a total of up to 11 evaluable subjects at the final dose. The null hypothesis will be rejected if **7** or more responses are observed at the final dose. This design yields a type I error rate of 0.018 and power of 96.44% when the true response rate is 0.85.

Null response rate: P_0	Alt. response rate: P_1	Alpha one-tailed	Power	n1	r1	N	R
0.30	0.85	0.018	96.44%	5	2	11	6

N is the total number of evaluable subjects at the final dose

$\boldsymbol{n_1}$ is the number of evaluable subjects accrued during Stage 1

$\boldsymbol{r_1}$, if r_1 or fewer responses are observed during stage 1, escalate the dose if safety requirements are met

R, if *R* or fewer responses are observed by the end of Stage 2 in N subjects, then an effective dose is not found

Note: If the dose is not increased after Stage 1, the test will be performed using all evaluable subjects from Stage 1 and 2. If the dose is adjusted after Stage 1, the test will be performed using all evaluable subjects from Stage 2.

10.3 Pharmacokinetics

PK parameters will include AUC, C_{max}, C_{min}, T_{max}, and $t_{1/2}$. Systemic and tumor and lymph nodes (if available) platinum levels will be summarized using descriptive statistics.

10.4 Study Populations

10.4.1 Safety Population

All subjects who have at least 1 PRV111 patch applied will be included in the Safety Population.

10.4.2 Evaluable Population

All subjects who have met the inclusion and exclusion criteria, and have completed at least 75% of the total dose required for that stage will be included in the Evaluable Population. This population will be used for the efficacy analysis.

10.5 Statistical Analyses

10.5.1 Analysis Timepoints

Data from this study will be documented at 2 timepoints:

- Final report at End of Stage 2: after all subjects complete PRV111treatment, efficacy, PK, and technical data will be analyzed, including all available safety data from the long-term follow-up.
- Long term safety report: after all subjects complete all observations.

10.5.2 Subject Accountability, Demographics, and Baseline Characteristics

Subject completion status, dose modification, and study terminations will be summarized for each of the study stages and for the study overall. Subjects with major protocol violations will be listed. Demographics will be summarized along with baseline characteristics such as medical history, and baseline physical examination, weight, VS, ECOG PS, and concomitant therapy.

10.5.3 Primary Endpoints

The primary endpoints for the study involve determination of a safe and efficacious dose of PRV111 (Cisplatin Transmucosal System) (cisplatin drug-loaded patch + PE) under the conditions of the trial.

Therefore, the primary endpoint is based on the incidence of DLTs and tumor volume CFB.

10.5.4 Secondary Endpoints

Based on secondary objectives, the secondary endpoints are:

- Evaluate tumor response
  - CT or MRI of the head and neck based on RECIST 1.1
  - Histology at the time of surgical excision
- Assess short and long-term safety of the treatment
  - Loco-regional control: subjects will be followed-up for disease recurrence and safety following surgery
  - Incidence of recurrence at 6 months
- Total platinum assessment in blood, tumor and lymph nodes (if available)
- Technical success:
  - Successful adhesion of the patch to the target lesion
  - Successful delivery (including release and permeation) of the drug to the target lesion, without getting to surrounding oral surfaces
  - Successful peel of the used patch after 10 minutes without damaging the target lesion underneath
  - Adhesion of the backing to the polymeric matrix embedded CLPs
  - Taste of the PE solution
  - Taste of the PRV111 patch
  - Residual cisplatin level in the patch after the application period

10.5.5 Efficacy Data

Efficacy outcomes include:

- Response to treatment evaluated by Tumor volume change from baseline (CFB)
- CT Scan/MRI RECIST 1.1
- Incidence of recurrence at 6 months
- Histological effect of PRV111 on the tumor tissue

10.5.6 Safety Data

Safety observations and measurements including study drug exposure, AEs, laboratory data, physical examination findings, VS, weight, and concomitant therapy will be summarized and presented in tables and listings.

Laboratory parameters will be summarized using descriptive statistics and data listings. The incidence of clinically significant abnormalities will be summarized. Physical examinations, VS, and weight will be summarized by changes from baseline values using descriptive statistics.

AEs occurring after the start of treatment are TEAEs. The severity of the TEAEs will be graded according to the CTCAE criteria, version 4.03. The severity of oral mucositis will be graded according to the WHO grade (Section 8.2).

The number and percentages of subjects experiencing TEAEs will be tabulated by system organ class and preferred term. Assessments of TEAEs will include characterization of the type, incidence, severity, seriousness, and relationship to treatment. Summary subject listings will be

provided for subjects with dose modification, SAEs, TEAEs resulting in study discontinuation, and deaths. The rates of local tolerability and oral mucositis will be reported.

10.5.7 Pharmacokinetics Data

PK parameters will include AUC, C_{max}, C_{min}, T_{max}, and $t_{1/2}$.

10.5.8 Technical Data

Technical success is defined as:

- Successful adhesion of the patch to the target lesion.
- Successful delivery (including release and permeation) of the drug to the target lesion, without getting to surrounding oral surfaces.
- Successful peel of the used patch after 10 minutes without damaging the target lesion underneath.

11 QUALITY CONTROL AND QUALITY ASSURANCE

Quality Assurance (QA) and Quality Control systems will be implemented and maintained with Standard Operating Procedures by Privo Technologies, as appropriate, to ensure that this clinical study is conducted and data are generated, documented and reported in compliance with the Protocol, ICH E6 GCP and applicable regulatory requirements.

Before enrolling any subjects in this study, a Sponsor representative and the Investigator will review the Protocol, IB, CRFs and instructions for their completion, as well as the procedures for obtaining informed consent and for reporting TEAEs and SAEs. A qualified representative of the Sponsor will monitor the conduct of the study by visiting the site and by contacting the site by telephone and email. During site visits, the study Monitor will assure accurate and reliable data collection by verifying the information recorded on the CRFs against source documents and medical records (ie, source document verification). Measures will be undertaken to protect the confidentiality of records that could identify subjects respecting the privacy and confidentiality rules in accordance with applicable regulatory requirements.

12 DOCUMENTATION AND INSPECTIONS

12.1 Study Monitoring

The Sponsor has responsibility to governing regulatory authorities to take all reasonable steps to ensure the proper conduct of the study with respect to trial ethics, Protocol adherence, and data integrity and validity.This trial will be closely monitored by Sponsor representatives throughout its duration. Monitoring will be in the form of personal visits with the Investigator and staff as well as any appropriate communications by telephone, mail, or email transmission.

The personal visits and monitoring are conducted to the extent possible however under certain circumstances such as COVID-19 pandemic, to minimize or eliminate immediate hazards or to protect the life and well-being of research staff and participants, discussions, monitoring and site

closures will be performed remotely via technologies including but not limited to phone calls, email, eCRF, eTMF and file sharing on video conferencing for source documents. Sponsor and sites can utilize video and teleconferencing such as ZOOM to remotely discuss, review and approve documents. The PI sign off can be obtained and saved electronically for all eCRFs at the site.

The purpose of these contacts is to review trial progress, Investigator and subject adherence to the protocol, and to determine if there are any problems associated with the conduct of the study. The following may be assessed during site monitoring visits:

- Required regulatory documentation
- Signed ICFs
- Subject accrual and follow-up
- Study drug inventory records
- Investigator and subject compliance to the study Protocol
- Concomitant therapy uses
- AE documentation
- Protocol deviation documentation
- Data are accurate, complete, and verifiable when compared to source documents

The Investigator and study staff are expected to cooperate with monitors during such visits and provide all relevant study documents.

12.2 Audits and Inspections

All documentation pertaining to this clinical trial may be subject to a QA audit by the Sponsor, or the FDA. Upon request, the auditor will have access to inspect, copy, review, and audit all source documents, CRFs, medical records, correspondence, and ICFs pertaining to the subjects in the trial. The Investigator agrees to promptly take any reasonable steps that are requested by the Sponsor as a result of an audit to cure deficiencies in the study documents and CRFs. Other documentation subject to QA audit includes the IRB files, certification and quality control of supporting laboratories, and records relevant to the trial in any supporting pharmacy facilities. Conditions of storage of study materials are also subject to inspection. Sponsor representatives may observe the conduct of any aspect of the clinical trial or its supporting activities both within and outside of the Investigator's institution.

13 ETHICAL AND LEGAL ISSUES

13.1 Ethical Conduct/Good Clinical Practice

This study will be conducted in accordance with the ethical principles that have their origin in the current Declaration of Helsinki and will be consistent with ICH GCP and applicable regulatory requirements.

13.2 Institutional Review Board

This study must be reviewed and approved by the IRB representing the institution prior to enrolling subjects. The IRB must be appropriately constituted and meet all requirements as described in Part 56, Title 21 of the US CFR. The review must include both the Protocol and the ICF for the trial. A copy of the Letter or Notice of Approval from the IRB must be received by the Sponsor prior to shipment of drug supplies to the Investigator. The IRB membership list must be submitted to the Sponsor with the written IRB approval and updated lists, if applicable.

The Investigator must promptly report all changes in the research activity and all unanticipated problems involving risk to the subjects or others to their IRB. The Investigator will provide progress reports as required by the IRB. The Investigator is responsible for assuring continuing review and approval of the clinical study on an annual basis and submitting documentation of renewal to the Sponsor. The Investigator will give notice to the IRB when participation in the study has been completed.

13.3 Written Informed Consent

The Investigator agrees to protect the rights, safety, and welfare of the subjects entered into the study, including obtaining written informed consent prior to performing any study-related procedures and informing each subject that the study drug is being used for investigational purposes. A copy of the IRB-approved ICF to be used during the study must be submitted for Sponsor review prior to study initiation.

Prior to entry into the study, the purpose and nature of the study and possible AEs must be explained to each patient. All questions about the study should be answered to the patient’s satisfaction or the patient’s legal representative. It is the responsibility of the Investigator or designee to obtain written informed consent from each patient, thereby attesting that consent was freely given. A copy of the signed and dated ICF will be given to the patient. Documentation of the informed consent process must be evident in the patient’s clinical files, and the original executed ICF must be available for review by the study Monitor.

13.3.1 Update of Informed Consent

In the event that modifications in the experimental design, dosages, parameters, subject selection, etc., of the Protocol are indicated or required, and in the event that such modifications substantially alter the study design or increase the potential risk to subjects, revisions to the existing ICF will be made. Such a revision will be reviewed and approved by the appropriate IRB, and documentation of this approval will be forwarded to the Sponsor for submission to the appropriate regulatory body.

In addition, all current study participants, as well as subsequent study candidates, will be informed of the study design modification or increase in potential risk, and written informed consent will be obtained as outlined in Section 13.3.

13.4 Records and Case Report Forms

The Investigator is required to prepare and maintain adequate and accurate case histories designed to record all observations and other data pertinent to the investigation on each individual treated with the study drug. Data reported on the CRF that are derived from source documents must be consistent with the source documents or the discrepancies must be explained. All requested information must be entered on the CRF in the spaces provided.

The confidentiality of records that could identify subjects must be protected, respecting the privacy and confidentiality rules in accordance with the applicable regulatory requirements.

The completed CRF must be promptly reviewed, signed, and dated by a qualified physician who is an Investigator or Sub-Investigator. The Investigator must retain a copy of the CRFs including records of the changes and corrections.

13.5 Protocol Deviations

The study is to be conducted as described in this Protocol without deviation unless there is a safety concern. The Protocol should not be modified for any subject without the prior consent of the Sponsor and, if necessary, the IRB responsible at the investigative site.

In the event of an important deviation, a report will be submitted to the IRB (as applicable) and site explaining the circumstances.

13.6 Retention of Records

US federal law requires that the Investigator retain copies of all files pertaining to the trial (ie, medical records, laboratory reports, drug inventory/disposition records, signed ICFs, CRFs, all correspondence, dates and reports of monitoring visits) for a period of 2 years following the date of marketing application approval of the drug for the indication investigated in the study, and until there is no pending or contemplated marketing application, or for 2 years following the Sponsor’s discontinuing worldwide clinical development of the study drug, as notified by the Sponsor, whichever is longer.

If the Investigator relocates, retires, or withdraws for any reason from the study, trial records may be transferred to an acceptable designee, such as another Investigator within the institution. Prior notice of such transfer will be provided in writing to the Sponsor. The Investigator must obtain written permission from the Sponsor prior to disposing of any records.

14 REFERENCES

Brockstein B, Stenson K, Song S. Overview of Treatment for Head and Neck. Posner M, Fried M, Brizel D, Ross M, editors. 2016. UpToDate.

da Silva SD, Hier M, Mlynarek A, Kowalski LP, Alaoui-Jamali MA. Recurrent oral cancer: current and emerging therapeutic approaches. Front Pharmacol 2012; 3:149.

Galluzzi L, Vitale I, Michels J, Brenner C, Szabadkai G, Harel-Bellan A et al. Systems biology of cisplatin resistance: past, present and future. Cell Death Dis 2014; 5:e1257.

Gregoire V, Lefebvre JL, Licitra L, Felip E. Squamous cell carcinoma of the head and neck: EHNS-ESMO-ESTRO Clinical Practice Guidelines for diagnosis, treatment and follow-up. Ann Oncol 2010; 21 Suppl 5:v184-v186.

Gurudutt VV, Genden EM. Cutaneous squamous cell carcinoma of the head and neck. J Skin Cancer 2011; 2011:502723.

Hong M, Puaux AL, Huang C, Loumagne L, Tow C, Mackay C et al. Chemotherapy induces intratumoral expression of chemokines in cutaneous melanoma, favoring T-cell infiltration and tumor control. Cancer Res 2011; 71(22):6997-7009.

Johnstone TC, Suntharalingam K, Lippard SJ. Third row transition metals for the treatment of cancer. Philos Trans A Math Phys Eng Sci 2015; 373(2037).

Kelner N, Vartanian JG, Pinto CA, Coutinho-Camillo CM, Kowalski LP. Does elective neck dissection in T1/T2 carcinoma of the oral tongue and floor of the mouth influence recurrence and survival rates? Br J Oral Maxillofac Surg 2014; 52(7):590-597.

Marta GN, William WN, Jr, Feher O, Carvalho AL, Kowalski LP. Induction chemotherapy for oral cavity cancer subjects: Current status and future perspectives. Oral Oncol 2015; 51(12):1069-1075.

Milano M, Chmura S, Haraf D, Banuchi V, Kraus D. Treatment of Locally Recurrent Squamous Cell Carcinoma of the Head and Neck. Brizel DFMRM, editor. 2015. UptoDate.

National Cancer Institute. Cancer Staging. March 2015. Accessed 3 August 2017 https://www.cancer.gov/about-cancer/diagnosis-staging/staging.

National Cancer Institute. SEER Cancer Statistics Fact Sheet: Oral Cavivity and Pharynx Cancer. NIH, editor. 2016. 8-24-2016.

National Institute of Dental and Craniofacial Research. Oral Cancer 5-year Survival Rates by Race, Gender, and Stage of Diagnosis. 2014. 8-24-2016.

National Institute of Dental and Craniofacial Research. Oral Cancer Incidence (New Cases) by Age, Race, and Gender. 2016. NIH. 9-15-2016.

Seiwert TY, Salama JK, Vokes EE. The chemoradiation paradigm in head and neck cancer. Nat Clin Pract Oncol 2007; 4(3):156-171.

US Department of Health and Human Services. "National Cancer Institute. Common Terminology Criteria for Adverse Events (CTCAE). Version 4.0. 2009 (v4. 03: June 14, 2010)."

Vermorken JB, Remenar E, van HC, Gorlia T, Mesia R, Degardin M et al. Cisplatin, fluorouracil, and docetaxel in unresectable head and neck cancer. N Engl J Med 2007; 357(17):1695-1704.

Vermorken JB, Specenier P. Optimal treatment for recurrent/metastatic head and neck cancer. Ann Oncol 2010; 21 Suppl 7:vii252-vii261.

WG Critical Care, LLC. Cisplatin Injection (NDA 018057), product labeling. 2015. Paramus, NJ, WG Critical Care, LLC.

15 APPENDIX 1: AMENDMENT 1

The purpose of Amendment 1, dated 19 October 2017, was to respond to FDA comments of 25 September 2017. The requested revisions included:

Changes to the 3 August 2017 Protocol

New text presented in **Bold** typeface; deleted text presented with a ~~Strikethrough~~

Section	Description of Change
**General Revisions**	Version number changed from ~~1.0~~ to **2.0**
	Phase changed from Phase 2 to Phase **1**/2
	Row added: Date of Amendment1 19 October 2017
**Synopsis: Methodology and Section 6.1 Overall Trial Design and Plan: Description (2nd paragraph, 1st sentence)**	***Formerly read:***<br>***Study Design:*** The study will employ an adaptive design with up to 20 subjects enrolled for the purpose of 15 evaluable subjects for the primary analysis.<br><br>***Now reads:***<br>*Study Design:* **Twenty-one** ~~The study will employ an adaptive design with up to 20~~ subjects **will be** enrolled for the purpose of 15 evaluable subjects for the primary analysis. **All subjects will undergo evaluation by a Multidisciplinary Team including a Medical Oncologist and a Radiation Oncologist with expertise in Head and Neck cancer**.
**Synopsis: Methodology and Section 6.1 Overall Trial Design and Plan: Description (3rd paragraph, 1st sentence)**	***Formerly read:***<br>*Stage 1:* Up to 5 subjects will be enrolled and undergo 3 treatment applications at each of the 4 planned treatment visits.<br><br>***Now reads:***<br>*Stage 1:* **Six** ~~Up to 5~~ subjects will be enrolled and undergo 3 treatment applications at each of the 4 planned treatment visits.
**Synopsis: Methodology and Section 6.1 Overall Trial Design and Plan: Description (4th paragraph, 1st sentence)**	***Formerly read:***<br>These 5 subjects will be monitored for local and systemic safety, tumor volume shrinkage due to the treatment, and systemic drug exposure. At the conclusion of Stage 1, the Principal Investigators (PI) and Medical Monitor (reviewers) will review all available data regarding systemic safety, local tolerability, and systemic exposure.<br><br>***Now reads:***<br>These **6** ~~5~~ subjects will be monitored for local and systemic safety, tumor volume shrinkage due to the treatment, and systemic drug exposure. At the conclusion **of the treatment phase** of Stage 1, the Principal Investigators (PI) and Medical Monitor (reviewers) will review all available data regarding systemic safety, local tolerability, and systemic exposure.
**Synopsis: Methodology and Section 6.1 Overall Trial Design and Plan: Description**	***Formerly read:***<br>• If at the end of Stage 1, at least 3 subjects out of 5 show no response (RECIST criteria stable disease [SD] or progressive disease [PD]),

Section	Description of Change
**(4th paragraph - bullet list)**	the dose will be increased to 5 treatment applications per treatment visit for Stage 2.<br>• If at the end of Stage 1, at least 3 subjects out of 5 show a response (RECIST criteria complete response [CR] or partial response [PR]), the dose for Stage 2 will be the same as for Stage 1.<br><br>***Now reads:***<br>• **If at the end of Stage 1, at least 4 out of 6 subjects have no response (RECIST criteria stable disease [SD] or progressive disease [PD]) AND <2 subjects have a dose-limiting toxicity (DLT), the dose will be increased to 5 treatment applications per treatment visit for Stage 2.**<br>• **If at the end of Stage 1, at least 3 out of 6 subjects have a response (RECIST criteria complete response [CR] or partial response [PR]), AND <2 patients have a DLT, the dose will remain the same for Stage 2.**<br>• **If at the end of Stage 1, at least 3 out of 6 subjects have a response AND ≥2 subjects have a DLT, the dose will be considered intolerable and will be de-escalated for Stage 2. De-escalation will consist of 2 treatment applications at each of the 4 planned treatment visits.**<br>• **If at the end of Stage 1, fewer than 3 out of 6 subjects have a response AND >2 subjects have a DLT, the dose will be considered intolerable and the study will be stopped.**<br>• ~~If at the end of Stage 1, at least 3 subjects out of 5~~ ~~**show no response**~~ ~~(RECIST criteria stable disease [SD] or progressive disease [PD]), the dose will be increased to 5 treatment applications per treatment visit for Stage 2.~~<br>• ~~If at the end of Stage 1, at least 3 subjects out of 5~~ ~~**show a response**~~ ~~(RECIST criteria complete response [CR] or partial response [PR]), the dose for Stage 2 will be the same as for Stage 1.~~
**Synopsis: Methodology and Section 6.1 Overall Trial Design and Plan: Description (5th paragraph, 1st sentence)**	***Formerly read:***<br>*Stage 2:* Up to an additional 15 subjects will be enrolled in Stage 2 for the purpose of 15 total evaluable in Stage 1 and 2 for the primary analysis. Subjects will receive either 3 treatment applications or 5 treatment applications at each of the 4 planned treatment visits, depending on the results of Stage 1.<br><br>***Now reads:***<br>**An interim safety report will be generated after all subjects in Stage 1 have completed all observations.**<br>*Stage 2:* **An** ~~Up to an~~ additional 15 subjects will be enrolled in Stage 2 for the purpose of 15 total evaluable in Stage 1 and 2 for the primary analysis. Subjects will receive either **2**, 3 ~~treatment applications~~, or 5 treatment applications at each of the 4 planned treatment visits, depending on the results of Stage 1.
**Synopsis: Methodology and Section 6.1 Overall Trial**	***Deleted:***

Section	Description of Change
**Design and Plan: Description (6th paragraph)**	~~The decision to increase the dose can be made as soon as the third non-responder is observed. In this case, the dose will be increased for all subsequently enrolled subjects, but ongoing subjects, if any, will continue on the dose for Stage 1. Up to 20 subjects will be enrolled for the purpose of 15 evaluable subjects for the primary analysis.~~
**Synopsis: Methodology 8th Paragraph**	***Added new last sentence:***<br>**The surgery is to be performed no later than 21 days post Visit 1.**
**Synopsis: Number of Subjects**	***Formerly read:***<br>Up to 20 subjects at up to 4 sites in the United States<br><br>***Now reads:***<br>**Twenty-one** ~~Up to 20~~ subjects **will be enrolled** at up to 4 sites in the United States **for the purpose of 15 evaluable subjects for the primary analysis.**
**Synopsis: Inclusion Criteria and Section 7.1 Inclusion Criteria**	***Formerly read:***<br>1. Adult subjects, men and women, defined by age ≥18 years at the time of screening.<br>2. Pathologically confirmed SCC of the oral cavity, and availability of tumor tissue block or slides from pathological confirmation, for histological assessment<br>3. Tumor must be amenable to surgical resection approximately 3 weeks after initiation of PRV111 System treatment.<br>4. Accessible tumors >T1 for local treatment (lip, tongue, gum, floor of mouth, salivary gland, and other locations in the oral cavity)<br>5. Clinically and/or radiologically measurable tumor.<br>6. Eastern Collaborative Oncology Group Performance Status of ≤3<br>7. Adequate renal function as demonstrated by serum creatinine ≤2 mg/dL or an institutional normal value<br>8. Candidates for standard of care treatment consisting of surgery<br>9. Male and female subjects of childbearing potential must agree to use appropriate birth control (barrier methods with spermicides, oral or parenteral contraceptives and/or intrauterine devices) during the entire duration of the study, or the subject must be naturally or surgically sterile (with documentation in the subject's medical records). Postmenopausal women are defined as presenting at least 12 months' natural spontaneous amenorrhea, or at least 6 weeks following surgical menopause (bilateral oophorectomy). Females of childbearing potential must be non-lactating and have a negative serum hCG within 14 days of treatment initiation<br>10. Absence of any serious underlying medical conditions which could impair the ability of the subject to participate in the study<br><br>***Now reads:***<br>1. Adult subjects, men and women, defined by age ≥18 years at the time of screening.<br>**2. Pathologically confirmed T1 (<2 cm) or T2 (>2 cm but ≤4 cm) SCC of the lip or oral cavity (anterior 2/3 of the tongue, floor of**

Section	Description of Change
	**mouth, lower and upper gingiva, salivary gland and hard palate).**<br>**3. Tumor must be easily accessible, with no evidence of infection or active bleeding, encroaching major vessels or clinical evidence of neural invasion. Tumors should not have been previously irradiated.**<br>~~4. Pathologically confirmed SCC of the oral cavity, and availability of tumor tissue block or slides from pathological confirmation, for histological assessment~~<br>4. Tumor must be amenable to surgical resection approximately 3 weeks after initiation of PRV111 System treatment.<br>~~5. Accessible tumors >T1 for local treatment (lip, tongue, gum, floor of mouth, salivary gland, and other locations in the oral cavity)~~<br>5. Clinically and/or radiologically measurable tumor.<br>6. Eastern Collaborative Oncology Group Performance Status of ≤**2**~~3~~.<br>7. Adequate renal function as demonstrated by serum creatinine ≤**1.5**~~2~~mg/dL **times the upper limit of normal (ULN),** or **creatinine clearance ≥50 mL/min (calculated using the Cockcroft-Gault formula)** ~~an institutional normal value~~.<br>**8. Adequate organ function defined as: ANC ≥1000/µL, platelet ≥100,000/µL; Hb ≥9.0 g/dL; AST and ALT ≤2.5 times the ULN, serum bilirubin ≤1.5 times the ULN or direct bilirubin ≤ULN for subjects with total bilirubin >1.5 times the ULN.**<br>9. Candidates for standard of care treatment consisting of surgery.<br>10. Male and female subjects of childbearing potential must **agree to use agree to use 2 methods of effective contraception from screening during the entire duration of the study and for at least 30 days after the final dose of investigational product. A**ppropriate birth control **is defined as** ~~(~~barrier methods with spermicides, oral or parenteral contraceptives and/or intrauterine devices~~) during the entire duration of the study~~, or ~~the subject must be~~ naturally or surgically sterile (with documentation in the subject's medical records). Postmenopausal women are defined as presenting at least 12 months' natural spontaneous amenorrhea, or at least 6 weeks following surgical menopause (bilateral oophorectomy). Females of childbearing potential must be non-lactating and have a negative serum hCG within 14 days of treatment initiation<br>11. Absence of any serious underlying medical conditions which could impair the ability of the subject to participate in the study.<br>**12. Have a life expectancy of ≥3 months.**<br>**13. Willing and able to provide written informed consent.**<br>14. **Able to return to study site for treatment and follow-up visits as defined in the protocol.**
**Synopsis: Exclusion Criteria and Section 7.2 Exclusion Criteria**	***Formerly read:***<br>2. Prior radiation and/or systemic chemotherapy for the treatment of SCC of the head and neck less than 2 years prior to screening<br>3. Concurrent malignancy, with the exception of localized squamous cell carcinomas of the skin

Section	Description of Change
	Now reads:<br>2. ~~Prior radiation and/or s~~Systemic chemotherapy for the treatment of SCC of the head and neck less than 2 years prior to screening<br>3. Concurrent **documented** malignancy, with the exception of localized squamous cell carcinomas of the skin
**Synopsis: Test Product, Dose and Mode of Administration (2nd bullet)**	***Formerly read:***<br>• Patch with mucoadhesive polymeric matrix, embedded chitosan-loaded particles (CLPs), and a non-permeable backing. Each 2 cm x 2 cm PRV111 topical patch covers a tumor region of 4 cm^2 and contains 0.5 mg/cm^2 (total 2 mg) of cisplatin.<br><br>***Now reads:***<br>• Patch with mucoadhesive polymeric matrix, embedded **cisplatin** ~~chitosan~~ -loaded **chitosan** particles (CLPs), and a non-permeable backing. Each 2 cm x 2 cm PRV111 topical patch covers a tumor region of 4 cm^2 and contains 0.5 mg/cm^2 (total 2 mg) of cisplatin.
**Synopsis: Criteria for Evaluation (2nd bullet list)**	***Added new third bullet:***<br>• Computed tomography or magnetic resonance imaging of the head and neck
**Synopsis: Statistical Methods**	***Formerly read:***<br>The primary analysis will be conducted after all subjects have completed their post treatment visit (Visit 5). All summaries will be presented by dose, as appropriate. Incidence of pre-surgery AEs will be summarized using number and percent for each System Organ Class and Preferred Term, including investigator assessment of relatedness and severity grade. Other safety assessments will be summarized using mean, standard deviation, median, minimum, and maximum for continuous variables and using number and percent for categorical assessments.<br>Tumor volume will be summarized using mean, standard deviation, median, minimum, maximum, and 95% confidence interval. RECIST clinical assessment (CR, PR, SD, or PD) will be summarized using number and percent. A 2-stage design will be used for Stage 1 to Stage 2 dosing decision and will be based on incidence of DLTs and on response using RECIST clinical assessment.<br>***Stage 1:*** Up to 5 subjects will be enrolled and undergo 3 treatment applications at each of the 4 planned treatment visits. Each treatment visit will include 1 application of the PE. Starting 5 minutes after application of the PE, 1 or 2 patches (depending on tumor size) will be applied. A total of 3 treatment applications of patches will occur. These successive applications will span approximately 35 minutes.<br><br>If ≥ 3 subjects among the first 5 subjects experience a response (RECIST criteria CR or PR), then subsequently enrolled subjects will receive 3 treatment applications at each of the 4 planned treatment visits.

Section	Description of Change
	If $\leq$ 2 subjects among the first 5 subjects experience a response, then subsequently enrolled subjects will receive 5 treatment applications at each of the 4 planned treatment visits.<br><br>***Stage 2:*** Up to an additional 15 subjects will be enrolled in Stage 2 receiving either 3 treatment applications or 5 treatment applications at each of the 4 planned treatment visits depending on the results of Stage 1.<br><br>***Now reads:***<br>**Data from this study will be assessed at 4 timepoints:**<br>• **Memo to file: after 6 subjects in Stage 1 finish PRV111treatment, response and DLTs will be assessed to determine Stage 2 dosing.**<br>• **Interim report: after 6 subjects in Stage 1 finish all observations up to 6 months, safety data will be analyzed.**<br>• **Interim report: after all subjects in Stage 2 finish PRV111treatment, efficacy, PK, and technical data will be analyzed. Subjects will continue to be followed for safety, so this report will have whatever safety data exist at this point in time.**<br>• **Final report: after all subjects complete all observations.**<br>All summaries will be presented by dose, as appropriate. Incidence of pre-surgery AEs will be summarized using number and percent for each System Organ Class and Preferred Term, including investigator assessment of relatedness and severity grade. Other safety assessments will be summarized using mean, standard deviation, median, minimum, and maximum for continuous variables and using number and percent for categorical assessments. Tumor volume will be summarized using mean, standard deviation, median, minimum, maximum, and 95% confidence interval. RECIST clinical assessment (CR, PR, SD, or PD) will be summarized using number and percent. A 2-stage design will be used for Stage 1 to Stage 2 dosing decision and will be based on incidence of DLTs and on response using RECIST clinical assessment.<br>~~***Stage 1:*** Up to 5 subjects will be enrolled and undergo 3 treatment applications at each of the 4 planned treatment visits. Each treatment visit will include 1 application of the PE. Starting 5 minutes after application of the PE, 1 or 2 patches (depending on tumor size) will be applied. A total of 3 treatment applications of patches will occur. These successive applications will span approximately 35 minutes.~~<br><br>~~If $\geq$ 3 subjects among the first 5 subjects experience a response (RECIST criteria CR or PR), then subsequently enrolled subjects will receive 3 treatment applications at each of the 4 planned treatment visits.~~<br><br>~~If $\leq$ 2 subjects among the first 5 subjects experience a response, then subsequently enrolled subjects will receive 5 treatment applications at each of the 4 planned treatment visits.~~

Section	Description of Change
	~~***Stage 2:*** Up to an additional 15 subjects will be enrolled in Stage 2 receiving either 3 treatment applications or 5 treatment applications at each of the 4 planned treatment visits depending on the results of Stage 1.~~
**Synopsis: Statistical Methods – Probability of Change in Treatment Regimen (1st paragraph)**	***Formerly read:***<br>The decision to increase the dose can be made as soon as the third non-responder is observed. In this case, the dose will be increased for all subsequently enrolled subjects, but ongoing subjects, if any, will continue on the dose for Stage 1. A total of 15 evaluable subjects will be enrolled. The probability of a change in treatment regimen is presented in the following table.<br><br>***Now reads:***<br>~~The decision to increase the dose can be made as soon as the third non-responder is observed. In this case, the dose will be increased for all subsequently enrolled subjects, but ongoing subjects, if any, will continue on the dose for Stage 1.~~ ~~A total of~~ 15 **of 21 enrolled subjects will be** evaluable ~~subjects will be enrolled.~~ The probability of a change in treatment regimen is presented in the following table.
**Synopsis: Statistical Methods – Power and Sample Size**	***Formerly read:***<br>At the end of Stage 2, the null hypothesis H0: π=0.3 will be tested against the 1-sided alternative H1: π>0.3 at 2.5% level. If the number of applications is not increased after Stage 1, the test will be performed using all 15 subjects. If the number of applications is increased after Stage 1, the test will be performed using all subjects from Stage 2. For 15 subjects, the power of the test is 81.1% if the true probability of success for the treatment regimen in Stage 1 is 0.65 and 92.2% if this success probability is 0.70. For 10 subjects, the power of the test is 78.8% if the true probability of success for the treatment regimen in Stage 2 is 0.70 and 90.8% if this success probability is 0.75.<br>***Now reads:***<br>At the end of Stage 2, the null hypothesis **for the anti-tumor effect of PRV111**, H0: π=0.3 will be tested against the 1-sided alternative H1: π>0.3 at 2.5% level. If the number of applications is not increased after Stage 1, the test will be performed using all ~~15~~ **evaluable** subjects. If the number of applications is increased after Stage 1, the test will be performed using all **evaluable** subjects from Stage 2. For 15 **evaluable** subjects, the power of the test is 81.1% if the true probability of success for the treatment regimen in Stage 1 is 0.65 and 92.2% if this success probability is 0.70. For 10 subjects, the power of the test is 78.8% if the true probability of success for the treatment regimen in Stage 2 is 0.70 and 90.8% if this success probability is 0.75.
**List of Abbreviations**	***Added:***<br>**CT Computed tomography**<br>**MRI Magnetic resonance imaging**<br>**ULN Upper limit of normal**
**Section 6.2 Discussion of Trial Design**	***Formerly read:***

Section	Description of Change
	This study is intended to establish the first safety observations in man and the feasibility of using 2 doses of the PRV111 (Cisplatin Transmucosal System) prior to the surgical removal of SCC tumors of the oral cavity.<br>An adaptive design with up to 20 subjects enrolled is used to have at least 15 evaluable subjects for the primary analysis. This adaptive design allows determination of an appropriate dose for further development while exposing as few subjects as possible to an ineffective low dose and yet having a sufficient number of subjects if the low dose is efficacious.<br>***Now reads:***<br>This study is intended to establish the first safety observations in man and the feasibility of using **3**~~2~~ doses of the PRV111 (Cisplatin Transmucosal System) prior to the surgical removal of SCC tumors of the oral cavity.<br>**Based on discussions with FDA, only subjects with T1 or T2 tumors are planned in this study. FDA's concerns are based on safety in a first-in-human study.**<br>~~An adaptive design with up to 20 subjects enrolled is used to have at least 15 evaluable subjects for the primary analysis. This adaptive design allows determination of an appropriate dose for further development while exposing as few subjects as possible to an ineffective low dose and yet having a sufficient number of subjects if the low dose is efficacious.~~
**Section 7.6 Premature Termination or Suspension of Study**<br>**(2nd paragraph – bullet list)**	***Added 3rd bullet:***<br>• **Excessive study treatment toxicity leading to delays in the scheduled surgery**
**Section 8.1.5 Dosing and Administration**<br>**(2nd paragraph)**	***Formerly read:***<br>In Stage 2, subjects will receive either 3 treatment applications or 5 treatment applications at each of the 4 planned treatment visits, depending on the results of Stage 1.<br><br>***Now reads:***<br>In Stage 2, subjects will receive either **2,** 3,~~ treatment applications~~ or 5 treatment applications at each of the 4 planned treatment visits, depending on the results of Stage 1.<br>***Inserted new text after 2nd paragraph:***<br>**Six subjects will be enrolled into Stage 1 to include the following criteria for decisions regarding dose escalation:**<br>• **If at the end of Stage 1, at least 4 out of 6 have no response AND fewer than 2 have a dose-limiting toxicity (DLT), the dose will be increased to 5 treatment applications per treatment visit for Stage 2.**<br>• **If at the end of Stage 1, at least 3 out of 6 subjects have a response AND ≥2 subjects have a DLT, the dose will be considered intolerable and will be de-escalated for Stage 2. De-escalation will consist of 2 treatment applications at each of the 4 planned treatment visits.**<br>• **If at the end of Stage 1, fewer than 3 out of 6 subjects have a response AND >2 subjects have a DLT, the dose will be considered intolerable and the study will be stopped.**

Section	Description of Change
**Section 8.1.5 Dosing and Administration *Table 2. Dosing Terminology:***	***Formerly read:***<br>Three or 5 patch applications will occur during each treatment visit in Stage 2.<br><br>***Now reads:***<br>T**wo**~~hree~~**, 3,** or 5 patch applications will occur during each treatment visit in Stage 2.
**Section 8.2 Dose-limiting Toxicity (Stopping Rules for an Individual Subject)**	***2nd Paragraph – bullet list (1st and 2nd bullets) formerly read:***<br>• Grade 3 or 4 non-hematologic toxicity excluding alopecia and sub-optimally treated nausea, vomiting or diarrhea.<br>• Grade 3 or 4 nausea, vomiting, or diarrhea, lasting >48 hours, despite standard prophylaxis and/or treatment<br>***Now reads:***<br>• Grade 3 or 4 non-hematologic toxicity excluding alopecia ~~and sub-optimally treated nausea, vomiting, or diarrhea~~ **and**<br>• Grade 3 or 4 nausea, vomiting, or diarrhea, lasting >48 hours, despite standard prophylaxis and/or treatment **(refer to Section 8.7.2 for a list of suggested antiemetic medications)**<br>***Added new last bullet:***<br>• **Any related AE leading to surgery delay for more than 96 hours is a DLT**
**Section 8.3 Dose Modification (1st paragraph – bullet list)**	***Deleted previous list:***<br>• ~~If at the end of Stage 1, at least 3 subjects out of **5 show no response** (RECIST criteria SD or PD), the dose will be increased to 5 treatment applications per treatment visit for Stage 2.~~<br>• ~~If at the end of Stage 1, at least 3 subjects out of 5 **show a response** (RECIST criteria CR or PR), the dose for Stage 2 will be the same as for Stage 1.~~
**Section 8.4 Dose Interruption**	***Formerly read:***<br>At the Investigator's discretion, study drug may be interrupted for no more than 96 hours for reasons not related to toxicity (eg, intercurrent illness, subject scheduling conflict) 1 time during the subject's treatment schedule.<br><br>***Now reads:***<br>At the Investigator's discretion, study drug may be interrupted ~~for no more than 96 hours~~ for reasons not related to toxicity (eg, intercurrent illness, subject scheduling conflict) ~~1 time during the subject's treatment schedule~~. **Such interruption may not last more than 96 hours and may not occur more than 1 time during the subject's treatment schedule.**<br>**Subjects who experience an AE(s) requiring interruption of study drug for ≥96 hours will be removed from treatment**
**Section 8.5 Study Drug Handling and Accountability**	***Formerly read:***<br>Ensure study drug supplies are stored between 2º and 8º C (PRV111 patches) and at 25ºC (PE) in an appropriately controlled limited-access room in a secure location.

Section	Description of Change
	Now reads:<br>Ensure study drug supplies are stored between 2º and 8º C (PRV111 ~~patches~~**study kits**) and at 25ºC (PE) in an appropriately controlled limited-access room in a secure location.
**Section 8.7 Concomitant Medications and Therapy (2nd paragraph, 1st sentence)**	***Formerly read:***<br>Concomitant medications and therapies are other prescription medications, over-the-counter drugs, or dietary supplements that a study participant takes in addition to the investigational product.<br>Symptomatic medications (eg, analgesics, anti-emetics) might be used during the study. Subjects need to consult with the Investigator or the primary care physician before taking any medication including over-the-counter drugs.<br>***Now reads:***<br>Concomitant medications and therapies are other prescription medications, over-the-counter drugs, or dietary supplements that a study participant takes in addition to the investigational product. **Based on FDA's recommendation, anti-emetic prophylaxis will be mandatory.**<br><br>Symptomatic **anti-emetics** ~~medications (eg, analgesics, anti-emetics) might~~ **will** be used during the study.<br><br>~~Subjects need to consult with the Investigator or the primary care physician before taking any medication including over-the-counter drugs.~~<br><br>**For emesis prophylaxis, subjects will receive ondansetron 8 mg orally approximately 30 minutes before administration of the PRV111treatment. After treatment, subjects may self-administer ondansetron 8 mg orally approximately every 12 hours for 24 to 48 hours for persistent nausea.**<br><br>**Alternatively, subjects may receive promethazine 12.5 mg orally 60 minutes before administration of the PRV111 treatment. After treatment, subjects may self-administer promethazine 12.5 mg approximately every 4 to 6 hours for 24 to 48 hours for persistent nausea.**<br><br>**Subjects with diarrhea may be supported clinically with loperamide, at dosing schedules recommended in the package insert.**<br><br>**Based upon the Investigator's judgment, the subject's tongue might need to be locally anesthetized to prevent accidental touching and removal of the patch.**
**Section 9 Efficacy and Safety Variables Assessed**	***Inserted new section after 9.2.3***<br>**9.2.4 Computed Tomography or Magnetic Resonance Imaging of the Head and Neck**<br>**A computed tomography (CT) scan or magnetic resonance imaging (MRI) of the head and neck will be performed.**
**Section 9.1.2 Tumor Staging**	***Deleted sentence:***<br>~~Eligible subjects will have tumors >T1.~~

Section	Description of Change
**Section 9.3.2 Oral Cavity Examination**	***Inserted new paragraph at the end of section:***<br>**Special attention must be given to the application site and the tumor, with documentation of any local adverse reactions, including documentation with photographs if warranted.**
**Section 9.3.4 Safety Laboratory Assessments**	***Formerly read:***<br>Clinical laboratories to be assessed will be Complete Blood Count (CBC) and chemistry panel (creatinine, blood urea nitrogen [BUN], liver function test [LFT]), electrolytes (potassium, magnesium, calcium, and sodium),coagulation, urinalysis.<br><br>***Now reads:***<br>Clinical laboratories to be assessed will be Complete Blood Count (CBC) and chemistry panel (creatinine, blood urea nitrogen [BUN], liver function test [LFT]), electrolytes (potassium, magnesium, calcium, and sodium), ~~coagulation,~~ urinalysis.<br>**Human papilloma virus will be ascertained using immunohistochemistry for p16 overexpression per institutional standards.**
**Section 9.6.1 Screening (Visit 0) – bullet list**	***Formerly read:***<br>• Obtain and document consent from potential subject on Informed Consent Form (ICF)<br>• Review inclusion/exclusion criteria to determine eligibility<br>• Review medical and medications history to determine eligibility based on inclusion/exclusion criteria<br>• Perform a general physical examination and check VS<br>• Perform a systematic and thorough visual and manual examination of the oral cavity mouth and cervical and local lymph nodes examination<br>• Perform an ECG<br>• Perform clinical labs: blood draw for CBC, chemistry panel (creatinine, BUN, LFT, electrolytes [potassium, magnesium, calcium, and sodium], coagulation, hCG [serum]) for females of childbearing potential, and perform urinalysis.<br>• AE documentation begins when the subject signs the ICF<br>• Schedule study visits<br><br>***Now reads:***<br>• Obtain and document consent from potential subject on Informed Consent Form (ICF)<br>• Review inclusion/exclusion criteria to determine eligibility<br>• Review medical and medications history to determine eligibility based on inclusion/exclusion criteria<br>• Perform a general physical examination and check VS<br>• **Perform CT of the chest (with or without contrast)**<br>• Perform a systematic and thorough visual and manual examination of the oral cavity mouth and cervical and local lymph nodes examination<br>• Perform an ECG<br>• **Perform CT or MRI head and neck**

Section	Description of Change
	• Perform clinical labs: blood draw for CBC, chemistry panel (creatinine, BUN, LFT, electrolytes [potassium, magnesium, calcium, and sodium], ~~coagulation,~~ hCG [serum]) for females of childbearing potential, and perform urinalysis.<br>• AE documentation begins when the subject signs the ICF<br>• Schedule study visits
**Section 9.6.2 Baseline, Treatment (Visit 1) (1st bullet list)**	***Formerly read:***<br>• Verify inclusion/exclusion criteria<br>• Perform and record result of physical examination and VS<br>• Perform and record visual oral cavity examination and neck lymph node examination<br>• Perform and document baseline clinical tumor measurement before the treatment<br>• Collect clinical labs: blood draw for CBC, chemistry panel (creatinine, BUN, LFT, electrolytes [potassium, magnesium, calcium, and sodium], coagulation, hCG [urine] for females of childbearing potential), and perform urinalysis<br>• Photograph the tumor<br>• Draw blood for PK time -15 to 0 minutes<br>• Administer PE and PRV111 patch(es)<br><br>***Now reads:***<br>• Verify inclusion/exclusion criteria<br>• Perform and record result of physical examination and VS<br>• Perform and record visual oral cavity examination and neck lymph node examination<br>• Perform and document baseline clinical tumor measurement before the treatment<br>• Collect clinical labs: blood draw for CBC, chemistry panel (creatinine, BUN, LFT, electrolytes [potassium, magnesium, calcium, and sodium], ~~coagulation,~~ hCG [urine] for females of childbearing potential), and perform urinalysis<br>• **Testing for the human papilloma virus**<br>• **Administer oral anti-emetics (ondansetron 8 mg approximately 30 minutes prior to the start of PRV111 treatment, or promethazine 12.5 mg approximately 60 minutes before the start of PRV111 treatment)**<br>• Photograph the tumor<br>• Draw blood for PK time -15 to 0 minutes<br>• Administer PE and PRV111 patch(es)
**Section 9.6.3 Treatment (Visit 2)<br>(Predose Assessments)**	***Inserted new 4th bullet:***<br>• **Administer oral anti-emetics (ondansetron 8 mg approximately 30 minutes prior to the start of PRV111 treatment, or promethazine 12.5 mg approximately 60 minutes before the start of PRV111 treatment)**
**Section 9.6.4 Treatment (Visit 3)**	***Formerly read:***<br>• Collect clinical labs: blood draw for CBC, chemistry panel (creatinine, BUN, LFT, electrolytes [potassium, magnesium,

Section	Description of Change
**(Predose Assessments, 4th bullet)**	calcium, and sodium], coagulation, hCG [urine] for females of childbearing potential), and perform urinalysis<br><br>***Now reads:***<br>• Collect clinical labs: blood draw for CBC, chemistry panel (creatinine, BUN, LFT, electrolytes [potassium, magnesium, calcium, and sodium], ~~coagulation,~~ hCG [urine] for females of childbearing potential), and perform urinalysis
**Section 9.6.4 Treatment (Visit 3)**<br>**(Predose Assessments)**	***Inserted new 5th bullet:***<br>• **Administer oral anti-emetics (ondansetron 8 mg approximately 30 minutes prior to the start of PRV111 treatment, or promethazine 12.5 mg approximately 60 minutes before the start of PRV111 treatment)**
**Section 9.6.4 Treatment (Visit 3)**<br>**(Postdose Assessments)**<br>**Section 9.6.5 Treatment (Visit 4)**<br>**(Postdose Assessments)**	***3rd bullet formerly read:***<br>• Collect all used patches (include the ones that detached prematurely), record the time of the detachment of each patch, store the patches in the vial supplied by the Sponsor (Refer to Pharmacy Manual for instructions).<br><br>***Now reads:***<br>• Collect all used patches (include the ones that detached prematurely), record the time of the detachment of each patch, store the patches in the ~~vial~~ **plastic bags** supplied by the Sponsor (Refer to Pharmacy Manual for instructions).
**Section 9.6.5 Treatment (Visit 4)**<br>**(Predose Assessments)**	***Inserted new 4th bullet:***<br>• **Administer oral anti-emetics (ondansetron 8 mg approximately 30 minutes prior to the start of PRV111 treatment, or promethazine 12.5 mg approximately 60 minutes before the start of PRV111 treatment)**
**Section 9.6.6 Post-Treatment/Pre-Op Visit (Visit 5)**	***Formerly read:***<br>Occurs a minimum of 72 hours prior to surgery. The surgery is to be performed no later than 28 days post the screening visit.<br><br>***Now reads:***<br>Occurs a minimum of 72 hours **after the last visit and** prior to surgery. The surgery is to be performed no later than 2~~8~~**1** days post **Visit 1**~~the screening visit~~.<br><br>***Inserted new 3rd bullet:***<br>• **Perform CT of the head and neck**<br><br>***4th bullet formerly read:***<br>• Collect clinical labs: blood draw for CBC, chemistry panel (creatinine, BUN, LFT, electrolytes [potassium, magnesium, calcium, and sodium], coagulation, hCG [urine] for females of childbearing potential), and perform urinalysis

Section	Description of Change
	Now reads:<br>• Collect clinical labs: blood draw for CBC, chemistry panel (creatinine, BUN, LFT, electrolytes [potassium, magnesium, calcium, and sodium], ~~coagulation,~~ hCG [urine] for females of childbearing potential), and perform urinalysis
**Section 9.6.8 Post-Treatment Follow-Up (Visits 7, 8, & 9) (3rd bullet)**	***Formerly read:***<br>• Collect clinical labs: blood draw for CBC, chemistry panel (creatinine, BUN, LFT, electrolytes [potassium, magnesium, calcium, and sodium], coagulation, hCG [urine] for females of childbearing potential), and perform urinalysis<br><br>***Now reads:***<br>• Collect clinical labs: blood draw for CBC, chemistry panel (creatinine, BUN, LFT, electrolytes [potassium, magnesium, calcium, and sodium], ~~coagulation~~, hCG [urine] for females of childbearing potential), and perform urinalysis
**Section 10.2 Sample Size Determination**	***Formerly read:***<br>At the end of Stage 2, the null hypothesis H0: π=0.3 will be tested against the 1-sided alternative H1: π>0.3 at 2.5% level using the normal approximation to the binomial distribution. If the number of applications is not increased after Stage 1, the test will be performed using all 15 subjects. If the number of applications is increased after Stage 1, the test will be performed using all subjects from Stage 2. For 15 subjects, the power of the test is 81.1% if the true probability of success for the treatment regimen in Stage 1 is 0.65 and 92.2% if this success probability is 0.70. For 10 subjects, the minimum number of subjects for Stage 2, the power of the test is 78.8% if the true probability of success for the treatment regimen in Stage 2 is 0.70 and 90.8% if this success probability is 0.75.<br>***Now reads:***<br>At the end of Stage 2, the null hypothesis H0: π=0.3 will be tested against the 1-sided alternative H1: π>0.3 at 2.5% level using the normal approximation to the binomial distribution. If the number of applications is not increased after Stage 1, the test will be performed using all ~~15~~ **evaluable** subjects. If the number of applications is increased after Stage 1, the test will be performed using all **evaluable** subjects from Stage 2. For 15 **evaluable** subjects, the power of the test is 81.1% if the true probability of success for the treatment regimen in Stage 1 is 0.65 and 92.2% if this success probability is 0.70. For 10 **evaluable** subjects, ~~the minimum number of subjects for Stage 2,~~ the power of the test is 78.8% if the true probability of success for the treatment regimen in Stage 2 is 0.70 and 90.8% if this success probability is 0.75.
**Section 10.3.2 Efficacy Population**	***Formerly read:***<br>All subjects in the Safety Population with at least 1 tumor response according the RECIST Criteria will be included in the Efficacy Population.<br><br>***Now reads:***

Section	Description of Change
	All subjects in the Safety Population with at least 1 tumor response **assessment** according **to** the RECIST Criteria will be included in the Efficacy Population.
**Section 10.3.3 Evaluable Population**<br>**(1st sentence)**	***Formerly read:***<br>All subject in the Efficacy Population who complete at least 3 out of 4 PRV111 treatment cycles will be included in the Evaluable Population<br><br>***Now reads:***<br>All subject in the Efficacy Population who complete at least 3 out of 4 PRV111 treatment **visits** ~~cycles~~ will be included in the Evaluable Population
**Section 10.4 Statistical Analyses**	***Inserted new section 10.4.1:***<br><br>***10.4.1 Analysis Timepoints***<br>**Data from this study will be assessed at 4 timepoints:**<br>• **Memo to file: after 6 subjects in Stage 1 finish PRV111treatment, response and DLTs will be assessed to determine Stage 2 dosing.**<br>• **Interim report: after 6 subjects in Stage 1 finish all observations up to 6 months, safety data will be analyzed.**<br>• **Interim report: after all subjects in Stage 2 finish PRV111treatment, efficacy, PK, and technical data will be analyzed. Subjects will continue to be followed for safety, so this report will have whatever safety data exist at this point in time.**<br>• **Final report: after all subjects complete all observations.**
**Section 10.4 Statistical Analyses**	***Inserted new sections after 10.4.1:***<br>***10.4.2 Primary Endpoints***<br>**The primary objective is the determination of systemic safety and local tolerability. Therefore, the primary endpoint is incidence of adverse events.**<br><br>***10.4.3 Secondary Endpoints***<br>**Based on secondary objectives, the secondary endpoints are:**<br>• **Tumor response:**<br>o **RECIST clinical assessment (CR, PR, SD, or PD)**<br>o **Caliper measurements of tumor volume**<br>o **CT or MRI of the head and neck**<br>o **Histology at the time of surgical excision**<br>o **Loco-regional control: subjects will be followed-up for disease recurrence and safety following surgery**<br>• **Blood samples for plasma levels of total platinum**<br>• **Technical success:**<br>o **Successful adhesion of the patch to the target lesion**<br>o **Successful delivery (including release and permeation) of the drug to the target lesion, without getting to surrounding oral surfaces**<br>o **Successful peel of the used patch after 10 minutes without damaging the target lesion underneath**

Section	Description of Change
	○ **Adhesion of the backing to the polymeric matrix embedded CLPs**<br>○ **Taste of the PE solution**<br>○ **Taste of the PRV111 patch**<br>○ **Residual cisplatin level in the patch after the application period**
**Section 10.4.4 Efficacy Data (3rd bullet)**	***Formerly read:***<br>• Incidence of recurrence at 6 and 12 months<br><br>***Now reads:***<br>• Incidence of recurrence at 6 ~~and 12~~ months

Schedule of Events:

Formerly read:

	Screening Within 28 Days Prior to Visit 1	Days					Surgery	Months Post Surgery
		Treatment Visits				Post Treatment[g] Pre-Op		SOC Follow-up
		Visit 1	Visit 2	Visit 3	Visit 4	Visit 5	Visit 6	Visits 7, 8, 9
	0	1	At least 48 hours, up to 96 hours, after previous visit					1, 3, & 6±3 Weeks
Confirmed SCC	X							
Informed consent	X							
Inclusion/exclusion criteria	X	X[d]						
Schedule study visits	X	X	X	X	X	X		
Demographics, medical/ medication history	X							
Physical examination, VS[a]	X	X[d]	X[d]	X[d]	X[d]	X		X
Oral cavity examination[i]	X	X[d]	X[d]	X[d]	X[d]	X		X
Neck lymph nodes exam	X	X[d]	X[d]	X[d]	X[d]	X		X
Electrocardiogram	X				X[d]			
Clinical labs[b]	X	X[d]		X		X		X
Pregnancy test[c]	X	X[d]						
PK sampling		X[e]	X[f]	X[f]	X[e]			
Tumor resection							X[h]	
PRV111 Patch treatment		X	X	X	X			
Tumor measurement		X[d]	X	X	X[d]	X		
RECIST criteria			X[d]	X[d]	X[d]	X		
Tumor photographs		X[d]	X[d]	X[d]	X[d]	X		X
Loco-regional recurrence								X

		Days						Months Post Surgery
	Screening Within 28 Days Prior to Visit 1	Treatment Visits				Post Treatment[g] Pre-Op	Surgery	SOC Follow-up
		Visit 1	Visit 2	Visit 3	Visit 4	Visit 5	Visit 6	Visits 7, 8, 9
	0	1	At least 48 hours, up to 96 hours, after previous visit					1, 3, & 6±3 Weeks
Histology	X						X	
Technical success		X	X	X	X			
Taste of patch and PE solution		X	X	X	X			
Concomitant therapy	X	X	X	X	X	X		X
AE assessment	X	X	X	X	X	X		X

Now reads:

		Days						Months Post Surgery
	Screening Within 28 Days Prior to Visit 1	Treatment Visits				Post Treatment[g] Pre-Op	Surgery	SOC Follow-up
		Visit 1	Visit 2	Visit 3	Visit 4	Visit 5	Visit 6	Visits 7, 8, 9
	0	1	≥~~At least~~ 48 ~~hours, up to~~ and ≤96 hours, post previous visit			≥72 hours post last treatment	≤21 days post Visit 1	1, 3, & 6±3 Weeks
Confirmed SCC	X							
Informed consent	X							
Inclusion/exclusion criteria	X	X[d]						
Schedule study visits	X	X	X	X	X	X		
Demographics, medical/ medication history	X							
Physical examination, VS[a]	X	X[d]	X[d]	X[d]	X[d]	X		X
**Anti-emetic drugs**		**X[d]**	**X[d]**	**X[d]**	**X[d]**			

	Screening Within 28 Days Prior to Visit 1	Days						Months Post Surgery
		Treatment Visits				Post Treatment[g] Pre-Op	Surgery	SOC Follow-up
		Visit 1	Visit 2	Visit 3	Visit 4	Visit 5	Visit 6	Visits 7, 8, 9
	0	1	≥~~At least~~ 48 ~~hours,~~ ~~up to~~ and ≤96 hours, post previous visit			≥72 hours post last treatment	≤21 days post Visit 1	1, 3, & 6±3 Weeks
**CT chest**	**X**							
Oral cavity examination[i]	X	X[d]	X[d]	X[d]	X[d]	X		X
Neck lymph nodes exam	X	X[d]	X[d]	X[d]	X[d]	X		X
Electrocardiogram	X				X[d]			
CT or MRI head and neck	X					X		
Clinical labs[b,j]	X	X[d]		X		X		X
Pregnancy test[c]	X	X[d]						
PK sampling		X[e]	X[f]	X[f]	X[e]			
Tumor resection							X[h]	
PRV111 Patch treatment		X	X	X	X			
Tumor measurement		X[d]	X	X	X[d]	X		
RECIST criteria			X[d]	X[d]	X[d]	X		
Tumor photographs		X[d]	X[d]	X[d]	X[d]	X		X
Loco-regional recurrence								X
Histology	X						X	
Technical success		X	X	X	X			
Taste of patch and PE solution		X	X	X	X			
Concomitant therapy	X	X	X	X	X	X		X
AE assessment	X	X	X	X	X	X		X

Footnotes b & g formerly read:

b. Complete blood count and chemistry panel (creatinine, blood urea nitrogen, liver function test, electrolytes (potassium, magnesium, calcium, and sodium), coagulation, urinalysis.

g. To be completed at least 72 hours after the last treatment and prior to surgery. The surgery is to be performed no later than 28 days post the screening visit.

Now reads:

b. Complete blood count and chemistry panel (creatinine, blood urea nitrogen, liver function test, electrolytes (potassium, magnesium, calcium, and sodium), ~~coagulation,~~ urinalysis. **Human papilloma virus will be ascertained using immunohistochemistry for p16 overexpression per institutional standards**

g. To be completed at least 72 hours after the last treatment and prior to surgery. The surgery is to be performed no later than 2~~8~~**1** days post **Visit 1**~~the screening visit~~.

New footnote:

[j] Human papilloma virus will be ascertained using immunohistochemistry for p16 overexpression per institutional standards.

16 APPENDIX 2: AMENDMENT 2

The purpose of Amendment 2, dated 16 May 2018, was to respond to FDA's recommendations of 14 November 2017, and Baylor College of Medicine's PRMC, University of Texas Health IRB, and WIRB reviews. The requested revisions included:

FDA requested revisions:

- Add drug interaction information from the Platinol package insert to the Concomitant Medications section
- Renal function to be assessed based on creatinine clearance and not serum creatinine.
- Amount of drug remaining in all removed patches to be assessed.
- Bioanalysis for pharmacokinetics include assessment of the platinum-containing moieties that circulate (ie, particle size distribution) as well as total platinum.
- Attempt to optimize study dose prior to initiating the primary efficacy and safety studies that will support the NDA.

Feedback by all other institutions:

- PRMC suggested that response to treatment should be considered concomitantly from a safety and an efficacy perspective.
- PRMC requested addition of a Stage 2a to the existing Stage 2 design, to assess toxicity of the dose (if it changes from Stage 1).
- Statistical section was updated to include the specific method of evaluating efficacy response: Simon's test.

Changes to the 19 October 2017 Protocol

New text presented in **Bold** typeface; deleted text presented with a ~~Strikethrough~~

Section	Description of Change
**General Revisions**	Version number changed from ~~2.0~~ to **7.0**
	Date of Amendment 2
**Study Title:**	***Formerly read:***<br>Phase 1/2, Open-Label, Single-Arm, Safety, Tolerability, Anti-Tumor Effects, Systemic Exposure, and Device Technical Effects of PRV111 (Cisplatin Transmucosal System) in Subjects with Oral Squamous Cell Carcinoma<br><br>***Now reads:***<br>Phase 1/2, Open-Label, Single-Arm, Safety~~, Tolerability, Anti-Tumor Effects~~ **and Efficacy Dose-Finding,** Systemic Exposure, and Device Technical Effects of PRV111 (Cisplatin Transmucosal System) in Subjects with Oral Squamous Cell Carcinoma
**Study Contact Information**	***Formerly read:***<br>Serious and Unexpected Adverse Events<br>Any death, serious adverse event (SAE), or serious unexpected and suspected adverse reaction (SUSAR) occurring in a subject while receiving study drug or within 30 days of receiving study drug, even though the event may not appear to be study drug related,

Section	Description of Change
	must be promptly reported (within 24 hours) by telephone, fax, or e-mail to the Sponsor (or designee).<br>Unless otherwise instructed, SAE and SUSAR reports are to be called in to:<br>Tel: 508-404-3454<br>Who will forward a copy to:<br>Medical Monitor:<br>Alexandre Valentin, MD, MS<br>Medical Monitor<br>Monitor:<br>Susan I. Szambelan<br>Clinical Operations<br><br>***<u>Now reads:</u>***<br>~~Serious and Unexpected Adverse Events~~<br>~~Any death, serious adverse event (SAE), or serious unexpected and suspected adverse reaction (SUSAR) occurring in a subject while receiving study drug or within 30 days of receiving study drug, even though the event may not appear to be study drug related, must be promptly reported (within 24 hours) by telephone, fax, or e-mail to the Sponsor (or designee).~~<br>Unless otherwise instructed, SAE and SUSAR reports are to be ~~called in~~ **submitted** to:<br>Tel: 508-404-3454<br>**Unless otherwise instructed, SAE and SUSAR reports are to be submitted to:**<br>**Susan Szambelan**<br>Tel: 508-404-3454<br>**Email: susan.szambelan@gmail.com**<br>Who will forward a copy to:<br>Medical Monitor:<br>Alexandre Valentin, MD, MS<br>~~Medical Monitor~~<br>~~Monitor:~~<br>~~Susan I. Szambelan~~<br>~~Clinical Operations~~
**Synopsis: *Objectives***<br>**Section 5.1 Primary Objective**<br>**Section 5.2 Secondary Objectives**<br>**(PRMC/IRB)**	***<u>Formerly read:</u>***<br>Primary Objective<br>• Determine the systemic safety and local tolerability of PRV111 (Cisplatin Transmucosal System) (cisplatin drug-loaded patch+permeation enhancer [PE]) under the conditions of the trial<br>Secondary Objectives<br>• Evaluate tumor response to the PRV111 System<br>• Evaluate systemic and tumor tissue platinum levels following administration<br>• Evaluate device technical success including the residual cisplatin level in the patch after the application period<br><br>***<u>Now reads:</u>***<br>Primary Objective**s**<br>• Determine ~~the systemic safety~~ **a safe** and ~~local tolerability~~**efficacious dose** of PRV111 (Cisplatin Transmucosal System) (cisplatin drug-loaded patch + permeation enhancer [PE]) under the conditions of the trial.<br>Secondary Objectives<br>• Evaluate tumor ~~response to~~**volume shrinkage**

Section	Description of Change
	• **Assess long-term safety of the**~~the PRV111 system~~ **treatment**<br>• Evaluate systemic ~~and~~**,** tumor ~~tissue~~ **and lymph node (if available)** platinum levels following administration<br>• Evaluate device technical success including the residual cisplatin level in the patch after the application period
**Synopsis:**<br>***Methodology***<br>**Section 6.1 Overall Trial Design and Plan: Description (1st paragraph)**<br>**(PRMC/IRB)**	***Formerly read:***<br>This is a Phase 1/2, open-label, prospective, single-arm study of the systemic and local safety, tolerability, anti-tumor effects, systemic exposure, and device technical success of PRV111 (Cisplatin Transmucosal System) (cisplatin drug-loaded patch+PE) in subjects with oral squamous cell carcinoma (SCC) amenable to surgery.<br><br>***Now reads:***<br>This is a Phase 1/2, open-label, prospective, single-arm study of the ~~systemic~~**safety** and ~~local safety, tolerability, anti-tumor~~ ~~**effects**~~**efficacy dose-finding**, systemic exposure, and device technical success of PRV111 (Cisplatin Transmucosal System) (cisplatin drug-loaded patch+PE) in subjects with oral squamous cell carcinoma (SCC) amenable to surgery.
**Synopsis:**<br>***Study Design***<br>**(PRMC/IRB)**	***Formerly read:***<br>Twenty-one subjects will be enrolled for the purpose of 15 evaluable subjects for the primary analysis. All subjects will undergo evaluation by a multidisciplinary team including a Medical Oncologist and a Radiation Oncologist with expertise in Head and Neck cancer. Treatment applications will occur during 4 treatment visits, separated by 2 to 4 days (at least 48 hours), during the 3 weeks prior to the scheduled tumor resection. The study has 2 stages:<br>*Stage 1:* Six subjects will be enrolled and undergo 3 treatment applications at each of the 4 planned treatment visits. Each treatment visit will include 1 application of the PE. Starting 5 minutes after application of the PE, 1 or 2 patches (depending on tumor size) will be applied. A total of 3 treatment applications of patches will occur. These successive applications will span approximately 35 minutes. The 3 applications provide a total of 6 mg of cisplatin per visit (12 mg if 2 patches are applied at the same time). The 4 treatment visits will be separated by 2 to 4 days (at least 48 hours).<br>These 6 subjects will be monitored for local and systemic safety, tumor volume shrinkage due to the treatment, and systemic drug exposure. At the conclusion of the treatment phase of Stage 1, the Principal Investigators (PI) and Medical Monitor (reviewers) will review all available data regarding systemic safety, local tolerability, and systemic exposure. They will also review tumor volume shrinkage based on Response Evaluation Criteria in Solid Tumors (RECIST) criteria and clinical measurement of tumor volume and will make the following decisions:<br>• If at the end of Stage 1, at least 4 out of 6 subjects have no response (RECIST criteria stable disease [SD] or progressive disease [PD]) AND <2 subjects have a dose-limiting toxicity (DLT), the dose will be increased to 5 treatment applications per treatment visit for Stage 2.<br>• If at the end of Stage 1, at least 3 out of 6 subjects have a response (RECIST criteria complete response [CR] or partial response [PR]), AND <2 subjects have a DLT, the dose will remain the same for Stage 2.<br>• If at the end of Stage 1, at least 3 out of 6 subjects have a response AND ≥2 subjects have a DLT, the dose will be considered intolerable and will be de-escalated for Stage 2. De-escalation will consist of 2 treatment applications at each of the 4 planned treatment visits.

Section	Description of Change
	• If at the end of Stage 1, fewer than 3 out of 6 subjects have a response AND >2 subjects have a DLT, the dose will be considered intolerable and the study will be stopped.<br>An interim safety report will be generated after all subjects in Stage 1 have completed all observations.<br>*Stage 2:* An additional 15 subjects will be enrolled in Stage 2 for the purpose of 15 total evaluable in Stage 1 and 2 for the primary analysis. Subjects will receive either 2, 3, or 5 treatment applications at each of the 4 planned treatment visits, depending on the results of Stage 1.<br>*Note:* If in the clinical judgment of the Medical Monitor and the PIs, clinically relevant safety issues are identified, the dose regimen may be reduced at any time during the conduct of the study.<br>After completing their study treatments, a post-treatment visit at a minimum of 72 hours after the last treatment visit and prior to surgery will assess tumor response and safety. The surgery is to be performed no later than 21 days post Visit 1.<br>All subjects will subsequently undergo standard of care, surgical ablation of the tumor and satellite nodes, and possibly additional radiotherapy and chemotherapy. All subjects will be followed for up to 6 months post surgery at 3 visits: 1, 3, and 6 months post-surgery for loco-regional response and safety.<br><br>***Now reads:***<br>~~Twenty one~~**This is a 2-stage adaptive study. For Stage 1, up to 10** subjects will be enrolled for the purpose of ~~15~~**6** evaluable subjects for the ~~primary analysis. All~~**1st stage of the study to determine a safe and efficacious dose. For Stage 2, the target goal is 16 evaluable** subjects **for the efficacy analysis of the final dose. If the dose remains the same as Stage 1, Stage 2** will ~~undergo evaluation by a multidisciplinary team including a Medical Oncologist and~~ **target 10 additional evaluable subjects but up to 21 subjects will be recruited if the dose is changed between Stage 1 and 2**.<br>**For the purpose of dose selection, tolerable dose is defined as** a ~~Radiation Oncologist~~**dose where fewer than 33% of subjects being evaluated within the safety population present** with ~~**expertise**~~**DLTs. Effective dose is determined based on the evaluable population as described** in ~~Head and Neck cancer. Treatment applications will occur during 4 treatment visits, separated by 2 to 4 days (at least 48 hours), during the 3 weeks prior to the scheduled tumor resection.~~ **the Statistical Methods section.**<br>The ~~study has~~ 2 stages **of the study are described below:**<br>*Stage 1:* ~~Six~~**Up to 10** subjects will be enrolled **for the purpose of 6 evaluable subjects** and undergo 3 treatment applications at each of the 4 planned treatment visits. Each treatment visit will include 1 application of the PE. Starting **approximately** 5 minutes after application of the PE, 1 or 2 patches (depending on tumor size) will be applied. A total of 3 treatment applications of patches will occur. These successive applications will span approximately 35 minutes. The 3 applications provide a total of 6 mg of cisplatin per visit (12 mg if 2 patches are applied at the same time). The 4 treatment visits will be separated by 2 to 4 days (at least 48 hours).<br>~~These 6 subjects will be monitored for local and systemic safety, tumor volume shrinkage due to the treatment, and systemic drug exposure. At the conclusion of the treatment phase of Stage 1, the Principal Investigators (PI) and Medical Monitor (reviewers) will review all available data regarding systemic safety, local tolerability, and systemic exposure. They will also review tumor volume shrinkage based on Response Evaluation Criteria in Solid Tumors (RECIST) criteria and clinical measurement of tumor volume and will make the following decisions:~~<br>**The following decisions will be made based on safety and efficacy of the dose:**

Section	Description of Change
	(continued below)

Dosing Rules for Stage 1:

1) If ~~at the end of Stage 1, at least 4 out of 6~~**fewer than 2 subjects experience DLTs, then the following scheme will be considered for stage 2 dosing:**

   a. **If fewer than or equal to 2 of the evaluable** subjects have ~~no~~**a** response (RECIST criteria ~~stable disease [SD]~~**CR** or ~~progressive disease [PD])~~ ~~AND <2 subjects have a dose-limiting toxicity (DLT),~~**PR**) the dose will be ~~increased~~**escalated from 3** to 5 treatment applications per treatment visit for Stage 2.

   b. If ~~at~~ **3 or more evaluable subjects have a response (RECIST criteria CR or PR),** the ~~end of~~**dose will remain the same for Stage 2 (3 applications per treatment visit).**

2) **If 2 or more subjects experience DLTs at any time during** Stage 1, ~~at least 3~~**the following scheme will be considered for dose de-escalation in Stage 2:**

   a. **If there are 6 evaluable subjects in Stage 1:**

      - **If** out of 6 **evaluable subjects, 2 or more** subjects have a response (RECIST criteria ~~complete response [~~CR or ~~partial response [~~PR~~]),~~ ~~AND <2 subjects have a DLT,~~), the dose will ~~remain the same for Stage 2~~be de-escalated to 2 applications per treatment visit.

   - If ~~at the end of Stage 1, at least 3~~ out of 6 **evaluable** subjects**, fewer than 2** have a response ~~AND ≥2 subjects have a DLT, the dose will be considered intolerable and will be de-escalated for Stage 2. De-escalation will consist of 2 treatment applications at each of the 4 planned treatment visits.~~

      - ~~If at the end of Stage 1, fewer than 3 out of 6 subjects have a response AND ≥2 subjects have a DLT,~~**(RECIST criteria CR or PR),** the dose will be considered intolerable and the study will be stopped.

   b. ~~An interim safety report will be generated after all subjects in Stage 1 have completed all observations.~~**If Stage 1 dosing was stopped prior to having 6 evaluable subjects, the dose will be de-escalated from 3 applications to 2 applications per treatment visit.**

Stage 1 Dose Determination Scheme:

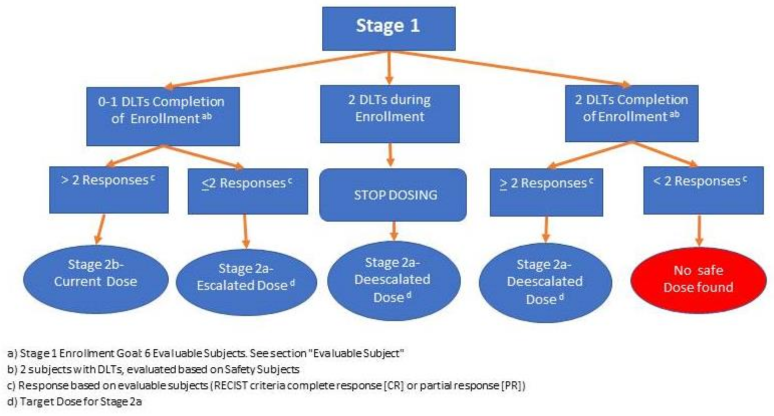

Stage 2: **In case of dose escalation or de-escalation,** Stage 2~~: An additional 15 subjects~~ will be ~~enrolled~~**conducted** in **2 parts:** Stage ~~2 for the purpose of 15 total evaluable in~~ **2a and** Stage ~~1 and 2 for the primary analysis.~~ **2b**.

**Section**	**Description of Change**
	Subjects will receive ~~either~~ 2, 3, or 5 treatment applications at each of the 4 planned treatment visits, depending on the results of Stage 1. **Up to 21 additional subjects will be enrolled in Stage 2 if necessary, for the purpose of 16 total evaluable subjects at the final dose for the efficacy analysis.**<br>**Stage 2a:**<br>**This stage will include 6 subjects (Stage 2a flowcharts). In this stage, only safety outcomes will be considered for determining the dosing regimen for the set of subjects to be enrolled in Stage 2b. All subjects enrolled in this stage will be monitored for possible DLTs.**<br>**1. If fewer than 2 subjects with DLTs in Stage 2a, enrollment will continue for the remaining number of subjects required in Stage 2b at the same dose.**<br>**2. If 2 subjects experience DLTs in Stage 2a, dosing will be re-considered. It may be stopped or, when possible, decreased for the remaining subjects enrolled in Stage 2b:**<br>**a. If “Stage 2a - Escalated Dose”, the original dose will be used for up to 10 additional evaluable subjects enrolled for Stage 2b.**<br>**b. If “Stage 2a - Same Dose”, dosing will be stopped. Additional subjects, for up to 10 evaluable subjects, will be enrolled for Stage 2b at a lower dose, consisting of 2 applications per treatment visit.**<br>**c. If “Stage 2a – De-escalated Dose”, the study will stop, as no safe dose could be determined**<br>**Stage 2a Safety Scheme in Case of Escalated Dose Decision:**<br>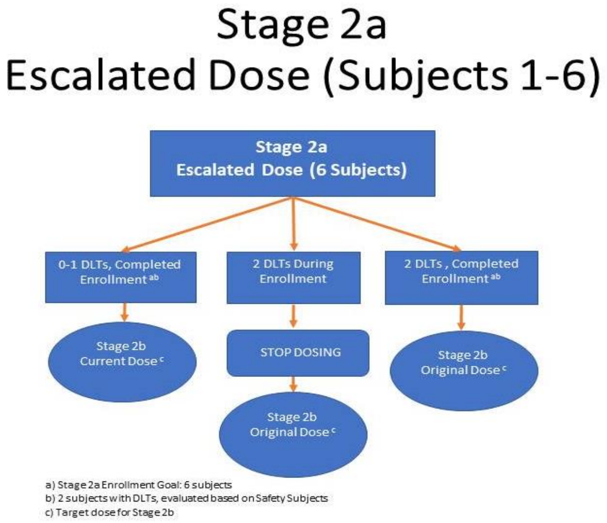<br>**Stage 2a Safety Scheme in Case of De-escalated Dose Decision**

Section	Description of Change
	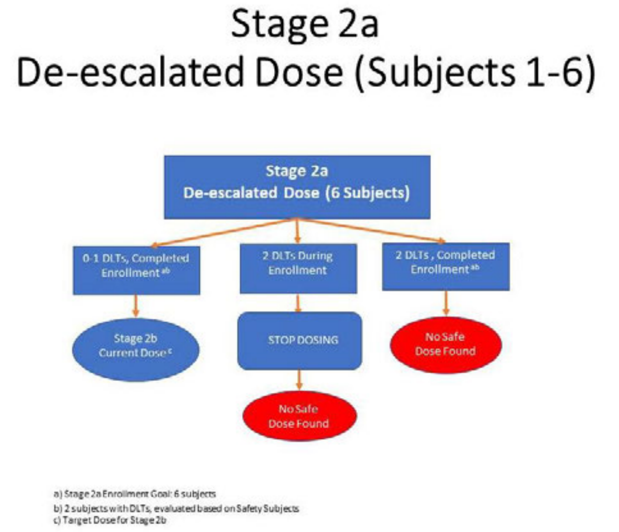<br><br><br>**Stage** ~~***Note:*** If in the clinical judgment of the Medical Monitor and the PIs, clinically relevant safety issues are identified, the dose regimen may be reduced at any time during the conduct of the study.~~<br><br>~~After completing their study treatments, a post-treatment visit at a minimum of 72 hours after the last treatment visit and prior to surgery will assess tumor response and safety.~~**2b: In this stage both safety and efficacy will be evaluated. All subjects will be monitored for possible DLTs (Stage 2b – All Doses flowchart).**<br><br>**Stopping Rules at Stage 2b:**<br><br>**If 3 subjects with DLTs, no additional subjects will be dosed.**<br><br>**Efficacy Rules at Stage 2b**<br><br>**Efficacy rules are described in the statistical plans for this stage since it is end of the study.**<br><br>**Stage 2b Safety and Efficacy Scheme:**<br><br>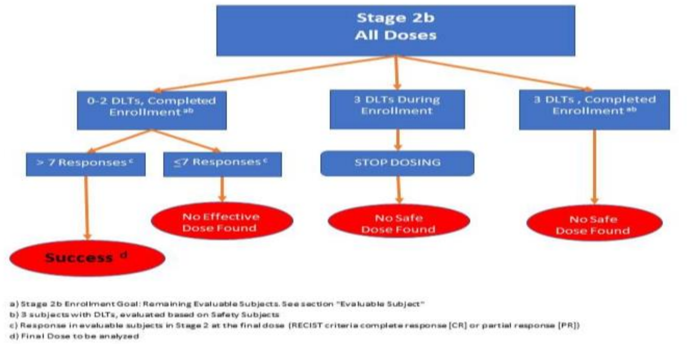<br><br><br>***Note:*** The surgery is to be performed no later than 21 days post Visit 1.<br><br>All subjects will subsequently undergo standard of care, surgical ablation of the tumor and satellite nodes, and possibly additional radiotherapy and chemotherapy. All subjects will be followed for up to 6 months post-surgery at 3 visits: 1, 3, and 6 months post-surgery for loco-regional response and safety.

Section	Description of Change
**Synopsis:** ***Number of Subjects***<br>**(PRMC/IRB)**	***Formerly read:***<br>Twenty-one subjects will be enrolled at up to 4 sites in the United States for the purpose of 15 evaluable subjects for the primary analysis.<br><br>***Now reads:***<br>~~Twenty-one~~**Up to 31** subjects will be enrolled at up to 4 sites in the United States for the purpose of **16** evaluable subjects **at the final dose** for the ~~primary~~**efficacy** analysis.
**Synopsis:** ***Diagnosis and Main Criteria for Inclusion*** **(#2 & #4)**<br>**Section 7.1 Inclusion Criteria (#2 & #4)**<br>**(Clarification)**	***Formerly read:***<br>2. Pathologically confirmed T1 (<2 cm) or T2 (>2 cm but <4 cm) SCC of the lip or oral cavity (anterior 2/3 of the tongue, floor of mouth, lower and upper gingiva, salivary gland and hard palate).<br>4. Tumor must be amenable to surgical resection approximately 3 weeks after initiation of PRV111 System<br><br>***Now reads:***<br>2. Pathologically confirmed T1 (≤2 cm) or T2 (>2 cm but <4 cm) SCC of the lip or oral cavity (anterior 2/3 of the tongue, floor of mouth, lower and upper gingiva, salivary gland ~~and~~, hard palate**, and buccal tissue**).<br>4. Tumor must be amenable to surgical resection ~~approximately 3 weeks after initiation of PRV111 System~~**no later than 21 days post Visit 1**.
**Synopsis:** ***Diagnosis and Main Criteria for Inclusion*** **(#7)**<br>**Section 7.1 Inclusion Criteria (#7)**<br>**(FDA)**	***Formerly read:***<br>7. Adequate renal function as demonstrated by serum creatinine ≤1.5 mg/dL times the upper limit of normal (ULN), or creatinine clearance ≥50 mL/min (calculated using the Cockcroft-Gault formula).<br><br>***Now reads:***<br>7. Adequate renal function as demonstrated by ~~serum creatinine ≤1.5 mg/dL times the upper limit of normal (ULN), or~~ **renal** creatinine clearance ≥50 mL/min (calculated using the Cockcroft-Gault formula).
**Synopsis:** ***Test Product, Dose and Mode of Administration***<br>***(1st three sentences after bullet list)***<br>**(PRMC/IRB)**	***Formerly read:***<br>The PE solution is applied to the oral tumor prior to the first patch applied at each treatment visit. After 5 minutes, 1 to 2 PRV111 patches (depending on tumor size) are applied. The patch(es) remain on the tumor for 10 minutes, then are removed and replaced with a new patch(es).<br><br>***Now reads:***<br>The PE solution is applied to the oral tumor prior to the first patch applied at each treatment visit. After **approximately** 5 minutes, 1 to 2 PRV111 patches (depending on tumor size) are applied. The patch(es) remain on the tumor for **approximately** 10 minutes, then are removed and replaced with a new patch(es).
**Synopsis:** ***Duration of Treatment***<br>**(Clarification)**	***Formerly read:***<br>Up to 3 weeks of treatment, prior to the scheduled resection of the tumor.<br><br>***Now reads:***

Section	Description of Change
	Up to 3 weeks of treatment~~,~~ prior to the scheduled resection of the tumor **and 6 months post-surgical follow-up.**
**Synopsis:** ***Criteria for Evaluation***<br>**(PRMC/IRB)**	***<u>Formerly read:</u>***<br>*Safety/Tolerability evaluation based on:*<br>• Adverse events (AEs)<br>• Vital signs and weight<br>• Physical examination, including sensori-motor and audition status<br>• Blood and urinary tests: complete blood count, chemistry panel, and urinalysis<br>• Electrocardiogram<br>• Oral examination: including observation for mucositis and other signs of local reaction to the test article<br>*Tumor response evaluation, based on:*<br>• RECIST clinical assessment (CR, PR, SD, or PD)<br>• Caliper measurements of tumor volume<br>• Computed tomography or magnetic resonance imaging of the head and neck<br>• Histology at the time of surgical excision<br>• Loco-regional control: subjects will be followed-up for disease recurrence and safety following surgery<br>*Pharmacokinetics:*<br>• Blood and tumor tissue platinum levels<br>*Technical Success will be defined as:*<br>• Successful adhesion of the patch to the target lesion<br>• Successful delivery (including release and permeation) of the drug to the target lesion, without getting to surrounding oral surfaces<br>• Successful peel of the used patch after 10 minutes without damaging the target lesion underneath<br>• Adhesion of the backing to the polymeric matrix embedded CLPs<br>*Other criteria to be considered:*<br>• Taste of the PE solution<br>• Taste of the PRV111 patch<br>• Residual cisplatin level in the patch after the application period<br><br>***<u>Now reads:</u>***<br>*Safety/Tolerability evaluation based on:*<br>• Adverse events (AEs)<br>~~• Vital signs and weight~~<br>• Physical examination, ~~including sensori-motor and audition status~~**vital signs, and weight**<br>• Blood and urinary tests: complete blood count, chemistry panel, and urinalysis<br>• Electrocardiogram<br>• Oral examination: including observation for mucositis and other signs of local reaction to the test article<br>• ~~*Tumor response evaluation*~~**Neck lymph nodes examination**<br>*Efficacy, based on:*<br>• **Tumor photography and measurements**<br>• RECIST clinical assessment (CR, PR, SD, or PD**). Response defined as CR or PR; non-response defined as SD or PD.**

Section	Description of Change
	• ~~Caliper~~**Ruler** measurements of tumor volume<br>• Computed tomography or magnetic resonance imaging of the head and neck<br>• Histology at the time of surgical excision<br>• Loco-regional control: subjects will be followed-up for disease recurrence and safety following surgery<br>*Pharmacokinetics:*<br>• ~~Blood and tumor tissue~~**Whole blood samples will be assessed for total** platinum ~~levels~~ **content**<br>*Technical Success will be defined as:*<br>~~• Successful adhesion of the patch to the target lesion~~<br>~~• Successful delivery (including release and permeation) of the drug to the target lesion, without getting to surrounding oral surfaces~~<br>~~• Successful peel of the used patch after 10 minutes without damaging the target lesion underneath~~<br>• ~~Adhesion of the backing to the polymeric matrix embedded CLPs~~<br>~~*Other criteria to be considered:*~~<br>• Taste of the PE solution<br>• Taste of the PRV111 patch<br>• **Adhesion, including the time of adhesion of PRV111 patch**<br>• **PRV111 patch peel-off**<br>• Residual cisplatin level in the patch after the application period. **All PRV111 patches will be assessed for the amount of residual cisplatin in each patch after each application period. If the product detaches prematurely, the residual cisplatin will be assessed and the time of detachment will be noted.**
**Synopsis:** ***Statistical Methods***<br>**(PRMC/IRB)**	***Formerly read:***<br>Data from this study will be assessed at 4 timepoints:<br>• Memo to file: after 6 subjects in Stage 1 finish PRV111treatment, response and DLTs will be assessed to determine Stage 2 dosing.<br>• Interim report: after 6 subjects in Stage 1 finish all observations up to 6 months, safety data will be analyzed.<br>• Interim report: after all subjects in Stage 2 finish PRV111 treatment, efficacy, PK, and technical data will be analyzed. Subjects will continue to be followed for safety, so this report will have whatever safety data exist at this point in time.<br>• Final report: after all subjects complete all observations.<br>All summaries will be presented by dose, as appropriate. Incidence of pre-surgery AEs will be summarized using number and percent for each System Organ Class and Preferred Term, including investigator assessment of relatedness and severity grade. Other safety assessments will be summarized using mean, standard deviation, median, minimum, and maximum for continuous variables and using number and percent for categorical assessments. Tumor volume will be summarized using mean, standard deviation, median, minimum, maximum, and 95% confidence interval. RECIST clinical assessment (CR, PR, SD, or PD) will be summarized using number and percent. A 2-stage design will be used for Stage 1 to Stage 2 dosing decision and will be based on incidence of DLTs and on response using RECIST clinical assessment.<br><br>Probability of Change in Treatment Regimen<br>It is expected that at least 15 of 21 enrolled subjects will be evaluable. The probability of a change in treatment regimen is presented in the following table.

<table>
<tr><th>Section</th><th>Description of Change</th></tr>
<tr><td></td><td>

π	Prob. of Change in treatment regimen
0.3	0.84
0.4	0.69
0.5	0.50

π= true probability of success for the treatment regimen in Stage 1

Power and Sample Size

At the end of Stage 2, the null hypothesis for the anti-tumor effect of PRV111, H0: π=0.3 will be tested against the 1-sided alternative H1: π>0.3 at 2.5% level. If the number of applications is not increased after Stage 1, the test will be performed using all evaluable subjects. If the number of applications is increased after Stage 1, the test will be performed using all evaluable subjects from Stage 2. For 15 evaluable subjects, the power of the test is 81.1% if the true probability of success for the treatment regimen in Stage 1 is 0.65 and 92.2% if this success probability is 0.70. For 10 subjects, the power of the test is 78.8% if the true probability of success for the treatment regimen in Stage 2 is 0.70 and 90.8% if this success probability is 0.75.

PK

PK parameters will include renal clearance (CLr), AUC, C_{max}, C_{min}, T_{max}, and $t_{1/2}$.

<u>Now reads:</u>

Safety outcomes resulting in a DLT will be used during the conduct of this study to select doses being tested according to pre-defined thresholds of toxicity.

- Local and systemic safety and tolerability will be summarized using descriptive statistics.

  The number of subjects with DLTs will be capped to be fewer than 2 subjects with DLTs out of 6 new subjects enrolled.

  The treatment regimen will be deemed tolerable if fewer than a third of the subjects exposed to it present DLTs.
- Efficacy based on RECIST criteria will be summarized, and the null hypothesis that the probability of response is at most 30%, where a response is the occurrence of CR or PR will be tested using a 1-sided test exact binomial test at level 0.06.
- Other efficacy assessments such as change in tumor volume or tumor histology will be summarized using descriptive statistics.
- Systemic and tumor and lymph nodes (if available) platinum levels will be summarized using descriptive statistics.
- Variables measuring technical success (including residual cisplatin level in the patch) will be summarized using descriptive statistics.

All summaries will be presented by dose, as appropriate. Incidence of pre-surgery AEs will be summarized using number and percent for each System Organ Class and Preferred Term (MedDRA), including investigator assessment of relatedness and severity grade.

All assessments will be listed and summarized using mean, standard deviation, median, minimum, and maximum for continuous variables and using number and percent for categorical assessments.
</td></tr>
</table>

<table>
<tr><th>Section</th><th>Description of Change</th></tr>
<tr><td></td><td>
Data from this study will be summarized at 2 timepoints:
• Final report at End of Stage 2: after all subjects complete PRV111treatment, efficacy, PK, and technical data will be analyzed, including all available safety data from the long-term follow-up.
• Long term safety report: after all subjects complete all observations.

Power and Sample Size
The planned enrollment for the final dose is intended to obtain N= 16 evaluable subjects.
The planned enrollment for the final dose is intended to recruit N= 16 evaluable subjects.
In the first stage, 6 evaluable subjects will be accrued. If there are 2 or fewer responses in these 6 subjects, the dose will be escalated. Otherwise, the dose will remain the same or de-escalated based on safety data. Additional subjects will be accrued for a total of 16 evaluable subjects at the final dose.
If the dose is not increased after Stage 1, the test will be performed using all evaluable subjects from Stage 1 and 2. If the dose is adjusted after Stage 1, the test will be performed using all evaluable subjects from Stage 2.
Based on Simon's 2-stage procedure, the null hypothesis that the true response rate is 0.30 will be tested against a one-sided alternative. The null hypothesis will be rejected if 8 or more responses are observed in the 16 evaluable subjects at the final dose. This design yields a type I error rate of 0.06 and power of 85.1% when the true response rate is 0.65.
<table>
<tr><th>Null response rate: P_0</th><th>Alt. response rate: P_1</th><th>Alpha one-tailed</th><th>Power</th><th>n1</th><th>r1</th><th>N</th><th>R</th></tr>
<tr><td>0.30</td><td>0.65</td><td>0.06</td><td>85.1%</td><td>6</td><td>2</td><td>16</td><td>7</td></tr>
</table>
N is the total number of evaluable subjects at the final dose
n_1 is the number of evaluable subjects accrued during Stage 1
r_1, if r_1 or fewer responses are observed during stage 1, escalate the dose if safety requirements are met
R, if *R* or fewer responses are observed by the end of Stage 2 in N subjects, then an effective dose is not found

Pharmacokinetics
PK parameters will include AUC, C_{max}, C_{min}, T_{max}, and $t_{1/2}$.
</td></tr>
<tr><td>**Section 6.2 Discussion of Trial Design**
(Clarification)</td><td>
Formerly read:
This study is intended to establish the first safety observations in man and the feasibility of using 3 doses of the PRV111 (Cisplatin Transmucosal System) prior to the surgical removal of SCC tumors of the oral cavity.

Now reads:
This study is intended to establish the first-**in-human** safety **and efficacy** observations ~~in man~~ and the ~~feasibility of using 3 doses~~ **optimal dose** of the PRV111 (Cisplatin Transmucosal System) prior to the surgical removal of SCC tumors of the oral cavity.
</td></tr>
<tr><td>**Section 8.1.2 Packaging (1st sentence)**
(Clarification)</td><td>
Formerly read:
The PRV111 4 cm^2 patch is packaged in a self-sealing pouch, 2.25” x 4.0” (constructed of medical grade paper).
</td></tr>
</table>

Section	Description of Change
	Now reads:<br>The PRV111 4 cm^2 patch **and Permeation Enhancer Powder for Reconstitution** is packaged in a self-sealing pouch, 2.25” x 4.0” (constructed of medical grade paper).
**Section 8.1.5 Dosing and Administration**<br>**(PRMC/IRB)**	***Formerly read:***<br>In Stage 2, subjects will receive either 2, 3, or 5 treatment applications at each of the 4 planned treatment visits, depending on the results of Stage 1.<br>Six subjects will be enrolled into Stage 1 to include the following criteria for decisions regarding dose escalation:<br>• If at the end of Stage 1, at least 4 out of 6 have no response AND fewer than 2 have a dose-limiting toxicity (DLT), the dose will be increased to 5 treatment applications per treatment visit for Stage 2.<br>• If at the end of Stage 1, at least 3 out of 6 have a response AND <2 have a DLT, the dose will remain the same for Stage 2.<br>• If at the end of Stage 1, at least 3 out of 6 subjects have a response AND ≥2 subjects have a DLT, the dose will be considered intolerable and will be de-escalated for Stage 2. De-escalation will consist of 2 treatment applications at each of the 4 planned treatment visits.<br>• If at the end of Stage 1, fewer than 3 out of 6 subjects have a response AND ≥2 subjects have a DLT, the dose will be considered intolerable and the study will be stopped.<br>Refer to the Pharmacy Manual for detailed instructions for use.<br>Table 2 presents terminology used for dosing and treatment in this study. Treatment will consist of 4 treatment visits, separated by 2-4 days, for up to 3 weeks prior to the scheduled tumor resection. A treatment application will consist of pre-treatment with the PE solution and application of the PRV111 patches. Treatment is expected to be 35 minutes for each treatment visit.<br><br>***Now reads:***<br>In Stage 2 (**Stage 2a and Stage 2b**), subjects will receive ~~either~~2, 3, or 5 treatment applications at each of the 4 planned treatment visits, depending on the results of Stage 1.<br>~~Six subjects will be enrolled into Stage 1 to include the following criteria for decisions regarding dose escalation:~~<br>• ~~If at the end of Stage 1, at least 4 out of 6 have no response AND fewer than 2 have a dose-limiting toxicity (DLT), the dose will be increased to 5 treatment applications per treatment visit for Stage 2.~~<br>• ~~If at the end of Stage 1, at least 3 out of 6 have a response AND <2 have a DLT, the dose will remain the same for Stage 2.~~<br>• ~~If at the end of Stage 1, at least 3 out of 6 subjects have a response AND ≥2 subjects have a DLT, the dose will be considered intolerable and will be de-escalated for Stage 2. De-escalation will consist of 2 treatment applications at each of the 4 planned treatment visits.~~<br>• ~~If at the end of Stage 1, fewer than 3 out of 6 subjects have a response AND ≥2 subjects have a DLT, the dose will be considered intolerable and the study will be stopped.~~<br>Refer to the Pharmacy Manual for detailed instructions ~~for use~~on **applying the PRV111 patches.**

Section	Description of Change
	Table 2 presents ~~terminology~~ **definition** used for dosing and treatment in this study. ~~Treatment will consist of 4 treatment visits, separated by 2-4 days, for up to 3 weeks prior to the scheduled tumor resection. A treatment application will consist of pre-treatment with the PE solution and application of the PRV111 patches~~. Treatment is expected to be **approximately** 35 minutes for each treatment visit.
**Section 8.5 Study Drug Handling and Accountability**	***Formerly read:***<br>The Investigator (or designee) will:<br>• Ensure study drug supplies are stored between 2º and 8º C (PRV111 study kits)) in an appropriately controlled limited-access room in a secure location.<br>• Dispense the appropriate number of study patches.<br>• Record pertinent information regarding the number of study kits used (eg, subject identification code, date of use, number of patches used, etc.) on the Drug Dispensing Log, or other appropriate inventory form. This inventory will be maintained throughout the trial and will be periodically reviewed by a Sponsor representative.<br>• All used patches must be sent to the pharmacokinetics (PK) lab so that residual levels of cisplatin in the patches will be assessed. Preparation for shipping and shipping logistics may be found in the Pharmacy Manual.<br><br>***Now reads:***<br>The Investigator (or designee) will:<br>• Ensure study drug supplies (**PRV111 study kits**) are stored between 2º and 8º C ~~(PRV111 study kits))~~ in an appropriately controlled limited-access room in a secure location.<br>• Dispense the appropriate number of study patches.<br>• Record pertinent information regarding the number of study kits used (eg, subject identification code, date of use, number of patches used, etc.) on the Drug Dispensing Log, or other appropriate inventory form. This inventory will be maintained throughout the trial and will be periodically reviewed by a Sponsor representative.<br>• All used **and unused** patches **during a treatment visit** must be sent to the pharmacokinetics (PK) lab ~~so that~~**. They should be separated into properly labeled sealing plastic bags. The used patches will be processed by the PK lab to assess the** residual levels of cisplatin ~~in the patches will be assessed.~~ **of each application**. Preparation for shipping and shipping logistics ~~may~~**will** be found in the ~~Pharmacy~~**Laboratory** Manual.
**Section 8.6 Study Drug Disposal**<br>**(PRMC/IRB)**	***Formerly read:***<br>Periodically throughout and at the conclusion of the trial, an inventory of unused study drug kits will be conducted by a Sponsor representative. At the completion of the trial, all unused study drug materials must be returned to the Sponsor or destroyed on site at the direction of the Sponsor in an appropriate manner according to institutional policy. Destruction of the kits will be documented, and disposition records will be reviewed by a Sponsor representative.<br>At the termination of the trial, a final drug accountability review and reconciliation must be completed and any discrepancies must be investigated and their resolution documented.

Section	Description of Change
	Now reads:<br>Periodically throughout and at the conclusion of the trial, an inventory of unused study drug kits will be conducted by a Sponsor representative. ~~At the completion of the trial, all~~ **All** unused study drug materials **during a treatment visit** must be ~~returned~~**sent** to the ~~Sponsor or destroyed on site at the direction of the~~ ~~**Sponsor**~~ **PK lab in labelled, sealing plastic bags and they will be disposed** in an appropriate manner according to ~~institutional~~ the **laboratory** policy. ~~Destruction of the kits will be documented, and disposition records will be reviewed by a Sponsor representative.~~<br>**At the treatment completion, any remaining unopened and unused study drug in the kit will be sent back to the sponsor. Preparation for shipping and shipping logistics will be found in the Laboratory Manual.** At the termination of the trial, a final drug accountability review and reconciliation must be completed and any discrepancies must be investigated and their resolution documented.
**Section 8.7 Concomitant Medications and Therapy**<br>**(PRMC/IRB)**	***Formerly read:***<br>Concomitant medications and therapies are other prescription medications, over-the-counter drugs, or dietary supplements that a study participant takes in addition to the investigational product. Based on FDA's recommendation, anti-emetic prophylaxis will be mandatory.<br>Symptomatic anti-emetics will be used during the study.<br>For emesis prophylaxis, subjects will receive ondansetron 8 mg orally approximately 30 minutes before administration of the PRV111 treatment. After treatment, subjects may self-administer ondansetron 8 mg orally or via feeding tube approximately every 12 hours for 24 to 48 hours for persistent nausea.<br>Alternatively, subjects may receive promethazine 12.5 mg orally approximately 60 minutes before administration of the PRV111 treatment. After treatment, subjects may self-administer promethazine 12.5 mg approximately every 4 to 6 hours for 24 to 48 hours for persistent nausea.<br>Subjects with diarrhea may be supported clinically with loperamide, at dosing schedules recommended in the package insert.<br>Based upon the Investigator's judgment, the subject's tongue might need to be locally anesthetized to prevent accidental touching and removal of the patch.<br><br>***Now reads:***<br>Concomitant medications and therapies ~~are~~**include** other prescription medications, over-the-counter drugs, or dietary supplements that a study participant takes in addition to the investigational product. Based on FDA's recommendation, anti-emetic prophylaxis will be mandatory.<br>Symptomatic anti-emetics will be used during the study.<br>~~For emesis prophylaxis~~, **All** subjects will receive ~~ondansetron 8 mg orally~~ **emesis prophylaxis**, administered approximately 30 minutes before ~~administration of the~~ PRV111 treatment. **The drug used may be either ondansetron or promethazine, the dose to be determined at the discretion of the PI**. After treatment, subjects may self-administer ~~ondansetron 8 mg~~ orally or via feeding tube approximately every 12 hours for 24 to 48 hours for persistent nausea. Alternatively, subjects may receive promethazine 12.5 mg orally approximately 60 minutes before administration of the PRV111 treatment.

Section	Description of Change
	After treatment, subjects may self-administer promethazine 12.5 mg approximately every 4 to 6 hours for 24 to 48 hours for persistent nausea.<br><br>Subjects with diarrhea ~~may be supported clinically with loperamide~~ **will receive appropriate interventions/medications** at ~~dosing schedules recommended in~~ the ~~package insert~~ **discretion of the treating physician consistent with current clinical practice.**<br><br>Based upon the Investigator's judgment, the ~~subject's tongue~~ **subject** might **need topical anesthetic (ie. Lidocaine)** ~~be locally anesthetized~~ to **ameliorate discomfort** or prevent accidental touching and removal of the patch.
**Section 8.8 Drugs Contraindicated with PRV111**<br>**(FDA)**	***<u>Added</u>***<br>**Plasma levels of anticonvulsant agents may become subtherapeutic during cisplatin therapy.**
**Section 9.1.1 Confirmation of Squamous Cell Carcinoma**<br>**Section 9.3.4 Safety Laboratory Assessments**<br>**(FDA)**	***<u>Added in 9.1.1:</u>***<br>**Human papilloma virus will be ascertained using immunohistochemistry for p16 overexpression per institutional standards.**<br><br>***<u>Deleted in 9.3.4:</u>***<br>~~Human papilloma virus will be ascertained using immunohistochemistry for p16 overexpression per institutional standards.~~
**Section 9.1.3 Demographics, Medical, and Medication History**<br>**(PRMC/IRB)**	***<u>Formerly read:</u>***<br>Demographic information, including age, sex, race, history of tobacco and alcohol use, occupation, and medical history will be obtained by interviewing the subject.<br><br>***<u>Now reads:</u>***<br>Demographic information, including age, sex, race, history of tobacco and alcohol use, ~~occupation~~, and medical history will be obtained by interviewing the subject.
**Section 9.1.4 Pregnancy Test**<br>**(PRMC/IRB)**	***<u>Formerly read:</u>***<br>A serum pregnancy test will be performed to establish eligibility for female subjects of childbearing potential. Urine pregnancy test will be collected on Day 1 pre-treatment.<br><br>***<u>Now reads:</u>***<br>A serum pregnancy test ~~will be performed~~ to establish eligibility for female subjects of childbearing potential~~. Urine~~ **will be performed. In addition, a negative urine** pregnancy test ~~will~~ **must** be ~~collected on Day 1 pre-~~**documented within 7 days prior to the first** treatment **visit, and at Visit 5**.
**Section 9.2.1 Tumor Photography and Measurements**<br>**(PRMC/IRB)**	***<u>Formerly read:</u>***<br>The primary lesion (both longest and shortest axes) will be marked before PRV111 treatment by at least 4 points, which will be 0.5 cm away from the lesion; this will be done to allow for a reliable clinical assessment of response to treatment.<br><br>***<u>Now reads:</u>***

Section	Description of Change
	The primary lesion (~~both longest and shortest axes~~) will be ~~marked~~ **photographed** before PRV111 treatment ~~by at least 4 points, which will be 0.5 cm away from the lesion;~~ **This** will be done to allow for a ~~reliable~~ clinical assessment of response to treatment. **For details refer to Study Manual for instructions.**<br>**The tumor will be measured in cm using a disposable plastic ruler. Three dimensions must be recorded (Length x Width x Height). The following formula will be used to calculate tumor volume (cm^3)**<br>$\boldsymbol{L\ x\ W\ x\ H\ x\ \pi/6}$
**Section 9.2.2 Response and Non-Response (RECIST Criteria)**<br>**(PRMC/IRB)**	***Formerly read:***<br>Response will be defined using RECIST criteria as CR or PR. SD or PD will be defined as a non-response.<br><br>***Now reads:***<br>~~Response will be defined using RECIST criteria as CR or PR. SD or PD will be defined as a non-response~~ **The terms "response" and "non-response" will be defined according to the RECIST criteria. Response will be defined** as CR or PR**.** ~~SD or PD~~ **Non-Response** will be defined as ~~a non-response~~**SD or PD** (Table 4)**.**
**Section 9.2.3 Tumor Photographs**<br>**(PRMC/IRB)**	**Deleted section**
**Section 9.2.4 Computed Tomography or Magnetic Resonance Imaging of the Head and Neck**<br>**(Clarification)**	***Updated numbering following omission of the previous section (now Section 9.2.3)***<br><br>***Formerly read:***<br>A computed tomography scan (CT) or magnetic resonance imaging (MRI) of the head and neck will be performed.<br><br>***Now reads:***<br>A computed tomography scan (CT) or magnetic resonance imaging (MRI) of the head and neck will be performed. **For each subject, the same imaging modality should be used at Screening and Visit 5 for consistency.**
**Section 9.2.4 Histopathology Response to Treatment**<br>**(Clarification)**	***Formerly read:***<br>Histological samples obtained at Baseline and from the surgical tumor excision will be compared.<br><br>***Now reads:***<br>Histological samples obtained (Laboratory Manual) at Baseline and from the surgical tumor excision will be compared.
**Section 9.3.1 - Physical Examination, Vital Signs, Weight**<br>**(Clarification)**	***Formerly read:***<br>A complete general physical examination, including height and weight and vital signs (VS), will be assessed and recorded.<br><br>***Now reads:***<br>~~A complete general physical examination, including height and weight and vital signs (VS), will be assessed and recorded.~~ **A complete physical examination (PE) will be performed by the Investigator or Sub-investigator. The PE will include a review of**

<table>
<tr><th>Section</th><th>Description of Change</th></tr>
<tr><td></td><td>**all body systems (head, eyes, nose and throat, cardiovascular, respiratory, gastrointestinal, musculoskeletal, neurological, lymph nodes, dermatological, extremities/joints), and weight.**</td></tr>
<tr><td>**Section 9.3.4 Safety Lab Assessment**

(PRMC/IRB)</td><td>***Formerly read:***
Clinical laboratories to be assessed will be Complete Blood Count (CBC) and chemistry panel (creatinine, blood urea nitrogen [BUN], liver function test [LFT]), electrolytes (potassium, magnesium, calcium, and sodium), urinalysis.

Now reads:
Clinical laboratories to be assessed will be ~~Complete Blood Count (CBC) and chemistry panel (creatinine to determine creatinine clearance, blood urea nitrogen [BUN], liver function test [LFT]), electrolytes (potassium, magnesium, calcium, and sodium), urinalysis~~ **as in Table 6.**

Inserted Table 6
<table>
<tr><th>COMPLETE BLOOD COUNT</th><th colspan="2">CHEMISTRY PANEL</th></tr>
<tr><td>• hematocrit
• hemoglobin
• platelet count
• red blood cell count
• white blood cell count and differential (absolute and percentage); neutrophils, lymphocytes, monocytes, eosinophils, basophils, and reticulocyte count</td><td rowspan="3">• alanine aminotransferase
• albumin
• alkaline phosphatase
• aspartate aminotransferase
• bilirubin (direct and total)
• blood urea nitrogen
• calcium
• CO2</td><td rowspan="3">• chloride
• creatinine[a]
• gamma glutamyl transferase
• glucose
• magnesium
• potassium
• sodium
• total protein</td></tr>
<tr><td>URINALYSIS</td></tr>
<tr><td>• urinalysis dipstick with reflex microscopic examination for abnormalities</td></tr>
</table>
[a] Creatinine Clearance will be used to assess renal function using Cockcroft-Gault Equation</td></tr>
<tr><td>**Section 9.3.6.8.1 Events to Be Reported and Timeframe**

Section 9.3.6.10 Follow-up Information</td><td>***Deleted the term "telefax" at the end of the sentence from Section 9.3.6.8.1***

Deleted "preferably" and "telefax or" in Section 9.3.6.10</td></tr>
<tr><td>**Section 9.4 Pharmacokinetics Assessment**

(Correction)</td><td>***Formerly read:***
Blood samples for plasma levels of total platinum will be assessed for all subjects.

Now reads:
~~Blood~~**Whole blood** samples ~~for plasma levels of total platinum~~ will be assessed for **PK parameters AUC, C_{max}, C_{min}, T_{max}, and $t_{1/2}$, and total platinum content for** all subjects **(See Laboratory Manual for sample collection and shipping instructions). Tumor and lymph node cis platinum content may be provided as well.**</td></tr>
<tr><td>**Section 9.6.1 Screening (Visit 0) (FDA)**</td><td>***Formerly read:***
Occurs within 28 days prior to initial treatment (Visit 1).</td></tr>
</table>

<table>
<tr><th>Section</th><th>Description of Change</th></tr>
<tr><td></td><td>Obtain and document consent from potential subject on Informed Consent Form (ICF)
Review inclusion/exclusion criteria to determine eligibility
Review medical and medications history to determine eligibility based on inclusion/exclusion criteria
Perform a general physical examination and check VS
Perform CT of the chest (with or without contrast)
Perform a systematic and thorough visual and manual examination of the oral cavity mouth and cervical and local lymph nodes examination
Perform an ECG
Perform CT or MRI head and neck
Perform clinical labs: blood draw for CBC, chemistry panel (creatinine, BUN, LFT, electrolytes [potassium, magnesium, calcium, and sodium], hCG [serum]) for females of childbearing potential and perform urinalysis.
AE documentation begins when the subject signs the ICF
Schedule study visits
<u>Now reads:</u>
Confirmed SCC
Obtain and document consent from potential subject on Informed Consent Form (ICF)
Review inclusion/exclusion criteria to determine eligibility
Review demographics, medical and medications history to determine eligibility based on inclusion/exclusion criteria
Perform a general physical examination and check VS
Perform CT of the chest (with or without contrast)
Perform a systematic and thorough visual and manual examination of the oral cavity mouth and cervical and local lymph nodes examination
Perform an ECG
Perform CT or MRI head and neck. For each subject, the same imaging modality should be used at Screening and Visit 5 for consistency.
Perform clinical labs: blood draw for CBC, chemistry panel (creatinine for determining creatinine clearance, BUN, LFT, electrolytes [potassium, magnesium, calcium, and sodium], hCG [serum]) for females of childbearing potential and perform urinalysis.
AE documentation begins when the subject signs the ICF
Schedule study visits
Testing for the human papilloma virus</td></tr>
<tr><td>Section 9.6.2 Baseline, Treatment (Visit 1)

(Clarification and removing redundancy)</td><td><u>Formerly read:</u>
Predose Assessments (Baseline):
Verify inclusion/exclusion criteria
Perform and record result of physical examination and VS
Perform and record visual oral cavity examination and neck lymph node examination
Perform and document baseline clinical tumor measurement before the treatment
Collect clinical labs: blood draw for CBC, chemistry panel (creatinine, BUN, LFT, electrolytes [potassium, magnesium, calcium, and sodium], hCG [urine] for females of childbearing potential), and perform urinalysis
Testing for the human papilloma virus</td></tr>
</table>

Section	Description of Change
	• Administer oral anti-emetics (ondansetron 8 mg approximately 30 minutes prior to the start of PRV111 treatment, or promethazine 12.5 mg approximately 60 minutes before the start of PRV111 treatment)<br>• Photograph the tumor<br>• Draw blood for PK time -15 to 0 minutes<br>• Administer PE and PRV111 patch(es)<br>**Postdose Assessments:**<br>• Draw blood for PK 30±5 minutes, 60±5 minutes and 120 ±5 minutes.<br>• Vital signs shall be monitored for 30±5 minutes, 60±5 minutes and 120 ±5 minutes following application of the last patch applied at that treatment visit.<br>• Collect all used patches (include the ones that detached prematurely), record the time of the detachment of each patch, store the patches in the plastic bags supplied by the Sponsor (Refer to Pharmacy Manual for instructions).<br>• Assess AEs (treatment emergent AEs will be AEs that occur after the first dose of study drug)<br>• Review any concomitant therapy<br>• Schedule study visits<br><br>***Now reads:***<br>**Occurs within 28 days following Screening visit (Visit 0).**<br>**Predose Assessments (Baseline):**<br>• Verify inclusion/exclusion criteria<br>• Perform and record result of physical examination and VS<br>• Perform and record visual oral cavity examination and neck lymph node examination<br>• Perform and document baseline clinical tumor measurement ~~before the treatment~~<br>• Collect clinical labs: blood draw for CBC, chemistry panel (creatinine **for determining creatinine clearance**, BUN, LFT, electrolytes [potassium, magnesium, calcium, and sodium], hCG [urine] for females of childbearing potential) and perform urinalysis.<br>~~• Testing for the human papilloma virus~~<br>• Administer oral anti-emetics (ondansetron ~~8 mg approximately 30 minutes prior to the start of PRV111 treatment,~~ or promethazine ~~12.5 mg approximately 60 minutes~~**, the dose to be determined at the discretion of the PI)** before the start of PRV111 treatment~~)~~<br>• Photograph the tumor<br>• Draw blood for PK ~~time~~ **(** -15 to 0 minutes **predose)**<br>• Administer PE ~~and~~ **(refer to Pharmacy Manual for administration instructions)**<br>▪ **document time of application**<br>▪ **ask for taste of PE solution**<br>• **Administer** PRV111 patch(es) **and document time of each application and removal. Ask for taste of patch.**<br>**Postdose Assessments:**<br>• Draw blood for PK 30±5 minutes, 60±5 minutes and 120 ±5 minutes **following application of the last patch at that treatment visit.**<br>• Vital signs shall be monitored ~~for~~**at** 30±5 minutes, 60±5 minutes and 120 ±5 minutes~~ following application of the last patch applied at that treatment visit~~.

Section	Description of Change
	• Collect all used patches (include the ones that detached prematurely), record the time of the detachment of each patch, store the patches in the plastic bags supplied by the Sponsor (~~Refer~~**refer** to ~~Pharmacy~~**Laboratory** Manual for instructions).<br>• ~~Assess AEs (treatment emergent AEs will be AEs that occur after the first dose of study drug)~~<br>• **Record AEs as reported by subject or observed by Investigator**<br>• Review any concomitant therapy<br>• Schedule study visits
**Section 9.6.3 Treatment (Visit 2)**<br>**Section 9.6.4 Treatment (Visit 3)**<br>**Section 9.6.5 Treatment (Visit 4)**<br><br>**(clarification)**	***Formerly read:***<br>Predose Assessments:<br>• Perform and document physical examination and VS<br>• Perform and document visual oral examination and neck lymph node examination<br>• Perform and document clinical tumor measurement and RECIST criteria<br>• Administer oral anti-emetics (ondansetron 8 mg approximately 30 minutes prior to the start of PRV111 treatment, or promethazine 12.5 mg approximately 60 minutes before the start of PRV111 treatment)<br>• Photograph the tumor<br>• Draw blood for PK time -15 to 0 minutes<br>• Administer PRV111 treatment.<br>Postdose Assessments:<br>• Draw blood for PK 30±5 minutes<br>• VS shall be monitored for 30±5 minutes, 60±5 minutes and 120 ±5 minutes following application of the last patch applied at that treatment visit<br>• Collect all used patches (include the ones that detached prematurely), record the time of the detachment of each patch, store the patches in the plastic bags supplied by the Sponsor (Refer to Pharmacy Manual for instructions)<br>• Record AEs as reported by subject or observed by Investigator<br>• Review any concomitant therapy<br>• Schedule study visits<br><br>***Now reads:***<br>Predose Assessments:<br>• Perform and document physical examination and VS<br>• Perform and document visual oral examination and neck lymph node examination<br>• **Perform and document clinical tumor measurement and RECIST criteria**<br>• Administer oral anti-emetics (ondansetron ~~8 mg approximately 30 minutes prior to the start of PRV111 treatment,~~ or promethazine, **the dose to be determined at the discretion of the PI)** before the start of PRV111 treatment~~)~~<br>• Photograph the tumor<br>• Draw blood for PK ~~time~~ **(**-15 to 0 minutes predose**)**<br>• Administer **PE (refer to Pharmacy Manual for administration instructions)**<br>▪ **document time of application**<br>• **Administer PRV111 patch(es) and document time of each application and removal**<br>▪ **Document adherence of each patch**<br>Postdose Assessments:

Section	Description of Change
	• **Draw blood for PK 30±5 minutes following application of the last patch applied at that treatment visit.**<br>• **VS shall be monitored at 30±5 minutes, 60±5 minutes and 120 ±5 minutes**<br>• Collect all used patches (include the ones that detached prematurely), record the time of the detachment of each patch, store the patches in the plastic bags supplied by the Sponsor (Refer to ~~Pharmacy~~ **Laboratory Manual for instructions)**<br>• Record AEs as reported by subject or observed by Investigator<br>• Review any concomitant therapy<br>• Schedule study visits
**Section 9.6.6 Post-Treatment/Pre-Op Visit (Visit 5) (Clarification)**	***Formerly read:***<br>Occurs a minimum of 72 hours after the last visit and prior to surgery. The surgery is to be performed no later than 21 days post Visit 1.<br>• Perform and document physical examination and VS<br>• Perform and document visual oral examination and neck lymph node examination<br>• Perform CT of the head and neck<br>• Perform and document clinical tumor measurement and RECIST criteria<br>• Collect clinical labs: blood draw for CBC, chemistry panel (creatinine, BUN, LFT, electrolytes [potassium, magnesium, calcium, and sodium]), and perform urinalysis.<br>• Photograph the tumor<br>• Record AEs as reported by subject or observed by Investigator<br>• Review any concomitant therapy<br><br>***Now reads:***<br>Occurs a minimum of 72 hours after the last visit and prior to surgery. The surgery is to be performed no later than 21 days post Visit 1.<br>• Perform and document physical examination and VS<br>• Perform and document visual oral examination and neck lymph node examination<br>• Perform CT **or MRI** of the head and neck. **For each subject, the same imaging modality should be used at Screening and Visit 5 for consistency.**<br>• Perform and document clinical tumor measurement and RECIST criteria<br>• **Electrocardiogram**<br>• Collect clinical labs: blood draw for CBC, chemistry panel (creatinine, BUN, LFT, electrolytes [potassium, magnesium, calcium, and sodium]), and perform urinalysis<br>• Photograph the tumor<br>• Record AEs as reported by subject or observed by Investigator<br>• Review any concomitant therapy
**Section 9.6.7 Surgery Day (Visit 6)<br>(PRMC/IRB)**	***Formerly read:***<br>• Tumor resection as per the SOC<br>• Obtain tissue samples for:<br>o Hematoxylin & Eosin staining for histology evaluation<br>o Platinum level in the tissue<br><br>***Now reads:***

Section	Description of Change
	• Tumor resection as per the standard of care<br>• Obtain tissue samples ~~for~~(**see Laboratory Manual**):<br>o Hematoxylin & Eosin staining for histology evaluation<br>o Platinum level in the ~~tissue~~ **tumor and lymph nodes (If available)**
**Section 9.6.8 Post-Treatment Follow Up (Visits 7, 8, & 9)**<br><br>**(PRMC/IRB)**	***Formerly read:***<br>• Perform and record result physical examination and VS<br>• Perform visual oral examination and neck lymph node examination<br>• Collect clinical labs: blood draw for CBC, chemistry panel (creatinine, BUN, LFT, electrolytes [potassium, magnesium, calcium, and sodium]), and perform urinalysis Assess subject for signs of loco-regional recurrence<br>• Assessment of AEs<br>• Review any concomitant therapy<br><br>***Now reads:***<br>• Perform and record result physical examination and VS<br>• Perform visual oral examination and neck lymph node examination **to assess for signs of loco-regional recurrence**<br>• Collect clinical labs: blood draw for CBC, chemistry panel (creatinine, BUN, LFT, electrolytes [potassium, magnesium, calcium, and sodium]), and perform urinalysis**.** ~~Assess subject for signs of loco-regional recurrence~~<br>• Assessment of AEs<br>• Review any concomitant therapy
**Section 10.2 Sample Size Determination (PRMC/IRB)**	***Formerly read:***<br>At the end of Stage 2, the null hypothesis H0: π=0.3 will be tested against the 1-sided alternative H1: π>0.3 at 2.5% level using the normal approximation to the binomial distribution. If the number of applications is not increased after Stage 1, the test will be performed using all evaluable subjects. If the number of applications is increased after Stage 1, the test will be performed using all evaluable subjects from Stage 2. For 15 evaluable subjects, the power of the test is 81.1% if the true probability of success for the treatment regimen in Stage 1 is 0.65 and 92.2% if this success probability is 0.70. For 10 evaluable subjects, the power of the test is 78.8% if the true probability of success for the treatment regimen in Stage 2 is 0.70 and 90.8% if this success probability is 0.75.<br><br>***Now reads:***<br>Safety outcomes resulting in DLT will be used during the conduct of this study to select doses being tested according to pre-defined thresholds of toxicity.<br>• Local and systemic safety and tolerability will be summarized using descriptive statistics.<br>• The number of subjects with DLTs will be capped to be fewer than 2 subjects with DLTs out of 6 new subjects enrolled.<br>• The treatment regimen will be deemed tolerable if fewer than a third of the subjects exposed to it present DLTs.<br>• Efficacy based on RECIST criteria will be summarized, and the null hypothesis that the probability of response is at most 30%, where a response is the occurrence of CR or PR will be tested using a one-sided test exact binomial test at level 0.06.<br>• Other efficacy assessments such as change in tumor volume or tumor histology will be summarized using descriptive statistics.

<table>
<tr><th>Section</th><th>Description of Change</th></tr>
<tr><td></td><td>

- Variables measuring technical success (including residual cisplatin level in the patch) will be summarized using descriptive statistics.

At the end of Stage 2, the following is a statistical analysis of the sample size as it pertains to efficacy:
Power and Sample Size
The planned enrollment for the final dose is intended to obtain N=16 evaluable subjects. Based on Simon's 2-stage procedure, the null hypothesis that the true response rate is 0.30 will be tested against a 1-sided alternative. In the first stage, 6 evaluable subjects will be accrued. If there are 2 or fewer responses in these 6 subjects, the dose will be escalated. Otherwise, the dose will remain the same or de-escalated based on safety data. Additional subjects will be accrued for a total of 16 evaluable subjects at the final dose. The null hypothesis will be rejected if 8 or more responses are observed in the 16 evaluable subjects at the final dose. This design yields a type I error rate of 0.06 and power of 85.1% when the true response rate is 0.65.

<table>
<tr><th>Null response rate: P_0</th><th>Alt. response rate: P_1</th><th>Alpha one-tailed</th><th>Power</th><th>n1</th><th>r1</th><th>N</th><th>R</th></tr>
<tr><td>0.30</td><td>0.65</td><td>0.06</td><td>85.1%</td><td>6</td><td>2</td><td>16</td><td>7</td></tr>
</table>

N is the total number of evaluable subjects at the final dose
n_1 is the number of evaluable subjects accrued during Stage 1
r_1, if r_1 or fewer responses are observed during stage 1, escalate the dose if safety requirements are met
R, if *R* or fewer responses are observed by the end of Stage 2 in N subjects, then an effective dose is not found

Note: If the dose is not increased after Stage 1, the test will be performed using all evaluable subjects from Stage 1 and 2. If the dose is adjusted after Stage 1, the test will be performed using all evaluable subjects from Stage 2.
</td></tr>
<tr><td>Section 10.3</td><td>

<u>New section added:</u>

10.3 Pharmacokinetics

PK parameters will include AUC, C_{max}, C_{min}, T_{max}, and $t_{1/2}$. Systemic and tumor and lymph nodes (if available) platinum levels will be summarized using descriptive statistics.
</td></tr>
<tr><td>Section 10.4 Study Populations
(Unused)</td><td>

<u>Removed:</u>

~~10.4.2 Efficacy Population~~

~~All subjects in the Safety Population with at least 1 tumor response assessment according the RECIST Criteria will be included in the Efficacy Population.~~
</td></tr>
<tr><td>Section 10.4.2 Evaluable Population
(PRMC/IRB)</td><td>

<u>Formerly read:</u>

All subjects in the Efficacy Population who complete at least 3 out of 4 PRV111 treatment visits will be included in the Evaluable Population. This population will be used for the primary analysis.

<u>Now reads:</u>

All subjects in the Efficacy Population who complete at least ~~3 out~~**75%** of ~~4 PRV111 treatment visits~~ **the total dose required for that stage** will be included in the Evaluable Population. This population will be used for the ~~primary~~ **efficacy** analysis
</td></tr>
<tr><td>Section 10.5.1 Analysis Timepoints
(PRMC/IRB)</td><td>

<u>Formerly read:</u>

Data from this study will be assessed at 4 timepoints:

- Memo to file: after 6 subjects in Stage 1 finish PRV111treatment, response and DLTs will be assessed to determine Stage 2 dosing.
</td></tr>
</table>

Section	Description of Change
	• Interim report: after 6 subjects in Stage 1 finish all observations up to 6 months, safety data will be analyzed.<br>• Interim report: after all subjects in Stage 2 finish PRV111treatment, efficacy, PK, and technical data will be analyzed. Subjects will continue to be followed for safety, so this report will have whatever safety data exist at this point in time.<br>• Final report: after all subjects complete all observations.<br><br>***<u>Now reads:</u>***<br>Data from this study will be ~~assessed~~ **documented** at ~~4~~ **2** timepoints:<br>~~• Memo to file: after 6 subjects in Stage 1 finish PRV111treatment, response and DLTs will be assessed to determine Stage 2 dosing.~~<br>~~• Interim report: after 6 subjects in Stage 1 finish all observations up to 6 months, safety data will be analyzed.~~<br>• ~~Interim report: after all subjects in~~ **Final report at End of** Stage 2~~ finish~~**: after all subjects complete** PRV111 treatment, efficacy, PK, and technical data will be analyzed~~.Subjects will continue to be followed for~~**, including all available safety data from the long-term follow-up.**
**Section 10.5.2**<br>**(PRMC/IRB)**	***<u>Updated heading - formerly read:</u>***<br>10.4.2 Subject Accountability, Demographics, and Baseline Characteristics<br><br>***<u>Now reads:</u>***<br>**10.5.2 Long term safety report: after all subjects complete all observations.** Subject Accountability, Demographics, and Baseline Characteristics
**Section 10.5.3 Primary Endpoints**<br>**(PRMC/IRB)**	***<u>Formerly read:</u>***<br>The primary objective is the determination of systemic safety and local tolerability. Therefore, the primary endpoint is incidence of adverse events.<br><br>***<u>Now reads:</u>***<br>The primary ~~objective is~~ **endpoints for** the **study involves** determination of ~~systemic safety~~ **a safe** and ~~local tolerability~~**efficacious dose of PRV111 (Cisplatin Transmucosal System) (cisplatin drug-loaded patch + permeation enhancer [PE]) under the conditions of the trial.**<br>Therefore, the primary endpoint is **based on the** incidence of adverse events **and tumor response.**
**Section 10.5.4 Secondary Endpoints**<br>**(PRMC/IRB)**	***<u>Formerly read:</u>***<br>Based on secondary objectives, the secondary endpoints are:<br>• Tumor response:<br>o RECIST clinical assessment (CR, PR, SD, or PD)<br>o Caliper measurements of tumor volume<br>o CT or MRI of the head and neck<br>o Histology at the time of surgical excision<br>o Loco-regional control: subjects will be followed-up for disease recurrence and safety following surgery<br>• Blood samples for plasma levels of total platinum

<table>
<tr><th>Section</th><th>Description of Change</th></tr>
<tr><td></td><td>
Technical success:
Successful adhesion of the patch to the target lesion
Successful delivery (including release and permeation) of the drug to the target lesion, without getting to surrounding oral surfaces
Successful peel of the used patch after 10 minutes without damaging the target lesion underneath
Adhesion of the backing to the polymeric matrix embedded CLPs
Taste of the PE solution
Taste of the PRV111 patch
Residual cisplatin level in the patch after the application period
<u>Now reads:</u>
~~Tumor response:~~
~~RECIST clinical assessment (CR, PR, SD, or PD)~~
~~Caliper~~ Evaluate tumor volume shrinkage
Ruler measurements of tumor volume
CT or MRI of the head and neck
Histology at the time of surgical excision
Assess short and long-term safety of the treatment
Loco-regional control: subjects will be followed-up for disease recurrence and safety following surgery
~~Blood samples for plasma levels~~ Incidence of ~~total~~ recurrence at 6 months
Total platinum assessment in blood, tumor and lymph nodes (if available)
Technical success
Successful adhesion of the patch to the target lesion
Successful delivery (including release and permeation) of the drug to the target lesion, without getting to surrounding oral surfaces
Successful peel of the used patch after 10 minutes without damaging the target lesion underneath
Adhesion of the backing to the polymeric matrix embedded CLPs
Taste of the PE solution
Taste of the PRV111 patch
Residual cisplatin level in the patch after the application period
</td></tr>
<tr><td>Section 10.5.7 Pharmacokinetics Data
(Correction)</td><td><u>Formerly read:</u>
PK parameters will include renal clearance (CLr), AUC, C_{max}, C_{min}, T_{max}, and $t_{1/2}$

<u>Now reads:</u>
PK parameters will include ~~renal clearance (CLr),~~ AUC, C_{max}, C_{min}, T_{max}, and $t_{1/2}$</td></tr>
<tr><td>Section 12.1 Study Monitoring (6th bullet)
(Correction)</td><td><u>Formerly read:</u>
Concomitant therapy use</td></tr>
</table>

Section	Description of Change
	Now reads:<br>• Concomitant therapy uses
**Section 13.5 Protocol Deviations (2nd paragraph)**<br><br>**(Correction)**	***Formerly read:***<br>In the event of an important deviation, a report will be submitted to the IRB (as applicable) and Sponsor explaining the circumstances.<br><br>***Now reads:***<br>In the event of an important deviation, a report will be submitted to the IRB (as applicable) and ~~Sponsor~~ **site** explaining the circumstances.

Table 1 Schedule of Events formerly read:

	**Screening Within 28 Days Prior to Visit 1**	**Days**						**Months Post Surgery**
		**Treatment Visits**				**Post Treatment[g] Pre-Op**	**Surgery**	**SOC Follow-up**
		**Visit 1**	**Visit 2**	**Visit 3**	**Visit 4**	**Visit 5**	**Visit 6**	**Visits 7, 8, 9**
	**0**	**1**	**≥48 and ≤96 hours, post previous visit**			**≥72 hours post last treatment**	**≤21 days post Visit 1**	**1, 3, & 6±3 Weeks**
Confirmed SCC	X							
Informed consent	X							
Inclusion/exclusion criteria	X	X[d]						
Schedule study visits	X	X	X	X	X	X		
Demographics, medical/ medication history	X							
Physical examination, VS[a]	X	X[d]	X[d]	X[d]	X[d]	X		X
Anti-emetics		X[d]	X[d]	X[d]	X[d]			
CT chest	X							
Oral cavity examination[i]	X	X[d]	X[d]	X[d]	X[d]	X		X
Neck lymph nodes exam	X	X[d]	X[d]	X[d]	X[d]	X		X
Electrocardiogram	X				X[d]			
CT or MRI head and neck	X					X		
Clinical labs[b,j]	X	X[d]		X		X		X
Pregnancy test[c]	X	X[d]						
PK sampling		X[e]	X[f]	X[f]	X[e]			
Tumor resection							X[h]	
PRV111 Patch treatment		X	X	X	X			
Tumor measurement		X[d]	X	X	X[d]	X		
RECIST criteria			X[d]	X[d]	X[d]	X		
Tumor photographs		X[d]	X[d]	X[d]	X[d]	X		X

	Screening Within 28 Days Prior to Visit 1	Days					Surgery	Months Post Surgery
		Treatment Visits				Post Treatment[g] Pre-Op		SOC Follow-up
		Visit 1	Visit 2	Visit 3	Visit 4	Visit 5	Visit 6	Visits 7, 8, 9
	0	1	≥48 and ≤96 hours, post previous visit			≥72 hours post last treatment	≤21 days post Visit 1	1, 3, & 6±3 Weeks
Loco-regional recurrence								X
Histology	X						X	
Technical success		X	X	X	X			
Taste of patch and PE solution		X	X	X	X			
Concomitant therapy	X	X	X	X	X	X		X
AE assessment	X	X	X	X	X	X		X

Abbreviations: AE=adverse event; CT=computed tomography; MRI= magnetic resonance imaging; PE=permeation enhancer; PK=pharmacokinetics; RECIST=Response Evaluation Criteria in Solid Tumors; SCC=squamous cell carcinoma; SOC=standard of care; VS=vital signs.

[a] Vital signs - seated blood pressure and pulse will be measured after the subject has been sitting for at least 3 minutes, respiration, temperature, height and weight will be recorded at every visit. Vital signs will be obtained pre-dose and subject will also be monitored for 30±5 minutes, 60±5 minutes and 120 ±5 minutes following application of the last patch applied at that treatment visit.

[b] Complete blood count and chemistry panel (creatinine, blood urea nitrogen, liver function test, electrolytes (potassium, magnesium, calcium, and sodium), urinalysis.

[c] Serum pregnancy test to establish eligibility for female subjects of childbearing potential. Urine pregnancy test will be collected on Day 1 pre-treatment.

[d] Performed prior to dosing.

[e] PK blood samples for total platinum collected pre- treatment (time -15 to 0 minutes), and 30±5 minutes, 60±5 minutes and 120±5 minutes post removal of last patch.

[f] PK blood samples for total platinum will be collected pre-treatment (time -15 to 0 minutes), and 30±5 minutes post the removal of the last patch.

[g] To be completed at least 72 hours after the last treatment and prior to surgery. The surgery is to be performed no later than 21 days post Visit 1.

[h] Sampling for platinum levels in tumor tissue.

[i] Mark the borders of the tumor for further assessment of tumor shrinkage

[j] Human papilloma virus will be ascertained using immunohistochemistry for p16 overexpression per institutional standards.

Table 1 Schedule of Events now reads:

	Screening Within 28 Days Prior to Visit 1	Days						Months Post Surgery
		Treatment Visits				Post Treatment Pre-Op	Surgery	Standard of Care Follow-up
		Visit 1	Visit 2	Visit 3	Visit 4	Visit 5	Visit 6	Visits 7, 8, 9
	0	1	≥48 and ≤96 hours, post previous visit			≥72 hours post last treatment	≤21 days post Visit 1	1, 3, & 6±3 Weeks
Confirmed SCC	X							
Informed consent	X							
Inclusion/exclusion criteria	X	X[a]						
Schedule study visits	X	X	X	X	X	X		
Demographics, medical/ medication history	X							
Physical examination[b], VS[c]	X	X[a]	X[a]	X[a]	X[a]	X		X
Anti-emetics		X[a]	X[a]	X[a]	X[a]			
CT chest	X							
Oral cavity examination[d]	X	X[a]	X[a]	X[a]	X[a]	X		X
Neck lymph nodes exam	X	X[a]	X[a]	X[a]	X[a]	X		X
Electrocardiogram	X					X		
CT or MRI head and neck[e]	X					X		
Clinical labs[f]	X	X[a]		X[a]		X		X
HPV	X[g]							
Pregnancy test	X[h]	X[a]						
PK sampling		X[i]	X[i]	X[i]	X[i]			
Tumor resection							X[j]	

		**Days**						**Months Post Surgery**
	**Screening Within 28 Days Prior to Visit 1**	**Treatment Visits**				**Post Treatment Pre-Op**	**Surgery**	**Standard of Care Follow-up**
		**Visit 1**	**Visit 2**	**Visit 3**	**Visit 4**	**Visit 5**	**Visit 6**	**Visits 7, 8, 9**
	**0**	**1**	**≥48 and ≤96 hours, post previous visit**			**≥72 hours post last treatment**	**≤21 days post Visit 1**	**1, 3, & 6±3 Weeks**
PRV111 Patch treatment		X	X	X	X			
Tumor measurement		X[a]	X[a]	X[a]	X[a]	X		
RECIST criteria			X[a]	X[a]	X[a]	X		
Tumor photographs		X[k]	X[k]	X[k]	X[k]	X		X
Loco-regional recurrence								X
Histology	X[l]						X[j]	
Technical success[m]		X	X	X	X			
Concomitant therapy	X	X	X	X	X	X		X
AE assessment	X	X	X	X	X	X		X

Abbreviations: AE=adverse event; CT=computed tomography; HPV=human papilloma virus; MRI=magnetic resonance imaging; PE=permeation enhancer; PK=pharmacokinetics; RECIST=Response Evaluation Criteria in Solid Tumors; SCC=squamous cell carcinoma; VS=vital signs.

a. Performed prior to dosing.
b. If a physical exam was performed within 14 days prior to the screening visit, it does not need to be repeated at the screening.
c. VS: seated blood pressure and pulse will be measured after the subject has been sitting for at least 3 minutes, respiration, temperature, height and weight will be recorded at every visit. Vital signs will be obtained pre-dose and subject will also be monitored for 30±5 minutes, 60±5 minutes and 120 ±5 minutes following application of the last patch applied at that treatment visit.
d. Take photos of the tumor region for further assessment of tumor shrinkage.
e. Every attempt should be made to use the same test for each subject for both timepoints.
f. Complete blood count and chemistry panel (creatinine to determine the creatinine clearance, blood urea nitrogen, liver function test, electrolytes (potassium, magnesium, calcium, and sodium), urinalysis.
g. HPV status will be ascertained using immunohistochemistry for p16 according to institutional clinical protocols
h. Serum pregnancy test to establish eligibility for female subjects of childbearing potential. a negative urine pregnancy test must be documented within 7 days prior to the first treatment visit, and at Visit 5.
i. PK blood samples for total platinum collected pre- treatment (time -15 to 0 minutes), and 30±5 minutes, 60±5 minutes and 120±5 minutes post removal of last patch.
j. Sampling for tumor and lymph nodes (if available) platinum levels.
k. Photos need to be taken prior to patch application.
l. Confirmation of tumor histology by a clinical pathologist is required prior to enrollment. A final pathology report, issued by a clinical pathologist is required following surgical resection of the tumor specimen.

Taste of the PE solution and patch, adhesion, peel-off, residual cisplatin level in the patch.

17 APPENDIX 3: AMENDMENT 3

The purpose of Amendment 3, dated 26 March 2019, is to address clinical feedback and observations obtained from the field. The requested revisions include:

a) FDA-agreed primary response change:

- On 27 February 2019, the FDA accepted the use of volumetric tumor measurement as the primary method of measurement and RECIST 1.1 with CT scans and pathology results as secondary methods in the ongoing CLN-001 study. Therefore, tumor response was modified from RECIST to Tumor Volume Change from Baseline (Tumor Volume CFB).

b) Physician-requested revisions:

- Open the PRV111 treatment window to lessen the burden on patients and clinical staff. Definitive curative therapy (Surgery) is **not** delayed or modified due to this change.
- Flexibility on follow-up visit times based on physician discretion and standard of care. Each patient will still be followed for 6 months.
- Other minor changes based on physician feedback, all of which are detailed in the table below.

Changes to the 11 May 2018 Protocol

New text presented in **Bold** typeface; deleted text presented with a ~~Strikethrough~~

Section	Description of Change
**General Revisions**	Version number changed from ~~7.0~~ to **8.0**
	Date of Amendment 3
**Medical Monitor**	***Formerly read:***<br>Alexandre Valentin, MD, MS<br>***Now reads:***<br>~~Alexandre Valentin, MD, MS~~ **Jesse Hall, MD**
**Synopsis; Section 6.1**<br>(Primary Response change from RECIST to using tumor volume)	***Formerly read:***<br><br>1a. If fewer than or equal to 2 of the evaluable subjects have a response (RECIST) criteria complete response [CR] or partial response [PR]) the dose will be escalated from 3 to 5 treatment applications per treatment visit for Stage 2.<br><br>1b. If 3 or more evaluable subjects have a response RECIST criteria CR or PR, the dose will remain the same for Stage 2 (3 applications per treatment visit).<br><br>2a. If there are 6 evaluable subjects in Stage 1:<br>- If out of 6 evaluable subjects, 2 or more subjects have a RECIST criteria CR or PR), the dose will be de-escalated to 2 applications per treatment visit.<br>- If out of 6 evaluable subjects, fewer than 2 have a response (RECIST criteria CR or PR), the dose will be considered and the study will be stopped.

Section	Description of Change
	Now reads:<br><br>1a. If fewer than or equal to 2 of the evaluable subjects have a response ~~(RECIST) criteria~~ **Tumor volume change from baseline (CFB) expressed as percent change from baseline (PCFB)** complete response [CR], **strong response [SR]** or partial response [PR]) the dose will be escalated from 3 to 5 treatment applications per treatment visit for Stage 2.<br><br>1b. If 3 or more evaluable subjects have a response (~~RECIST criteria CR or~~ ~~**PR**~~ **as tumor volume PCFB complete response [CR], strong response [SR] or partial response [PR])**, the dose will remain the same for Stage 2 (3 applications per treatment visit).<br><br>2a. If there are 6 evaluable subjects in Stage 1:<br>- If out of 6 evaluable subjects, 2 or more subjects have **response** **(**~~RECIST criteria CR or PR~~ **as tumor volume PCFB complete response [CR], strong response [SR] or partial response [PR])** the dose will be de-escalated to 2 applications per treatment visit.<br>- If out of 6 evaluable subjects, fewer than 2 have a response (**as tumor volume PCFB complete response [CR], strong response [SR] or partial response [PR]** ~~RECIST criteria CR or PR~~), the dose will be considered **ineffective** and the study will be stopped.
**Synopsis: Study design**<br>(Clarification & Opening up the treatment window)	***Formerly read:***<br>***Note:*** The surgery is to be performed no later than 21 days post Visit 1.<br>All subjects will be followed for up to 6 months post-surgery at 3 visits: 1, 3, and 6 months post-surgery for loco-regional response and safety<br><br>***Now reads:***<br>***Note:*** ~~The surgery is to be performed no later than .21 days post Visit 1.~~<br>***Note:*** **The PRV111 treatment should not cause a delay to the scheduled surgery. Visit 1 can be performed within 42 days prior to the scheduled surgery.**<br>All subjects will be followed for up to 6 months post-surgery at 3 visits: 1 **month**, 3 **months**, and 6 months **or standard of care (discretion of the investigator)** post-surgery for loco-regional response and safety.
**Synopsis; Inclusion criteria**<br>(Clarification #2)<br>(#4 opening the treatment window)	***Formerly read:***<br>2. Pathologically confirmed T1 (<2 cm) or T2 (>2 cm but ≤4 cm)<br>4. Tumor must be amenable to surgical resection no later than 21 days post Visit 1.<br><br>***Now reads:***<br>2. Pathologically **proven and clinically** confirmed T1 (<2 cm) or T2 (>2 cm but ≤4 cm)<br>4. Tumor must be amenable to surgical resection no later than ~~21~~ **42** days post Visit 1.
**Synopsis**	***Formerly read:***

Section	Description of Change
(Clarification)	• [redacted]<br><br>***Now reads:***<br><br>• PE solution [redacted] to be applied superficially to the malignant tumor **to improve the drug permeation into the tumor (1 mL at each treatment visit) (Refer to the** Pharmacy Manual)**.**
**Synopsis**<br>(Clarification & Opening up the treatment window)	***Formerly read:***<br>Duration of Treatment: Up to 3 weeks of treatment prior to the scheduled resection of the tumor and 6 months post-surgical follow-up.<br>***Now reads:***<br>Up to ~~3~~ **6** weeks of treatment prior to the scheduled resection of the tumor and 6 months post-surgical follow-up. ***Note:*** **The PRV111 treatment should not cause a delay to the scheduled surgery. Visit 1 can be performed within 42 days prior to the scheduled surgery.**
**Synopsis**<br>(Primary Response change from RECIST to using tumor volume)	***Formerly read:***<br>• RECIST clinical assessment (CR, PR, SD, or PD). Response defined as CR or PR; non-response defined as SD or PD.<br>• Ruler measurements of tumor volume<br><br>***Now reads:***<br>• **Tumor volume change from baseline** clinical assessment (CR, PR, **SR**, SD, or PD). Response defined as CR, **SR**, or PR; non-response defined as SD or PD.<br>• Ruler **or caliper** measurements of tumor volume
**Synopsis**<br>(Primary Response change from RECIST to using tumor volume)	***Formerly read:***<br>• Efficacy based on RECIST criteria will be summarized, and the null hypothesis that the probability of response is at most 30%, where a response is the occurrence of CR or PR will be tested using a 1-sided test exact binomial test at level 0.06.<br><br>***Now reads:***<br>• Efficacy based on ~~RECIST~~ **tumor volume CFB** criteria will be summarized, and the null hypothesis that the probability of response is at most 30%, where a response is the occurrence of CR, **SR** or PR will be tested using a 1-sided test exact binomial test at level 0.06.
**List of abbreviations**	***Inserted:***<br>**CFB** **Change from Baseline**<br>**PCFB** **Percent change from baseline**
**Section 6.1**	***Formerly read:***

<table>
<tr><th>Section</th><th>Description of Change</th></tr>
<tr><td>(Clarification & Opening up the treatment window)</td><td>
The 4 treatment visits will be separated by 2 to 4 days (at least 48 hours).
Note: The surgery is to be performed no later than 21 days post Visit 1.
All subjects will be followed for up to 6 months post-surgery at 3 visits: 1, 3, and 6 months post-surgery for loco-regional response and safety.
<u>Now reads:</u>
The 4 treatment visits will be separated by 2 to <s>4</s> 6 days (at least 48 hours apart).
Note: The surgery is to be performed no later than <s>21</s> 42 days post Visit 1.
All subjects will be followed for up to 6 months post-surgery at 3 visits: 1 month, 3 months, and 6 months post-surgery for loco-regional response and safety. Follow-up visits may coincide with standard of care follow-up visits, if deemed appropriate by the Principal Investigator.
</td></tr>
<tr><td>Section 8.4
(Opening the treatment window as shown before)</td><td>
<u>Formerly read:</u>
Such interruption may not last more than 96 hours and may not occur more than 1 time during the subject’s treatment schedule.
Subjects who experience an AE(s) requiring interruption of study drug for ≥96 hours will be removed from treatment.
<u>Now reads:</u>
Such interruption may not last more than <s>96</s> 144 hours and may not occur more than 1 time during the subject’s treatment schedule.
Subjects who experience an AE(s) requiring interruption of study drug for ≥<s>96</s> 144 hours will be removed from treatment.
</td></tr>
<tr><td>Section 9.1.1
(Clarification)</td><td>
<u>Formerly read:</u>
Subjects eligible for enrollment must have histological confirmation of SCC. HPV, the possible etiology of SCC, will be ascertained using immunohistochemistry for p16 overexpression per institutional standards
<u>Now reads:</u>
Subjects eligible for enrollment must have histological confirmation of SCC. HPV, the possible etiology of SCC, will be ascertained using immunohistochemistry for p16 overexpression per institutional standards. Results are not an eligibility requirement, but should be available at some point during the subject’s participation in the study.
</td></tr>
<tr><td>Section 9.2.2
(Changed the primary response from RECIST to tumor volume/)</td><td>
<u>Formerly read:</u>
The terms “response” and “non-response” will be defined according to the RECIST criteria
<u>Response</u> will be defined as CR, PR or. <u>Non-Response</u> will be defined as SD or PD (Table 4).
Table 7. Clinical Response to Treatment
<table>
<tr><td>Complete Response (CR)</td><td>Disappearance of primary lesion.</td></tr>
</table>
</td></tr>
</table>

Partial Response (PR)	At least a 30% decrease in the sum of diameters of primary lesion, taking as reference the baseline sum diameters.
Progressive Disease (PD)	At least a 20% increase in the sum of diameters of primary lesion, taking as reference the smallest sum since the treatment started (this includes the baseline sum if that is the smallest on study). In addition to the relative increase of 20%, the sum must also demonstrate an absolute increase of at least 5 mm. (Note: the appearance of one or more new lesions is also considered progression).
Stable Disease (SD)	Neither sufficient shrinkage to qualify for PR nor sufficient increase to qualify for PD, taking as reference the smallest sum diameters while on study.

Now reads:

The terms “response” and “non-response” will be defined according to the ~~RECIST~~ **tumor volume change from baseline (CFB)** criteria

PCFB = % tumor volume change from the baseline

Response will be defined as CR, PR or **SR**. Non-Response will be defined as SD or PD (Table 4).

Table 8. Clinical Response to Treatment **Based on Tumor Volume Change***

Complete Response (CR)	Disappearance of primary lesion.
Partial Response (PR)	~~At least a 30% decrease in the sum of diameters of primary lesion, taking as reference the baseline sum diameters.~~<br>**PCFB: ≥30% to 69% decrease in the total tumor volume shrinkage (as determined by clinical measurement, taking as reference the baseline volume.**
**Strong Response (SR)**	**PCFB: ≥70% to 99% decrease in the total tumor volume shrinkage (as determined by clinical measurement, taking as reference the baseline volume.**
Progressive Disease (PD)	~~At least a 20% increase in the sum of diameters of primary lesion, taking as reference the smallest sum since the treatment started (this includes the baseline sum if that is the smallest on study). In addition to the relative increase of 20%, the sum must also demonstrate an absolute increase of at least 5 mm. (Note: the appearance of one or more new lesions is also considered progression).~~<br>**PCFB: At least a 20% increase tumor volume as determined by clinical measurement taking as reference the baseline volume (Note: the appearance of one or more new lesions is also considered progression).**
Stable Disease (SD)	Neither sufficient shrinkage to qualify for PR nor sufficient increase to qualify for PD, taking as reference the **volume determined by clinical measurement at baseline** ~~smallest sum diameters while on study~~.

Section	Description of Change
	*** Necrosis/inflammation will not be included towards clinical tumor measurements.**
**Section 9.3.1**	***<u>Inserted:</u>***<br>**Neuropathy and ototoxicity will be assessed via clinical examination since cisplatin can potentially cause neurotoxicity and ototoxicity. Questions will be asked to document symptoms such as tinnitus, pain in the extremities, or loss of sensation in the eCRFs.**
**Section 9.6.2**<br>**Section 9.6.3**<br>**Section 9.6.4**<br>**Section 9.6.5**<br>**Section 9.6.6**<br>(Clarification)	***<u>Formerly read:</u>***<br>• VS shall be monitored at 30±5 minutes, 60±5 minutes and 120 ±5 minutes.<br>• Perform and document clinical tumor measurement and RECIST criteria<br>***<u>Now reads:</u>***<br>• VS shall be monitored at 30±5 minutes, 60±5 minutes and 120 ±5 minutes **following application of the last patch applied.**<br>• Perform and document clinical tumor measurement and ~~RECIST~~ **Tumor volume CFB** criteria
**Section 9.6.5**<br>(Clarification)	***<u>Inserted: Pre-Dose Assessments</u>***<br>hCG [urine] for females of childbearing potential
**Section 9.6.7**<br>(Investigator request)	***<u>Inserted in Surgery Day (Visit 6)</u>***<br>• Post-Surgery, collect clinical labs. Draw blood for CBC, chemistry panel (creatinine, BUN, LFT, electrolytes [potassium, magnesium, calcium, and sodium], and perform urinalysis. Post-surgical labs may be used, if deemed appropriate by the Principal Investigator
**Section 9.6.8**<br>(Investigator request)	**Post-Treatment Follow-Up (Visits 7, 8, & 9)**<br>***Formerly read:***<br>• Collect clinical labs blood draw for CBC, chemistry panel (creatinine, BUN, LFT, electrolytes [potassium, magnesium, calcium, and sodium], and perform urinalysis.<br>***<u>Now reads:</u>***<br>• ~~Collect clinical labs blood draw for CBC, chemistry panel (creatinine, BUN, LFT, electrolytes [potassium, magnesium, calcium, and sodium], and perform urinalysis.~~
**Section 10.2**<br>(changed primary tumor response from RECIST to tumor volume)	***<u>Formerly read:</u>***<br>• Efficacy based on RECIST criteria will be summarized, and the null hypothesis that the probability of response is at most 30%, where a response is the occurrence of CR or PR will be tested using a one-sided test exact binomial test at level 0.06.<br>***<u>Now reads:</u>***<br>• Efficacy based on ~~RECIST~~ **Tumor volume CFB** criteria will be summarized, and the null hypothesis that the probability of response is at most 30%, where a response is the occurrence of CR or PR will be tested using a one-sided test exact binomial test at level 0.06.

Section	Description of Change
**Section 10.4.2**<br>(clarification)	***Formerly read:***<br><br>All subjects who have completed at least 75% of the total dose required for that stage will be included in the Evaluable Population. This population will be used for the efficacy analysis.<br><br>***Now reads:***<br><br>All subjects who have **met the inclusion and exclusion criteria, and** have completed at least 75% of the total dose required for that stage will be included in the Evaluable Population. This population will be used for the efficacy analysis.
**Section 10.5.4**<br>(changed primary tumor response from RECIST to tumor volume)	***Formerly read:***<br><br>Based on secondary objectives, the secondary endpoints are:<br>• Evaluate tumor volume shrinkage<br>o Ruler measurements of tumor volume<br>o CT or MRI of the head and neck<br>***Now reads:***<br><br>Based on secondary objectives, the secondary endpoints are:<br>• Evaluate tumor ~~volume shrinkage~~ **response**<br>~~o Ruler measurements of tumor volume~~<br>o CT or MRI of the head and neck **based on RECIST 1.1**
**Section 10.5.5**<br>(changed primary tumor response from RECIST to tumor volume)	***Formerly read:***<br>Efficacy outcomes include:<br>• CT Scan/MRI<br>• Incidence of recurrence at 6 months<br>• Histological effect of PRV111 on the tumor tissue<br>***Now reads:***<br>Efficacy outcomes include:<br>• **Response to treatment evaluated by Tumor volume change from baseline (CFB)**<br>• CT Scan/MRI **RECIST 1.1**<br>• Incidence of recurrence at 6 months<br>• Histological effect of PRV111 on the tumor tissue

Formerly Read:

Table 1. Schedule of events

	Screening Within 28 Days Prior to Visit 1	Days					Surgery	Months Post Surgery
		Treatment Visits				Post Treatment Pre-Op		Standard of Care Follow-up
		Visit 1	Visit 2	Visit 3	Visit 4	Visit 5	Visit 6	Visits 7, 8, 9
	0	1	≥48 and ≤96 hours, post previous visit			≥72 hours post last treatment	≤42 days post Visit 1	1, 3, & 6±3 Weeks
Confirmed SCC	X							
Informed consent	X							
Inclusion/exclusion criteria	X	X[a]						
Schedule study visits	X	X	X	X	X	X		
Demographics, medical/ medication history	X							
Physical examination,[b] VS[c]	X	X[a]	X[a]	X[a]	X[a]	X		X
Anti-emetics		X[a]	X[a]	X[a]	X[a]			
CT chest	X							
Oral cavity examination[d]	X	X[a]	X[a]	X[a]	X[a]	X		X
Neck lymph nodes exam	X	X[a]	X[a]	X[a]	X[a]	X		X
ECG	X					X		
CT or MRI head and neck[e]	X					X		
Clinical labs[f]	X	X[a]		X[a]		X		X
HPV	X[g]							
Pregnancy test	X[h]	X[a]			X			

	Screening Within 28 Days Prior to Visit 1	Days					Surgery	Months Post Surgery
		Treatment Visits				Post Treatment Pre-Op		Standard of Care Follow-up
		Visit 1	Visit 2	Visit 3	Visit 4	Visit 5	Visit 6	Visits 7, 8, 9
	0	1	≥48 and ≤96 hours, post previous visit			≥72 hours post last treatment	≤42 days post Visit 1	1, 3, & 6±3 Weeks
PK sampling		X[i]	X[j]	X[j]	X[i]			
Tumor resection							X[k]	
PRV111 patch treatment		X	X	X	X			
Tumor measurement		X[a]	X[a]	X[a]	X[a]	X		
Tumor Volume change from baseline (CFB) criteria			X[a]	X[a]	X[a]	X		
Tumor photographs		X[l]	X[l]	X[l]	X[l]	X		X
Loco-regional recurrence								X
Histology	X[m]						X[m]	
Technical success[n]		X	X	X	X			
Concomitant therapy	X	X	X	X	X	X		X
AE assessment	X	X	X	X	X	X		X

Abbreviations: AE=adverse event; CT=computed tomography; ECG=electrocardiogram; HPV=human papilloma virus; MRI=magnetic resonance imaging; PE=permeation enhancer; PK=pharmacokinetics; RECIST=Response Evaluation Criteria in Solid Tumors; SCC=squamous cell carcinoma; VS=vital signs.

a Performed prior to dosing.

b If a physical exam was performed within 14 days prior to the screening visit, it does not need to be repeated at the screening.

c VS: seated blood pressure and pulse will be measured after the subject has been sitting for at least 3 minutes, respiration, temperature, height and weight will be recorded at every visit. VS will be obtained pre-dose at all visits and subject will also be monitored at 30+5 minutes, 60+5 minutes and 120+5 minutes following application of the last patch applied at

d Take photos of the tumor region for further assessment of tumor shrinkage.

e Every attempt should be made to use the same test for each subject for both timepoints.

f CBC and chemistry panel (creatinine to determine the creatinine clearance, BUN, LFT, electrolytes (potassium, magnesium, calcium, and sodium), urinalysis.

g Tissue will be collected at Screening but the HPV status will be ascertained at any time during the study using immunohistochemistry for p16 according to institutional clinical protocols.

h Serum pregnancy test to establish eligibility for female subjects of childbearing potential. A negative urine pregnancy test must be documented within 7 days prior to the first treatment visit, and at Visit 5.

i PK blood samples for total platinum collected pre- treatment (time -15 to 0 minutes), and 30±5 minutes, 60±5 minutes and 120±5 minutes post removal of last patch.k Sampling for tumor and lymph nodes (if available) platinum levels.

l Photos need to be taken prior to patch application.

m Confirmation of tumor histology by a clinical pathologist is required prior to enrollment. A final pathology report, issued by a clinical pathologist is required following surgical resection of the tumor specimen.

n Taste of the PE solution and patch, adhesion, peel-off, residual cisplatin level in the patch.

Now Reads:

Table 1. Schedule of events

		Days						Months Post Surgery
	Screening Within 28 Days Prior to Visit 1	Treatment Visits				Post Treatment Pre-Op	Surgery	Standard of Care **(SOC)** Follow-up
		Visit 1	Visit 2	Visit 3	Visit 4	Visit 5	Visit 6	Visits 7, 8, 9
	**0**	1	≥48 and ≤~~96~~ **144** hours, post previous visit			≥72 hours post last treatment	≤42 days post Visit 1	1, 3, & 6±3 ~~Weeks~~ **months (all can be<u>+ 3</u> weeks) SOC visits**
Confirmed SCC	X							
Informed consent	X							
Inclusion/exclusion criteria	X	X[a]						
Schedule study visits	X	X	X	X	X	X		
Demographics, medical/ ~~medication~~ history	X							
Physical examination,[b] VS[c]	X	X[a]	X[a]	X[a]	X[a]	X		X
Anti-emetics		X[a]	X[a]	X[a]	X[a]			
CT chest	X							
Oral cavity examination[d]	X	X[a]	X[a]	X[a]	X[a]	X		X
Neck lymph nodes exam	X	X[a]	X[a]	X[a]	X[a]	X		X
ECG	X					X		
CT or MRI head and neck[e]	X					X		
Clinical labs[f]	X	X[a]		X[a]		X	X[o]	

	Screening Within 28 Days Prior to Visit 1	Days					Surgery	Months Post Surgery
		Treatment Visits				Post Treatment Pre-Op		Standard of Care **(SOC)** Follow-up
		Visit 1	Visit 2	Visit 3	Visit 4	Visit 5	Visit 6	Visits 7, 8, 9
	**0**	1	≥48 and ≤~~96~~ **144** hours, post previous visit			≥72 hours post last treatment	≤42 days post Visit 1	1, 3, & 6±3 ~~Weeks~~ **months (all can be+ 3 weeks) SOC visits**
HPV	X[g]							
Pregnancy test	X[h]	X[a]			X			
PK sampling		X[i]	X[j]	X[j]	X[i]			
Tumor resection							X[k]	
PRV111 patch treatment		X	X	X	X			
Tumor measurement		X[a]	X[a]	X[a]	X[a]	X		
Tumor Volume change from baseline (CFB) criteria			X[a]	X[a]	X[a]	X		
Tumor photographs		X[l]	X[l]	X[l]	X[l]	X		~~X~~
Loco-regional recurrence								X
Histology	X[m]						X[m]	
Technical success[n]		X	X	X	X			
Concomitant therapy	X	X	X	X	X	X		X
AE assessment	X	X	X	X	X	X		X

Abbreviations: AE=adverse event; CT=computed tomography; ECG=electrocardiogram; HPV=human papilloma virus; MRI=magnetic resonance imaging; PE=permeation enhancer; PK=pharmacokinetics~~; RECIST=Response Evaluation Criteria in Solid Tumors~~; SCC=squamous cell carcinoma; VS=vital signs.

a Performed prior to dosing.
b If a physical exam was performed within 14 days prior to the screening visit, it does not need to be repeated at the screening.
c VS: seated blood pressure and pulse will be measured after the subject has been sitting for at least 3 minutes, respiration, temperature, height and weight will be recorded at every visit. VS will be obtained pre-dose at all visits and subject will also be monitored at 30+5 minutes, 60+5 minutes and 120+5 minutes following application of the last patch applied at **Visits 1 and 4 and at 30+5 minutes post last patch application at Visits 2 and 3.**
d Take photos of the tumor region for further assessment of tumor shrinkage.
e Every attempt should be made to use the same test for each subject for both timepoints.
f CBC and chemistry panel (creatinine to determine the creatinine clearance, BUN, LFT, electrolytes (potassium, magnesium, calcium, and sodium), urinalysis.

g Tissue will be collected at Screening but the HPV status will be ascertained at any time during the study using immunohistochemistry for p16 according to institutional clinical protocols.

h Serum pregnancy test to establish eligibility for female subjects of childbearing potential. A negative urine pregnancy test must be documented within 7 days prior to the first treatment visit, and at Visit 5.

i **Visit 1 & 4:** PK blood samples for total platinum collected pre- treatment (time -15 to 0 minutes), and 30±5 minutes, 60±5 minutes and 120±5 minutes post removal of last patch.

j. **Visit 2 & 3: PK blood samples for total platinum collected pre- treatment (time -15 to 0 minutes), and 30±5 minutes, post removal of last patch.**

k Sampling for tumor and lymph nodes (if available) platinum levels.

l Photos need to be taken prior to patch application.

m Confirmation of tumor histology by a clinical pathologist is required prior to enrollment. A final pathology report, issued by a clinical pathologist is required following surgical resection of the tumor specimen.

n Taste of the PE solution and patch, adhesion, peel-off, residual cisplatin level in the patch.

o Performed after surgery if possible.

18 APPENDIX 4: AMENDMENT 4

The purpose of Amendment 4, dated 24 May 2019, is to address clinical feedback and observations obtained from the field especially unnecessary reporting of AEs due to the tumor resection and reconstructive surgery. The requested revisions include:

Physician-requested revisions:

- All AEs (including SAEs) within the 5 pre-surgery visits are to be accurately recorded on the AE/SAE page(s) of the CRF.
- In addition, any SAEs within 30 days of visit 5 will need to be recorded.
- Other minor changes based on physician feedback, all of which are detailed in the table below.

Changes to the Amendment 3 dated 26 March 2019 Protocol

New text presented in **Bold** typeface; deleted text presented with a ~~Strikethrough~~

Section	Description of Change
**General Revisions**	Removed internal version numbers
	Date of Amendment 4 May 24, 2019
**STUDY CONTACT INFORMATION**	***Formerly read:***<br>Susan Szambelan<br>***Now reads:***<br>Ruthanna Costello
**Study Design**<br>**4.2 Privo's PRV111 Patch**<br>**6.1 Study Design**	***Formerly read:***<br>Each treatment visit will include 1 ml of the PE (see Pharmacy Manual for details).<br>***Now reads:***<br>Each treatment visit will include 1 ml of the PE **which will be applied pre and post patch application** (see Pharmacy Manual for details).
**Synopsis; Secondary Objectives**<br>**5.2 Secondary Objectives**	***Formerly read:***<br>• Evaluate tumor response<br>• Assess the long-term safety of the treatment<br>***Now reads:***<br>**Secondary Objectives**<br>• Evaluate tumor response<br>• Assess the ~~long-term safety~~ **safety and tolerability** of the treatment
**9.2.1 Tumor Photography and Measurements**	***Added:***<br>Note: If tumor or cancerous lesion is flat, a small number (0.05 cm) will be recorded for the Height. If the tumor is a concave, a negative Height is recorded. If the height is an unmeasurable dent, the height can be recorded as (-0.05 cm).

Section	Description of Change
**9.3.6.6 Recording Adverse Events**	***<u>Formerly read:</u>***<br>All AEs (including SAEs) to be accurately recorded on the AE/SAE page(s) of the CRF.<br><br>***<u>Now reads:</u>***<br>All AEs (including SAEs) **within the 5 pre-surgery visits** are to be accurately recorded on the AE/SAE page(s) of the CRF. **In addition, any SAEs within 30 days of visit 5 will need to be recorded.**
**9.3.6.8.1 Events to Be Reported and Timeframe**	***<u>Formerly read:</u>***<br>• Any death or other SAE experienced by the subject during treatment or within 30 days of receiving study drug, regardless of relationship to study drug, or any death that occurs more than 30 days after receiving study drug and is believed to be study drug-related, must be promptly reported to the Sponsor (within 24 hours of the Investigator becoming aware of the event) by telephone or email transmission.<br>***<u>Now reads:</u>***<br>• Any death or other SAE experienced by the subject during treatment or within 30 days of receiving study drug, regardless of **expectedness**, relationship to study drug, or any death that occurs more than 30 days after receiving study drug and is believed to be study drug-related, must be promptly reported to the Sponsor (within 24 hours (**of a working day**) of the Investigator becoming aware of the event) by telephone or email transmission.
**9.6.1Screening (Visit 0)**	***<u>Formerly read:</u>***<br>• Occurs within 28 days prior to initial treatment (Visit 1). Confirmed SCC<br>• Perform a general physical examination and check VS<br>• Perform CT of the chest (with or without contrast)<br>• Perform an ECG<br>• Perform CT or MRI head and neck. For each subject, the same imaging modality should be used at Screening and Visit 5 for consistency.<br>• AE documentation begins when the subject signs the ICF<br>• Test tissue for HPV<br><br>***<u>Now reads:</u>***<br>• **Visit and all historical exams/tests** must occur within 28 days prior to initial treatment (Visit 1). Confirmed SCC<br>• **Obtain or** perform a general physical examination (must be within 14 days of first dose) and check VS Obtain historical or perform CT of the chest (with or without contrast)<br>• **Obtain historical or** perform an ECG<br>• **Obtain historical or** perform CT or MRI head and neck. For each subject, the same imaging modality should be used at Screening and Visit 5 for consistency.<br>• AE documentation begins after the subject signs the ICF<br>• **Obtain historical** or test tissue for HPV

Table 1. Schedule of events

	Screening Within 28 Days Prior to Visit 1	Days					Surgery	Months Post Surgery
		Treatment Visits				Post Treatment Pre-Op		Standard of Care **(SOC)** Follow-up
		Visit 1	Visit 2	Visit 3	Visit 4	Visit 5	Visit 6	Visits 7, 8, 9
	**0**	1	≥48 and ≤ 144 hours, post previous visit			≥72 hours post last treatment	≤42 days post Visit 1	1, 3, & 6±3 months (all can be<u>+ 3</u> weeks) SOC visits
Confirmed SCC	X							
Informed consent	X							
Inclusion/exclusion criteria	X	X[a]						
Schedule study visits	X	X	X	X	X	X		
Demographics, medical/ ~~medication~~ history	X							
Physical examination,[b] VS[c]	X	X[a]	X[a]	X[a]	X[a]	X		X
Anti-emetics		X[a]	X[a]	X[a]	X[a]			
CT chest	X							
Oral cavity examination[d]	X	X[a]	X[a]	X[a]	X[a]	X		X
Neck lymph nodes exam	X	X[a]	X[a]	X[a]	X[a]	X		X
ECG	X					X		
CT or MRI head and neck[e]	X					X		
Clinical labs[f]	X	X[a]		X[a]		X	X[o]	
HPV	X[g]							
Pregnancy test	X[h]	X[a]			X			
PK sampling		X[i]	X[j]	X[j]	X[i]			
Tumor resection							X[k]	

	Screening Within 28 Days Prior to Visit 1	Days					Surgery	Months Post Surgery
		Treatment Visits				Post Treatment Pre-Op		Standard of Care **(SOC)** Follow-up
		Visit 1	Visit 2	Visit 3	Visit 4	Visit 5	Visit 6	Visits 7, 8, 9
	**0**	1	≥48 and ≤ 144 hours, post previous visit			≥72 hours post last treatment	≤42 days post Visit 1	1, 3, & 6±3 months (all can be+ 3 weeks) SOC visits
PRV111 patch treatment		X	X	X	X			
Tumor measurement		X[a]	X[a]	X[a]	X[a]	X		
Tumor Volume change from baseline (CFB) criteria			X[a]	X[a]	X[a]	X		
Tumor photographs		X[l]	X[l]	X[l]	X[l]	X		~~X~~
Loco-regional recurrence								X
Histology	X[m]						X[m]	
Technical success[n]		X	X	X	X			
Concomitant therapy	X	X	X	X	X	X		X
AE assessment	X	X	X	X	X	X		X

Abbreviations: AE=adverse event; CT=computed tomography; ECG=electrocardiogram; HPV=human papilloma virus; MRI=magnetic resonance imaging; PE=permeation enhancer; PK=pharmacokinetics; SCC=squamous cell carcinoma; VS=vital signs.

a Performed prior to dosing.

b If a physical exam was performed within 14 days prior to the screening visit, it does not need to be repeated at the screening.

c VS: seated blood pressure and pulse will be measured after the subject has been sitting for at least 3 minutes, respiration, temperature, height and weight will be recorded at every visit. VS will be obtained pre-dose at all visits and subject will also be monitored at 30+5 minutes, 60+5 minutes and 120+5 minutes following application of the last patch applied at Visits 1 and 4 and at 30+5 minutes post last patch application at Visits 2 and 3.

d Take photos of the tumor region for further assessment of tumor shrinkage.

e Every attempt should be made to use the same test for each subject for both timepoints.

f CBC and chemistry panel (creatinine to determine the creatinine clearance, BUN, LFT, electrolytes (potassium, magnesium, calcium, and sodium), urinalysis.

g Tissue will be collected at Screening but the HPV status will be ascertained at any time during the study using immunohistochemistry for p16 according to institutional clinical protocols.

h Serum pregnancy test to establish eligibility for female subjects of childbearing potential. A negative urine pregnancy test must be documented within 7 days prior to the first treatment visit, and at Visit 5.

i Visit 1 & 4: PK blood samples for total platinum collected pre- treatment (time -15 to 0 minutes), and 30±5 minutes, 60±5 minutes and 120±5 minutes post removal of last patch.

j. Visit 2 & 3: PK blood samples for total platinum collected pre- treatment (time -15 to 0 minutes), and 30±5 minutes, post removal of last patch.
k Sampling for tumor and lymph nodes (if available) platinum levels.
l Photos need to be taken prior to patch application.
m Confirmation of tumor histology by a clinical pathologist is required prior to enrollment. A final pathology report, issued by a clinical pathologist is required following surgical resection of the tumor specimen.
n Taste of the PE solution and patch, adhesion, peel-off, residual cisplatin level in the patch.
o Performed after surgery if possible.

<u>Now Reads:</u>

	Screening Within 28 Days Prior to Visit 1	Days					Surgery	Months Post Surgery
		Treatment Visits				Post Treatment Pre-Op		Standard of Care (SOC) Follow-up
		Visit 1	Visit 2	Visit 3	Visit 4	Visit 5	Visit 6	Visits 7, 8, 9
	0	1	≥48 and ≤ 144 hours, post previous visit			≥72 hours post last treatment	≤42 days post Visit 1	1, 3, & 6 months (all can be <u>+/-3</u> weeks) SOC visits
Confirmed SCC	X							
Informed consent	X							
Inclusion/exclusion criteria	X	X[a]						
Schedule study visits	X	X	X	X	X	X		
Demographic, medical/history	X							
Physical examination,[b,s] VS[c]	X	X[a]	X[a]	X[a]	X[a]	X		X
Anti-emetics		X[a]	X[a]	X[a]	X[a]			
CT chest [s]	X							
Oral cavity examination[d]	X	X[a]	X[a]	X[a]	X[a]	X		X
Neck lymph nodes exam	X	X[a]	X[a]	X[a]	X[a]	X		X
ECG [s]	X					X		
CT or MRI head and neck[e, s]	X					X		

	**Screening Within 28 Days Prior to Visit 1**	**Days**					**Surgery**	**Months Post Surgery**
		**Treatment Visits**				**Post Treatment Pre-Op**		**Standard of Care (SOC) Follow-up**
		**Visit 1**	**Visit 2**	**Visit 3**	**Visit 4**	**Visit 5**	**Visit 6**	**Visits 7, 8, 9**
	**0**	**1**	**≥48 and ≤ 144 hours, post previous visit**			**≥72 hours post last treatment**	**≤42 days post Visit 1**	**1, 3, & 6 months (all can be +/-3 weeks) SOC visits**
Clinical labs[f]	X	X[a]		X[a]		X	X[o]	
HPV [s]	X[g]							
Pregnancy test	X[h]	X[a]			X			
PK sampling		X[i]	X[j]	X[j]	X[i]			
Tumor resection							X[k]	
PRV111 patch treatment		X[p]	X[p]	X[p]	X[p]			
Tumor measurements[q]		X[a]	X[a]	X[a]	X[a]	X		
Tumor Volume change from baseline (CFB) criteria			X[a]	X[a]	X[a]	X		
Tumor photographs		X[l]	X[l]	X[l]	X[l]	X		
Loco-regional recurrence								X
Histology[s]	X[m]						X[m]	
Technical success[n]		X	X	X	X			
Concomitant therapy	X	X	X	X	X	X		X
AE assessment	X	X	X	X	X	X[r]		

Abbreviations: AE=adverse event; CT=computed tomography; ECG=electrocardiogram; HPV=human papilloma virus; MRI=magnetic resonance imaging; PE=permeation enhancer; PK=pharmacokinetics; SCC=squamous cell carcinoma; VS=vital signs.

a Performed prior to dosing.
b If a physical exam was performed within 14 days prior to the screening visit, it does not need to be repeated at the screening.
c VS: seated blood pressure and pulse will be measured after the subject has been sitting for at least 3 minutes, respiration, temperature, height and weight will be recorded at every visit. VS will be obtained pre-dose at all visits and subject will also be monitored at 30+5 minutes, 60+5 minutes and 120+5 minutes following application of the last patch applied at Visits 1 and 4 and at 30+5 minutes post last patch application at Visits 2 and 3.
d Take photos of the tumor region for further assessment of tumor shrinkage.
e Every attempt should be made to use the same test for each subject for both timepoints.
f CBC and chemistry panel (creatinine to determine the creatinine clearance, BUN, LFT, electrolytes (potassium, magnesium, calcium, and sodium), urinalysis.

g Tissue will be collected at Screening but the HPV status will be ascertained at any time during the study using immunohistochemistry for p16 according to institutional clinical protocols.

h Serum pregnancy test to establish eligibility for female subjects of childbearing potential. A negative urine pregnancy test must be documented within 7 days prior to the first treatment visit, and at Visit 5.

i Visit 1 & 4: PK blood samples for total platinum collected pre- treatment (time -15 to 0 minutes), and 30±5 minutes, 60±5 minutes and 120±5 minutes post removal of last patch.

j Visit 2 & 3: PK blood samples for total platinum collected pre- treatment (time -15 to 0 minutes), and 30±5 minutes, post removal of last patch and **PE application**.

k Sampling for tumor and lymph nodes (if available) platinum levels.

l Photos need to be taken prior to patch application.

m Confirmation of tumor histology by a clinical pathologist is required prior to enrollment. A final pathology report, issued by a clinical pathologist is required following surgical resection of the tumor specimen.

n Taste of the PE solution and patch, adhesion

Added:

p PE should be applied to the tumor area prior to first patch and allow to stay for 5 minutes. Then applied again to the tumor area for 1 minute. Note that the mouth should be kept open for the saliva not wash off the PE.

q Use “0.05” cm for the “Height” of a flat tumor. If a concave tumor, the “Height” can be shown as a negative number. If a non-measurable dent, use “-0.05” cm.

r Only SAEs within 30 days post visit 5 need to be recorded.

s. May use historical SOC information prior to consent if collected within 28 days prior to first treatment.

19 APPENDIX 5: AMENDMENT 5

The purpose of Amendment 5, dated 23 March 2020, is as follow:

1) To update statistical sections: Statistical power increased, alpha lowered based on updated clinical response rate
2) Added a section regarding remote monitoring and site closures

New text presented in **Bold** typeface; deleted text presented with a ~~Strikethrough~~

Section	Description of Change
**General Revisions**	Date of Amendment 5 March 23, 2020

Section: ***Statistical power increased, alpha lowered based on updated clinical response rate***

Synopsis – Power and Sample Size

Section 6 – Investigational Plan

Section 10.2 – Sample Size Determination

Description of Change:

Formerly read:

Power and Sample Size

The planned enrollment for the final dose is intended to obtain N=16 evaluable subjects. The planned enrollment for the final dose is intended to recruit N=16 evaluable subjects. In the first stage, 6 evaluable subjects will be accrued. If there are 2 or fewer responses in these 6 subjects, the dose will be escalated. Otherwise, the dose will remain the same or de-escalated based on safety data. Additional subjects will be accrued for upto 16 evaluable subjects at the final dose.

If the dose is not increased after Stage 1, the test will be performed using all evaluable subjects from Stage 1 and 2. If the dose is adjusted after Stage 1, the test will be performed using all evaluable subjects from Stage 2.

Based on Simon's 2-stage procedure, the null hypothesis that the true response rate is 0.30 will be tested against a one-sided alternative. The null hypothesis will be rejected if 8 or more responses are observed at the final dose. This design yields a type I error rate of 0.06 and power of 85.1% when the true response rate is 0.65.

Null response rate: P_0	Alt. response rate: P_1	Alpha one-tailed	Power	n1	r1	N	R
0.30	0.65	0.06	85.1%	6	2	16	7

N *is the total number of evaluable subjects at the final dose*

n*1 is the number of evaluable subjects accrued during Stage 1*

r*1, if* r*1 or fewer responses are observed during stage 1, escalate the dose if safety requirements are met*

R*, if* R *or fewer responses are observed by the end of Stage 2 in N subjects, then an effective dose is not found*

Now reads:

Power and Sample Size

The planned enrollment for the final dose is intended to obtain N=~~16~~ **11** evaluable subjects. The planned enrollment for the final dose is intended to recruit N=~~16~~ **11** evaluable subjects. In the first stage, ~~6~~ **5** evaluable subjects will be accrued. If there are 2 or fewer responses in these ~~6~~ **5** subjects, the dose will be escalated. Otherwise, the dose will remain the same or de-escalated based on safety data. Additional subjects will be accrued for a total of ~~16~~ up to **11** evaluable subjects at the final dose.

If the dose is not increased after Stage 1, the test will be performed using all evaluable subjects from Stage 1 and 2. If the dose is adjusted after Stage 1, the test will be performed using all evaluable subjects from Stage 2.

Based on Simon's 2-stage procedure, the null hypothesis that the true response rate is 0.30 will be tested against a one-sided alternative. The null hypothesis will be rejected if ~~8~~ 7 or

<table>
<tr><th>Section</th><th>Description of Change</th></tr>
<tr><td></td><td>
more responses are observed at the final dose. This design yields a type I error rate of <s>0.06</s> 0.018 and power of <s>85.1%</s> 96.44% when the true response rate is <s>0.65</s> 0.85.
<table>
<tr><th>Null response rate: P_0</th><th>Alt. response rate: P_1</th><th>Alpha one-tailed</th><th>Power</th><th>n1</th><th>r1</th><th>N</th><th>R</th></tr>
<tr><td>0.30</td><td><s>0.65</s> 0.85</td><td><s>0.06</s> 0.018</td><td><s>85.1%</s> 96.44%</td><td><s>6</s> 5</td><td>2</td><td><s>16</s> 11</td><td><s>7</s> 6</td></tr>
</table>
N is the total number of evaluable subjects at the final dose
n1 is the number of evaluable subjects accrued during Stage 1
r1, if r1 or fewer responses are observed during stage 1, escalate the dose if safety requirements are met
R, if R or fewer responses are observed by the end of Stage 2 in N subjects, then an effective dose is not found

<u>Formerly read:</u>

Study Design: This is a 2-stage adaptive study. For Stage 1, up to 10 subjects will be enrolled for the purpose of 6 evaluable subjects for the 1st stage of the study to determine a safe and efficacious dose. For Stage 2, the target goal is 16 evaluable subjects for the efficacy analysis of the final dose. If the dose remains the same as Stage 1, Stage 2 will target 10 additional evaluable subjects but up to 21 subjects will be recruited if the dose is changed between Stage 1 and 2.

<u>Now reads:</u>

Study Design: This is a 2-stage adaptive study. For Stage 1, up to 10 subjects will be enrolled for the purpose of <s>6</s> 5 evaluable subjects for the 1st stage of the study to determine a safe and efficacious dose. For Stage 2, the target goal is <s>16</s> 11 evaluable subjects for the efficacy analysis of the final dose. If the dose remains the same as Stage 1, Stage 2 will target <s>10</s> 6 additional evaluable subjects but up to 21 subjects will be recruited if the dose is changed between Stage 1 and 2.
</td></tr>
<tr><td>Section 12.1 – Study Monitoring</td><td>
<u>Added:</u>

The personal visits and monitoring are conducted to the extent possible however under certain circumstances such as COVID-19 pandemic, to minimize or eliminate immediate hazards or to protect the life and well-being of research staff and participants, discussions, monitoring and site closures will be performed remotely via technologies including but not limited to phone calls, email, eCRF, eTMF and file sharing on video conferencing for source documents. Sponsor and sites can utilize video and teleconferencing such as ZOOM to remotely discuss, review and approve documents. The PI sign off can be obtained and saved electronically for all eCRFs at the site
</td></tr>
</table>

PROTOCOL CLN-001 Amendment 5

Phase 2, Open-Label, Single-Arm Safety, Tolerability, Anti-Tumor Effects, Systemic Exposure, and Device Technical Effects of PRV111 (Cisplatin Transmucosal System) in Subjects with Oral Squamous Cell Carcinoma

STATISTICAL ANALYSIS PLAN

March 2020

CONFIDENTIAL

Submitted to:
Manijeh Goldberg, PhD, MBA
President and CEO

[redacted]
Medical Monitor

Privo Technologies, Inc.
200 Corporate Place, Suite 6B
Peabody, MA 01960

SIGNATURE PAGE

Reviewed by:

[redacted]

23 March-2020
Date

Sr. Director, Biostatistics
[redacted]

Approved by:

[redacted]

28 March-2020
Date

Medical Monitor

Manijeh

Manijeh Goldberg, PhD, MBA
President and CEO

27 March 2020
Date

LIST OF ABBREVIATIONS AND DEFINITIONS OF TERMS

Abbreviation	Definition
AE(s)	Adverse Event(s)/Adverse Experience(s)
ANCOVA	Analysis of Covariance
BUN	Blood urea nitrogen
CBC	Complete Blood Count
CFB(s)	Change(s) from Baseline
CFR	Code of Federal Regulations
CLP	Cisplatin-Loaded chitosan Particles
CR	Complete response
CRF	Case Report Form
CTCAE	Common Terminology Criteria for Adverse Events
DLT	Dose-limiting toxicity
ECG	Electrocardiogram
ECOG PS	Eastern Collaborative Oncology Group Performance Status
FDA	Food and Drug Administration
GCP	Good Clinical Practice
IB	Investigator’s Brochure
ICF	Informed Consent Form
ICH	International Council on Harmonisation
IND	Investigational New Drug
IRB	Institutional Review Board
IV	Intravenous
LFT	Liver function test
LOCF	Last Observation Carried Forward
NRI	Non-responder Imputation
ORR	Objective Response Rate (Tumor volume change from baseline assessment of CR or PR)
PCFB	Percent change from baseline
PD	Progressive disease
PE	Permeation enhancer
PI	Principal Investigator
PK	Pharmacokinetics
PR	Partial response
PRV111	Cisplatin Transmucosal System
RECIST	Response Evaluation Criteria in Solid Tumors
SAE	Serious Adverse Event
SCC	Squamous cell carcinoma
SD	Stable disease/ Standard deviation for statistical summaries
SOC	Standard of care /<br>MedDRA System Organ Class for AE assessment
TEAE	Treatment Emergent Adverse Events
VAS	Visual analog scale

TABLE OF CONTENTS Page

1 Introduction 6
1.1 Background 6
2 Trial Design 7
2.1 Study Objectives 7
2.2 Study Design 7
2.3 Sample Size 9
2.4 Selection and Withdrawal of Subjects 9
2.4.1 Inclusion Criteria 9
2.4.2 Exclusion Criteria 10
2.5 Criteria for Individual Subject Discontinuation of Protocol Therapy 10
2.6 Criteria for Individual Subject Discontinuation from the Trial 10
2.7 Handling of Subject Withdrawals or Subject Discontinuation of Study Intervention 11
2.8 Replacement of subjects 11
2.9 Treatments Administered 11
a. Description of Test Product 11
b. Dosing and Administration 11
2.10 Dose-limiting Toxicity (Stopping Rules for an Individual Subject) 12
2.11 Schedule of Events 13
2.12 Study Endpoints 16
2.12.1 Primary Endpoints 16
2.12.2 Secondary Endpoints 16
3 Efficacy Assessments 16
3.1 PRIMARY: Tumor Photography and Measurements 17
3.1.1 PRIMARY: Response and Non-Response (Tumor Volume Change from Baseline Criteria) 17
3.1.2 Primary Efficacy Analysis 18
3.2 SECONDARY: Computed Tomography or Magnetic Resonance Imaging of the Head and Neck 19
3.2.1 SECONDARY: Histopathology Response to Treatment 19
3.2.2 SECONDARY: Loco-regional Control 21
3.2.3 SECONDARY: Technical Success 22
3.2.4 Secondary Analysis 23
4 Safety Assessments 23
4.1.1 Physical Examinations Analysis: 24
4.2.1 Oral Cavity Examination Analysis: 26
4.3.1 Neck Lymph Nodes Analysis 26
4.4.1 Laboratory Tests Analysis: 27
4.5.1 ECG Parameters Analysis: 29
4.7 Adverse Events Analysis: 33

4.7.1 Deaths, Other Serious Adverse Events (SAEs), and Other Significant AEs Analysis: 34
4.8 Pharmacokinetics and Biodistribution Assessment 34
4.8.1 Pharmacokinetics Analysis: 34
5 Data Analysis and Statistical Considerations 34
5.1 Statistical Analysis 35
5.2 Treatment Analysis Guidelines 36
5.2.1 Efficacy Analyses 36
5.2.2 Evaluable Population 36
5.2.3 Baseline Values for Tumor Measurement 36
5.2.4 Change from Baseline (CFB) and Percent Change from Baseline (PCFB) 37
5.2.5 Descriptive Summary Statistics 37
5.3 Safety Analyses 37
5.3.1 Safety Population 37
5.3.2 Concomitant Medication and Therapy 37
5.3.3 Medical History, Medication History and Baseline Disease Characteristics 37
5.3.4 Subject Accountability, Demographics, and Baseline Characteristics 38
6 Data Handling 38
6.1 Pooling of Centers 38
6.2 Handling of Missing Data 38
6.3 Drop-outs, Withdrawals, Replacement Policy 38
7 Changes from the Protocol 38
8 Appendices 39
8.1 Appendix 1: Laboratory Values Collected 39
8.2 Appendix 2: ECOG Performance Status (ECOG PS) 51
8.3 Appendix 3: Common Terminology Criteria for Adverse Events (CTCAE) 51

1 Introduction

This Statistical Analysis Plan (SAP) is based on study procedures and analyses described in the protocol (Amendment 5 dated 25 MAR 2020). Table shells and mock listings corresponding to the contents of this document will be included with the final version.

1.1 Background

Squamous cell carcinomas (SCC) of the head and neck include tumors that occur in the oral cavity. Most lip and oral cavity cancers originate in squamous cells, the thin flat cells that line the lips and oral cavity. Risk factors for oral cancer include chronic heavy use of alcohol and tobacco, long periods of exposure to natural or artificial sunlight, and exposure to human papillomavirus. The 5-year survival rate for oral cancer is approximately 60%; diagnosis at an early stage significantly increases the 5-year survival rate. Oral cancer has a high rate of recurrence (18%–76% for subjects who undergo standard treatment). Approximately 1/3 of subjects treated with surgery and adjuvant therapy will experience local or regional recurrence and/or distant metastasis.

The most common chemotherapy drugs for advanced oral cancer includes cisplatin, methotrexate, carboplatin, 5-fluorouracil, paclitaxel, and docetaxel, which when administered alone or in combination have been shown to provide clinical benefit with demonstrated improvement in overall survival.

PRV111 (Cisplatin Transmucosal System) is a thin, 2-layer, self-adhesive transmucosal patch embedded with cisplatin and a non-woven fabric adhesive backing. In addition to the PRV111 drug product, a separately packaged Permeation Enhancer Powder for Reconstitution is used in conjunction with the PRV111 drug product. The reconstituted Permeation Enhancer Solution is applied prior to the administration of PRV111 to improve the absorption of the cisplatin active ingredient.

Privo's PRV111 is intended as a local induction chemotherapy, to improve the local control of the disease prior to surgery by shrinking the tumor size. Local cisplatin delivery may result in larger and more effective doses of cisplatin delivered to the tumor while significantly reducing or eliminating the systemic toxicities and side effects traditionally associated with intravenous (IV) cisplatin chemotherapy. Treatment with cisplatin which is targeted to the tumor site and not administered systemically may reduce the dose-limiting systemic toxicities that are observed with IV cisplatin administration. If proven safe and effective for the treatment of SCC of the oral cavity, PRV111, in association with the standard care, may decrease the risk of local tumor recurrence.

2 Trial Design

2.1 Study Objectives

Primary Objectives

- Determine a safe and efficacious dose of PRV111 (Cisplatin Transmucosal System) (cisplatin drug-loaded patch + permeation enhancer [PE]) under the conditions of the trial.

Secondary Objectives

- Evaluate tumor volume shrinkage
- Assess long-term safety of the treatment
- Evaluate device technical success including the residual cisplatin level in the patch after the application period
- Evaluate systemic, tumor and lymph node (if available) platinum levels following administration

2.2 Study Design

This is a two-stage adaptive design. The First stage with 6 patients focuses on determining a safe dose, while stage 2 expands the study at the previously determined dose. See the Protocol section on synopsis.

Methodology: This is a Phase 1/2, open-label, prospective, single-arm study of the safety and efficacy dose-finding, systemic exposure, and device technical success of PRV111 (Cisplatin Transmucosal System) (cisplatin drug-loaded patch+PE) in subjects with oral squamous cell carcinoma (SCC) amenable to surgery.

Study Design: This is a 2-stage adaptive study. For Stage 1, up to 10 subjects will be enrolled for the purpose of 5 evaluable subjects for the 1st stage of the study to determine a safe and efficacious dose. For Stage 2, the target goal is up to 11 evaluable subjects for the efficacy analysis of the final dose. If the dose remains the same as Stage 1, Stage 2 will target 6 additional evaluable subjects but up to 21 subjects will be recruited if the dose is changed between Stage 1 and 2.

For the purpose of dose selection, tolerable dose is defined as a dose where fewer than 33% of subjects being evaluated within the safety population present with dose-limiting toxicities (DLTs). Effective dose is determined based on the evaluable population as described in the Statistical Methods section.

The 2 stages of the study are described below:

Stage 1: Up to 10 subjects will be enrolled for the purpose of 5 evaluable subjects and undergo 3 treatment applications at each of the 4 planned treatment visits. Each treatment visit will include 1 application of the PE. Starting approximately 5 minutes after application

of the PE, 1 or 2 patches (depending on tumor size) will be applied. A total of 3 treatment applications of patches will occur. These successive applications will span approximately 35 minutes. The 3 applications provide a total of 6 mg of cisplatin per visit (12 mg if 2 patches are applied at the same time). The 4 treatment visits will be separated by 2 to 4 days (at least 48 hours).

The following decisions will be made based on safety and efficacy of the dose:

Dosing Rules for Stage 1:

1) If fewer than 2 subjects experience DLTs, then the following scheme will be considered for stage 2 dosing:
   a. If fewer than or equal to 2 of the evaluable subjects have a response (Defined in the efficacy table) the dose will be escalated from 3 to 5 treatment applications per treatment visit for Stage 2.
   b. If 3 or more evaluable subjects have a response (CR or PR), the dose will remain the same for Stage 2 (3 applications per treatment visit).
2) If 2 or more subjects experience DLTs at any time during Stage 1, the following scheme will be considered for dose de-escalation in Stage 2:
   a. If there are 6 evaluable subjects in Stage 1:
      - If out of 6 evaluable subjects, 2 or more subjects have a response (CR or PR), the dose will be de-escalated to 2 applications per treatment visit.
      - If out of 6 evaluable subjects, fewer than 2 have a response (CR or PR), the dose will be considered intolerable and the study will be stopped.
   b. If Stage 1 dosing was stopped prior to having 6 evaluable subjects, the dose will be de-escalated from 3 applications to 2 applications per treatment visit.

Stage 2: In case of dose escalation or de-escalation, Stage 2 will be conducted in 2 parts: Stage 2a and Stage 2b.

Subjects will receive 2, 3, or 5 treatment applications at each of the 4 planned treatment visits, depending on the results of Stage 1. Up to 21 additional subjects will be enrolled in Stage 2 if necessary, for the purpose of 16 total evaluable subjects at the final dose for the efficacy analysis.

Stage 2a: This stage will include 6 subjects (Stage 2a flowcharts). In this stage, only safety outcomes will be considered for determining the dosing regimen for the set of subjects to be enrolled in Stage 2b. All subjects enrolled in this stage will be monitored for possible DLTs.

1) If fewer than 2 subjects with DLTs in Stage 2a, enrollment will continue for the remaining number of subjects required in Stage 2b at the same dose.
2) If 2 subjects experience DLTs in Stage 2a, dosing will be re-considered. It may be stopped or, when possible, decreased for the remaining subjects enrolled in Stage 2b:
   a. If "Stage 2a - Escalated Dose", the original dose will be used for up to 10 additional evaluable subjects enrolled for Stage 2b.
   b. If "Stage 2a - Same Dose", dosing will be stopped. Additional subjects, for up to 10 evaluable subjects, will be enrolled for Stage 2b at a lower dose, consisting of 2 applications per treatment visit.

c. If "Stage 2a – De-escalated Dose", the study will stop, as no safe dose could be determined.

2.3 Sample Size

Number of Subjects: Up to 31 subjects will be enrolled in the United States for the purpose of 11 total evaluable subjects at the final dose for the efficacy analysis. The design involves 5 evaluable subjects in the 1st stage, and 6 to 11 additional subjects in 2nd stage dependent on any dose changes)

2.4 Selection and Withdrawal of Subjects

2.4.1 Inclusion Criteria

Subjects must meet the following inclusion criteria:

1. Adult subjects, men and women, defined by age ≥18 years at the time of screening.
2. Pathologically proven and clinically confirmed T1 (<2 cm) or T2 (>2 cm but ≤4 cm) SCC of the lip or oral cavity (anterior 2/3 of the tongue, floor of mouth, lower and upper gingiva, salivary gland, hard palate, and buccal mucosa).
3. Tumor must be easily accessible, with no evidence of infection or active bleeding, encroaching major vessels or clinical evidence of neural invasion. Tumors should not have been previously irradiated.
4. Tumor must be amenable to surgical resection no later than 42 days post Visit 1.
5. Clinically and/or radiologically measurable tumor.
6. Eastern Collaborative Oncology Group Performance Status of ≤2.
7. Adequate renal function as demonstrated by renal creatinine clearance ≥50 mL/min (calculated using the Cockcroft-Gault formula).
8. Adequate organ function defined as: ANC ≥1000/µL, platelet ≥100,000/µL; hemoglobin ≥9.0 g/dL; AST and ALT ≤2.5 times the upper limit of normal (ULN), serum bilirubin ≤1.5 times the ULN or direct bilirubin ≤ULN for subjects with total bilirubin >1.5 times the ULN.
9. Candidates for standard of care treatment consisting of surgery.
10. Male and female subjects of childbearing potential must agree to use 2 methods of effective contraception from screening and for at least 30 days after the final dose of investigational product. Appropriate birth control is defined as barrier methods with spermicides, oral or parenteral contraceptives and/or intrauterine devices, or naturally or surgically sterile (with documentation in the subject's medical records). Postmenopausal women are defined as presenting at least 12 months' natural spontaneous amenorrhea, or at least 6 weeks following surgical menopause (bilateral oophorectomy). Females of childbearing potential must be non-lactating and have a negative serum hCG within 14 days of treatment initiation.
11. Absence of any serious underlying medical conditions which could impair the ability of the subject to participate in the study.

12. Have a life expectancy of ≥3 months.
13. Willing and able to provide written informed consent.
14. Able to return to study site for treatment and follow-up visits as defined in the Protocol.

2.4.2 Exclusion Criteria

Subjects meeting any of the following criteria will be excluded from the study:

1. Known distant metastasis of the SCC of the oral cavity
2. Prior radiation and/or systemic chemotherapy for the treatment of SCC of the head and neck less than 2 years prior to screening
3. Concurrent malignancy, with the exception of localized squamous cell carcinomas of the skin
4. Exposure to any investigational agent within 3 months prior to screening
5. Known allergy or hypersensitivity to platinum-containing agents, or known intolerance to a prior platinum-containing agent, or to any of the excipients, which, in the judgement of the physician will preclude re-exposure to platinum-containing agent
6. Active, uncontrolled infection requiring systemic therapy, such as but not limited to HIV, Hepatitis B or C
7. Uncontrolled intercurrent illness that would risk subject safety, interfere with the objectives of the protocol, or limit subject compliance with study requirements, as determined by the Investigator
8. Known or suspected pregnancy, planned pregnancy, or lactation
9. Any medical or psychiatric condition that may compromise the ability to give written informed consent

2.5 Criteria for Individual Subject Discontinuation of Protocol Therapy

Subjects should be discontinued from protocol therapy, while follow-up as per protocol, for the following reasons:

- Completion of the scheduled PRV111 therapy
- Meets 1 of the stopping rules for an individual subject
- Pregnancy
- Noncompliance with trial or follow-up procedures
- Withdrawal at the Investigator's request for any reason that the Investigator believes continuation of study drug therapy would not be in the subject's best interest
- Subjects may be withdrawn at any time for drug intolerability, disease progression, availability of other therapeutic options, or subject judgment

2.6 Criteria for Individual Subject Discontinuation from the Trial

Subjects may withdraw from further participation in the study at any time and for any reason without prejudice. The degree to which a subject withdraws can vary and efforts will be made to collect important safety data if feasible and the subject agrees. A subject will be considered to be withdrawn from the trial for the following reasons:

- Withdrawn consent from all treatment and follow-up

- Death
- Lost to follow-up
- Termination of the trial by the Sponsor

2.7 Handling of Subject Withdrawals or Subject Discontinuation of Study Intervention

If a subject withdraws consent for the treatment, the Investigator will make every effort to complete all final evaluations and procedures required by the Protocol. The subject will be asked to return to the clinic for a follow-up visit to assess overall safety prior to study completion. In the event a subject withdraws for safety reasons (adverse event/serious adverse event [AE/SAE], National Cancer Institute's (NCI) Common Terminology Criteria for Adverse Events [CTCAE] toxicity related events, etc.), every effort will be made to continue to follow-up with the subject until the documented condition resolves to protocol-established grades.
The reasons for discontinuation of dosing must be clearly documented on the case report form (CRF).

2.8 Replacement of subjects

Subjects will not be replaced.

2.9 Treatments Administered

a. Description of Test Product

PRV111 (Cisplatin Transmucosal System) (referred to as PRV111) consists of:

- PRV111 4 cm^2 patch containing 2 mg of cisplatin (0.5 mg/cm^2) backed with a pressure sensitive acrylic adhesive non-woven fabric backing.
- PE Powder for Reconstitution (4 mL borosilicate glass vial containing 50 mg (on anhydrous basis) each of sodium cholate hydrate, sodium taurodeoxycholate hydrate, sodium deoxycholate and sodium glycodeoxycholate) (intended for reconstitution in 1 mL US Pharmacopeia Water for Injection, 50 mg/mL).

b. Dosing and Administration

Each treatment visit will include 1 mL application of the PE. Starting 5 minutes after application of the PE, 1 or 2 patches (depending on tumor size) will be applied. For Stage 1, a total of 3 treatment applications of patches will occur. These successive applications will span approximately 35 minutes. The 3 applications provide a total of 6 mg of cisplatin per visit (12 mg if 2 patches are applied at the same time).

In Stage 2 (Stage 2a and Stage 2b), subjects will receive 2, 3, or 5 treatment applications at each of the 4 planned treatment visits, depending on the results of Stage 1.
Refer to the Pharmacy Manual for detailed instructions on applying the PRV111 patches.
Table 1 presents definition used for dosing and treatment in this study. Treatment is expected to be approximately 35 minutes for each treatment visit.

Table 1. Dosing Terminology

Term	Definition
Treatment visit	Two to 6 days apart, during which PRV111 patches will be administered.
Treatment application	Three patch applications will occur during each treatment visit in Stage 1.<br>Two, 3, or 5 patch applications will occur during each treatment visit in Stage 2.

2.10 Dose-limiting Toxicity (Stopping Rules for an Individual Subject)

Toxicity will be graded and recorded according to the NCI CTCAE version 4.03 (US Department of Health and Human Services, 2010), which are considered at least possibly related to PRV111 Patch. A DLT is defined as a clinically significant Treatment-Emergent AE (TEAE) or laboratory abnormality unrelated to disease progression, concurrent illness, or concomitant therapy and that meets any of the following criteria:

- Grade 3 or 4 non-hematologic toxicity excluding alopecia
- Grade 3 or 4 nausea, vomiting, or diarrhea, lasting >48 hours, despite standard prophylaxis and/or treatment
- Grade 4 neutropenia lasting >5 days.
- Febrile neutropenia, defined as absolute neutrophil count <1000/mm^3 with a single temperature of >38.3°C (101°F) or a sustained temperature of ≥38°C (100.4°F) for more than 1 hour.
- Grade 4 thrombocytopenia or thrombocytopenia with clinically significant bleeding (ie, bleeding that requires blood or platelet transfusion or other medical intervention, or that may cause disability or death, such as cerebral hemorrhage).
- Any grade 3 or 4 toxicity related to the study drug that precludes administration of at least 3 of the 4 planned applications.
- Grade 4 local toxicity- oral mucositis according to World Health Organization scale:
  - Grade 0 - No oral mucositis
  - Grade 1 - Erythema and soreness
  - Grade 2 - Ulcer, able to eat solids
  - Grade 3 - Ulcers, requires liquid diet (due to mucositis)
  - Grade 4 - Ulcers, alimentation not possible (due to mucositis)
- Any treatment related AE leading to surgery delay for more than 96 hours is a DLT

2.11 Schedule of Events

Table 2. Schedule of Events

	**Screening Within 28 Days Prior to Visit 1**	**Days**						**Months Post Surgery**
		**Treatment Visits**				**Post Treatment Pre-Op**	**Surgery**	**Standard of Care (SOC) Follow-up**
		**Visit 1**	**Visit 2**	**Visit 3**	**Visit 4**	**Visit 5**	**Visit 6**	**Visits 7, 8, 9**
	**0**	**1**	**≥48 and ≤ 144 hours, post previous visit**			**≥72 hours post last treatment**	**≤42 days post Visit 1**	**1, 3, & 6 months (all can be +/-3 weeks) SOC visits**
Confirmed SCC	X							
Informed consent	X							
Inclusion/exclusion criteria	X	X[a]						
Schedule study visits	X	X	X	X	X	X		
Demographic, medical/history	X							
Physical examination,[b,s] VS[c]	X	X[a]	X[a]	X[a]	X[a]	X		X
Anti-emetics		X[a]	X[a]	X[a]	X[a]			
CT chest [s]	X							
Oral cavity examination[d]	X	X[a]	X[a]	X[a]	X[a]	X		X
Neck lymph nodes exam	X	X[a]	X[a]	X[a]	X[a]	X		X
ECG [s]	X					X		
CT or MRI head and neck[e, s]	X					X		
Clinical labs[f]	X	X[a]		X[a]		X	X[o]	
HPV [s]	X[g]							
Pregnancy test	X[h]	X[a]			X			
PK sampling		X[i]	X[j]	X[j]	X[i]			
Tumor resection							X[k]	

	Screening Within 28 Days Prior to Visit 1	Days					Surgery	Months Post Surgery
		Treatment Visits				Post Treatment Pre-Op		Standard of Care (SOC) Follow-up
		Visit 1	Visit 2	Visit 3	Visit 4	Visit 5	Visit 6	Visits 7, 8, 9
	0	1	≥48 and ≤ 144 hours, post previous visit			≥72 hours post last treatment	≤42 days post Visit 1	1, 3, & 6 months (all can be +/-3 weeks) SOC visits
PRV111 patch treatment		X[p]	X[p]	X[p]	X[p]			
Tumor measurements[q]		X[a]	X[a]	X[a]	X[a]	X		
Tumor Volume change from baseline (CFB) criteria			X[a]	X[a]	X[a]	X		
Tumor photographs		X[l]	X[l]	X[l]	X[l]	X		
Loco-regional recurrence								X
Histology[s]	X[m]						X[m]	
Technical success[n]		X	X	X	X			
Concomitant therapy	X	X	X	X	X	X		X
AE assessment	X	X	X	X	X	X[r]		

Abbreviations: AE=adverse event; CT=computed tomography; ECG=electrocardiogram; HPV=human papilloma virus; MRI=magnetic resonance imaging; PE=permeation enhancer; PK=pharmacokinetics; SCC=squamous cell carcinoma; VS=vital signs.

a Performed prior to dosing.

b If a physical exam was performed within 14 days prior to the screening visit, it does not need to be repeated at the screening.

c VS: seated blood pressure and pulse will be measured after the subject has been sitting for at least 3 minutes, respiration, temperature, height and weight will be recorded at every visit. VS will be obtained pre-dose at all visits and subject will also be monitored at 30+5 minutes, 60+5 minutes and 120+5 minutes following application of the last patch applied at Visits 1 and 4 and at 30+5 minutes post last patch application at Visits 2 and 3.

d Take photos of the tumor region for further assessment of tumor shrinkage.

e Every attempt should be made to use the same test for each subject for both timepoints.

f CBC and chemistry panel (creatinine to determine the creatinine clearance, BUN, LFT, electrolytes (potassium, magnesium, calcium, and sodium), urinalysis.

g Tissue will be collected at Screening but the HPV status will be ascertained at any time during the study using immunohistochemistry for p16 according to institutional clinical protocols.

h Serum pregnancy test to establish eligibility for female subjects of childbearing potential. A negative urine pregnancy test must be documented within 7 days prior to the first treatment visit, and at Visit 5.

i Visit 1 & 4: PK blood samples for total platinum collected pre- treatment (time -15 to 0 minutes), and 30±5 minutes, 60±5 minutes and 120±5 minutes post removal of last patch.

j Visit 2 & 3: PK blood samples for total platinum collected pre- treatment (time -15 to 0 minutes), and 30±5 minutes, post removal of last patch and PE application.

k Sampling for tumor and lymph nodes (if available) platinum levels.

l Photos need to be taken prior to patch application.
m Confirmation of tumor histology by a clinical pathologist is required prior to enrollment. A final pathology report, issued by a clinical pathologist is required following surgical resection of the tumor specimen.
n Taste of the PE solution and patch, adhesion, peel-off, residual cisplatin level in the patch.
o Performed after surgery if possible
p PE should be applied to the tumor area prior to first patch and allow to stay for 5 minutes. Then applied again to the tumor area for 1 minute. Note that the mouth should be kept open for the saliva not wash off the PE.
q Use “0.05” cm for the “Height” of a flat tumor. If a concave tumor, the “Height” can be shown as a negative number. If a non-measurable dent, use “-0.05” cm.

r Only SAEs within 30 days post visit 5 need to be recorded.
s. May use historical SOC information prior to consent if collected within 28 days prior to first treatment.

2.12 Study Endpoints

2.12.1 Primary Endpoints

The primary endpoints for the study involve determination of a safe and efficacious dose of PRV111 (Cisplatin Transmucosal System) (cisplatin drug-loaded patch + PE) under the conditions of the trial. Therefore, the primary endpoint is based on the incidence of DLTs and tumor volume change from baseline (CFB).

2.12.2 Secondary Endpoints

Based on secondary objectives, the secondary endpoints are:

- Evaluate tumor response
  - CT or MRI of the head and neck based on RECIST 1.1
  - Histology at the time of surgical excision
- Assess short and long-term safety of the treatment
  - Loco-regional control: subjects will be followed-up for disease recurrence and safety following surgery
  - Incidence of recurrence at 6 months
- Total platinum assessment in blood, tumor and lymph nodes (if available)
- Technical Success
  - Successful adhesion of the patch to the target lesion
  - Successful delivery (including release and permeation) of the drug to the target lesion, without getting to surrounding oral surfaces
  - Successful peel of the used patch after 10 minutes without damaging the target lesion underneath
  - Adhesion of the backing to the polymeric matrix embedded CLPs
  - Taste of the PE solution
  - Taste of the PRV111 patch
  - Residual cisplatin level in the patch after the application period

3 Efficacy Assessments

Efficacy outcomes include:

- Response to treatment evaluated by Tumor volume change from baseline (CFB)
- CT Scan/MRI RECIST 1.1
- Incidence of recurrence at 6 months
- Histological effect of PRV111 on the tumor tissue

3.1 PRIMARY: Tumor Photography and Measurements

The primary lesion will be photographed before PRV111 treatment. This will be done to allow for a clinical assessment of response to treatment. For details refer to Study Manual for instructions.
The tumor will be measured in cm to 1 digit to the right of the decimal point using a disposable plastic ruler or caliper. Three dimensions must be recorded (Length x Width x Height).

Note: If tumor or cancerous lesion is flat, a small number (0.05 cm) will be recorded for the Height. If the tumor is a concave, a negative Height is recorded. If the height is an unmeasurable dent, it can be recorded as (-0.05 cm). The volume calculations use the heights absolute value. The following formula will be used to calculate tumor volume (cm^3)

$$L \times W \times H \times \pi/6$$

3.1.1 PRIMARY: Response and Non-Response (Tumor Volume Change from Baseline Criteria)

The terms “response” and “non-response” will be defined according to the tumor volume change from baseline (CFB) criteria:

PCFB = % tumor volume change from the baseline

Response will be defined as CR, SR, or PR. Non-Response will be defined as SD or PD.

Clinical Response to Treatment

Table 2. The Efficacy Criteria

Complete Response (CR)	Disappearance of primary lesion.
Partial Response (PR)	PCFB: ≥30% to 69% decrease in the total tumor volume shrinkage (as determined by clinical measurement, **taking as reference the baseline volume.**
Strong Response (SR)	PCFB: ≥70% to 99% decrease in the total tumor volume shrinkage (as determined by clinical measurement, **taking as reference the baseline volume.**
Progressive Disease (PD)	PCFB: At least a 20% increase tumor volume as determined by clinical measurement **taking as reference the baseline volume** (Note: the appearance of one or more new lesions is also considered progression).
Stable Disease (SD)	Neither sufficient shrinkage to qualify for PR nor sufficient increase to qualify for PD, taking as reference the volume determined by clinical measurement **at baseline**

3.1.2 Primary Efficacy Analysis

The primary efficacy variable is response according to the Tumor Volume criteria per agreement from the FDA. (Section 7.3.1), where a response is CR, SR or PR (ORR; Objective Response Rate) and a non-response is SD or PD (See Efficacy Criteria table). The primary analysis is the analysis using the Efficacy Population and tumor volume change from baseline (CFB). Include 95% CI for CFB tumor size. The study will be deemed a success if at least 7 of the up to 11 evaluable subjects achieve a minimum of a PR.

Change of tumor volume will be summarized for each visit, however the final efficacy determination will be based on CFB from V1 to V5.

A frequency distribution of efficacy based on overall response rate (ORR) at each visit will be provided evaluable population. A one-sided test of response (CR, PR, SR) rate less than 30% will be calculated at each visit.

Photographs from V1 vs V5 will be presented

Size of Tumor 1

Length(cm)	1.9000
Width(cm)	1.3000
Height/Depth(cm)	0.0500
Tumor Volume (cm^3)	0.0646
Baseline Volume (cm^3) (From Visit 1)	0.6908

003-002	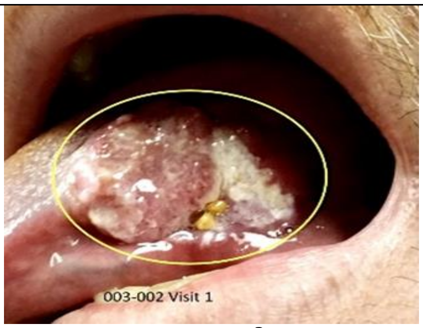 Volume: 1232 mm^3	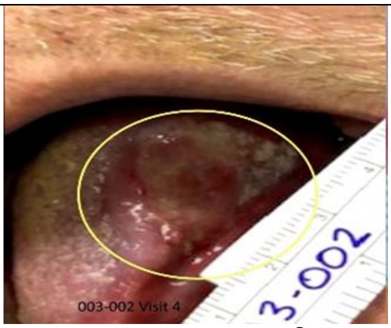 Volume: 14 mm^3

3.2 SECONDARY: Computed Tomography or Magnetic Resonance Imaging of the Head and Neck

A computed tomography scan (CT) or magnetic resonance imaging (MRI) of the head and neck will be performed. For each subject, the same imaging modality should be used at Screening and Visit 5 for consistency.

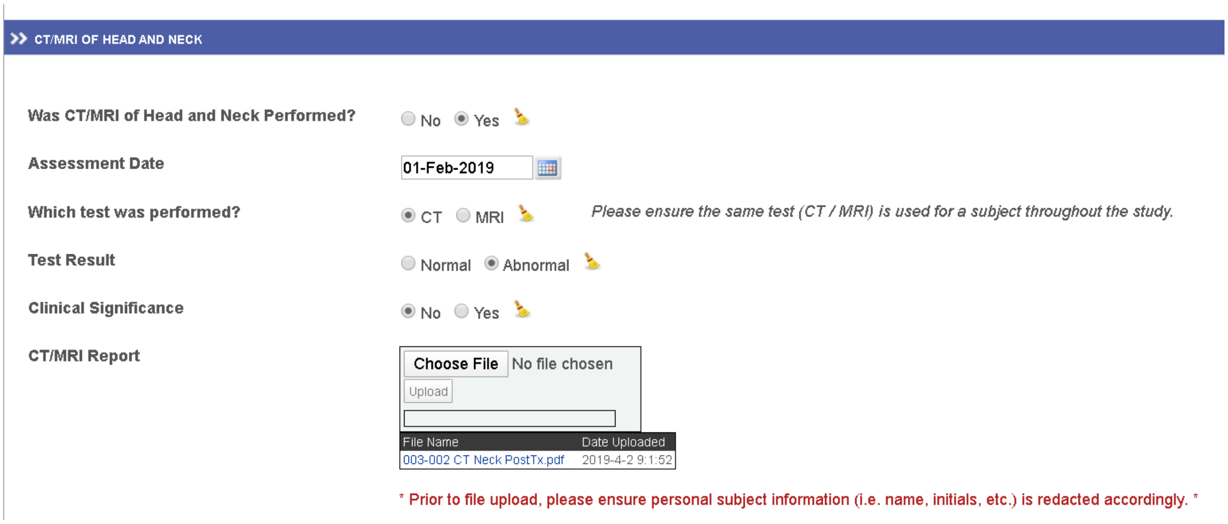

CT/MRI OF HEAD AND NECK

Was CT/MRI of Head and Neck Performed? ○ No ◉ Yes

Assessment Date 01-Feb-2019

Which test was performed? ◉ CT ○ MRI *Please ensure the same test (CT / MRI) is used for a subject throughout the study.*

Test Result ○ Normal ◉ Abnormal

Clinical Significance ◉ No ○ Yes

CT/MRI Report Choose File No file chosen

Upload

File Name	Date Uploaded
003-002 CT Neck PostTx.pdf	2019-4-2 9:1:52

* Prior to file upload, please ensure personal subject information (i.e. name, initials, etc.) is redacted accordingly. *

3.2.1 SECONDARY: Histopathology Response to Treatment

Histological samples obtained at Baseline and from the surgical tumor excision will be compared. A pathologist will assess tumor margins and regression defined as: reduction of

tumor density, necrosis, fibrosis, inflammatory infiltrates, histocyte accumulation, and giant cell reactions.

Grade	Pathological Response to Treatment
1	Extensive residual cancer with no evident tumor regression (poor or no response, more than 90% of viable tumor).
2	Residual cancer with evident tumor regression, but still more than half of tumor left (partial response, approximately 51% - 89% of viable tumor).
3	Great response, with less than half tumor left, but more than single cells or rare small groups of cancer cells (partial response, approximately 11%-50% of viable tumor).
4	Single cells or rare small groups of cancer cells (near complete response, less than or equal to 10% of viable tumor).
5	No viable cancer cells (complete response).

* Grading

Missing Data:
If Baseline data is missing: Only the surgical resection grading will be reported
If clear grading is missing: the secondary objective is not evaluable.

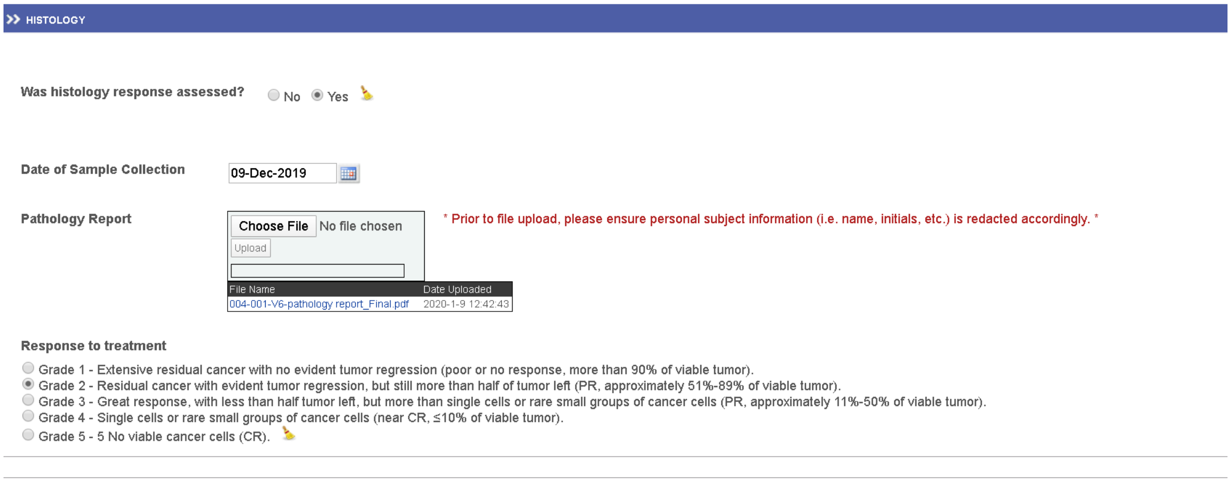
HISTOLOGY

Was histology response assessed? No / Yes (selected)

Date of Sample Collection: 09-Dec-2019

Pathology Report: Choose File — No file chosen — Upload

* Prior to file upload, please ensure personal subject information (i.e. name, initials, etc.) is redacted accordingly. *

File Name	Date Uploaded
004-001-V6-pathology report_Final.pdf	2020-1-9 12:42:43

Response to treatment
- Grade 1 - Extensive residual cancer with no evident tumor regression (poor or no response, more than 90% of viable tumor).
- Grade 2 - Residual cancer with evident tumor regression, but still more than half of tumor left (PR, approximately 51%-89% of viable tumor). (selected)
- Grade 3 - Great response, with less than half tumor left, but more than single cells or rare small groups of cancer cells (PR, approximately 11%-50% of viable tumor).
- Grade 4 - Single cells or rare small groups of cancer cells (near CR, ≤10% of viable tumor).
- Grade 5 - 5 No viable cancer cells (CR).

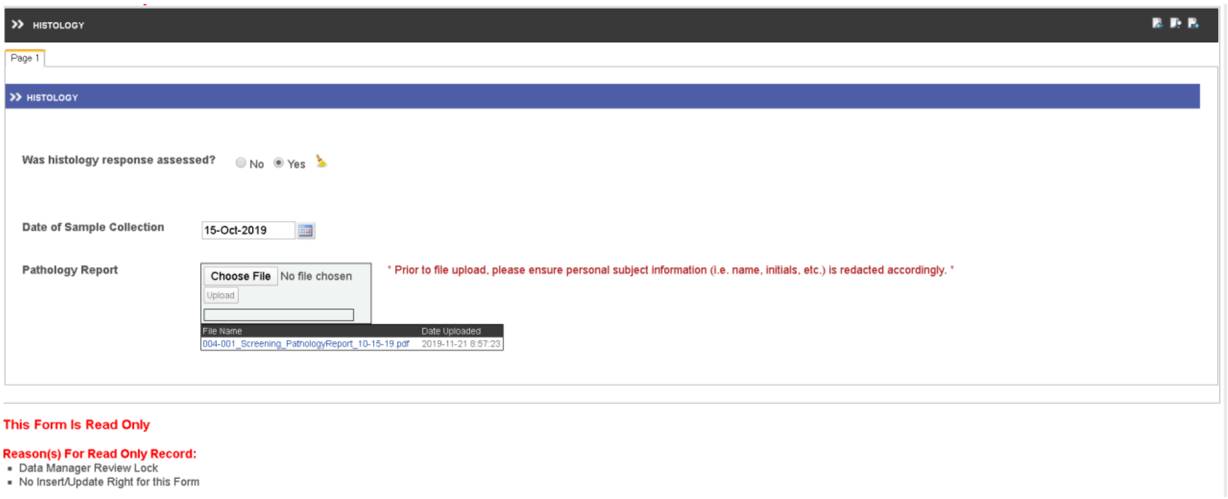

HISTOLOGY

Page 1

HISTOLOGY

Was histology response assessed? No Yes

Date of Sample Collection 15-Oct-2019

Pathology Report Choose File No file chosen Upload

* Prior to file upload, please ensure personal subject information (i.e. name, initials, etc.) is redacted accordingly. *

File Name	Date Uploaded
004-001_Screening_PathologyReport_10-15-19.pdf	2019-11-21 8:57:23

This Form Is Read Only

Reason(s) For Read Only Record:

- Data Manager Review Lock
- No Insert/Update Right for this Form

3.2.2 SECONDARY: Loco-regional Control

Subjects will be followed-up for 6 months, and the incidence of local and regional (nodes) tumor recurrence will be recorded.

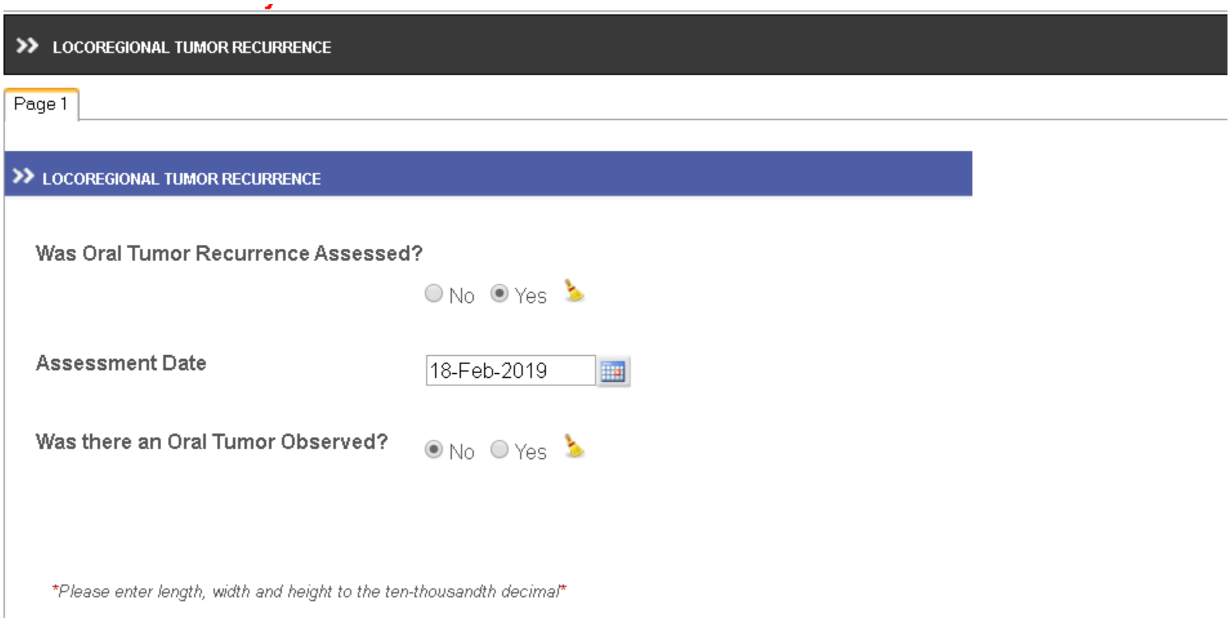

LOCOREGIONAL TUMOR RECURRENCE

Page 1

LOCOREGIONAL TUMOR RECURRENCE

Was Oral Tumor Recurrence Assessed? No Yes

Assessment Date 18-Feb-2019

Was there an Oral Tumor Observed? No Yes

Please enter length, width and height to the ten-thousandth decimal

The tables for this displaying this seconday should be per subject and the result of each followup visit should be displayed.

A summary table will show if any tumor recurred in the evaluable patients at 1,3, and 6 month follow up visits. The final analysis will include the summary of the incidence of tumor recurrence and survival status for the Efficacy of Evaluable Population.

Time to recurrence and time to death will be summarized, with Kaplan-Meier curves will be described in the CSR and corresponding summary statistics at months 1, 3 and 6.

3.2.3 SECONDARY: Technical Success

Technical assessment data will include:

- Taste of the PE solution
- Taste of the PRV111 patch
- Adhesion, including the time of adhesion of PRV111 patch
- PRV111 patch peel-off
- Residual cisplatin level in the patch after the application period. All PRV111 patches will be assessed for the amount of residual cisplatin in each patch after each application period. If the product detaches prematurely, the residual cisplatin will be assessed and the time of detachment will be noted.

Results will be summarized using descriptive statistics.

Were all patches easily removed from the application site?	Specify Reason if patch was not easily removed	Did any patch separate from the fabric backing?
No Yes	Excessive adherence to the application site<br>Abrasion of application site	No Yes
No Yes	Excessive adherence to the application site<br>Abrasion of application site	No Yes
No Yes	Excessive adherence to the application site<br>Abrasion of application site	No Yes

3.2.4 Secondary Analysis

This secondary CT/MRI analysis will be described in the CSR, since data can't be extracted from the unstandardized CT or pathology reports.

4 Safety Assessments

Safety observations and measurements including study drug exposure, AEs, laboratory data, physical examination findings, VS, weight, and concomitant therapy will be summarized and presented in tables and listings SAEs will be collected within 30 days post-surgery. Certain key parameters such as "Expected" and "Related to treatment" will be recorded. AEs that occur from visit 1 to visit 5 are documented. AEs (not SAEs) during or after surgery are not documented since the oral tumor and reconstructive surgeries can result in many AEs. The list of expected SAEs due to oral cancer surgery and reconstructive surgery is included in the Investigator Brochure. Laboratory parameters will be summarized using descriptive statistics and data listings. The incidence of clinically significant abnormalities will be summarized. Physical examinations, VS, and weight will be summarized by changes from baseline values using descriptive statistics.

AEs occurring after the start of treatment are TEAEs. The severity of the TEAEs will be graded according to the CTCAE criteria, version 4.0. The severity of oral mucositis will be graded according to the WHO grade. The number and percentages of subjects experiencing TEAEs will be tabulated by system organ class and preferred term. Assessments of TEAEs will include characterization of the type, incidence, severity, seriousness, and relationship to treatment. Summary subject listings will be provided for subjects with dose modification, SAEs, TEAEs resulting in study discontinuation, and deaths. The rates of local tolerability and oral mucositis will be reported.

Safety assessments include:

- Physical Examination, Vital Signs, Weight
- Oral Cavity Examination
- Neck Lymph Nodes Examination
- Safety Laboratory Assessments
- Electrocardiograms
- Adverse events

4.1 *Physical Examination, Vital Signs, Weight*

A complete physical examination will be performed by the Investigator or Sub-investigator.

The physical examination will include a review of all body systems (head, eyes, nose and throat, cardiovascular, respiratory, gastrointestinal, musculoskeletal, neurological, lymph nodes, dermatological, extremities/joints), and weight.

Seated blood pressure (mmHg), pulse (beats per minute) and temperature (degrees Celsius) will be measured after the subject has been sitting for at least 3 minutes.

Neuropathy and ototoxicity will be assessed via clinical examination since cisplatin can potentially cause neurotoxicity and ototoxicity. Questions will be asked to document symptoms such as tinnitus, pain in the extremities, or loss of sensation in the eCRFs.

4.1.1 Physical Examinations Analysis:

A complete general physical examination, including height and weight and vital signs (VS), will be assessed and recorded. Seated blood pressure (mmHg), pulse (beats per minute) and temperature (degrees Celsius) will be measured after the subject has been sitting for at least 3 minutes. Measured values of all vital signs and the corresponding CFBs (Change from baseline) will be summarized with descriptive statistics by visit. All observed vital signs and corresponding CFB will be presented in a listing. All abnormal physical examination findings will be included in the adverse events listings and summary tables.

Height is measured only at the screening visit and will not be included in the vital signs summary table but will be included in the listing.

Body System	Status	Clinical Significance	If Abnormal, specify (and record on Adverse Event eCRF if Clinically significant)
HEENT ▼	○ Normal ◉ Abnormal ○ Not Done	○ No ◉ Yes	Positive for mouth sores.
Cardiovascular ▼	○ Normal ◉ Abnormal ○ Not Done	◉ No ○ Yes	Positive for chest pain.
Respiratory ▼	◉ Normal ○ Abnormal ○ Not Done		
Gastrointestinal ▼	○ Normal ◉ Abnormal ○ Not Done	◉ No ○ Yes	Positive for abdominal paid, constipation, nausea and rectal pain.
Musculoskeletal ▼	○ Normal ◉ Abnormal ○ Not Done	◉ No ○ Yes	Positive for joint swelling; bilateral swelling in knees. Swan neck deformities r
Neurological ▼	○ Normal ◉ Abnormal ○ Not Done	◉ No ○ Yes	Positive for weakness; generalized
Dermatological ▼	◉ Normal ○ Abnormal ○ Not Done		
Extremities/Joints ▼	○ Normal ○ Abnormal ◉ Not Done		
Other ▼	○ Normal ○ Abnormal ◉ Not Done		

*If **Other**, please specify the body system along with the abnormality in "**If Abnormal, specify**" column.*

4.2 *Oral Cavity Examination*

A systematic and thorough visual and manual oral examination will be performed. This will include a visual examination of all the soft tissues of the mouth, including manual extension of

the tongue to examine its base, a bi-manual palpation of the floor of the mouth, and a digital examination of the borders of the tongue.

Special attention must be given to the application site and the tumor, with documentation of any local adverse reactions, including documentation with photographs if warranted.

4.2.1 Oral Cavity Examination Analysis:

A systematic and thorough visual and manual oral examination will be performed. This will include a visual examination of all the soft tissues of the mouth, including manual extension of the tongue to examine its base, a bi-manual palpation of the floor of the mouth, and a digital examination of the borders of the tongue. Results will be summarized using descriptive statistics

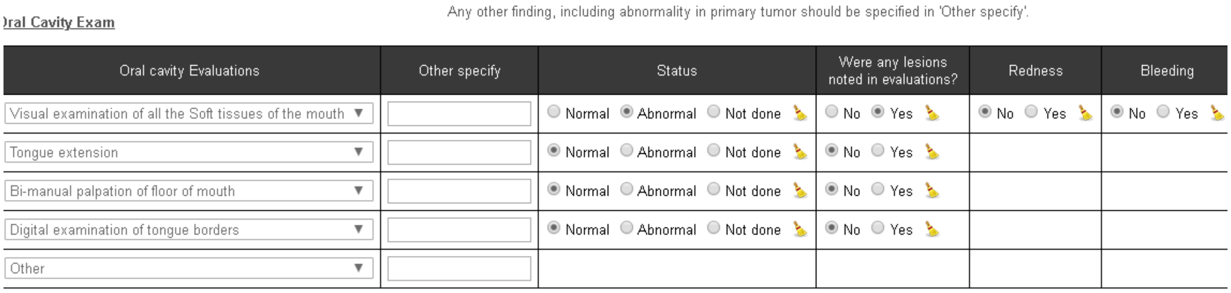

)ral Cavity Exam

Any other finding, including abnormality in primary tumor should be specified in 'Other specify'.

Oral cavity Evaluations	Other specify	Status	Were any lesions noted in evaluations?	Redness	Bleeding
Visual examination of all the Soft tissues of the mouth		Normal ◉ Abnormal Not done	No ◉ Yes	◉ No Yes	◉ No Yes
Tongue extension		◉ Normal Abnormal Not done	◉ No Yes		
Bi-manual palpation of floor of mouth		◉ Normal Abnormal Not done	◉ No Yes		
Digital examination of tongue borders		◉ Normal Abnormal Not done	◉ No Yes		
Other					

4.3 *Neck Lymph Nodes Examination*

A cervical and local lymph nodes examination will be performed.

4.3.1 Neck Lymph Nodes Analysis

A cervical and local lymph nodes' examination will be performed. Results will be summarized using descriptive statistics.

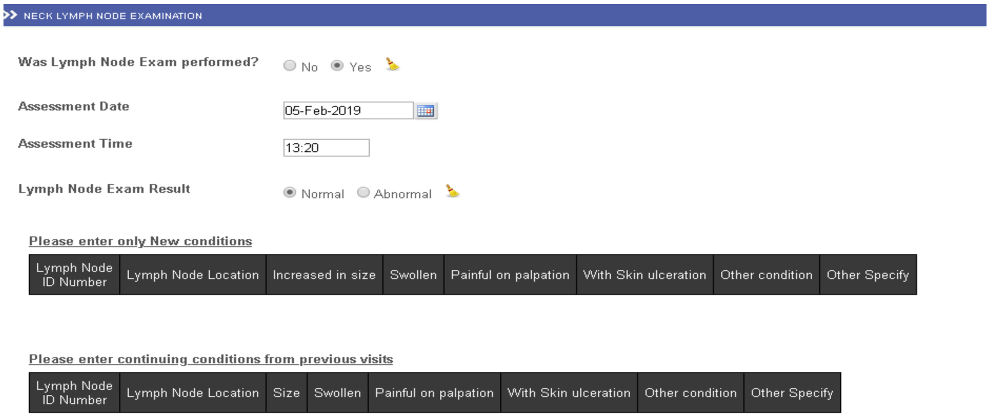

NECK LYMPH NODE EXAMINATION

Was Lymph Node Exam performed? No ◉ Yes

Assessment Date 05-Feb-2019

Assessment Time 13:20

Lymph Node Exam Result ◉ Normal Abnormal

Please enter only New conditions

Lymph Node ID Number	Lymph Node Location	Increased in size	Swollen	Painful on palpation	With Skin ulceration	Other condition	Other Specify

Please enter continuing conditions from previous visits

Lymph Node ID Number	Lymph Node Location	Size	Swollen	Painful on palpation	With Skin ulceration	Other condition	Other Specify

4.4 Safety Laboratory Assessments

Clinical laboratories to be assessed will be as in Table 3.

Table 3. Clinical Laboratories

COMPLETE BLOOD COUNT	CHEMISTRY PANEL	
• hematocrit<br>• hemoglobin<br>• platelet count<br>• red blood cell count<br>• white blood cell count and differential (absolute and percentage); neutrophils, lymphocytes, monocytes, eosinophils, basophils, and reticulocyte count	• alanine aminotransferase<br>• albumin<br>• alkaline phosphatase<br>• aspartate aminotransferase<br>• bilirubin (direct and total)<br>• blood urea nitrogen<br>• calcium<br>• CO2	• chloride<br>• creatinine[a]<br>• gamma glutamyl transferase<br>• glucose<br>• magnesium<br>• potassium<br>• sodium<br>• total protein
URINALYSIS		
• urinalysis dipstick with reflex microscopic examination for abnormalities		

[a] Creatinine Clearance will be used to assess renal function using Cockcroft-Gault Equation

4.4.1 Laboratory Tests Analysis:

Laboratory values such as routine blood chemistry, hematology, and urinalysis will be measured for each subject. Summary statistics will be provided by visit for blood chemistry and hematology and the corresponding CFBs. The calculation for CFB is described in Section9.5. If the value at Baseline is missing, then CFB will be defined as the change from the value recorded at Screening. Urinalysis results and serum and urine pregnancy test results will be included in the listings of laboratory parameters but not in the summary tables. Laboratory ranges displayed and used for normal values will be per site. If a laboratory is missing ranges, the next most-similar lab will be used. For example, if Site 001 is missing a normal range, another lab from the same site will be used as reference (i.e. Baylor HHS is missing red blood cells, the value from Baylor Austin will be used). Data will be displayed per lab and per patient.

Lab Test	Result	Unit	Is result out of normal range?	If out of range, is it clinically significant?	Not Done
White Blood Cell Count	7.3	10*3/µL	● No ○ Yes	○ No ○ Yes	☐
Red Blood Cell Count	3.81	10*6/µL	● No ○ Yes	○ No ○ Yes	☐
Hemoglobin	11.9	g/dL	● No ○ Yes	○ No ○ Yes	☐
Hematocrit	36.4	%	● No ○ Yes	○ No ○ Yes	☐
Platelet Count	315	Thous/µL	● No ○ Yes	○ No ○ Yes	☐
Neutrophils	62.3	%	● No ○ Yes	○ No ○ Yes	☐
Lymphocytes	28.0	%	● No ○ Yes	○ No ○ Yes	☐
Monocytes	8.0	%	● No ○ Yes	○ No ○ Yes	☐
Eosinophils	1.3	%	● No ○ Yes	○ No ○ Yes	☐
Basophils	0.4	%	● No ○ Yes	○ No ○ Yes	☐
Reticulocyte	1.56	%	● No ○ Yes	○ No ○ Yes	☐
Neutrophil Absolute Count	4548	uL	● No ○ Yes	○ No ○ Yes	☐
Lymphocyte Absolute Count	2044	uL	● No ○ Yes	○ No ○ Yes	☐
Monocytes Absolute Count	584	uL	● No ○ Yes	○ No ○ Yes	☐
Eosinophils Absolute Count	95	uL	● No ○ Yes	○ No ○ Yes	☐
Basophils Absolute Count	29	uL	● No ○ Yes	○ No ○ Yes	☐
Reticulocyte Absolute Count	59436	/uL	● No ○ Yes	○ No ○ Yes	☐
Albumin Level	4.0	g/dL	● No ○ Yes	○ No ○ Yes	☐
Alkaline Phosphatase	56	U/L	● No ○ Yes	○ No ○ Yes	☐
BUN	13	mg/dL	● No ○ Yes	○ No ○ Yes	☐
Calcium	9.9	mg/dL	● No ○ Yes	○ No ○ Yes	☐
Creatinine	0.91	mg/dL	● No ○ Yes	○ No ○ Yes	☐
Creatinine clearance	53	mL/min	○ No ○ Yes	○ No ○ Yes	☐
Glucose	112	mg/dL	○ No ● Yes	● No ○ Yes	☐
Protein Total	6.7	g/dL	● No ○ Yes	○ No ○ Yes	☐
AST(SGOT)	16	U/L	● No ○ Yes	○ No ○ Yes	☐
ALT(SGPT)	9	U/L	● No ○ Yes	○ No ○ Yes	☐
Bilirubin Total	0.6	mg/dL	● No ○ Yes	○ No ○ Yes	☐
Bilirubin Direct	0.1	mg/dL	● No ○ Yes	○ No ○ Yes	☐
Sodium	140	mmol/L	● No ○ Yes	○ No ○ Yes	☐
Potassium	3.7	mmol/L	● No ○ Yes	○ No ○ Yes	☐
Chloride	102	mmol/L	● No ○ Yes	○ No ○ Yes	☐
Magnesium	1.9	mg/dL	● No ○ Yes	○ No ○ Yes	☐
CO2	28	mmol/L	● No ○ Yes	○ No ○ Yes	☐
GGT	17	U/L	● No ○ Yes	○ No ○ Yes	☐

4.5 Electrocardiograms

Electrocardiograms (ECGs) will be evaluated for clinically relevant changes from Baseline.

4.5.1 ECG Parameters Analysis:

ECG parameters will be reported as normal or abnormal per subject and per visit.

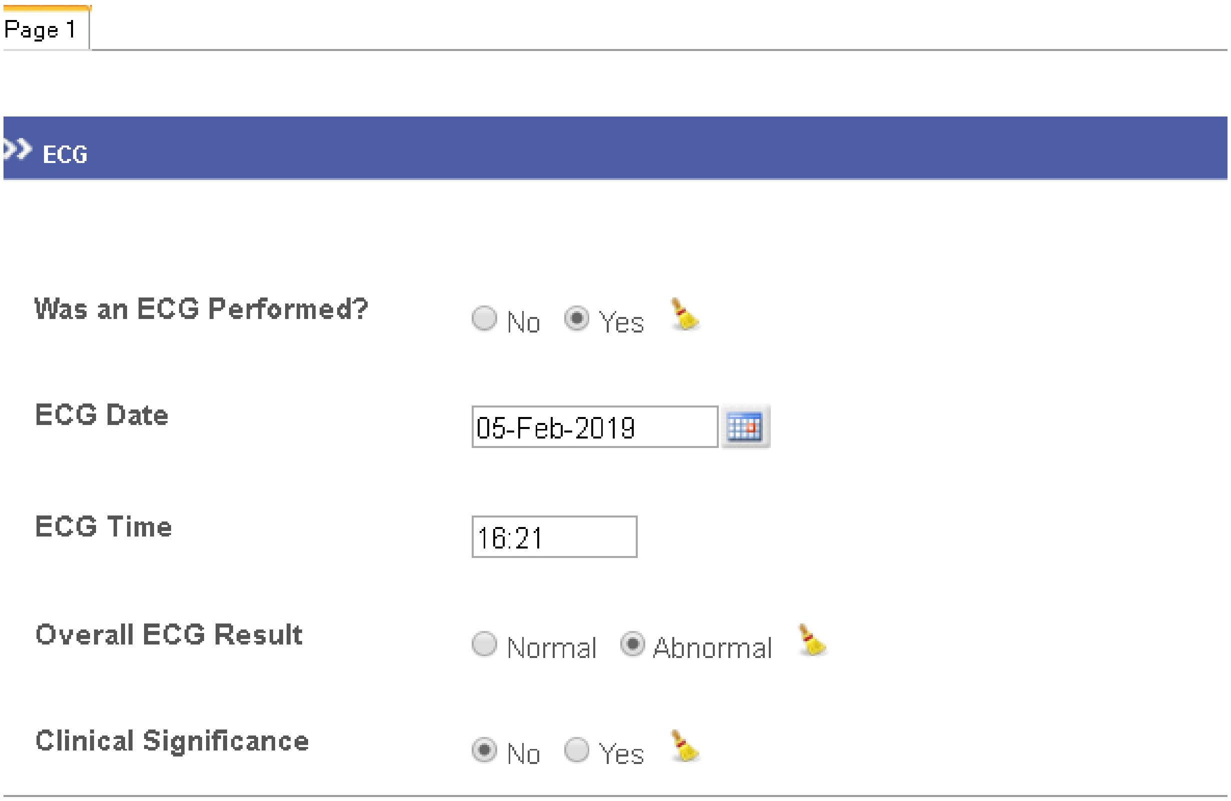

Page 1

ECG

Was an ECG Performed?	○ No ◉ Yes
ECG Date	05-Feb-2019
ECG Time	16:21
Overall ECG Result	○ Normal ◉ Abnormal
Clinical Significance	◉ No ○ Yes

4.6 Adverse Event Definitions/Reporting Requirements

An AE (also known as an "adverse experience") is defined as any untoward medical occurrence associated with the use of a drug in humans, whether or not considered drug related (US Code of Federal Regulations [CFR] 312.32).

An AE can therefore be any unfavorable and unintended sign (including an abnormal laboratory finding), symptom, or disease temporally associated with the use of the drug, without any judgment about causality. A TEAE, treatment emergent adverse event is an AE that occurred on or after the start of treatment.

All clinical AEs noted during the study will be reported on the appropriate AE page of the CRF.

AEs that are serious, unexpected, and at least possibly related to study participation will be reported to the IRB and to the US FDA within the timelines described below. All other AEs, such as those that are expected, or definitely not related to the study participation, are to be reported per the IRB's policy and to FDA as part of regular data submission at the time of annual Investigational New Drug report.

4.6.1 Expected Events

Expected events are those that have been previously identified as resulting from administration of the study drug. An AE is considered unexpected, for expedited reporting purposes only, when either the type of event or the severity of the event is not listed in the current Investigator's Brochure (IB).

4.6.2 Suspected Adverse Reaction

A suspected adverse reaction is a subset of all AEs for which there is a reasonable possibility (ie, evidence to suggest a causal relationship between the drug and the AE) that the drug caused the event. Suspected adverse reaction implies a lesser degree of certainty about causality than adverse reaction.

4.6.3 Adverse Reaction

An adverse reaction is a subset of all suspected adverse reactions and is defined as any AE caused by a drug.

4.6.4 Adverse Device Effects

Adverse device effects are those AEs thought to be related to the use of an investigational medical device. This definition includes AEs resulting from insufficient or inadequate instructions for use, deployment, implantation, installation, or operation, or any malfunction of the investigational medical device. This definition also includes any event resulting from use error or from intentional misuse of the investigational medical device.

An Unanticipated Adverse Device Effect is an adverse device effect, which by its nature, incidence, severity or outcome has not been identified in available information on the device.

Additional device issues, malfunctions or misuse will be documented in the electronic CRF.

4.6.5 Serious Adverse Event Definitions / Reporting Requirements

4.6.6 Serious Adverse Event or Serious Suspected Adverse Reaction

An AE or suspected adverse reaction is considered serious if, in the view of either the Investigator or Sponsor, it results in any of the following outcomes:

- Death. This includes any death that occurs while the subject is on study and/or within 30 days after the last dose of study drug. An autopsy will be requested, and if performed, results will be submitted to the Sponsor.
- A life-threatening AE. An AE or suspected adverse reaction is considered life-threatening if, in the view of either the Investigator or Sponsor, its occurrence places the subject at immediate risk of death. It does not include a reaction that had it occurred in a more severe form, might have caused death.
- Inpatient hospitalization or prolongation of existing hospitalization. In the absence of an AE, hospitalization or prolongation of hospitalization should not be reported as an serious AE (SAE) in the following situations:
  - Hospitalization or prolongation of hospitalization is needed for a procedure required by the Protocol.
  - Hospitalization or prolongation of hospitalization is part of routine procedure followed by study center.
  - Hospitalization for survey visits or annual physicals.
  - For a hospitalization planned (and documented) before the start of the study for a pre-existing condition which has not worsened.
- A persistent or significant incapacity or substantial disruption of the ability to conduct normal life functions.
- A congenital anomaly/birth defect.

Important medical events that may not result in death, be life-threatening, or require hospitalization may be considered serious when, based upon appropriate medical judgment, they may jeopardize the subject and may require medical or surgical intervention to prevent one of the outcomes listed in this definition. Examples include allergic bronchospasm requiring intensive treatment in an emergency room or at home, blood dyscrasias or convulsions that do not result in inpatient hospitalization, or the development of drug dependency or drug abuse.

4.6.7 Pregnancy

For safety reporting purposes, any pregnancy occurring during the study will be considered an “important medical event”. If a study subject becomes pregnant while participating in the study, the study drug will be discontinued immediately and the pregnancy will be reported to the Sponsor. The Investigator will then report follow-up information to the Sponsor regarding the course of the pregnancy, including perinatal and neonatal outcome, regardless of the fact that the subject has discontinued the study. Once the newborn is determined to be healthy, as defined by and agreed upon by the Sponsor and Investigator, additional follow-up will no longer be required.

4.6.8 Serious Unexpected and Suspected Adverse Reaction

An unexpected AE (or serious and unexpected suspected adverse reaction [SUSAR]) is any AE or suspected adverse reaction that is not listed in the IB or is not listed at the specificity or severity that has been observed, if an IB is not required/available, is not consistent with the risk information described in the general investigational plan. This also refers to AEs or suspected

adverse reactions mentioned in the IB as occurring with a class of drugs or as anticipated from the pharmacological properties of the drug but are not specifically mentioned as occurring with the study drug. A SUSAR requires mandatory expedited reporting to the applicable regulatory authority(ies). The Sponsor will forward the formal notification describing the SUSAR to Investigators. Each Investigator must then notify his/her IRB of the SUSAR, in accordance with the IRB's policy.

The Sponsor should be notified within 24 hours of the Investigator becoming aware of a SUSAR and the event documented on the CRF.

4.6.9 Recording Adverse Events

All AEs (including SAEs) within the 5 pre-surgery visits are to be accurately recorded on the AE/SAE page(s) of the CRF. In addition, any SAEs within 30 days of visit 5 will need to be recorded. The Investigator will carefully evaluate each AE to determine:

- Duration (start and end dates)
- Severity (grade)
- Seriousness
- Relationship to study drug (Not Related, Possible, Definite)
- Action taken (none, study drug modification, medical intervention)
- Outcome (resolved without sequelae, resolved with sequelae, ongoing)

The Investigator will evaluate all AEs and SAEs with regard to maximum intensity and relationship to study drug. Maximum intensity should be assigned using one of the severity grades as outlined in the NCI CTCAE v4.0. If the AE is not specifically listed in CTCAE v4.0, the following grades will be used:

- Grade 1: mild
- Grade 2: moderate
- Grade 3: severe
- Grade 4: life-threatening or disabling
- Grade 5: death

4.6.10 Determining Relationship of Adverse Events to Study Drug

The Investigator must attempt to determine if an AE or SAE is related to the use of the study drug. This relationship should be described as follows:

Possible The event follows a reasonable temporal sequence from administration of the study drug and the event follows a known response pattern to the study drug BUT the event could have been produced by an intercurrent medical condition which, based on the pathophysiology of the condition and the pharmacology of the study drug, would be unlikely to be related to the use of the study drug OR the event could be the effect of a concomitant therapy.

Definite The event follows a reasonable temporal sequence from administration of the study drug, the event follows a known response pattern to the study drug and

based on the known pharmacology of the study drug, the event is clearly related to the effect of the study drug.

Not Related Based on the evidence available, causality is not possible.

4.7 Adverse Events Analysis:

All treatment-emergent adverse events (TEAE) will be summarized. Counts and percents of each observed system organ class (SOC) and preferred term as defined in the Medical Dictionary for Regulatory Activities (MedDRA) version 9.0 or later will be presented. The preferred terms will be summarized in the following sets of tables:

- Baseline AEs, occurring after enrolment and prior to treatment;
- All TEAEs;
- All serious TEAEs;
- All TEAE leading to discontinuation from study
- All TEAEs by closest relationship (related or not related). Relationship will be recorded as definitely related, probably related, possibly related, or not related to the study medication; the recorded relationship will be included in listings.

Reported severity (mild, moderate or severe) will be included in the listing. The severity of the TEAEs will be graded according to the CTCAE criteria, version 4.0 (Appendix 3), A separate table for AE of CTCAE grade greater than 2 (moderate) will be generated. The severity of oral mucositis will be graded according to the WHO grade.

A subject having the same TEAE (preferred term) more than once over the course of the study will be counted only once in the incidence calculation for that preferred term. Similarly, if a subject had more than one TEAE in a single SOC, the subject will be counted only once in the incidence calculation for that SOC. If a subject had a TEAE more than once in the study, the occurrence with the maximum severity will be used in the calculation of the incidence of individual TEAE by severity. If a subject had a TEAE more than once in the study, the occurrence with the highest relationship to treatment will be used in the calculation of the incidence of individual TEAE by relationship.

Adverse Events Rows 15

ID	Reported Term (Verbatim)	AE start date	AE stop date	Serious AE?	Status
1831	Hyperglycemia	09-Feb-2019	10-Feb-2019	No	(3)
1859	Cardiac Chest Pain during Stress ECHO	31-Jan-2019	31-Jan-2019	No	(3)

1 2

4.7.1 Deaths, Other Serious Adverse Events (SAEs), and Other Significant AEs Analysis:

Listings will be provided for

- Deaths,
- SAEs,
- Discontinuations due to TEAEs,
- Grade 4 AEs, and
- Related AEs.

4.8 Pharmacokinetics and Biodistribution Assessment

4.8.1 Pharmacokinetics Analysis:

Whole blood samples will be assessed for PK parameters and maximum concentration (C_{max}) during treatment period will be determined. PK parameters will be derived from the platinum concentrations obtained from Excite Pharma Services. Tumor and lymph node cis platinum content will be provided as well.

Summary statistics for PK data will be displayed for the following category: :

1. Tumor tissue
2. Lymph nodes
3. Blood
4. Residual Patch

C_{max} based on blood concentrations across patch treatment period as well as other summary statistics for each patient will be listed.

Systemic, tumor and lymph node (if available) platinum levels will be summarized using descriptive statistics. Analysis will be adjusted based on the number of patches applied per application (1 or 2) and total number of treatment visits.

5 Data Analysis and Statistical Considerations

Statistical analyses and summaries will be provided when all corresponding data is collected and processed, and the corresponding database portions are locked through this stage of the

study. All available data from post-operative visits for other subjects, if any, will be included. The final analysis including all available post-operative data will be performed when the last subject has reached the 6-month post-operative follow-up visit and the final database is locked.

Statistical tables, data listings and graphs will be prepared using SAS Version 9.1 or higher (SAS Institute, Cary, North Carolina, USA).

5.1 Statistical Analysis

Safety outcomes resulting in DLTs will be used during the conduct of this study to select doses being tested according to pre-defined thresholds of toxicity.

- Local and systemic safety and tolerability will be summarized using descriptive statistics.
- The number of subjects with DLTs will be capped to be fewer than 2 subjects with DLTs out of 5 new subjects enrolled.
- The treatment regimen will be deemed tolerable if fewer than a third of the subjects exposed to it present DLTs.
- Efficacy based on Tumor volume CFB criteria will be summarized, and the null hypothesis that the probability of response is at most 30%, where a response is the occurrence of CR or PR will be tested using a one-sided test exact binomial test at level 0.018.
- Other efficacy assessments such as change in tumor volume or tumor histology will be summarized using descriptive statistics.
- Variables measuring technical success (including residual cisplatin level in the patch) will be summarized using descriptive statistics.

At the end of Stage 2, the following is a statistical analysis of the sample size as it pertains to efficacy:

Power and Sample Size

The planned enrollment for the final dose is intended to obtain N=11 evaluable subjects.

Based on Simon's 2-stage procedure, the null hypothesis that the true response rate is 0.30 will be tested against a 1-sided alternative. In the first stage, 5 evaluable subjects will be accrued. If there are 2 or fewer responses in these 5 subjects, the dose will be escalated. Otherwise, the dose will remain the same or de-escalated based on safety data. Additional subjects will be accrued for a total of 11 evaluable subjects at the final dose. The null hypothesis will be rejected if 7 or more responses are observed in the 11 evaluable subjects at the final dose. This design yields a type I error rate of 0.018 and power of 96.44% when the true response rate is 0.65.

Null response rate: P_0	Alt. response rate: P_1	Alpha one-tailed	Power	n1	r1	N	R
0.30	0.85	0.018	96.44%	5	2	11	6

N is the total number of evaluable subjects at the final dose
n_1 is the number of evaluable subjects accrued during Stage 1
r_1, if r_1 or fewer responses are observed during stage 1, escalate the dose if safety requirements are met
R, if *R* or fewer responses are observed by the end of Stage 2 in N subjects, then an effective dose is not found

Note: If the dose is not increased after Stage 1, the test will be performed using all evaluable subjects from Stage 1 and 2. If the dose is adjusted after Stage 1, the test will be performed using all evaluable subjects from Stage 2.

5.2 Treatment Analysis Guidelines

5.2.1 Efficacy Analyses

The primary efficacy analysis will be conducted after all evaluable subjects will have undergone their surgical ablation of the oral tumor, in addition to the interim analysis described below.
Note: All hypothesis tests will be performed at a 0.018 level.

5.2.2 Evaluable Population

All subjects who have **met the inclusion and exclusion criteria**, and have completed at least 75% of the total dose required for that stage will be included in the Evaluable Population*. This population will be used for the efficacy analysis. **Only subjects in the evaluable population will be assessed for tumor response.**

* 75% is defined as at least 3/4 patch application visits completed

5.2.3 Baseline Values for Tumor Measurement

The baseline values for each subject will be those recorded at Visit 1. If the value at the Baseline visit is missing, then the value from the earliest available visit will be used. For tumor measurements, if the Visit 1 measurement is missing, then the measurements from the screening CT scan will be used as baseline values.

5.2.4 Change from Baseline (CFB) and Percent Change from Baseline (PCFB)

CFB will be calculated at each post-baseline visit for all evaluable subjects as:

CFB = Post-Baseline value – Baseline value.

CFB will be reported to the same number of decimal places as the variable.

PCFB will be calculated at each post-baseline visit for all evaluable subjects as:

PCFB = 100*CFB/ Baseline value

= 100*(Post-Baseline value – Baseline value) /Baseline value.

PCFB will be reported to same number of decimal places as the variable.

5.2.5 Descriptive Summary Statistics

The following standards will be applied for each analysis unless otherwise specified. Simple summary statistics for continuous data are defined as: n (number of non-missing observations), mean, standard deviation, maximum, median, minimum. The number and percentage of subjects will be used to summarize categorical data. All data will be listed and sorted by investigator number, variable, subject number, and visit, if appropriate.

5.3 Safety Analyses

The Safety Population will be used for all summaries of safety data.
will be used in the calculation of the incidence of individual TEAE by relationship.

5.3.1 Safety Population

All subjects who have at least 1 PRV111 patch applied will be included in the Safety Population.

5.3.2 Concomitant Medication and Therapy

A listing of concomitant medications and therapy will be provided for the Safety Population.

5.3.3 Medical History, Medication History and Baseline Disease Characteristics

Medical history and baseline disease characteristics will be listed for the Safety Population.
ECOG PS (Appendix 2) will be included.

5.3.4 Subject Accountability, Demographics, and Baseline Characteristics

Subject completion status, dose modification, and study terminations will be summarized for each of the study stages and for the study overall. Subjects with major protocol violations will be listed. Demographics will be summarized along with baseline characteristics such as medical history, and baseline physical examination, weight, VS, ECOG PS, and concomitant therapy.

6 Data Handling

6.1 Pooling of Centers

This is a multi-center study. The data from all sites will be pooled for all analyses.

6.2 Handling of Missing Data

All missing data will be queried. Missing efficacy data which are not retrievable through queries will be imputed. Missing safety data will not be imputed. If the value of a vital sign assessment, ECG variable, laboratory value, or clinical tumor measurement is missing at Baseline, then CFB will be defined as the change from the value recorded at the next earliest available data point.

Non-Responder Imputation (NRI)

No missing values imputation will be applied.

6.3 Drop-outs, Withdrawals, Replacement Policy

If a subject withdraws from the study at any time, either at his or her request or at the Investigator’s discretion, the reason(s) for withdrawal will be recorded on the relevant page of the CRF. Subjects withdrawn due to AEs will be monitored until resolution of all AEs. Subjects who withdraw from dosing will be not replaced. However, their data will still be used if they are considered part of the evaluable population, unless they state otherwise.

7 Changes from the Protocol

No changes from the protocol are planned.

8 Appendices

8.1 Appendix 1: Laboratory Values Collected

Lab Test_Baylor Site 001 Austin ranges

Test name	Gender	low range	high range	unit	effective start date	effective end date
White Blood Cell Count	B	4	11	K/UL		
Red Blood Cell Count	M	4.1	5.7	M/UL		
Red Blood Cell Count	F	3.8	5.1	M/UL		
Hemoglobin	M	13	17	G/DL		
Hemoglobin	F	11.5	15.5	G/DL		
Hematocrit	M	37	49	%		
Hematocrit	F	34	45	%		
Platelet Count	B	130	400	K/UL		
Neutrophils	B	40	74	%		
Lymphocytes	B	19	48	%		
Monocytes	B	4	13	%		
Eosinophils	B	0	7	%		
Basophils	B	0	2	%		
Reticulocyte	B	0.5	2.3	%		
Neutrophil Absolute Count	B	1500	7000	Per UL		
Lymphocyte Absolute Count	B	1000	4000	Per UL		
Monocytes Absolute Count	B	100	1000	Per UL		
Eosinophils Absolute Count	B	0	500	Per UL		
Basophils Absolute Count	B	0	200	Per UL		
Reticulocyte Absolute Count	B	27	118	K/UL		
Albumin Level	B	3.5	5.2	G/DL		
Alkaline Phosphatase	M	39	118	U/L		
Alkaline Phosphatase	F	30	146	U/L		
BUN	B	8	23	MG/DL		
Calcium	B	8.5	10.5	MG/DL		
Creatinine	M	0.8	1.4	MG/DL		
Creatinine	F	0.6	1.3	MG/DL		
Calculated creatinine						
Glucose	B	70	99	MG/DL		
Protein Total	B	6.1	8.3	G/DL		
AST(SGOT)	M	9	50	U/L		
AST(SGOT)	F	9	40	U/L		
ALT(SGPT)	M	5	50	U/L		

ALT(SGPT)	F	5	40	U/L		
Bilirubin Direct	B	0	0.3	MG/DL		
Bilirubin Total	B	0	1.2	MG/DL		
Sodium	B	133	146	MEQ/L		
Potassium	B	3.5	5.4	MEQ/L		
Chloride	B	95	107	MEQ/L		
Magnesium	B	1.6	2.6	MG/DL		
CO2	B	19	31	MEQ/L		
GGT	B	7	70	U/L		
Color	B	Yellow/Straw				
Appearance	B	Clear				
Protein	B	Negative				
Glucose(urine)	B	Negative				
Ketone	B	Negative				
Bilirubin	B	Negative				
Occult blood	B	Negative				
Nitrite	B	Negative				
Leukocytes	B	Negative				
Urobilinogen	B	0	1.999	MG/DL		
Specific Gravity	B	1.005	1.035			
PH	B	5	9			
RBC	B	0	5	/HPF		
WBC	B	0	5	/HPF		

Site 001-001 Baylor HHS Ranges

Test name	Gender	low range	high range	unit	effective start date	effective end date
White Blood Cell Count	M	8	15.4	K/uL		
White Blood Cell Count	F	8.2	16.6	K/uL		
Red Blood Cell Count	M	4.1	5.55	M/uL		
Red Blood Cell Count	F	4.12	5.74	M/uL		
Hemoglobin	M	13.9	19.1	g/dL		
Hemoglobin	F	13.4	20	g/dL		
Hematocrit	M	39.8	53.6	%		

Hematocrit	F	39.6	57.2	%		
Platelet Count	M	218	419	K/uL		
Platelet Count	F	144	449	K/uL		
Neutrophils	M	20.2	46.2	%		
Neutrophils	F	15.2	66.1	%		
Lymphocytes	M	33.7	67.6	%		
Lymphocytes	F	24.9	68.5	%		
Monocytes	M	6.7	19.9	%		
Monocytes	F	5.2	20.6	%		
Eosinophils	M	0.3	5.2	%		
Eosinophils	F	0.4	4.6	%		
Basophils	M	0.1	0.8	%		
Basophils	F	0.1	0.6	%		
Neutrophil Absolute Count	M	1.6	6.06	K/uL		
Neutrophil Absolute Count	F	1.73	6.75	K/uL		
Lymphocyte Absolute Count	M	2.07	7.53	K/uL		
Lymphocyte Absolute Count	F	1.75	8	K/uL		
Monocytes Absolute Count	M	0.52	1.77	K/uL		
Monocytes Absolute Count	F	0.57	1.72	K/uL		
Eosinophils Absolute Count	M	0.12	0.66	K/uL		
Eosinophils Absolute Count	F	0.09	0.64	K/uL		
Basophils Absolute Count	M	0.02	0.11	K/uL		
Basophils Absolute Count	F	0.02	0.07	K/uL		
Albumin Level	B					
Alkaline Phosphatase	B	80	350	U/L		
BUN	B	7	18	mg/dL		
Calcium	B	8.5	10.2	mg/dL		
Creatinine	B	0.6	1.3	mg/dL		
Glucose	B	70	99	mg/dL		
Protein Total	B					
AST(SGOT)	B	15	37	U/L		
ALT(SGPT)	B	12	78	U/L		

Bilirubin Direct	B	0	0.3	mg/dL		
Bilirubin Total	B	0.2	1	mg/dL		
Sodium	B	136	145	mmol/		
Potassium	B	3.5	5.1	mmol/		
Chloride	B	98	107	mmol/		
Magnesium	B	1.8	2.4	mg/dL		
CO2	B	18	27	mmol/		
GGT	B	5	85	U/L		
Color	B					
Appearance	B					
Protein	B	Negative				
Glucose(urine)	B	Negative				
Ketone	B	Negative				
Bilirubin	B	Negative				
Occult blood	B	Negative				
Nitrite	B	Negative				
Leukocytes	B	Negative				
Urobilinogen	B	0.2	1 EU/dL			
Specific Gravity	B	1.001	1.035			
PH	B	5	8			
RBC	B	0	4 /HPF			
WBC	B	0	5 /HPF			

Site 001-001 Baylor-Houston

Test name	Gender	low range	high range	unit	effective start date	effective end date
White Blood Cell Count	M	8	15.4	K/uL		
White Blood Cell Count	F	8.2	16.6	K/uL		
Red Blood Cell Count	M	4.1	5.55	M/uL		
Red Blood Cell Count	F	4.12	5.74	M/uL		
Hemoglobin	M	13.9	19.1	g/dL		
Hemoglobin	F	13.4	20	g/dL		
Hematocrit	M	39.8	53.6	%		
Hematocrit	F	39.6	57.2	%		
Platelet Count	M	218	419	K/uL		
Platelet Count	F	144	449	K/uL		
Neutrophils	M	20.2	46.2	%		
Neutrophils	F	15.2	66.1	%		
Lymphocytes	M	33.7	67.6	%		
Lymphocytes	F	24.9	68.5	%		
Monocytes	M	6.7	19.9	%		
Monocytes	F	5.2	20.6	%		
Eosinophils	M	0.3	5.2	%		
Eosinophils	F	0.4	4.6	%		
Basophils	M	0.1	0.8	%		
Basophils	F	0.1	0.6	%		
Neutrophil Absolute Count	M	1.6	6.06	K/uL		
Neutrophil Absolute Count	F	1.73	6.75	K/uL		
Lymphocyte Absolute Count	M	2.07	7.53	K/uL		
Lymphocyte Absolute Count	F	1.75	8	K/uL		
Monocytes Absolute Count	M	0.52	1.77	K/uL		
Monocytes Absolute Count	F	0.57	1.72	K/uL		
Eosinophils Absolute Count	M	0.12	0.66	K/uL		
Eosinophils Absolute Count	F	0.09	0.64	K/uL		
Basophils Absolute Count	M	0.02	0.11	K/uL		

Basophils Absolute Count	F	0.02	0.07	K/uL		
Albumin Level	B					
Alkaline Phosphatase	B	80	350	U/L		
BUN	B	7	18	mg/dL		
Calcium	B	8.5	10.2	mg/dL		
Creatinine	B	0.6	1.3	mg/dL		
Glucose	B	70	99	mg/dL		
Protein Total	B					
AST(SGOT)	B	15	37	U/L		
ALT(SGPT)	B	12	78	U/L		
Bilirubin Direct	B	0	0.3	mg/dL		
Bilirubin Total	B	0.2	1	mg/dL		
Sodium	B	136	145	mmol/		
Potassium	B	3.5	5.1	mmol/		
Chloride	B	98	107	mmol/		
Color	B	Yellow/Straw				
Appearance	B	Clear				
Protein	B	Negative				
Glucose(urine)	B	Negative				
Ketone	B	Negative				
Bilirubin	B	Negative				
Occult blood	B	Negative				
Nitrite	B	Negative				
Leukocytes	B	Negative				
Urobilinogen	B	0	1.999	MG/DL		
Specific Gravity	B	1.005	1.035			
PH	B	5	9			
RBC	B	0	5	/HPF		
WBC	B	0	5	/HPF		

Site 001-001 Baylor-Methodist

Test name	Gender	low range	high range	unit	effective start date	effective end date
White Blood Cell Count	B	4.5	11	K/µL		
Red Blood Cell Count	M	4.4	6	M/µL		
Red Blood Cell Count	F	4.2	5.5	M/µL		
Hemoglobin	M	14	18	g/dL		
Hemoglobin	F	12	16	g/dL		
Hematocrit	M	41	51	%		
Hematocrit	F	37	47	%		
Platelet Count	B	150	400	K/µL		
Neutrophils	B	39	69	%		
Lymphocytes	B	25	45	%		
Monocytes	B	0	10	%		
Eosinophils	B	0	5	%		
Basophils	B	0	1	%		
Neutrophil Absolute Count	B					
Lymphocyte Absolute Count	B					
Monocytes Absolute Count	B					
Eosinophils Absolute Count	B					
Basophils Absolute Count	B					
Albumin Level	B	3.5	5	g/dL		
Alkaline Phosphatase	B	30	115	U/L		
BUN	B	8	24	mg/dL		
Calcium	B	8.6	10.6	mg/dL		
Creatinine	B	0.5	1.5	mg/dL		
Glucose	B	65	110	mg/dL		
Protein Total	B	6.3	8.2	g/dL		
AST(SGOT)	B	15	46	U/L		
ALT(SGPT)	B	10	55	U/L		
Bilirubin Total	B	0.2	1.2	mg/dL		
Sodium	B	135	148	mEq/L		
Potassium	B	3.5	5	mEq/L		
Chloride	B	96	111	mEq/L		
Magnesium	B					
CO2	B	24	31	mEq/L		
GGT	B					
Color	B					
Appearance	B					
Protein	B	Negative				

Glucose(urine)	B	Negative				
Ketone	B	Negative				
Bilirubin	B					
Occult blood	B	Negative				
Nitrite	B	Negative				
Leukocytes	B					
Urobilinogen	B	0.1	1			
Specific Gravity	B	1.005	1.03			
PH	B	4.5	8			
RBC	B					
WBC	B					

Site 002 University of Texas

Test name	Gender	low range	high range	unit	effective start date	effective end date
White Blood Cell Count	B	3.7	10.4	K/CMM		
Red Blood Cell Count	M	4.7	6.1	M/CMM		
Red Blood Cell Count	F	4.2	5.4	M/CMM		
Hemoglobin	M	14	18	g/dL		
Hemoglobin	F	12	16	g/dL		
Hematocrit	M	42	54	%		
Hematocrit	F	36	48	%		
Platelet Count	B	133	450	K/CMM		
Neutrophils	B	45	75	%		
Lymphocytes	B	20	40	%		
Monocytes	B	2	12	%		
Eosinophils	B	0	4	%		
Basophils	B	0	1	%		
Neutrophil Absolute Count	B	1.5	8.1			
Lymphocyte Absolute Count	B	1	5.5			
Monocytes Absolute Count	B	0	0.8			
Eosinophils Absolute Count	B					
Basophils Absolute Count	B	0	0.2			
Albumin Level	B	3.5	5	g/dL		
Alkaline Phosphatase	B	39	136	U/L		
BUN	B	7	22	mg/dL		
Calcium	B	8.5	10.5	mg/dL		
Creatinine	B	0.5	1.4	mg/dL		
Glucose	B	70	99	mg/dL		

Protein Total	B	6.4	8.4	g/dL		
AST(SGOT)	B	0	37	U/L		
ALT(SGPT)	B	0	65	U/L		
Bilirubin Total	B	0.2	1.3	mg/dL		
Sodium	B	135	145	mEq/L		
Potassium	B	3.5	5.1	mEq/L		
Chloride	B	95	109	mEq/L		
Magnesium	B					
CO2	B	24	32	mEq/L		
GGT	B					
Color	B					
Appearance	B					
Protein	B	Negative				
Glucose(urine)	B	Negative				
Ketone	B	Negative				
Bilirubin	B	Negative				
Occult blood	B	Negative				
Nitrite	B	Negative				
Leukocytes	B	Negative				
Urobilinogen	B	0.1	1			
Specific Gravity	B	0	1.0299			
PH	B	4.5	8			
RBC	B	0	2	/HPF		
WBC	B	0	5	/HPF		

Site 003 University of Cincinnati

Test name	Gender	low range	high range	unit	effective start date	effective end date
White Blood Cell Count	B	3.8	10.8	10*3/µL		
Red Blood Cell Count	M	4.2	5.8	10*6/µL		
Red Blood Cell Count	F	3.8	5.1	10*6/µL		
Hemoglobin	M	13.2	17.1	g/dL		
Hemoglobin	F	11.7	15.5	g/dL		
Hematocrit	M	38.5	50	%		
Hematocrit	F	35	45	%		
Platelet Count	B	140	400	Thous/µL		
Neutrophils	B	40	80	%		
Lymphocytes	B	15	45	%		
Monocytes	B	0	12	%		

Eosinophils	B	0	8	%		
Basophils	B	0	1	%		
Neutrophil Absolute Count	B	1500	7800	uL		
Lymphocyte Absolute Count	B	850	3900	uL		
Monocytes Absolute Count	B	200	950	uL		
Eosinophils Absolute Count	B	15	500	uL		
Basophils Absolute Count	B	0	200	uL		
Albumin Level	B	3.5	5.7	g/dL		
Alkaline Phosphatase	B	36	125	U/L		
BUN	B	7	25	mg/dL		
Calcium	B	8.6	10.3	mg/dL		
Creatinine	B	0.6	1.3	mg/dL		
Glucose	B	70	100	mg/dL		
Protein Total	B	6.4	8.9	g/dL		
AST(SGOT)	B	13	39	U/L		
ALT(SGPT)	B	7	52	U/L		
Bilirubin Total	B	0	1.5	mg/dL		
Sodium	B	133	146	mmol/L		
Potassium	B	3.5	5.3	mmol/L		
Chloride	B	98	110	mmol/L		
Magnesium	B	1.5	2.5	mg/dL		
CO2	B	21	33	mmol/L		
GGT	B	9	64	U/L		
Color	B	Yellow, Straw				
Appearance	B	Clear				
Protein	B	Negative				
Glucose(urine)	B	Negative				
Ketone	B	Negative				
Bilirubin	B	Negative				
Occult blood	B	Negative				
Nitrite	B	Negative				
Leukocytes	B	Negative				
Urobilinogen	B	0.2	1	E.U./dL		
Specific Gravity	B	1.005	1.035			
PH	B	5	8			
RBC	B	0	3	hpf		
WBC	B	0	5	hpf		

Site 004 Advanced ENT and Allergy

Test name Gender	Min Age	Max Age	low range high	range unit	
White Bloo B	0	999	3.4	10.8 x10E3/uL	
Red Blood M	0	999	4.14	5.8 x10E6/uL	
Red Blood F	0	999	3.77	5.28 x10E6/uL	
Hemoglobi M	0	999	13	17.7 g/dL	
Hemoglobi F	0	999	11.1	15.9 g/dL	
HematocritM	0	999	37.5	51 %	
HematocritF	0	999	34	46.6 %	
Platelet Co B	0	999	150	379 x10E3/uL	
Reticulocyt B	0	999	0.6	2.6 %	
Neutrophil B	0	999	1.4	7 x10E3/uL	
Lymphocyt B	0	999	0.7	3.1 x10E3/uL	
Monocytes B	0	999	0.1	0.9 x10E3/uL	
Eosinophils B	0	999	0	0.4 x10E3/uL	
Basophils AB	0	999	0	0.2 x10E3/uL	
Albumin Le B	4	60	3.5	5.5 g/dL	
Albumin Le B	61	70	3.6	4.8 g/dL	
Albumin Le B	71	80	3.5	4.8 g/dL	
Albumin Le B	81	90	3.5	4.7 g/dL	
Albumin Le B	91	999	3.2	4.6 g/dL	
Alkaline Ph M	18	18	56	127 IU/L	
Alkaline Ph F	18	18	43	101 IU/L	
Alkaline Ph B	19	999	39	117 IU/L	
BUN B	18	39	6	20 mg/dL	
BUN B	40	59	6	24 mg/dL	
BUN B	60	89	8	27 mg/dL	
BUN B	90	999	10	36 mg/dL	
Calcium B	18	59	8.7	10.2 mg/dL	
Calcium M	60	999	8.6	10.2 mg/dL	
Calcium F	60	999	8.7	10.3 mg/dL	
Creatinine M	0	999	0.76	1.27 mg/dL	
Creatinine F	0	999	0.57	1 mg/dL	
Creatinine M	0	999	97	137 mL/min	
Creatinine F	0	999	88	128 mL/min	
Glucose B	0	999	65	99 mg/dL	
Protein Tot B	0	999	6	8.5 g/dL	
AST(SGOT) B	0	999	0	40 IU/L	
ALT(SGPT) M	0	999	0	44 IU/L	
ALT(SGPT) F	0	999	0	32 IU/L	

Bilirubin ToB	0	999	0	1.2 mg/dL
Bilirubin Di B	0	999	0	0.4 mg/dL
Sodium B	0	999	134	144 mmol/L
Potassium B	0	999	3.5	5.2 mmol/L
Chloride B	0	999	97	106 mmol/L
MagnesiumB	0	999	1.6	2.3 mg/dL
CO2 B	0	999	20	29 mmol/L
GGT M	0	999	0	65 IU/L
GGT	F	0	999	0
Color	B	0	999	Yellow
Appearanc	B	0	999	Clear
Leukocytes	B	0	999	Negative
Nitrite	B	0	999	Negative
Protein	B	0	999	Negative/Trace
Glucose(ur	B	0	999	Negative
Occult bloo	B	0	999	Negative
Ketone	B	0	999	Negative
Bilirubin	B	0	999	Negative
Urobilinog	B	0	999	0.2
PH	B	0	999	5
Specific Gr	B	0	999	1.005
RBC	B	0	999	0
WBC	B	0	999	0

8.2 Appendix 2: ECOG Performance Status (ECOG PS)

Performance status will be assessed using ECOG PS at Baseline for inclusion criterion.

**Grade**	**ECOG PS**
0	Fully active, able to carry on all pre-disease performance without restriction
1	Restricted in physically strenuous activity but ambulatory and able to carry out work of a light or sedentary nature, e.g., light house work, office work
2	Ambulatory and capable of all self-care but unable to carry out any work activities; up and about more than 50% of waking hours
3	Capable of only limited self-care; confined to bed or chair more than 50% of waking hours
4	Completely disabled; cannot carry on any self-care; totally confined to bed or chair
5	Dead

8.3 Appendix 3: Common Terminology Criteria for Adverse Events (CTCAE)

CTCAE Grade refers to the severity of the AE. The CTCAE displays Grades 1 through 5 with unique clinical descriptions of severity for each AE based on this general guideline:

Grade	Severity Description
1	Mild; asymptomatic or mild symptoms; clinical or diagnostic observations only; intervention not indicated.
2	Moderate; minimal, local or noninvasive intervention indicated; limiting age-appropriate instrumental ADL*.
3	Severe or medically significant but not immediately life-threatening; hospitalization or prolongation of hospitalization indicated; disabling; limiting self-care ADL**.
4	Life-threatening consequences; urgent intervention indicated.
5	Death related to AE

*Instrumental ADL refer to preparing meals, shopping for groceries or clothes, using the telephone, managing money, etc.

**Self-care ADL refer to bathing, dressing and undressing, feeding self, using the toilet, taking medications, and not bedridden.
